# Supplementary material for: Development of culturally sensitive pain neuroscience education materials for Hausa-speaking patients with chronic spinal pain: A modified Delphi study
Source: PLoS One. 2021 Jul 2;16(7):e0253757. doi: 10.1371/journal.pone.0253757 (PMC8253446; doi:10.1371/journal.pone.0253757)
Supplement: S1 File — (PDF) [file pone.0253757.s001.pdf]

BARKAN KU DA ZUWA!

PAIN IN MOTION

200 YEARS
GHENT UNIVERSITY

FACULTY OF MEDICINE AND HEALTH SCIENCES

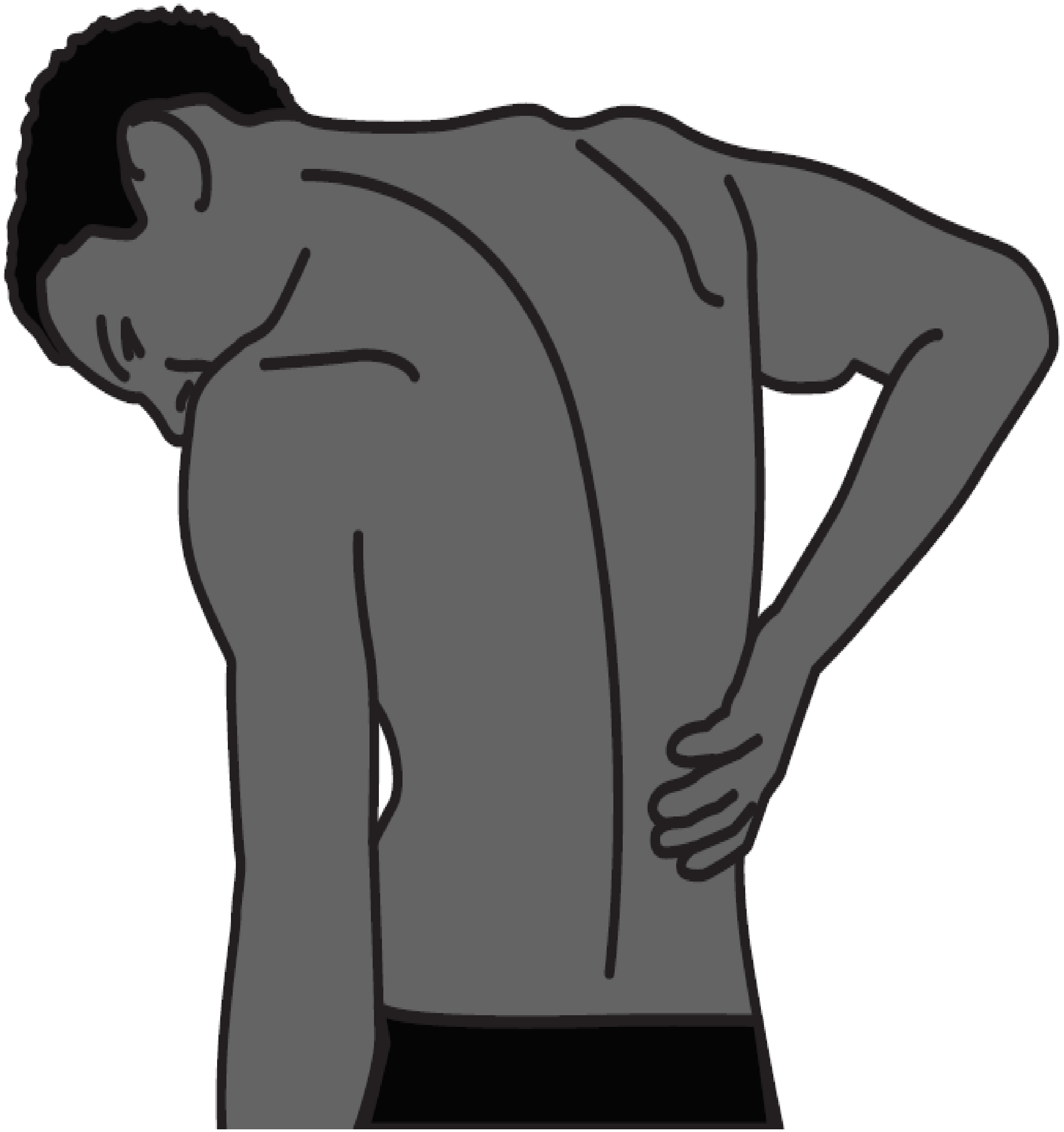

CIWO

MUHIMMAN BAYANAI GAME DA CIWO

Anya ciwo yana da amfani kuwa? da sannu zamu samu bayanai masu amfani akan ciwon da ke tattare da mu.

CIWO

- Ma’anar sa?
- Sababin sa?
- Manufar sa?
- Cin gajiyar sa?

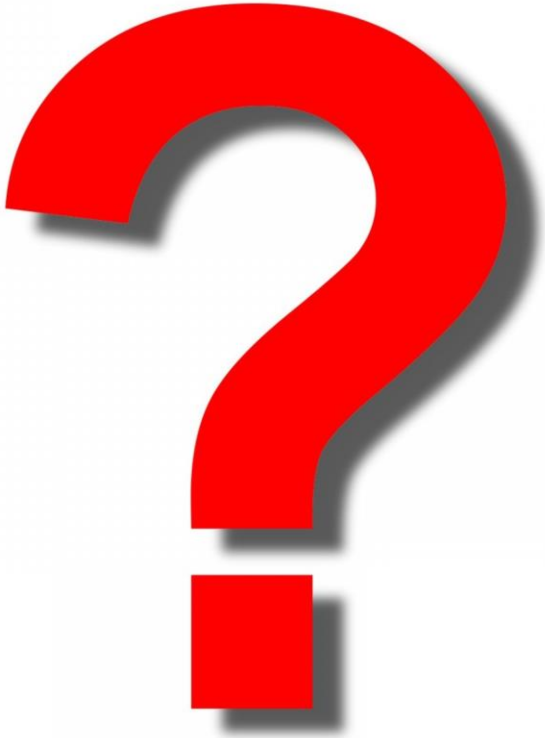

CIWO

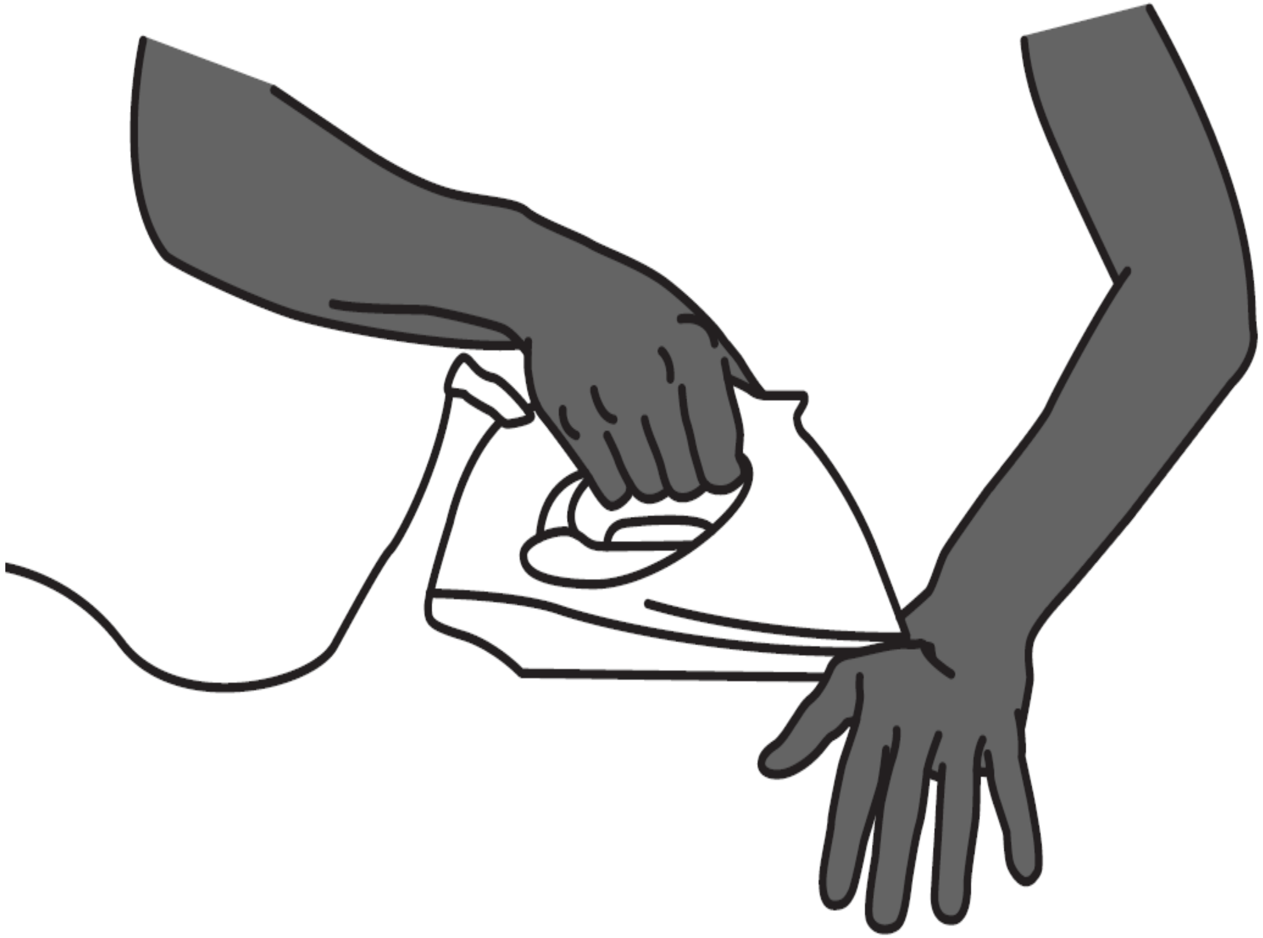

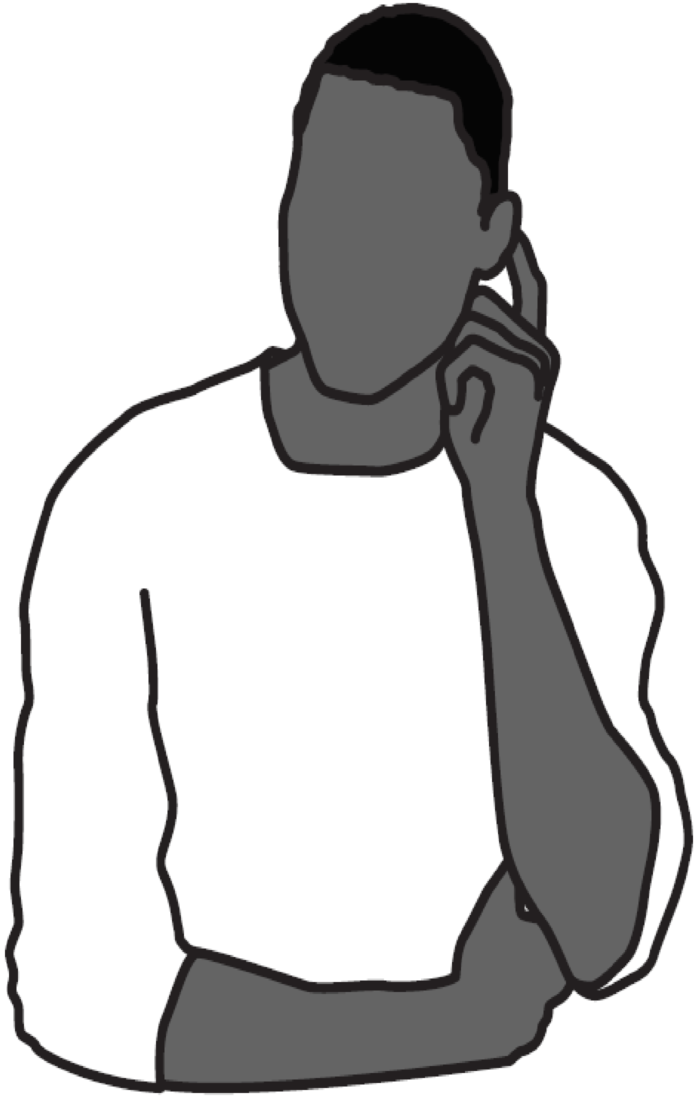

CIWO
(Cin gajiyar sa)

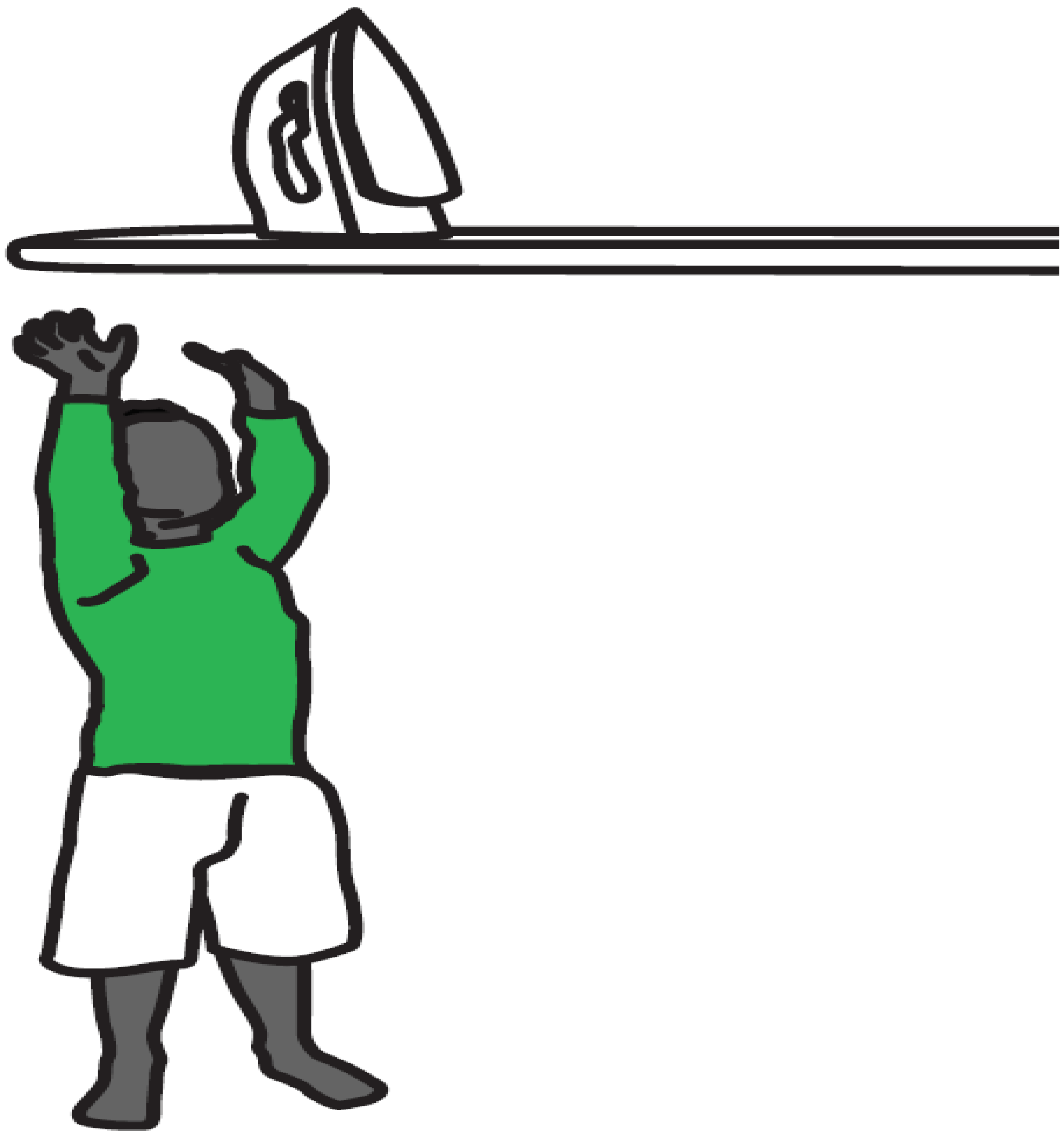

Menene Ciwo?

Wata hanya ce ta tun fil azal da ke baiwa jikin mu kariya

Tana sanar da jikin mu idan akwai barazana ko hatsari

Jiki kuma sai ya ɗauki mataki

TSIRA

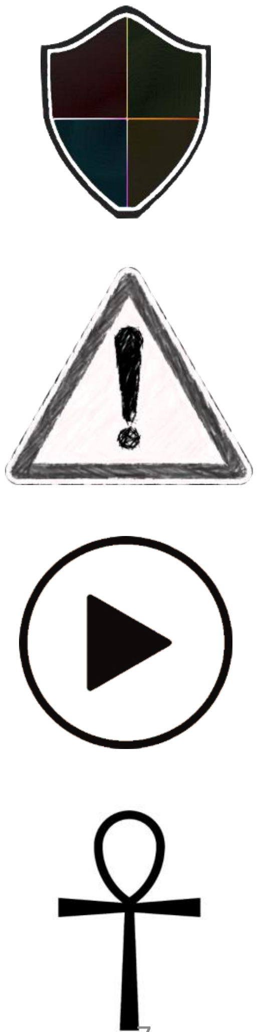

CIWO WATA KARARRAWA CE GA JIKIN MU

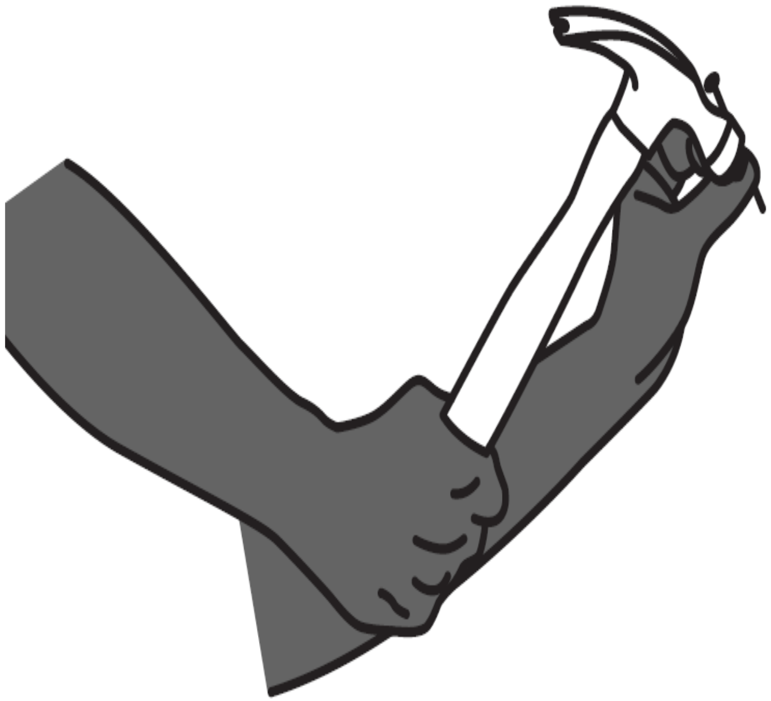

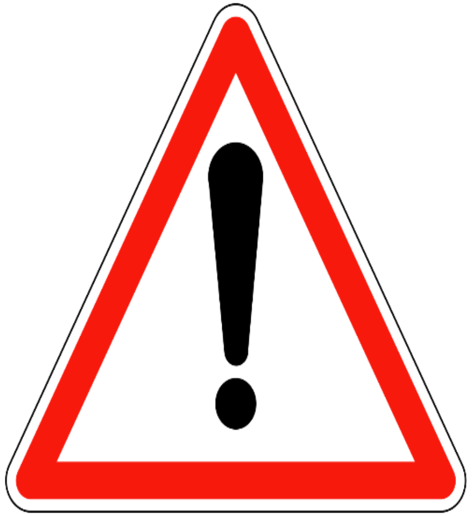

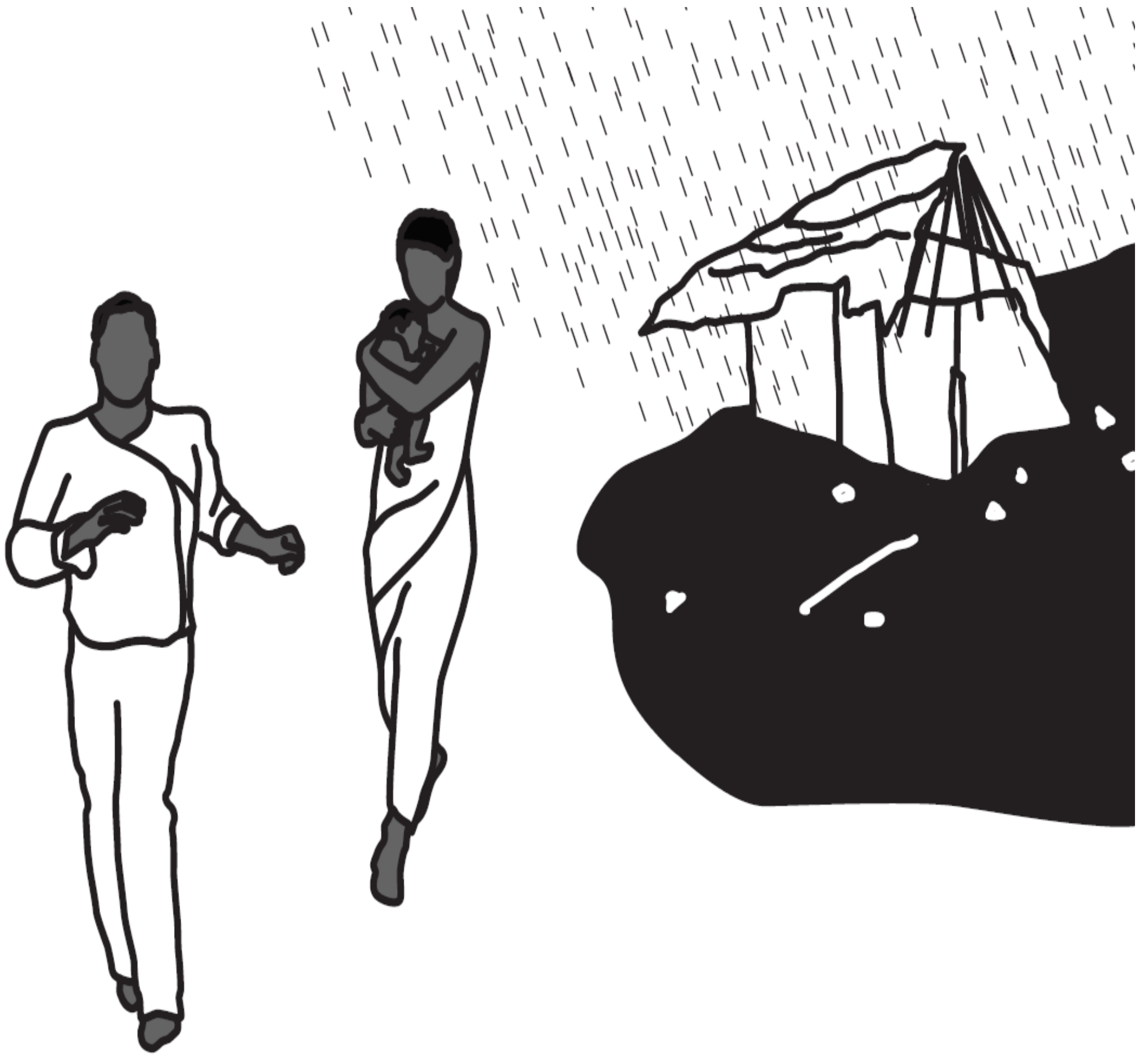

CIWO
sanarwa ce ga jikin mu

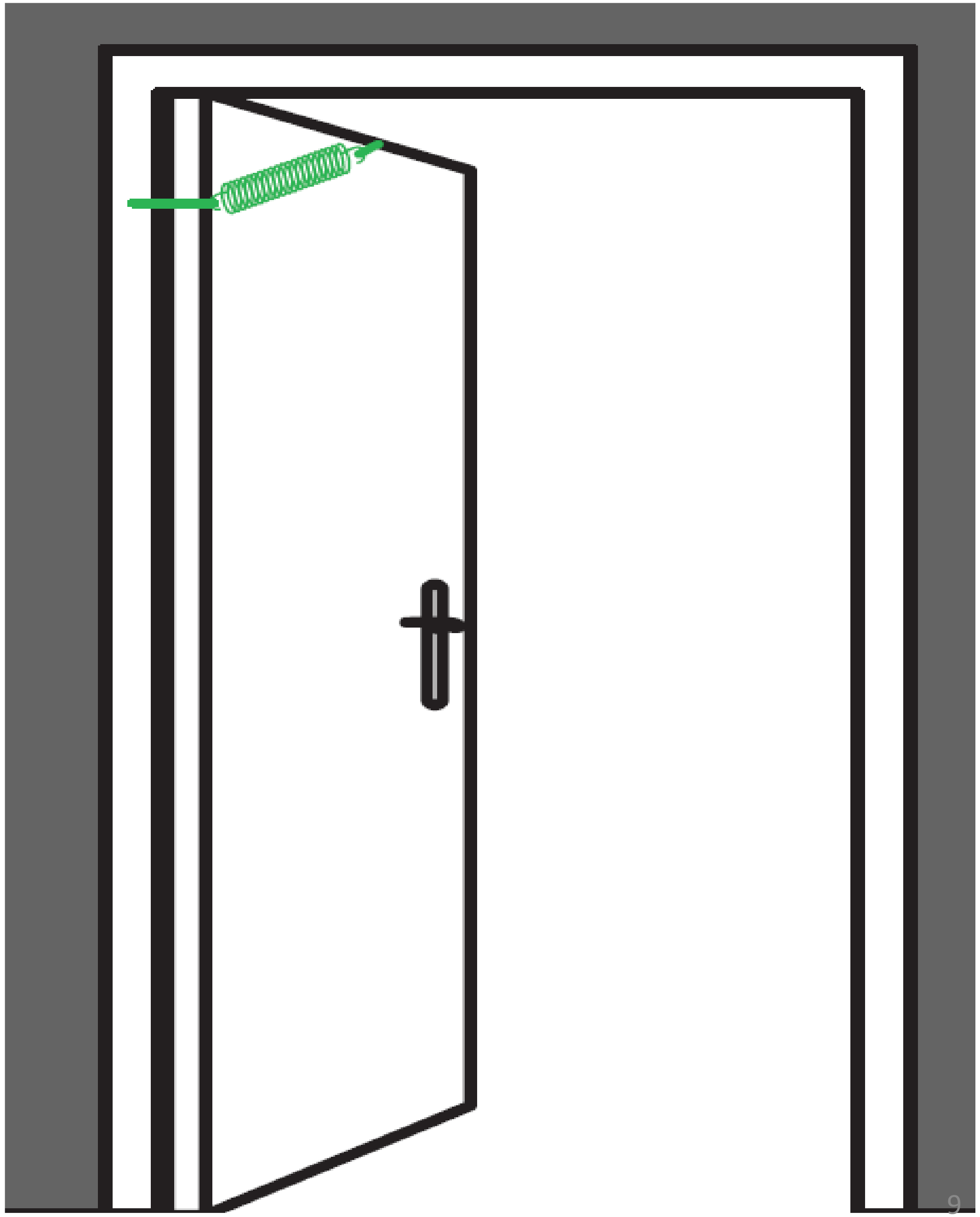

ASHE DAI CIWO NA DA AMFANI!

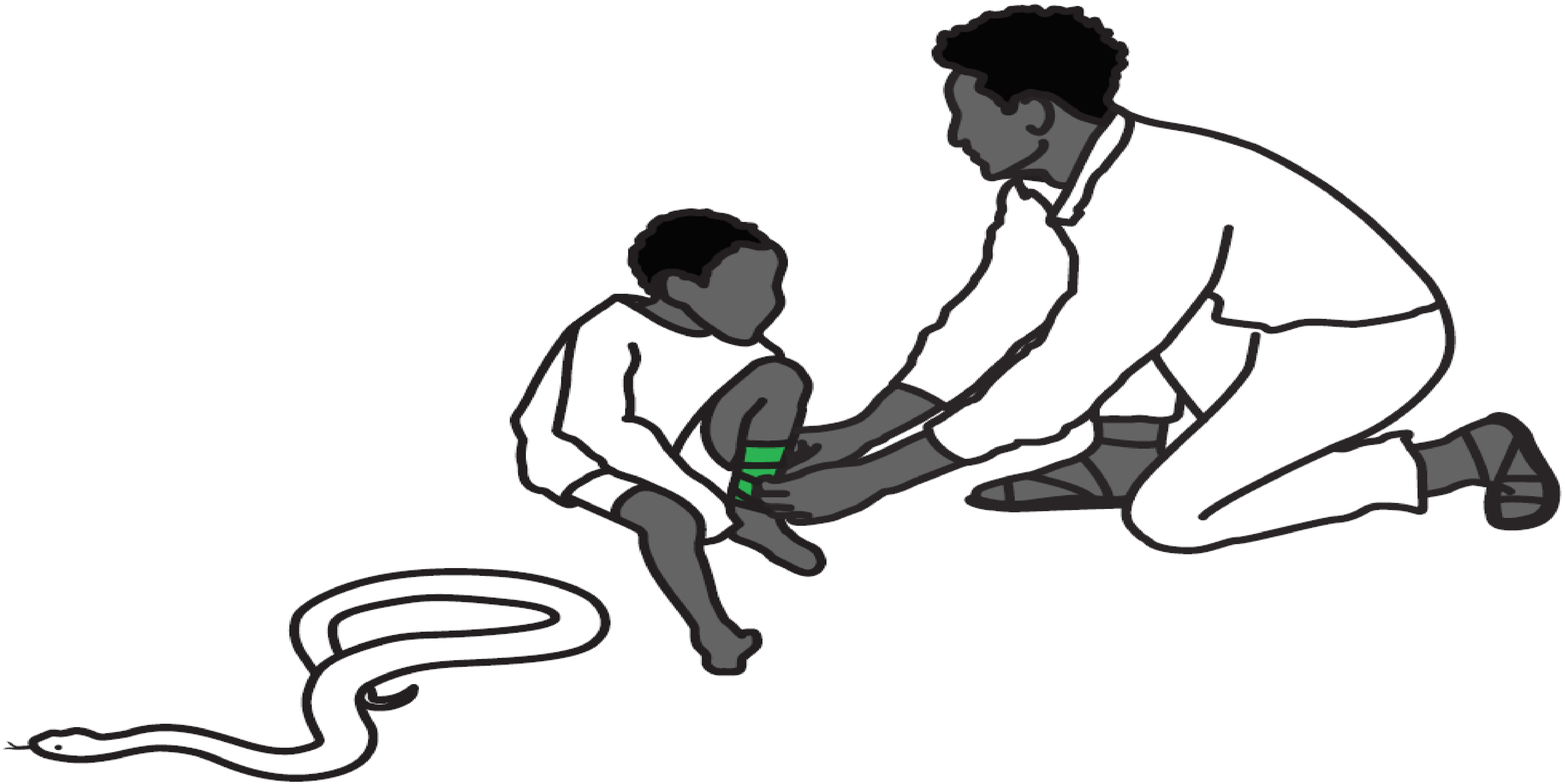

HATSARIN DA KE TATTARE DA RASHIN JIN CIWO

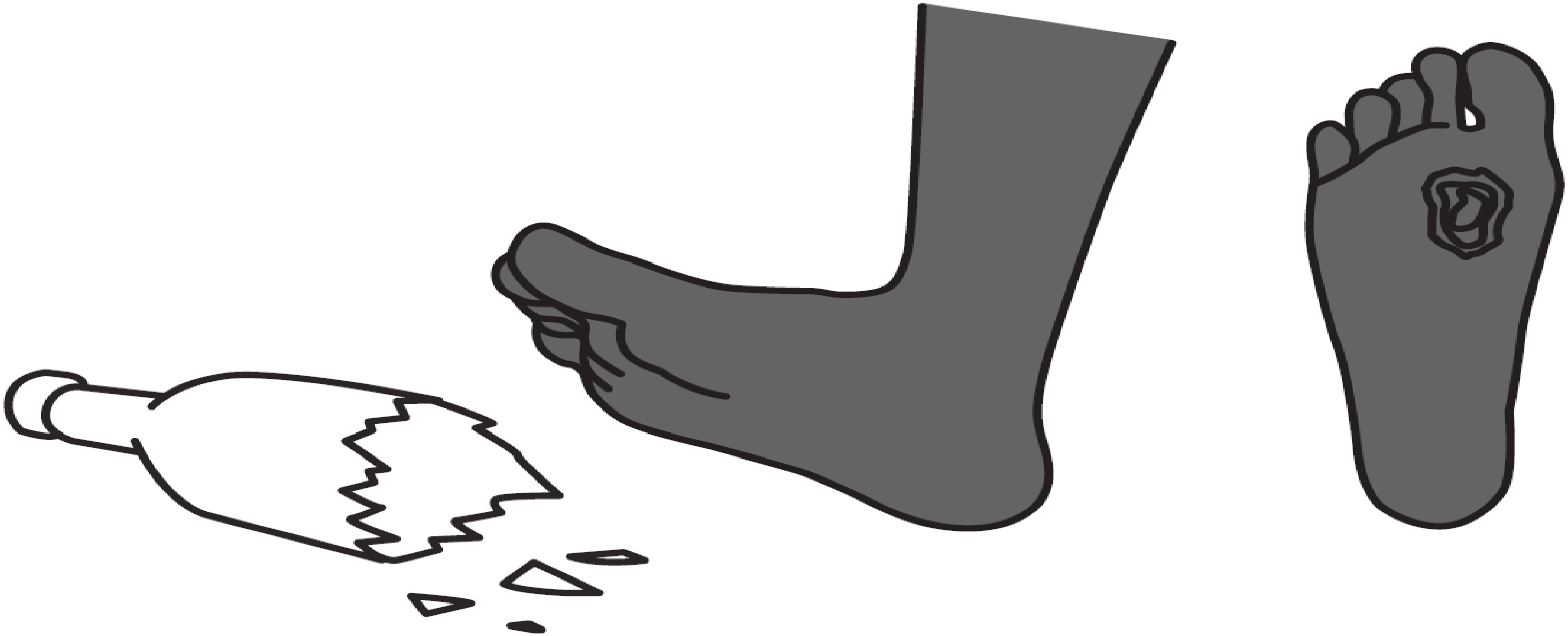

RABE-RABEN CIWO

SABON CIWO

DADADDEN CIWO

SABON CIWO

- ✓Ko dai ya samu ta dalilin rauni/bugewa
- ✓Ko kuma yawancin sa akwai taƙamaiman sababi

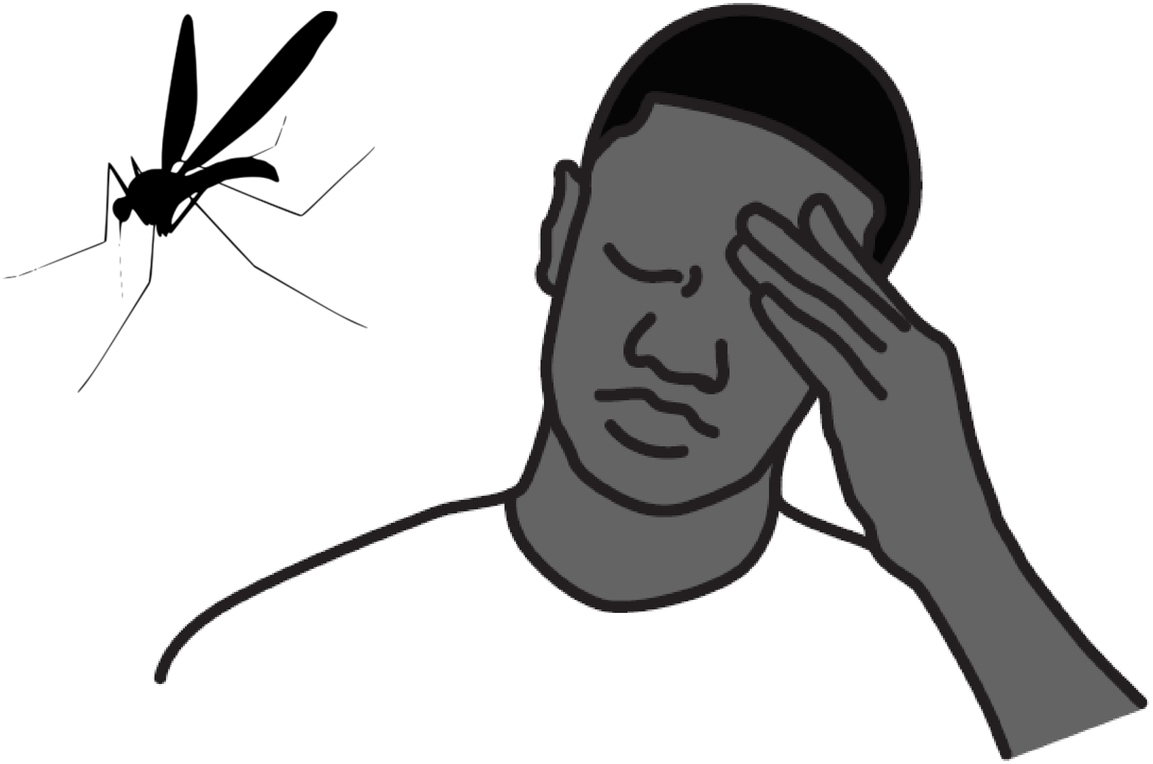

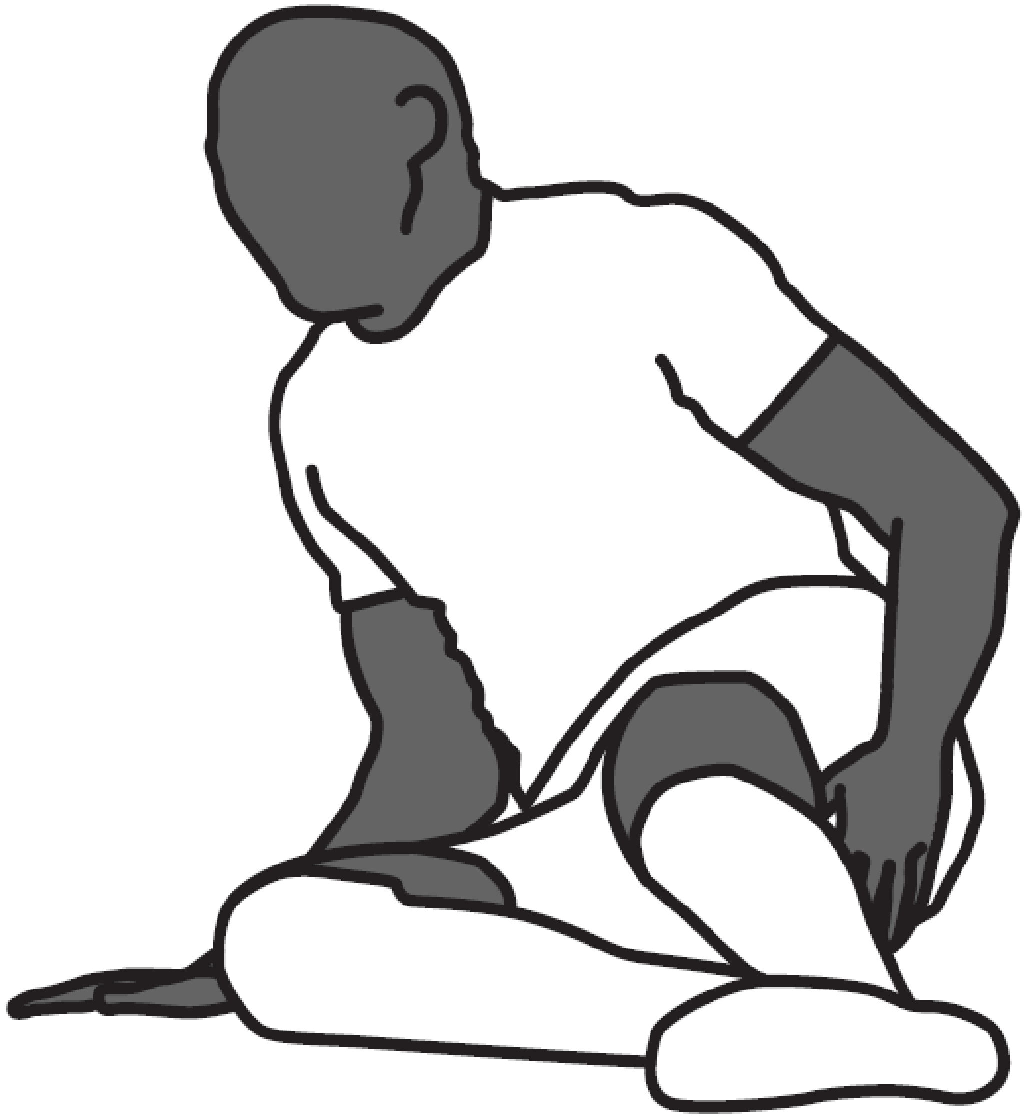

TSARIN JIJIYOYIN JIKI

ƙwayar jijiya

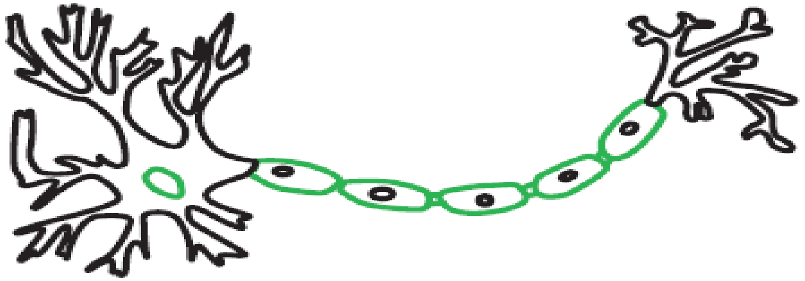

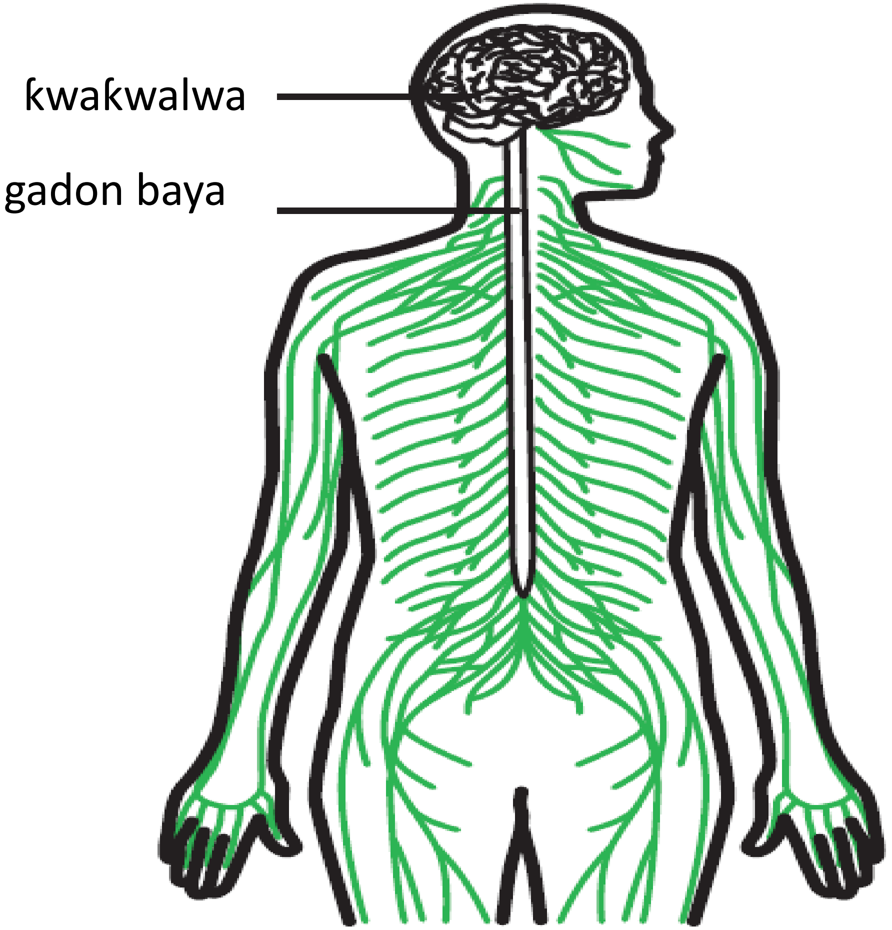

KWAYOYIN KARƁAR SAKO

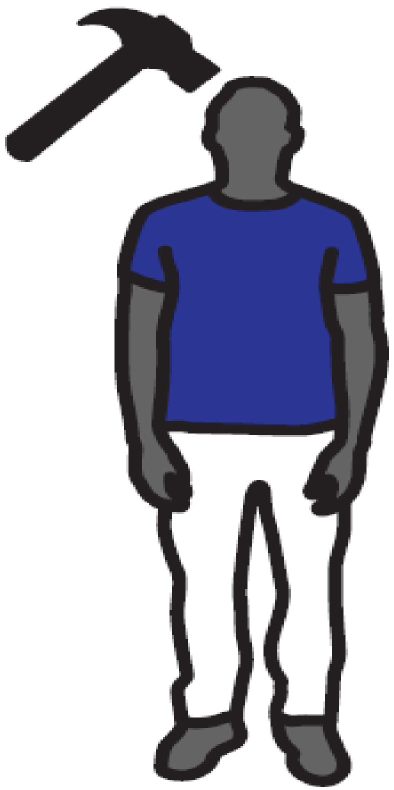

Danna

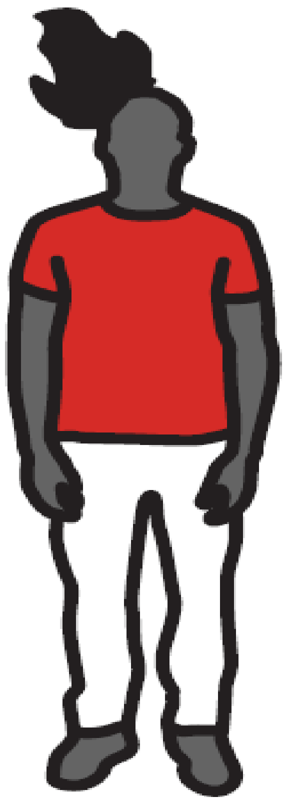

Zafi/Sanyi

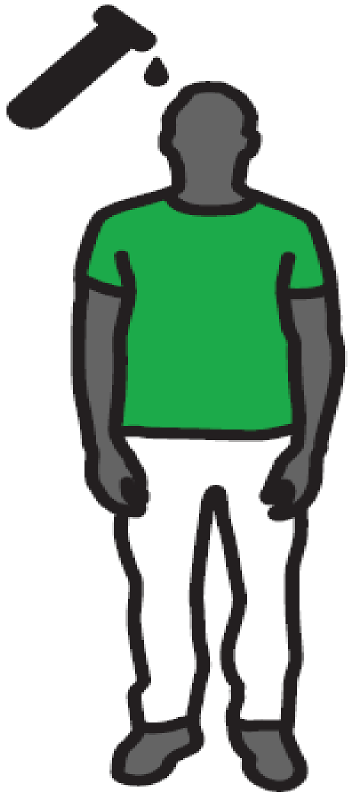

Sinadari/Asid

KWAYOYIN KARƁAR SAKO

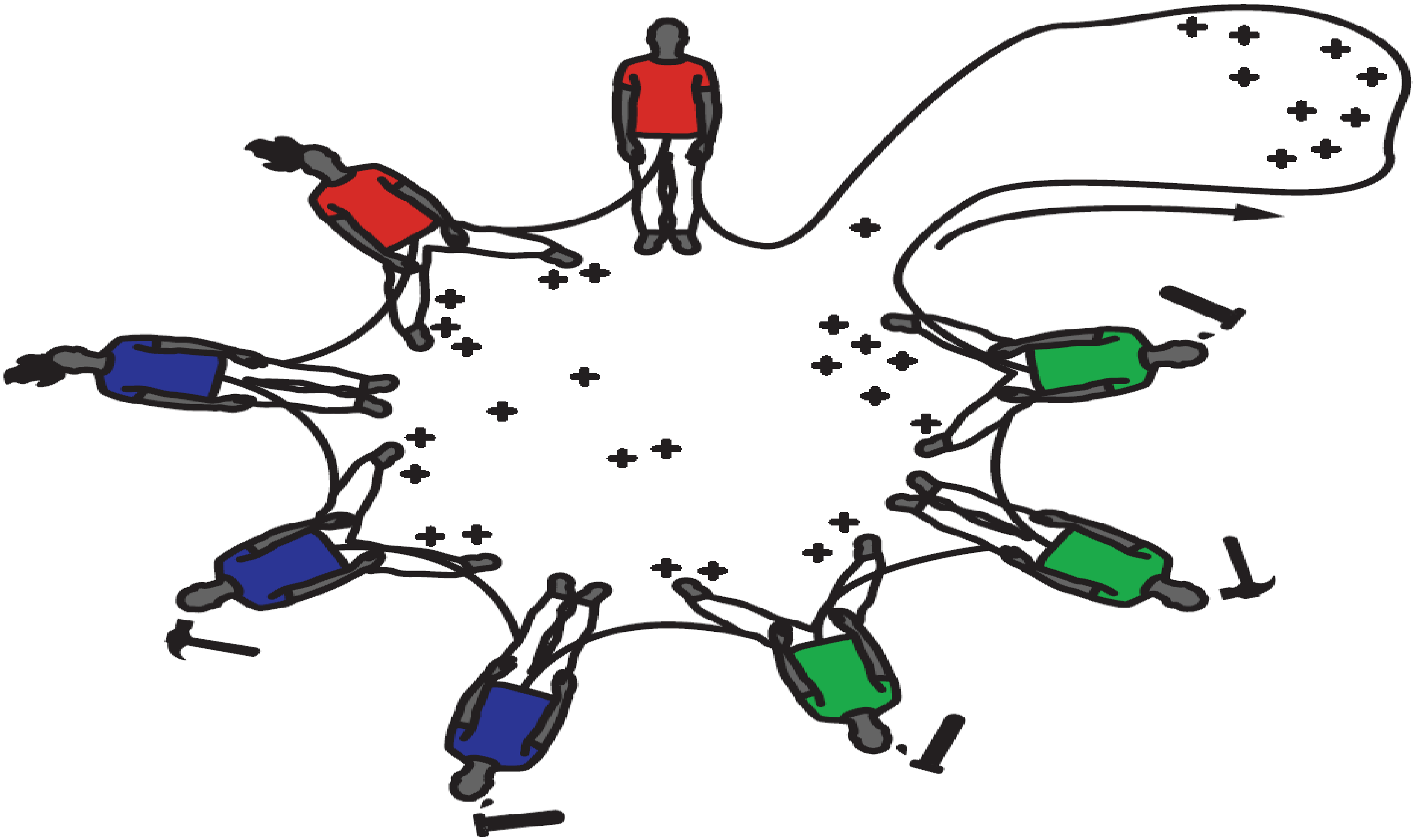

YADDA JIKI KE SARRAFA SABON CIWO

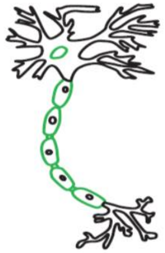

ƙwayar ƙarɓar saƙo

Jijiyar ƙarɓar saƙo

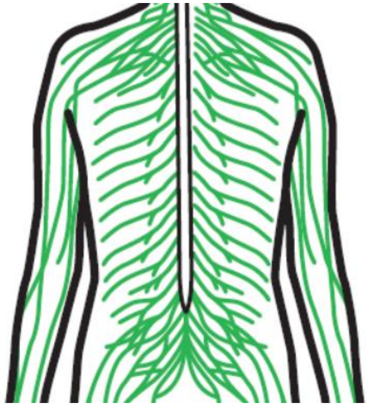

lakar gadon baya

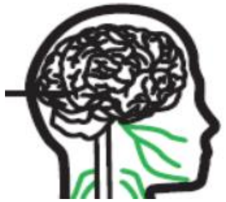

ƙwaƙwalwa

Jijiyar dawo da saƙo

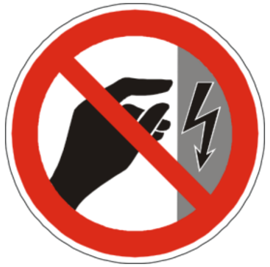

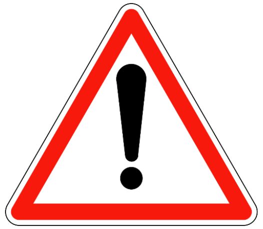

CIWO

Yanayin zabura

Girman rauni/illa ≠ Girman Ciwo ↔ Girman ciwo ≠ Girman rauni/illa

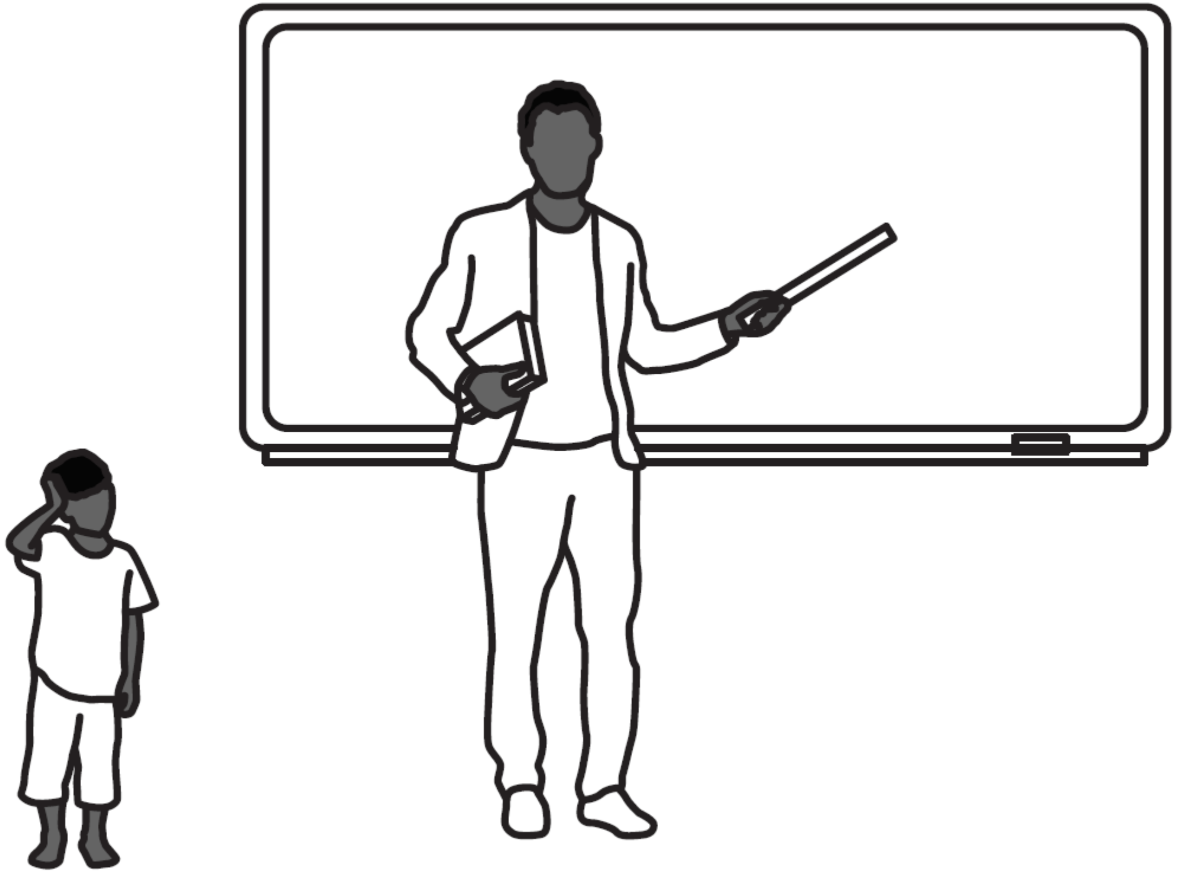

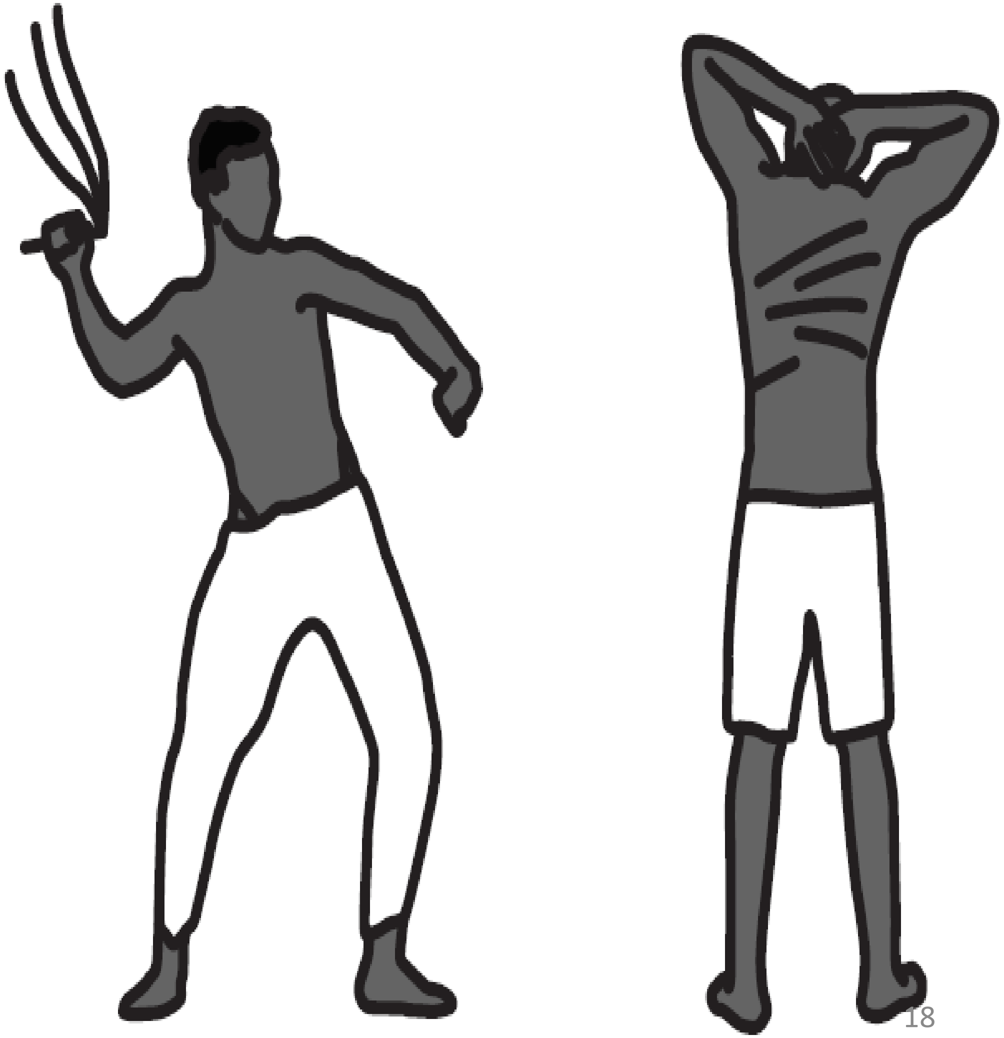

Rauni ≠ Ciwo

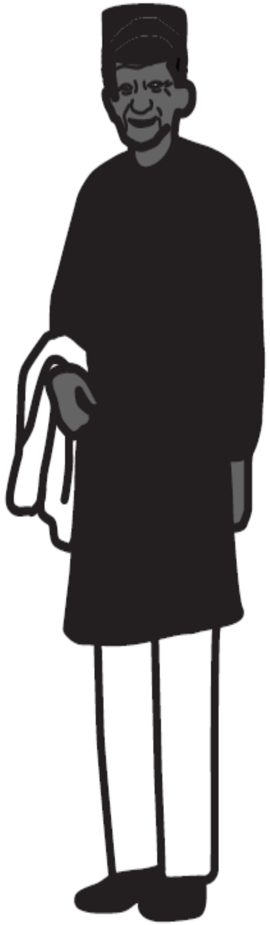

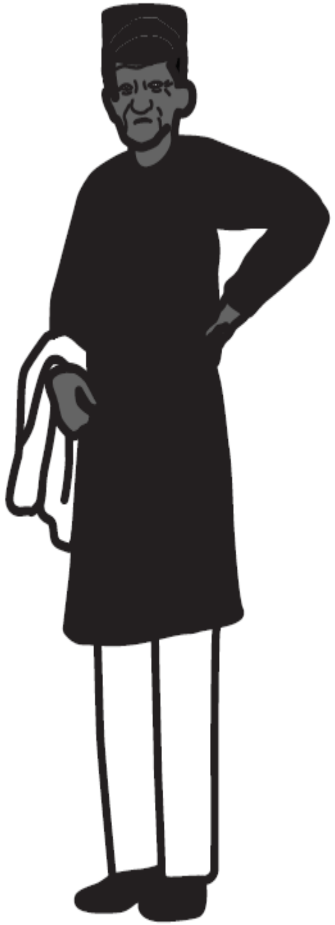

Shekaru

Matsala a cikin hoto	20	50	80	Ciwo
Alamomin tsufa a faifan mahaɗar ƙashin baya	37%	80%	96%	X
Motsewar faifan mahaɗar ƙashin baya	24%	56%	84%	X
Bulluƙowar faifan mahaɗar ƙashin baya	29%	36%	43%	X
Gocewar ƙashin gadon baya	3%	14%	50%	X

AIKIN KWAKWALWA A YANAYIN CIWO

Kwantar da ciwo

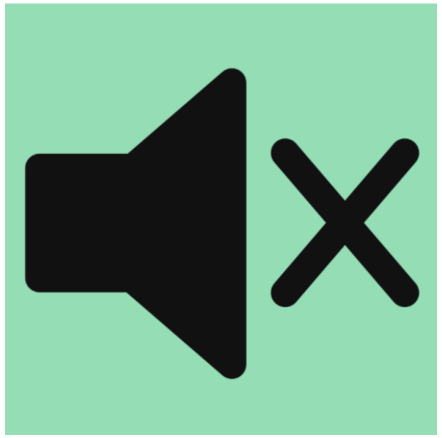

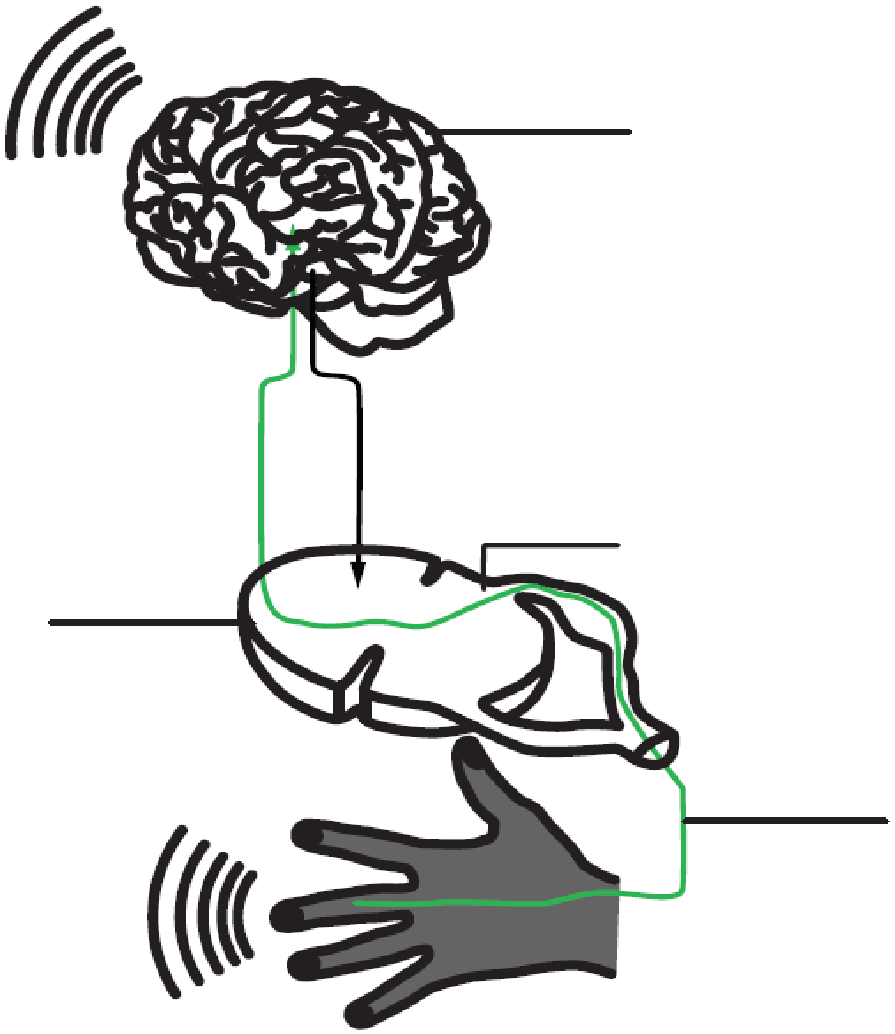

Kambama ciwo

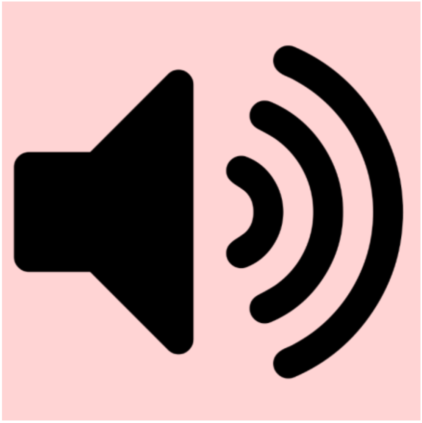

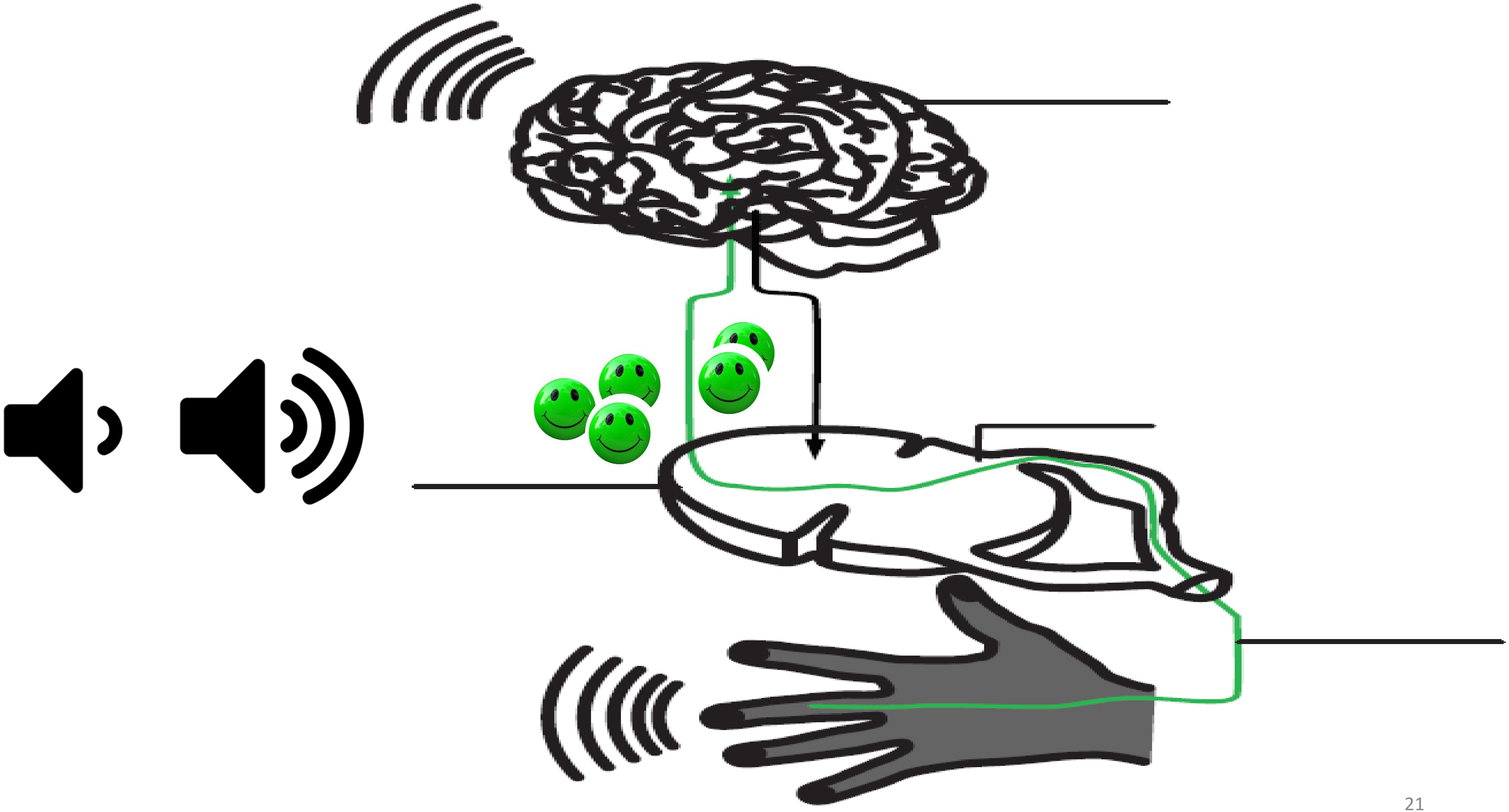

KWAKWALWA: AIKIN KWANTAR DA CIWO

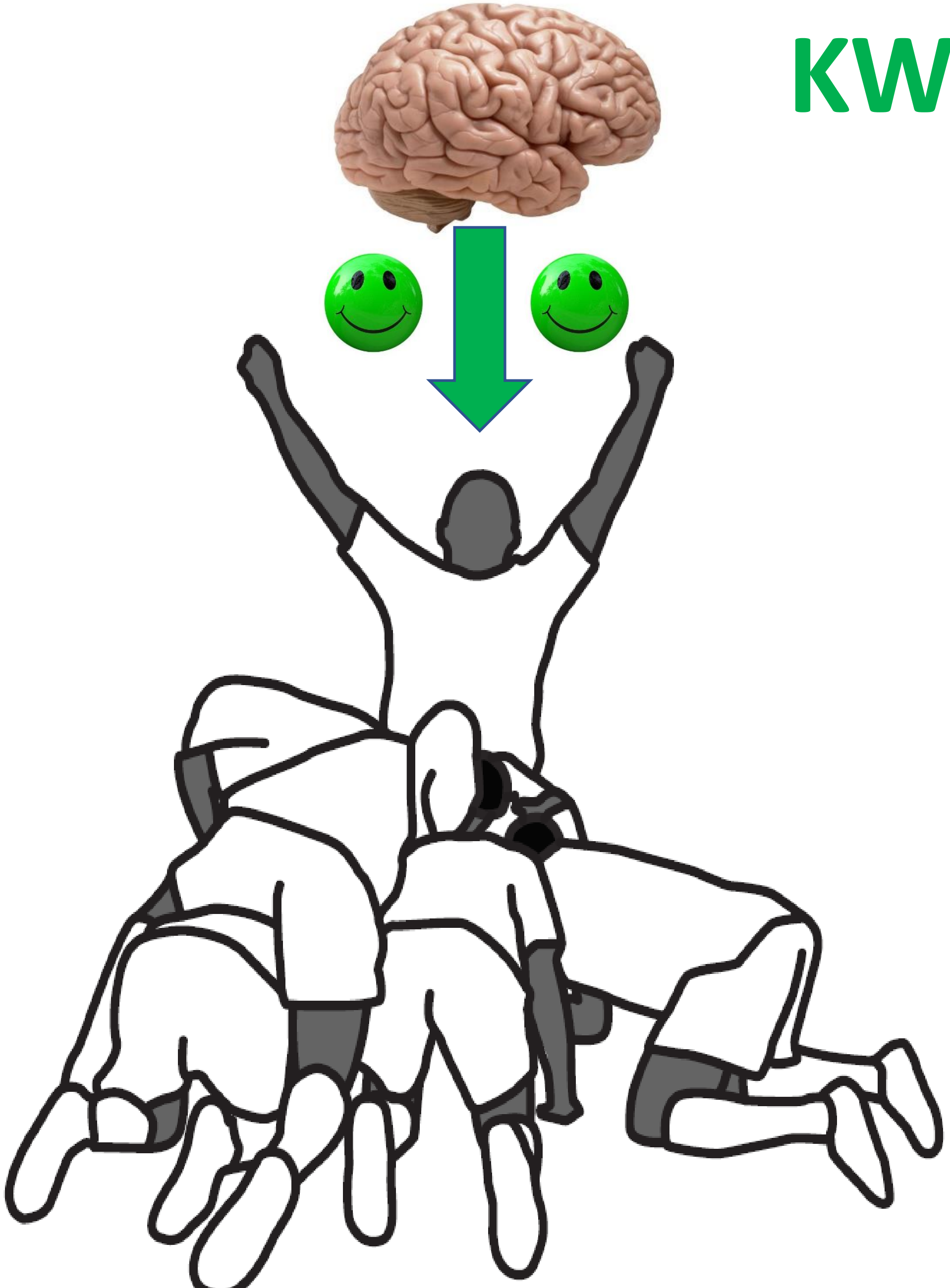

60x

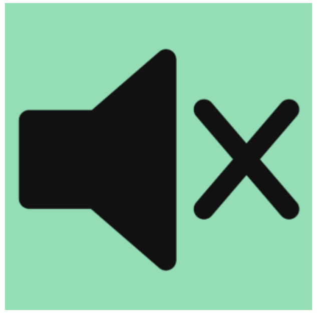

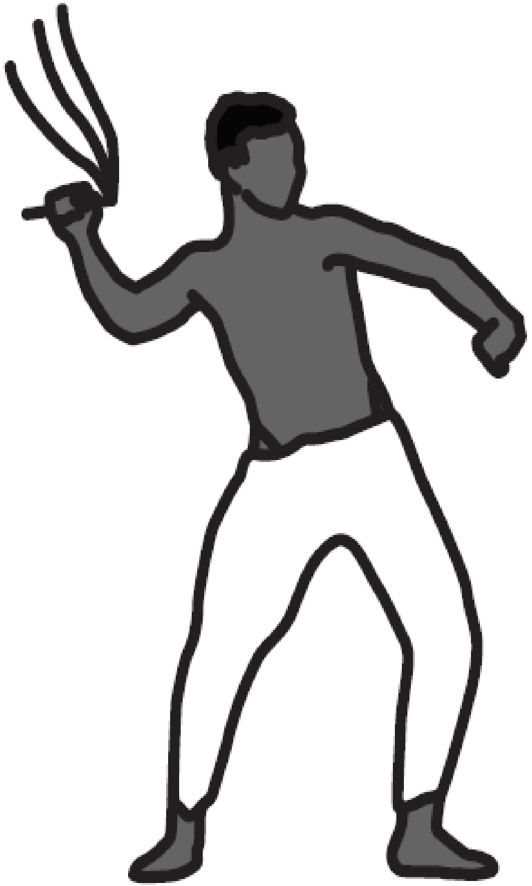

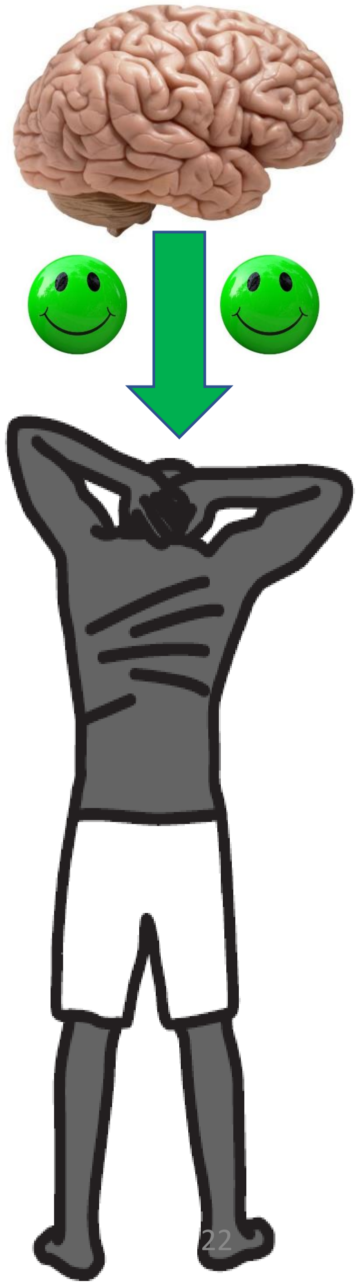

KWAKWALWA: KAMBAMA CIWO

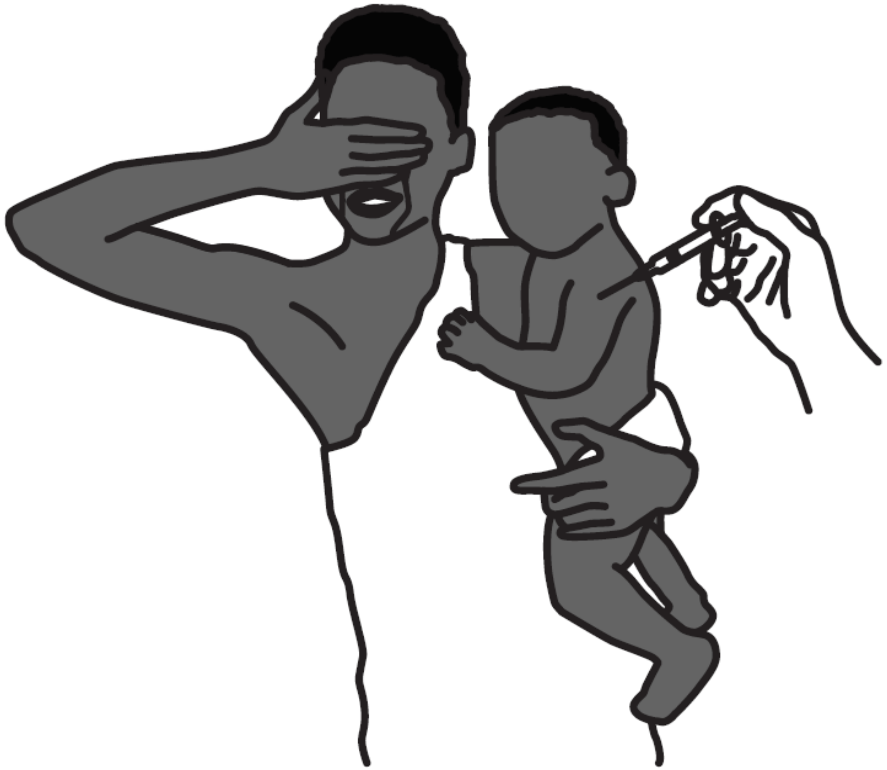

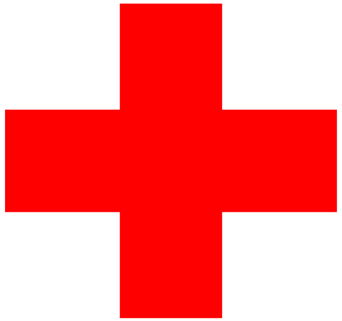

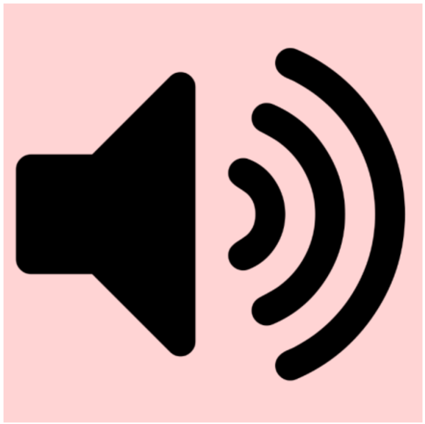

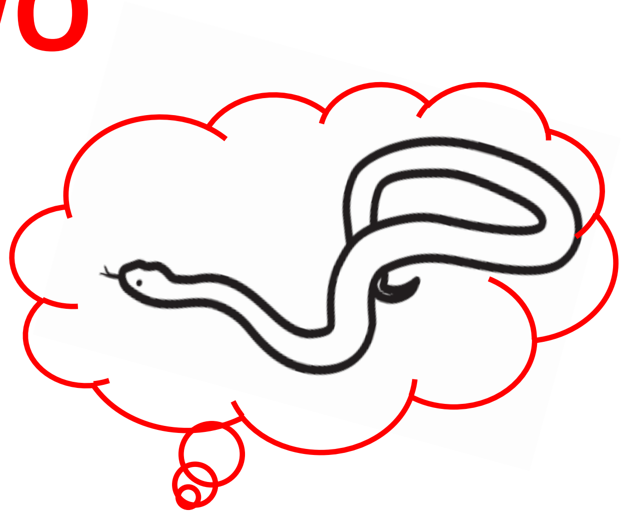

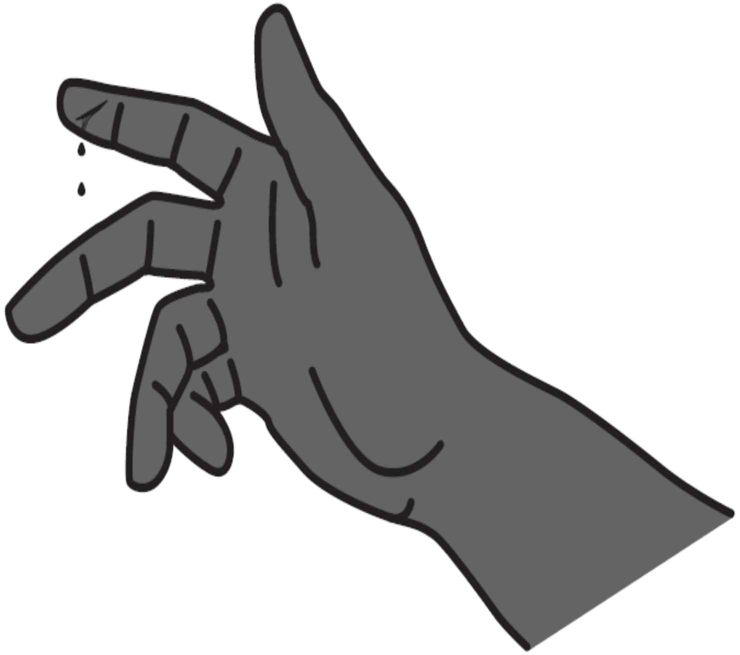

KWAKWALWA: KAMBAMA CIWO

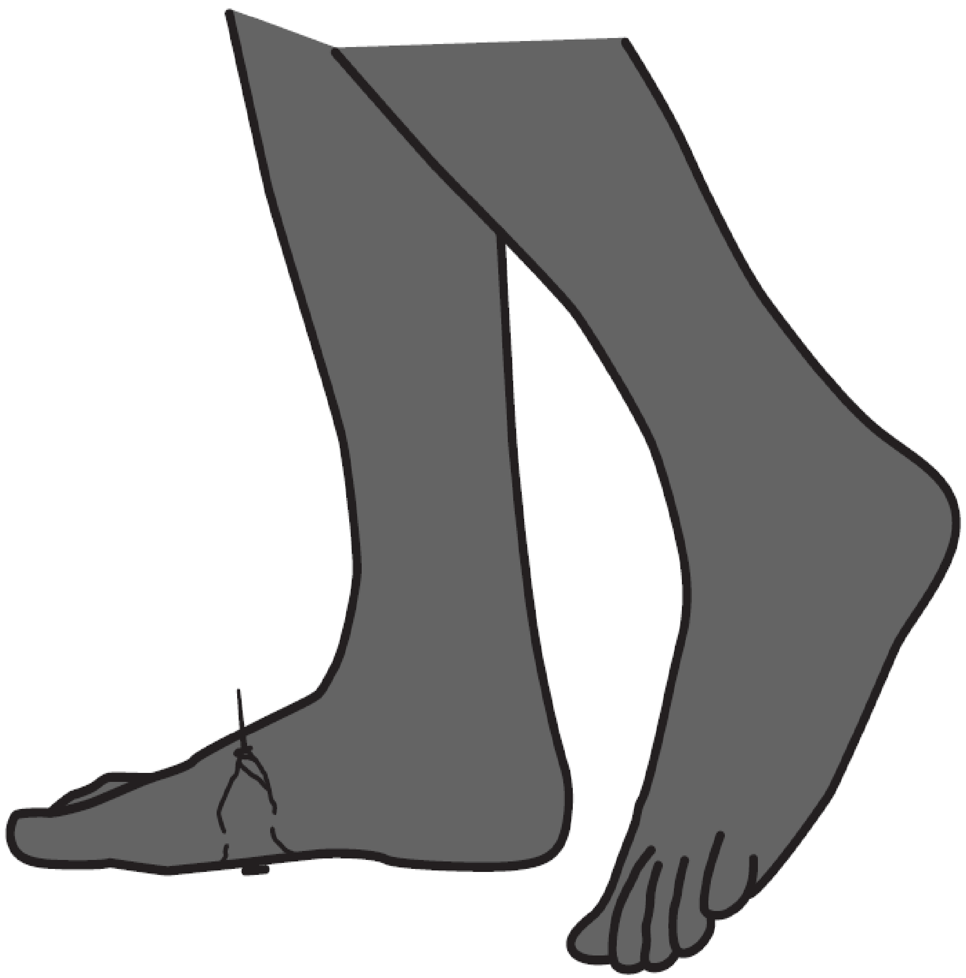

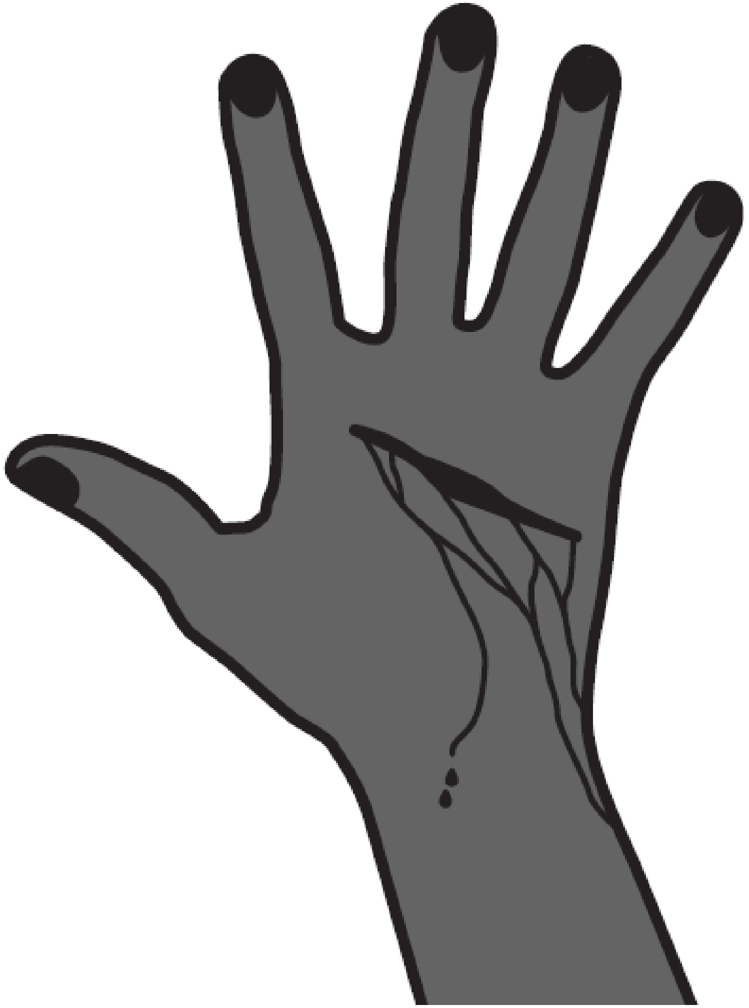

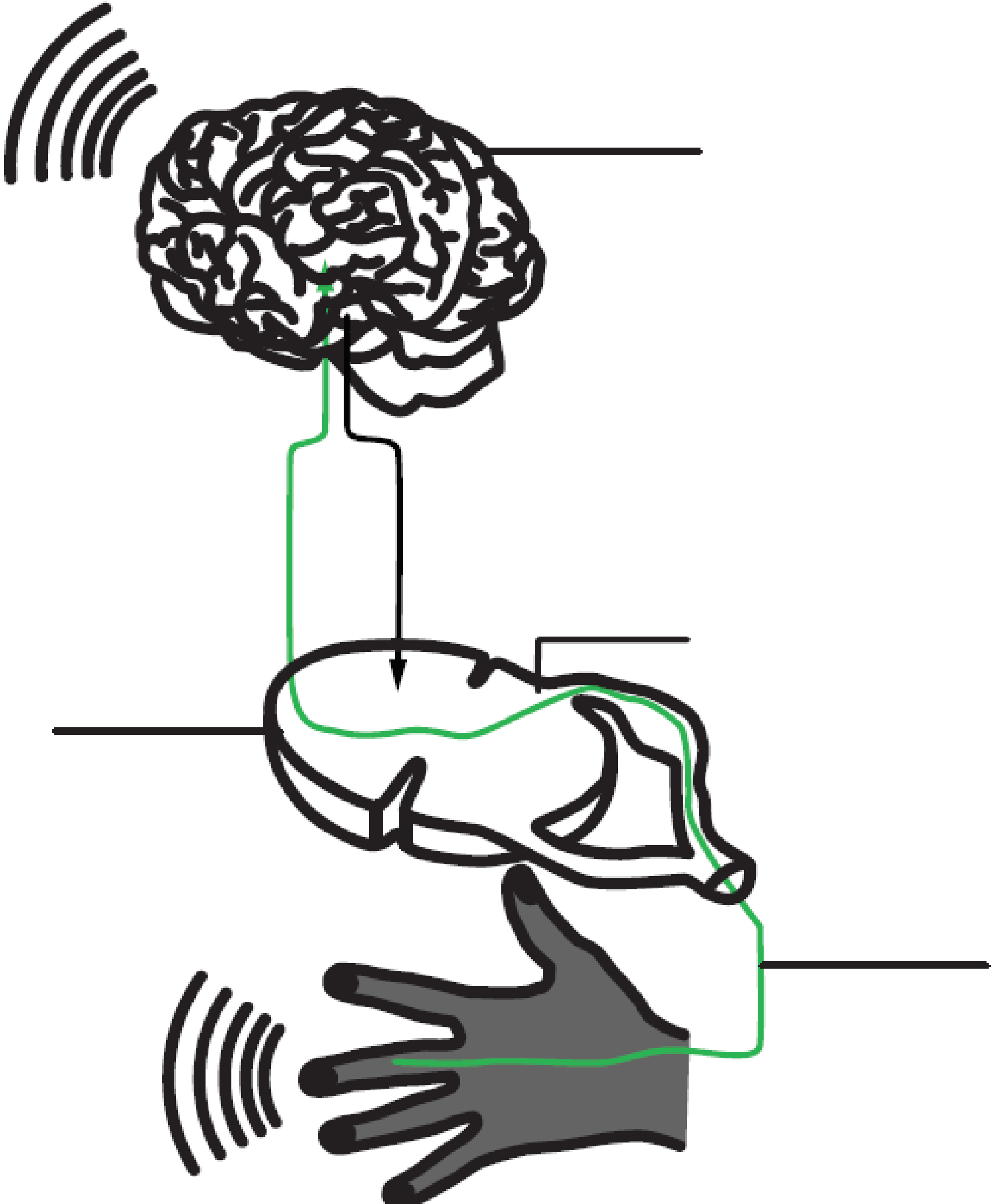

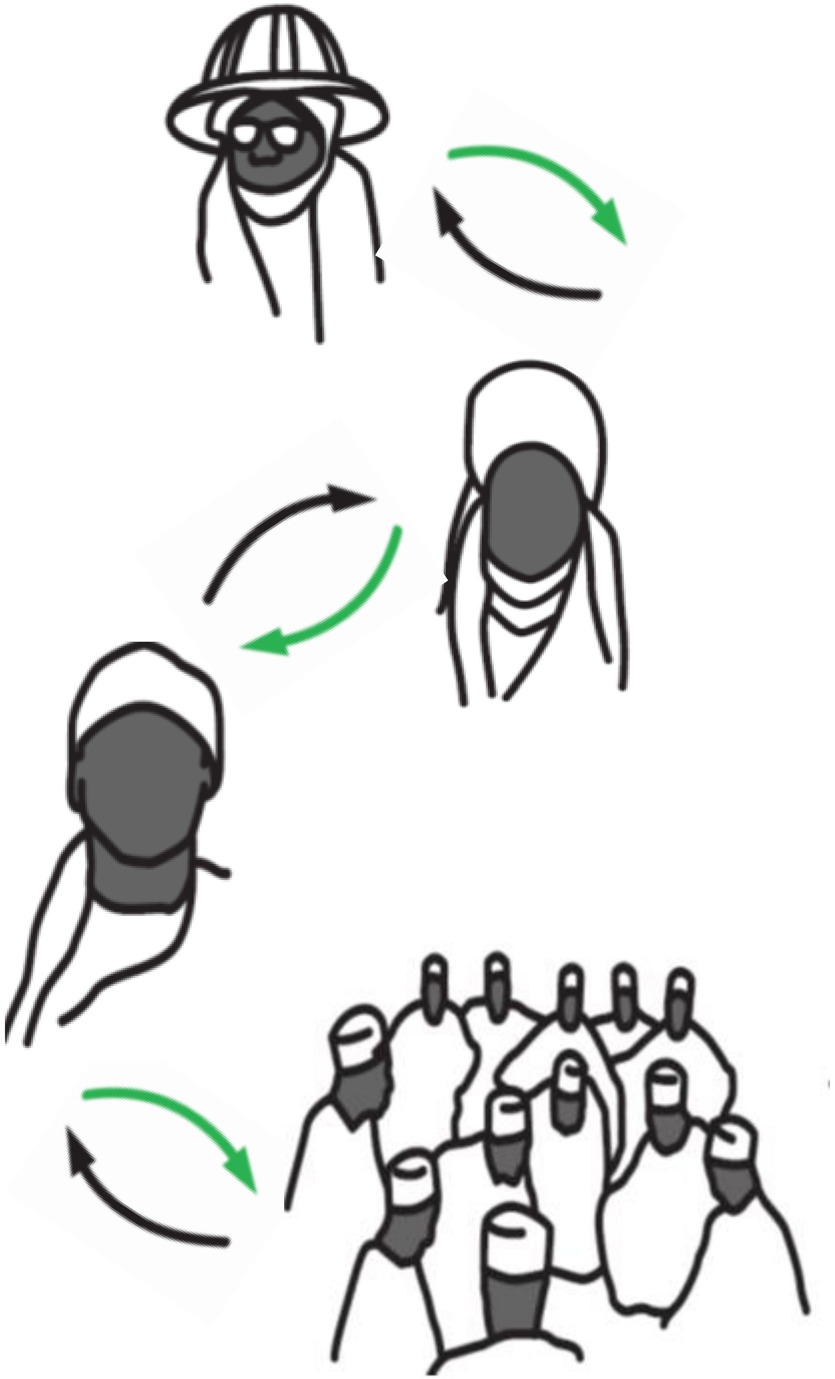

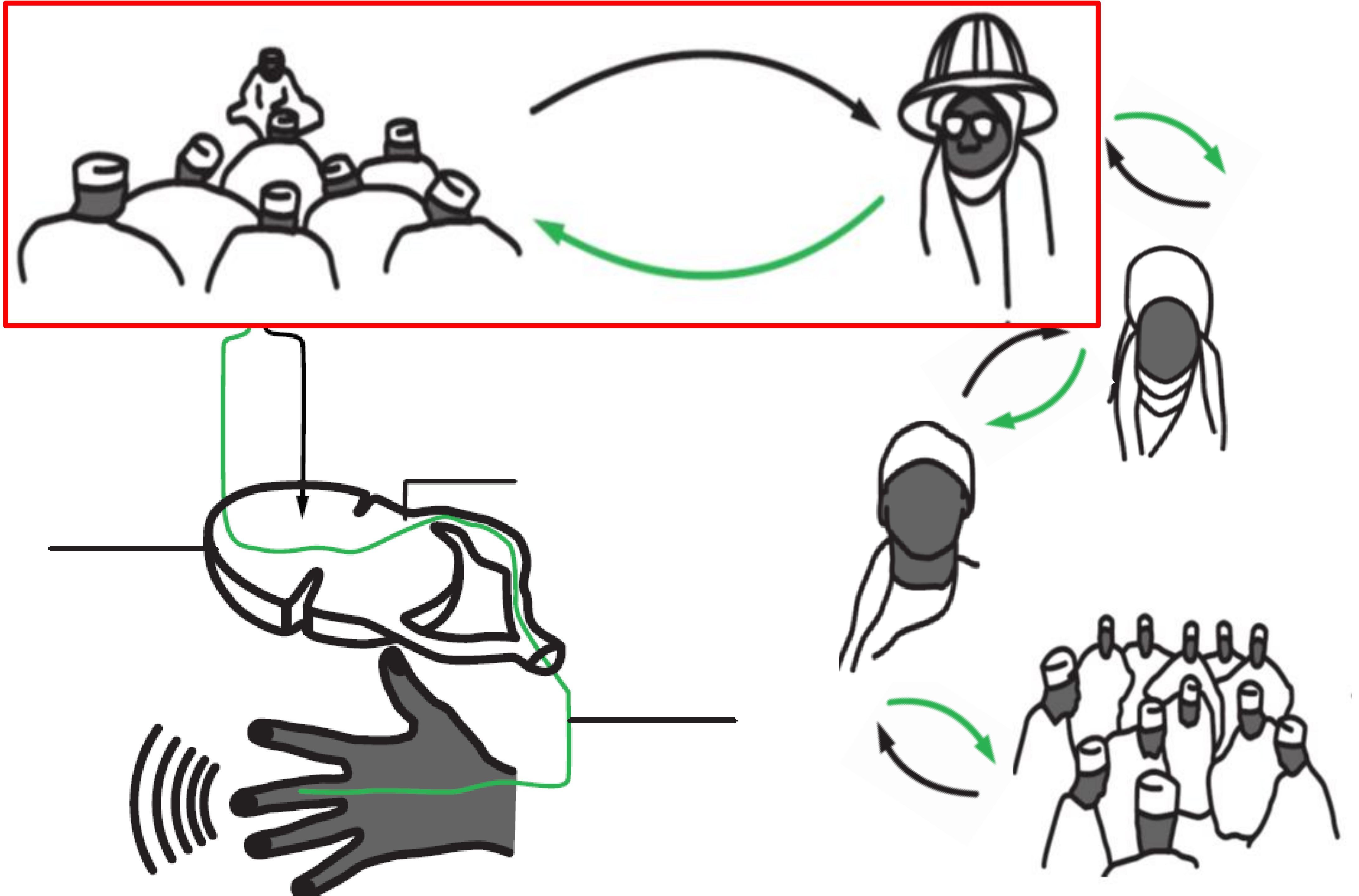

DAƊAƊƊEN CIWO

- ✓ Ciwon fiye da watanni uku
- ✓ Rashin takamaiman sababi
- ✓ A ƙiyasi duk mutum ɗaya a cikin biyar na da irin wannan ciwo

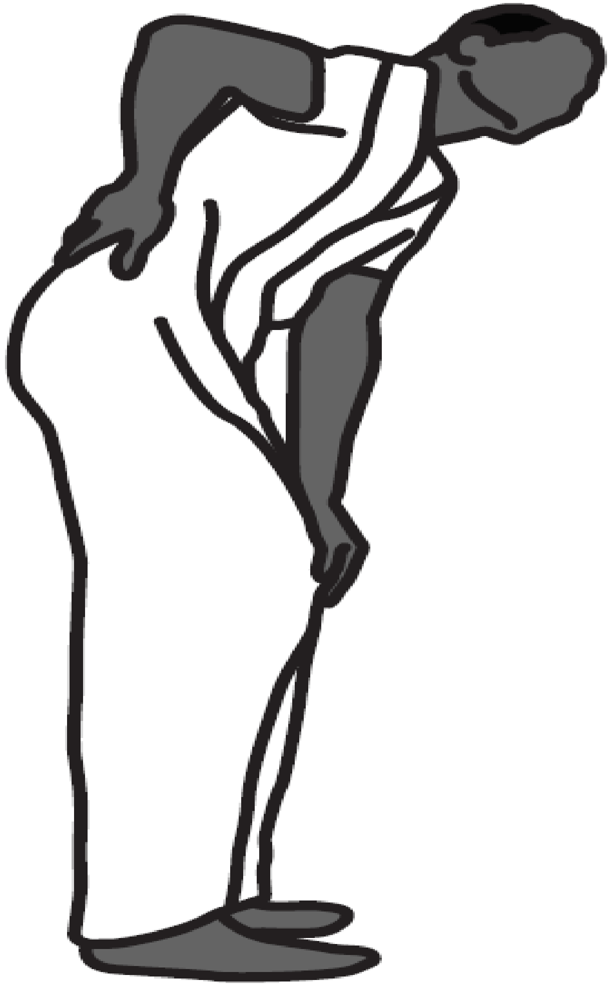

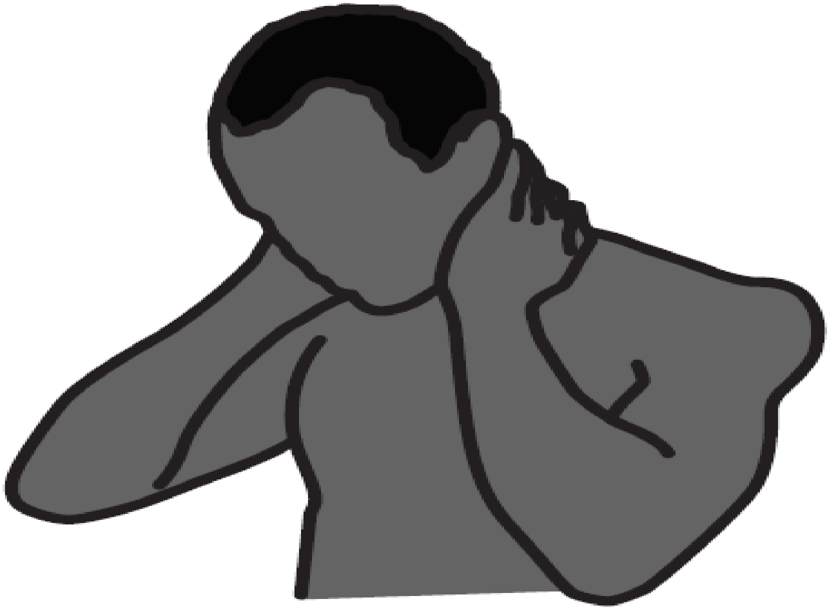

DAƊAƊƊEN CIWO

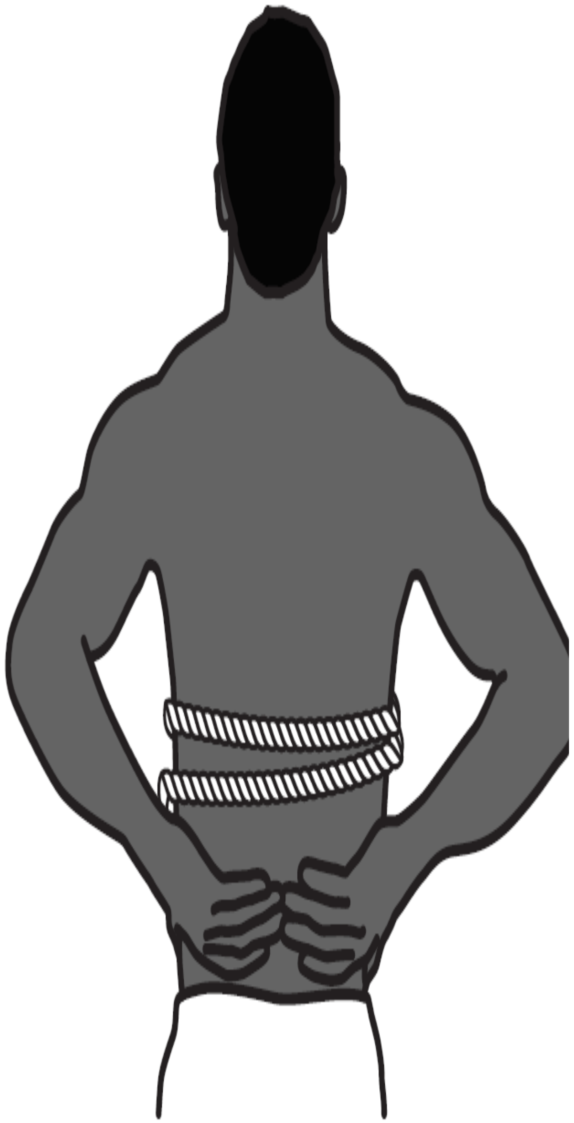

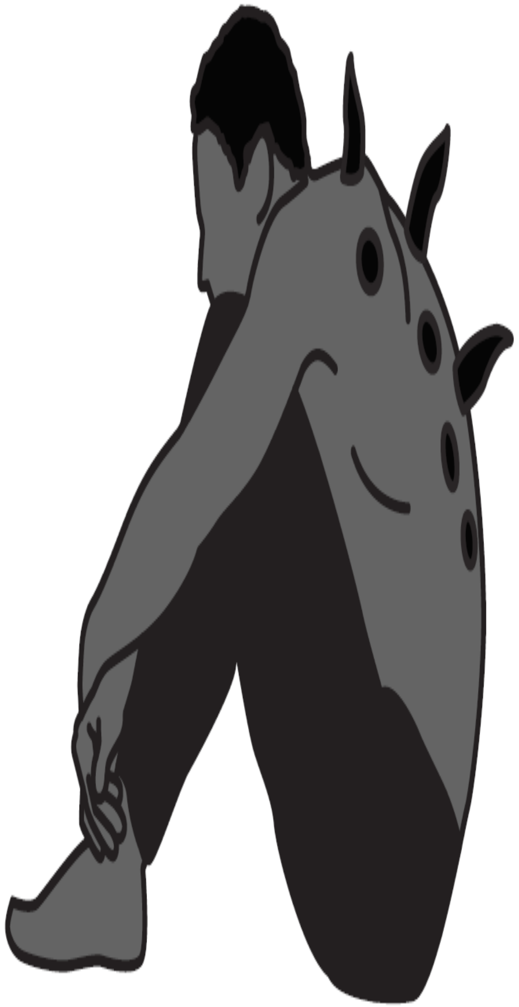

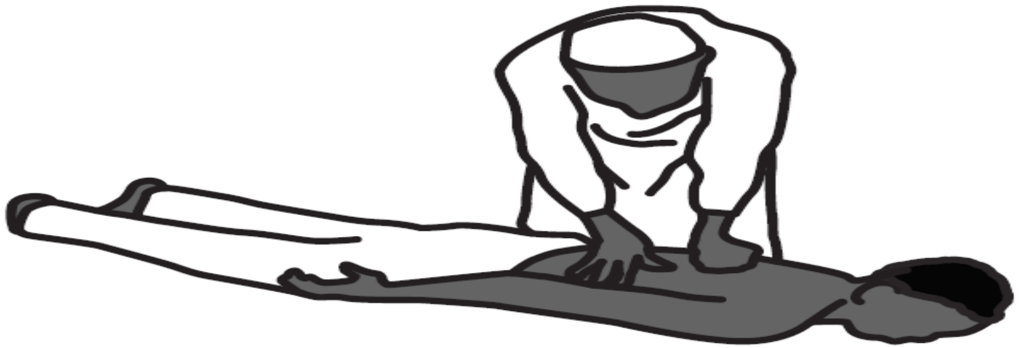

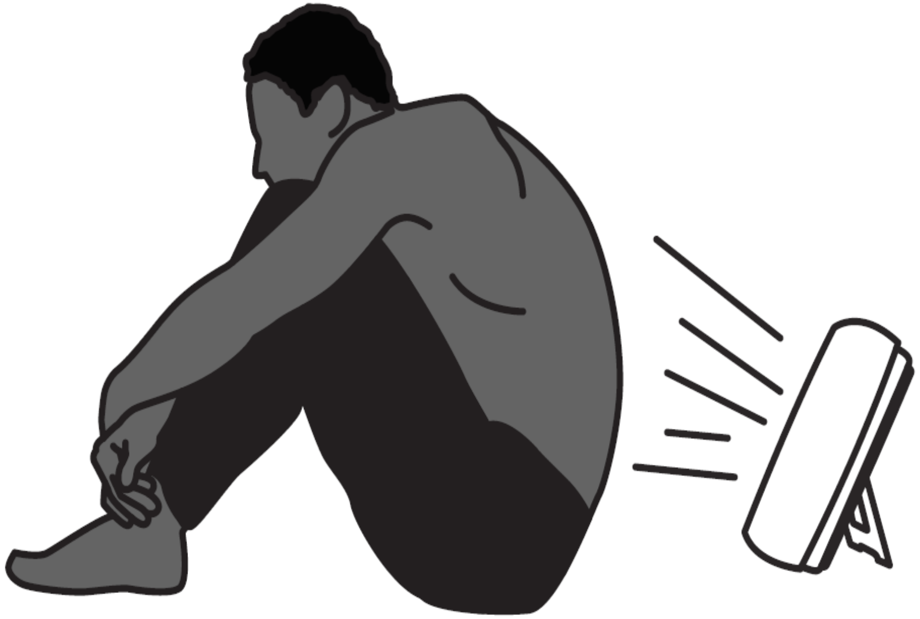

MASU TAKA MUHIMMIYAR RAWA A WAJEN CIWON MU?

Motsa jiki

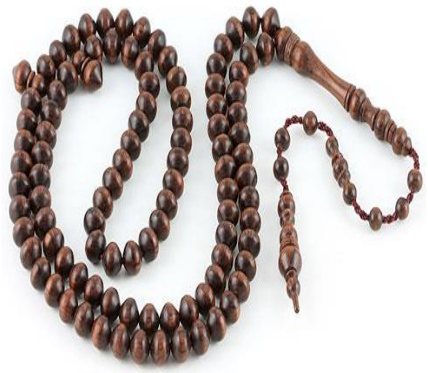

Rauni

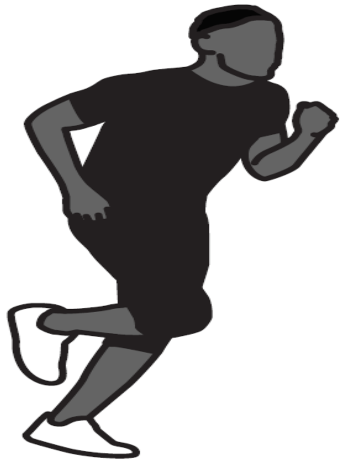

Addini da al’ada

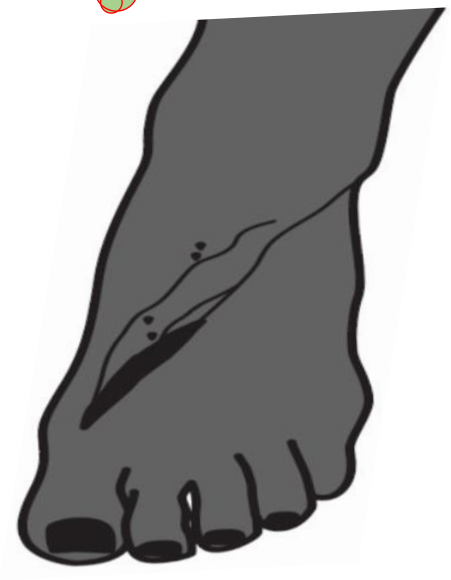

ME YASA CIWON ƊANLADI YAKI CI YAKI CINYEWA?

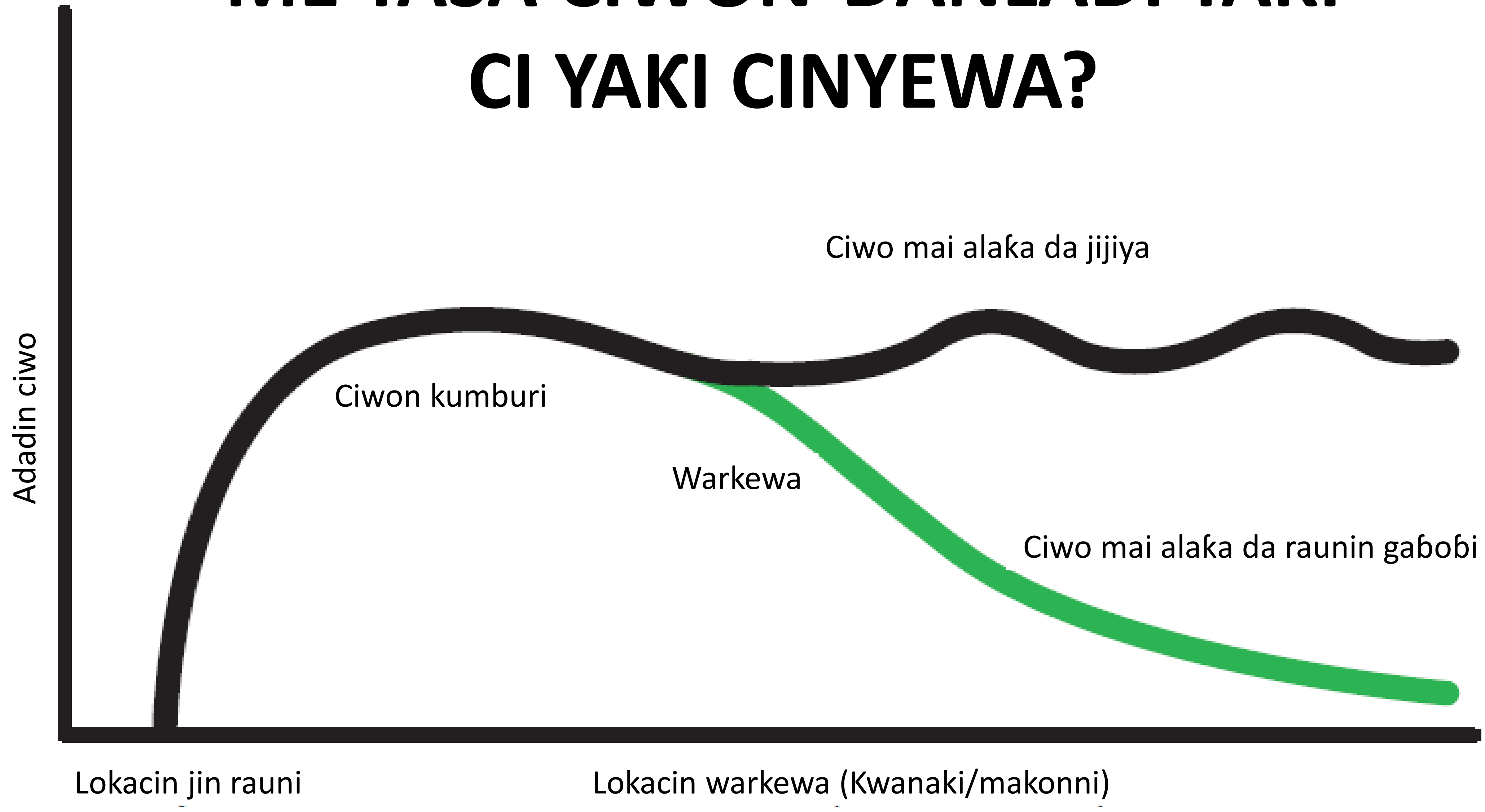

YADDA ƊAƊAƊƊEN CIWO KE ZAMA JIKI A JIKIN MU

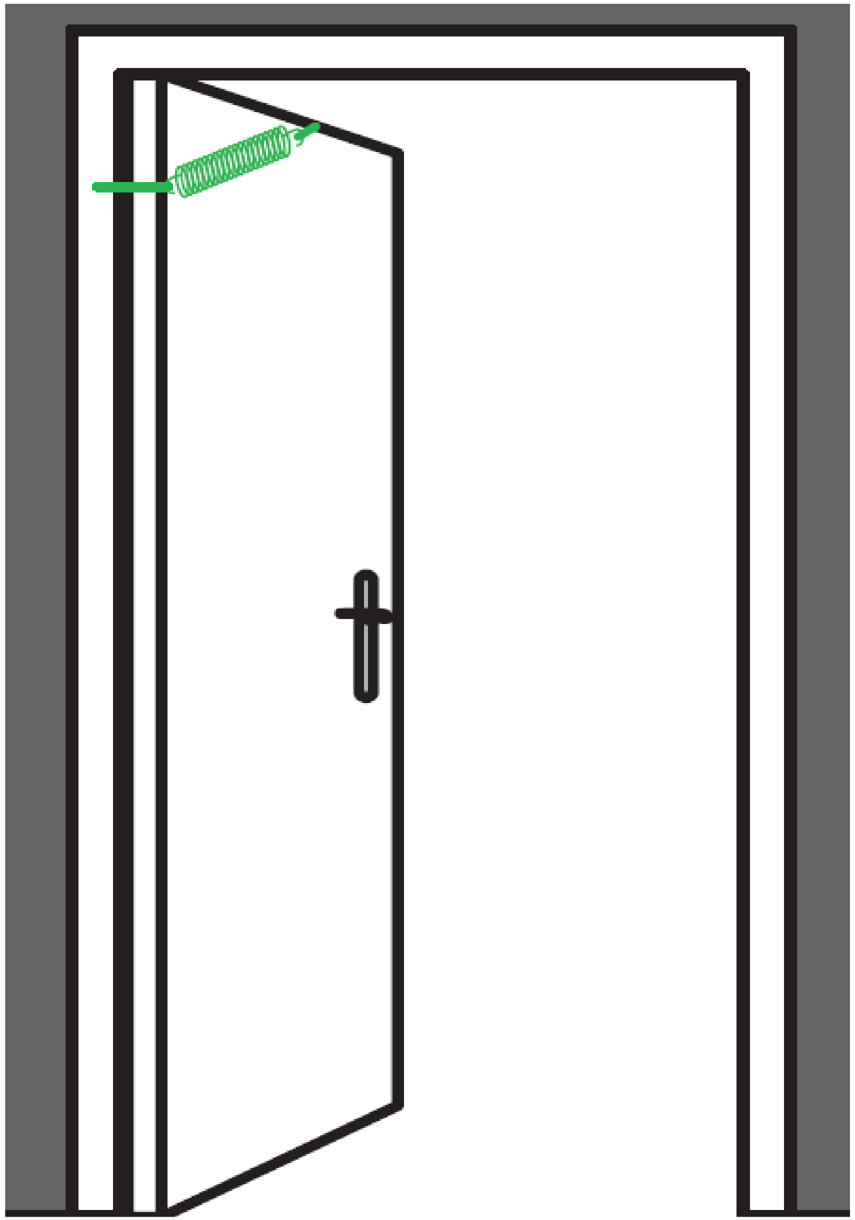

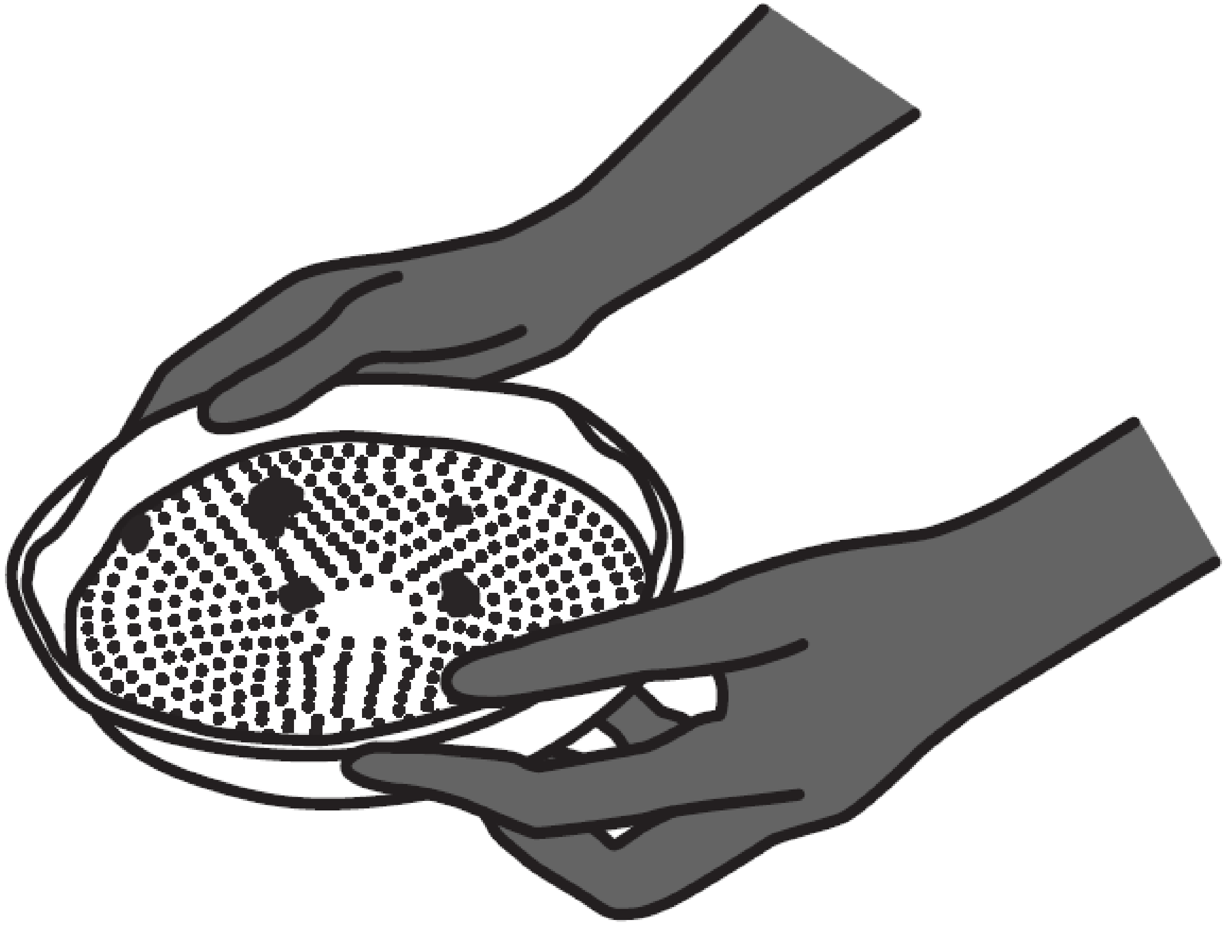

YADDA CIWO YAKE AURAR JIKIN MU A LOKACIN ƊAƊAƊƊEN CIWO

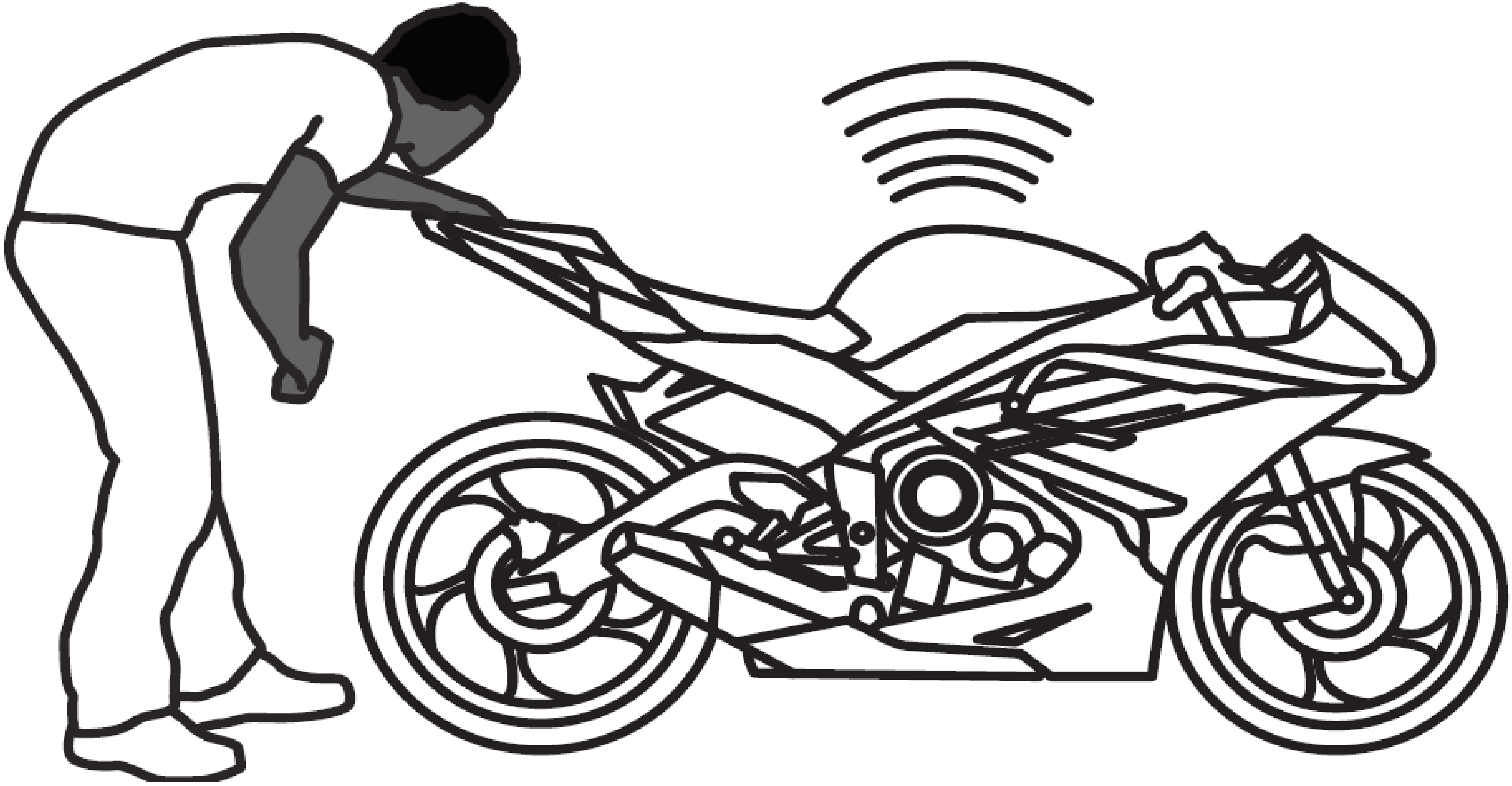

ME ZAMU IYA CEWA YA HADDASA WA ƊANLADI DAƊAƊƊEN CIWO HAKA?

1. Adadi ko girman raunin da suka ji?
2. Kwayoyin halittar jikin su?
3. Juriyar jiki?

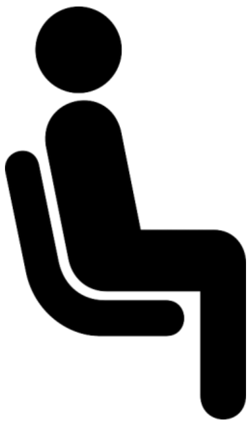

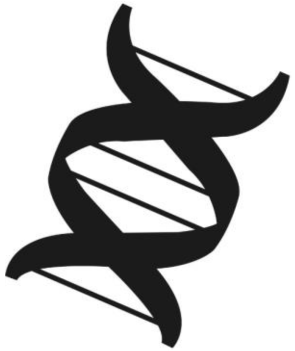

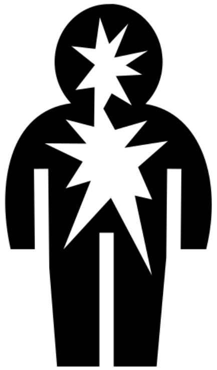

wannan shine muke da tasiri a kan sa

TA YAYA ZAN MAYAR DA JIKI NA MAI JURIYA DA JARUMTA WAJEN DANNE CIWO?

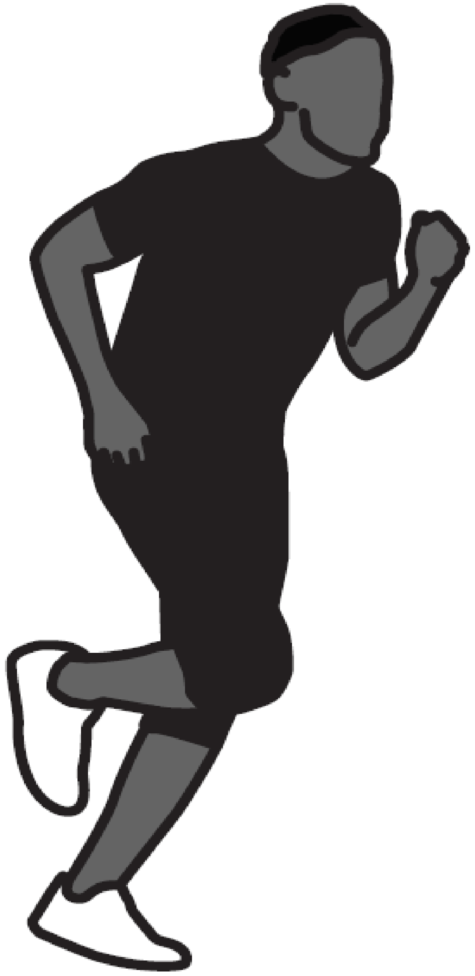

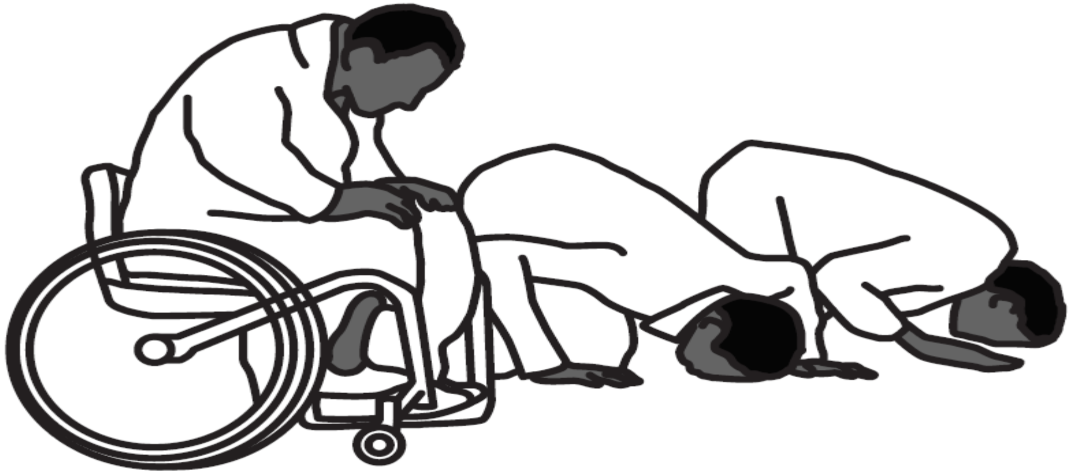

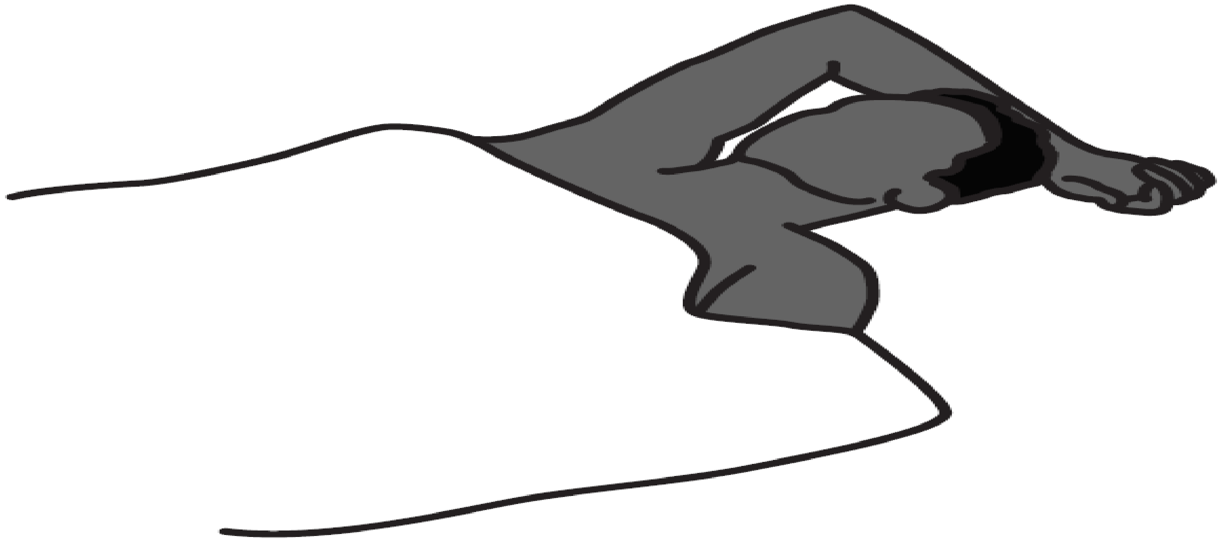

TA YAYA ZAN WA KAI NA KYAKYKYAWAN CANJI?

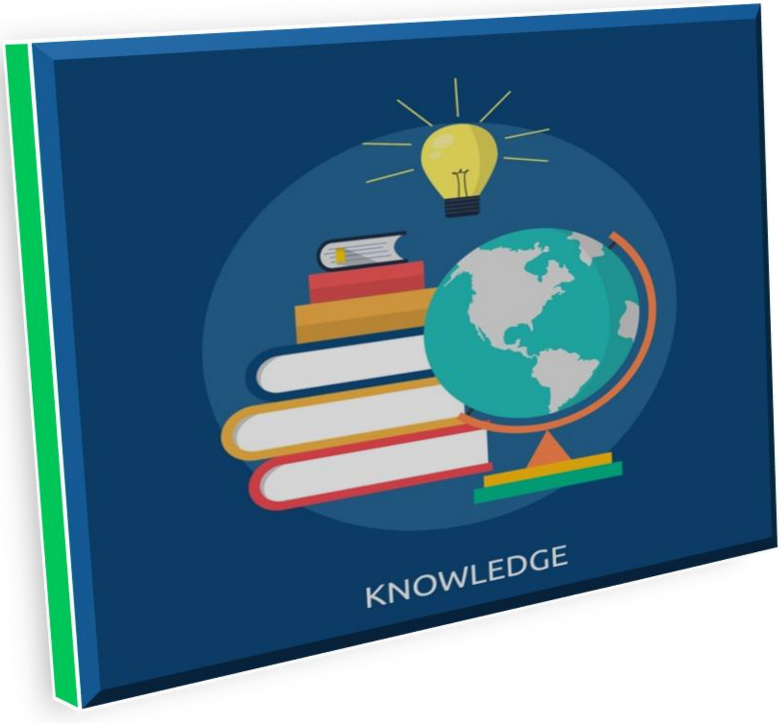

Nemi Ilimi!

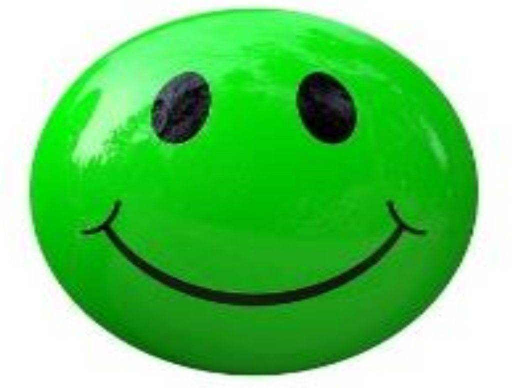

Rage damuwa!

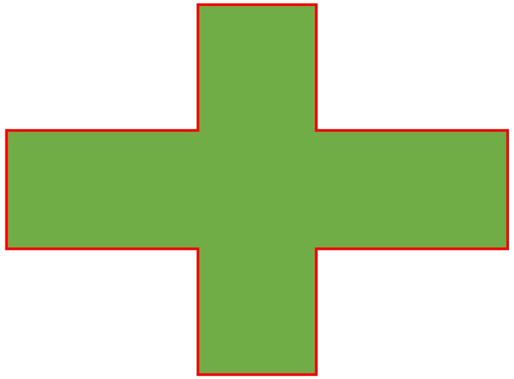

Zamo cikin kyakykyawan yanayi da zato!

DA KAƊAN KAƊAN JIKI ZAI SABA IN BA HAKA BA JIKI ZAI YI TSAMI!

Mintunan motsa jiki

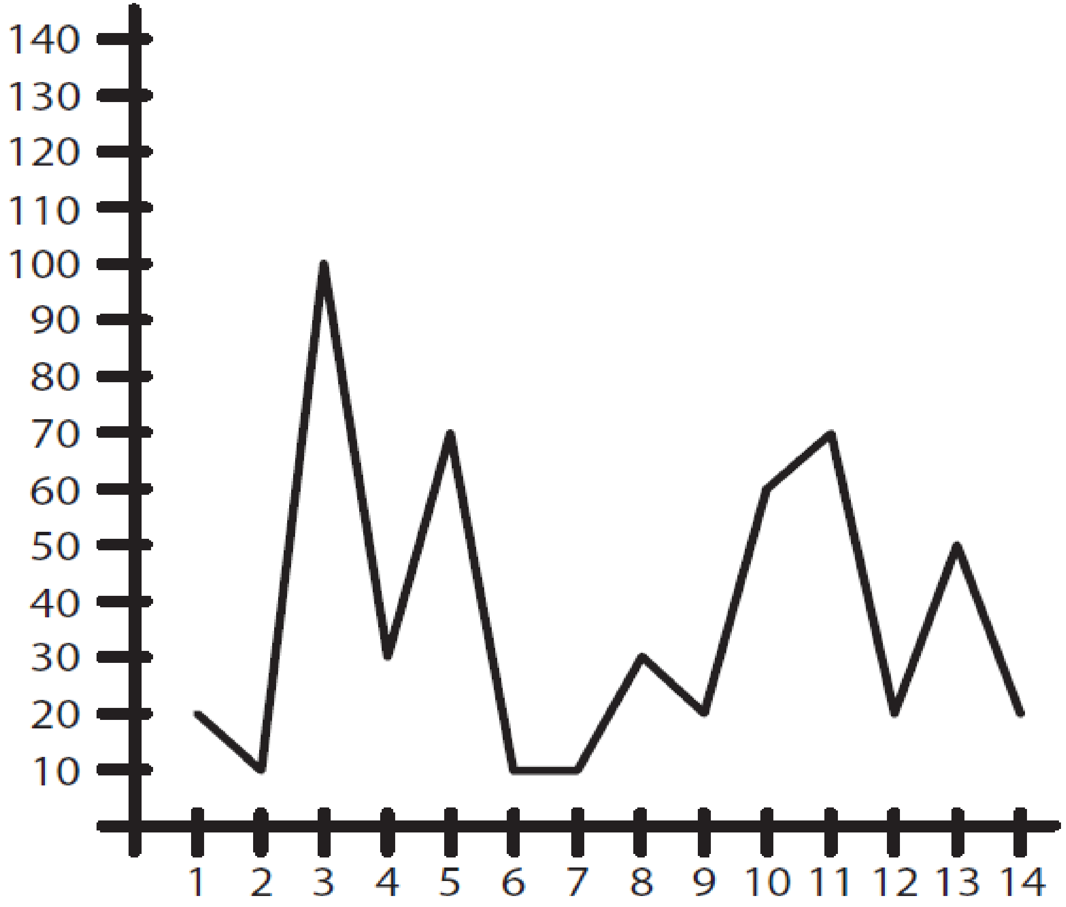

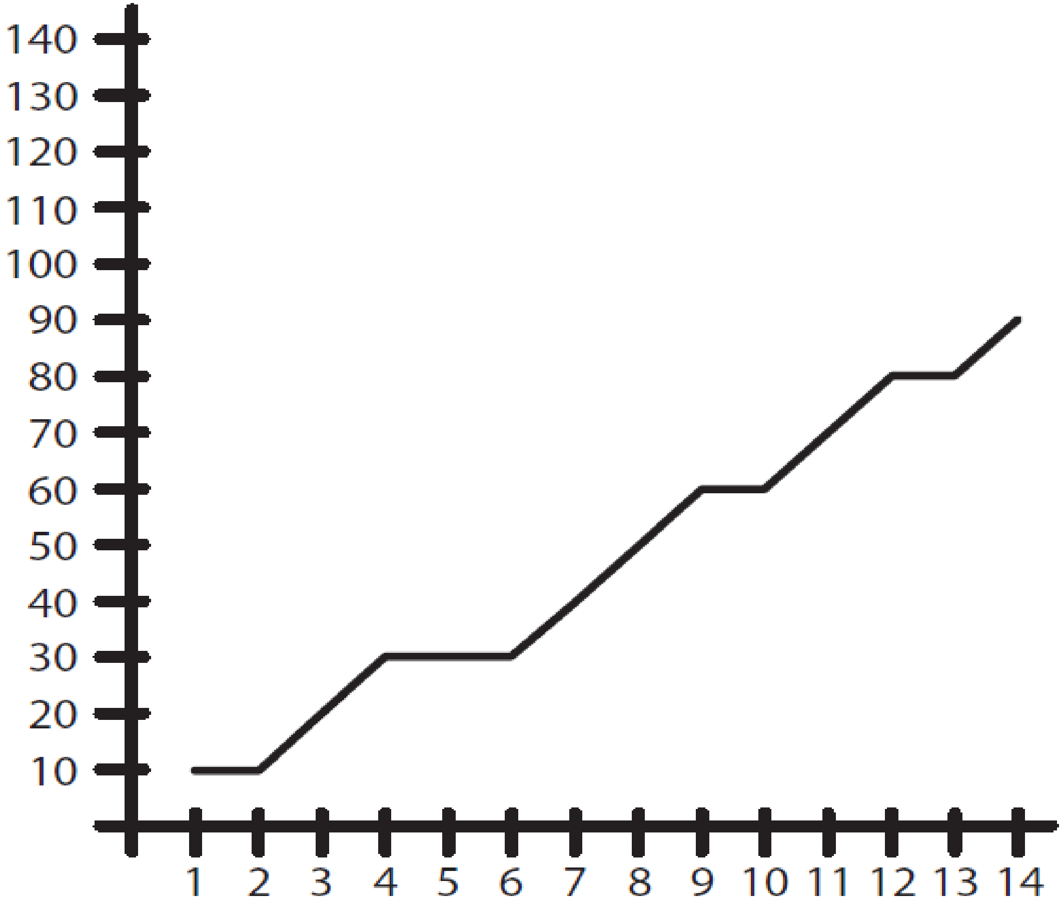

Kwanaki

TSARABA DOMIN SAURARO A GIDA

- Ka saurari saƙon a nutse
- Idan akwai abun da ya shige maka duhu kada ka manta shi
- Idan mun haɗu a zama na biyu sai ka tambaya

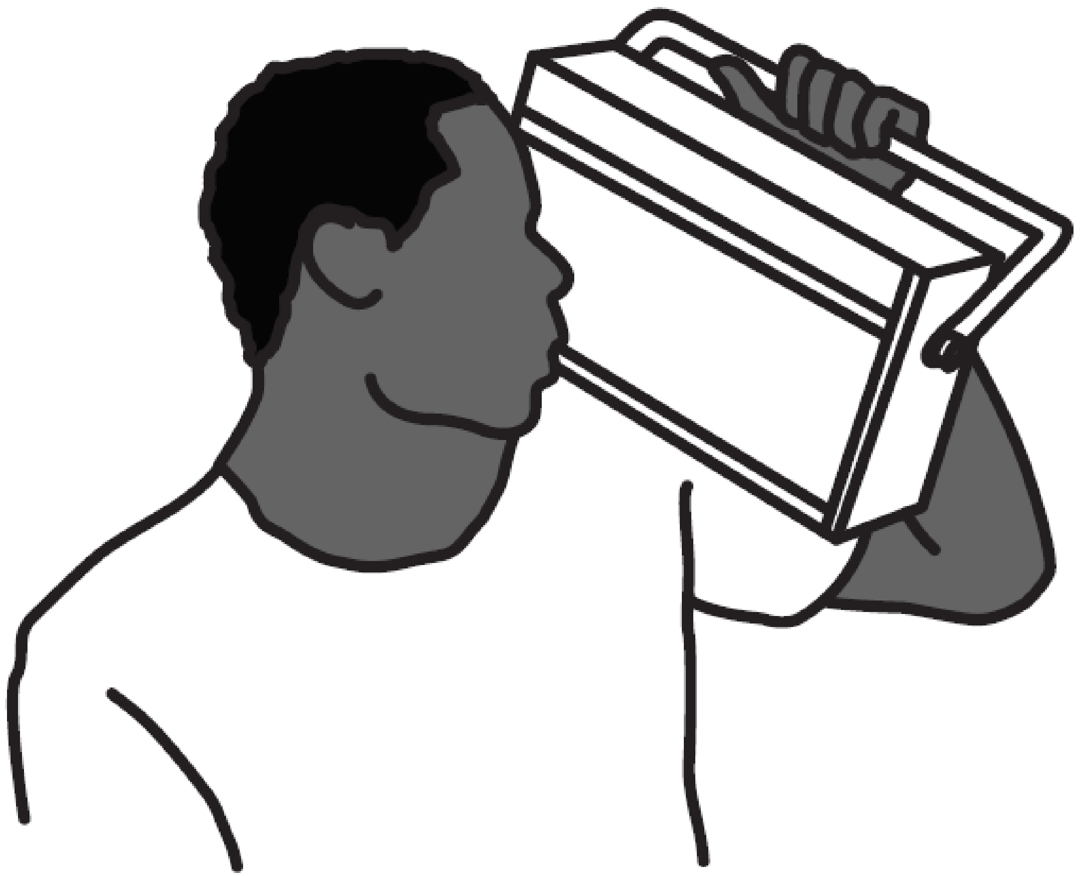

MUN GODE DA SAMUN HAƊIN KAN KU

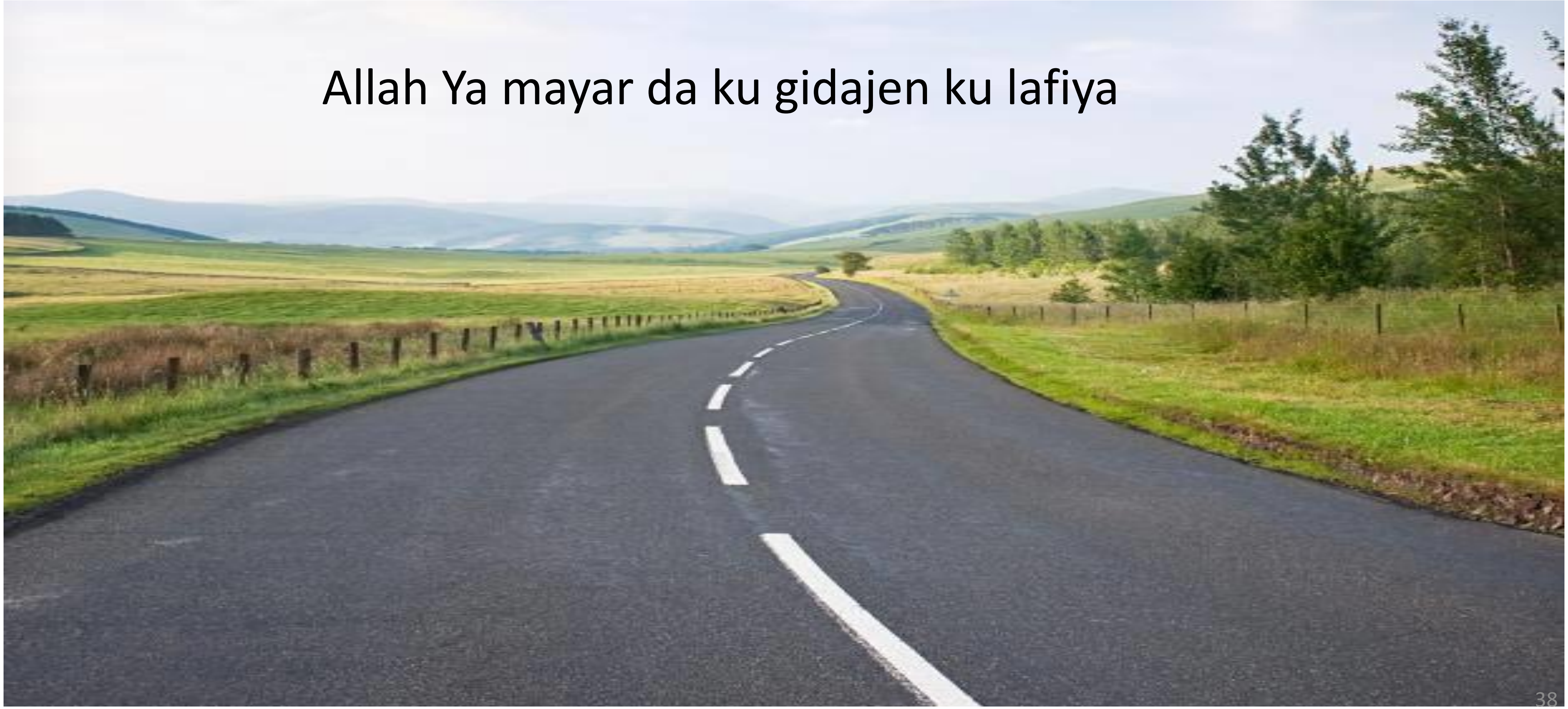

Allah Ya mayar da ku gidajen ku lafiya
