# Supplementary material for: Development of culturally sensitive pain neuroscience education materials for Hausa-speaking patients with chronic spinal pain: A modified Delphi study
Source: PLoS One. 2021 Jul 2;16(7):e0253757. doi: 10.1371/journal.pone.0253757 (PMC8253446; doi:10.1371/journal.pone.0253757)

YOU ARE WELCOME!

PAIN IN MOTION

200 YEARS
GHENT UNIVERSITY

FACULTY OF MEDICINE AND HEALTH SCIENCES

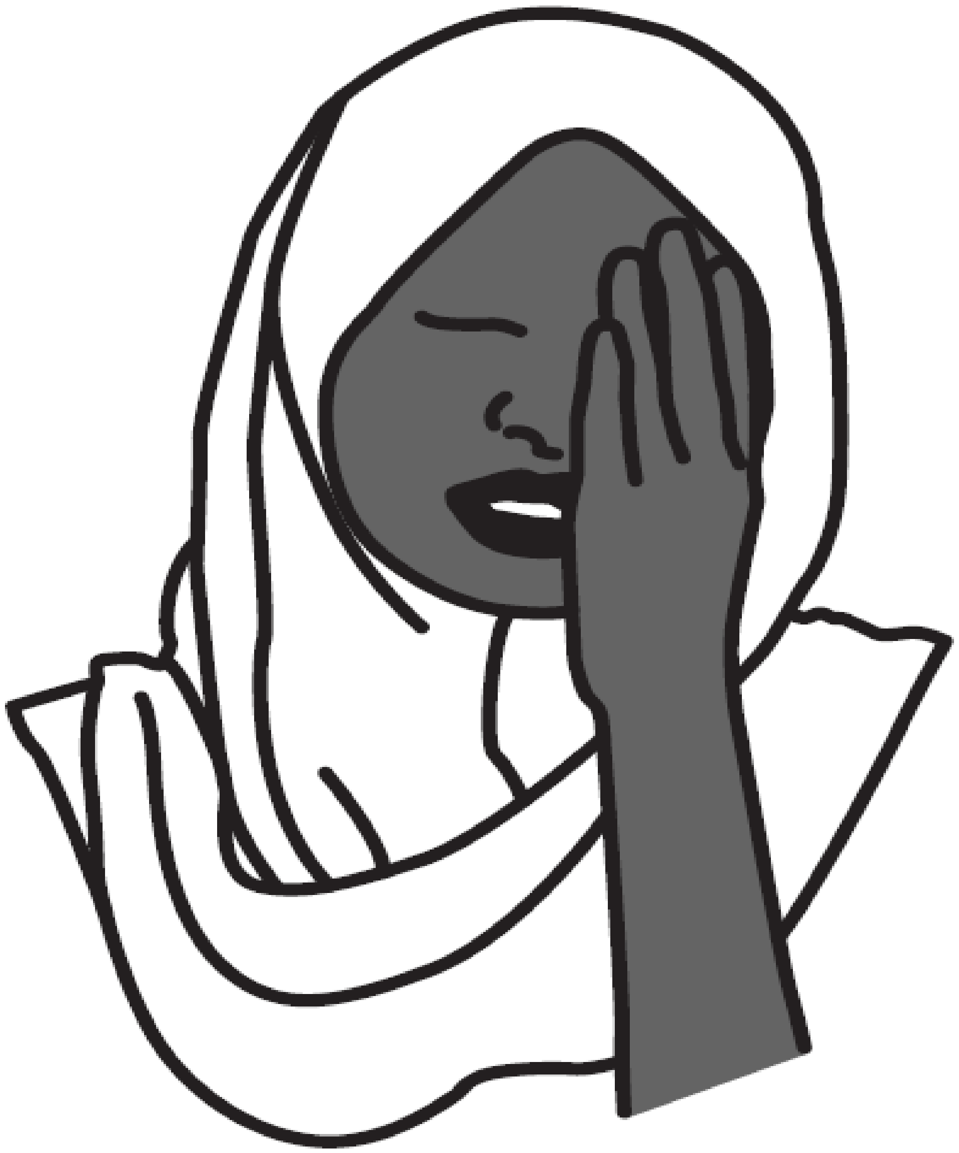

PAIN

WHAT YOU NEED TO KNOW ABOUT IT

Is pain useful at all?

Let’s learn some things about our pain.

PAIN

- ❑Its meaning?
- ❑How I got it?
- ❑Its purpose?
- ❑Experiences derived from pain?

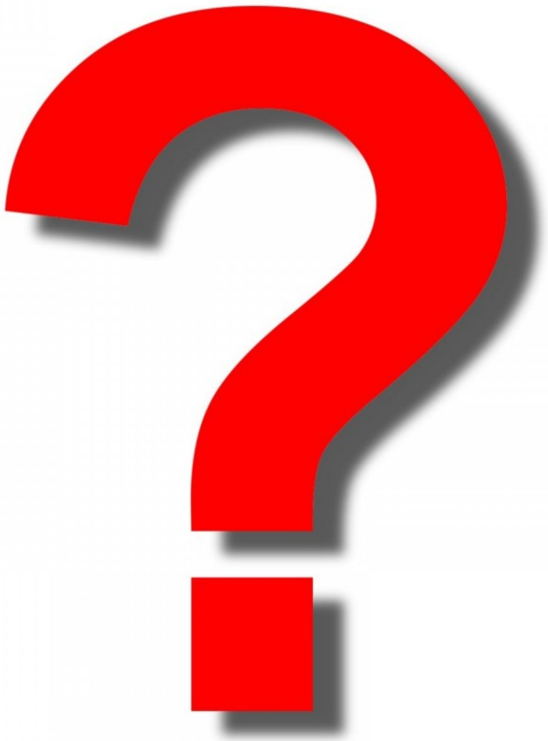

PAIN

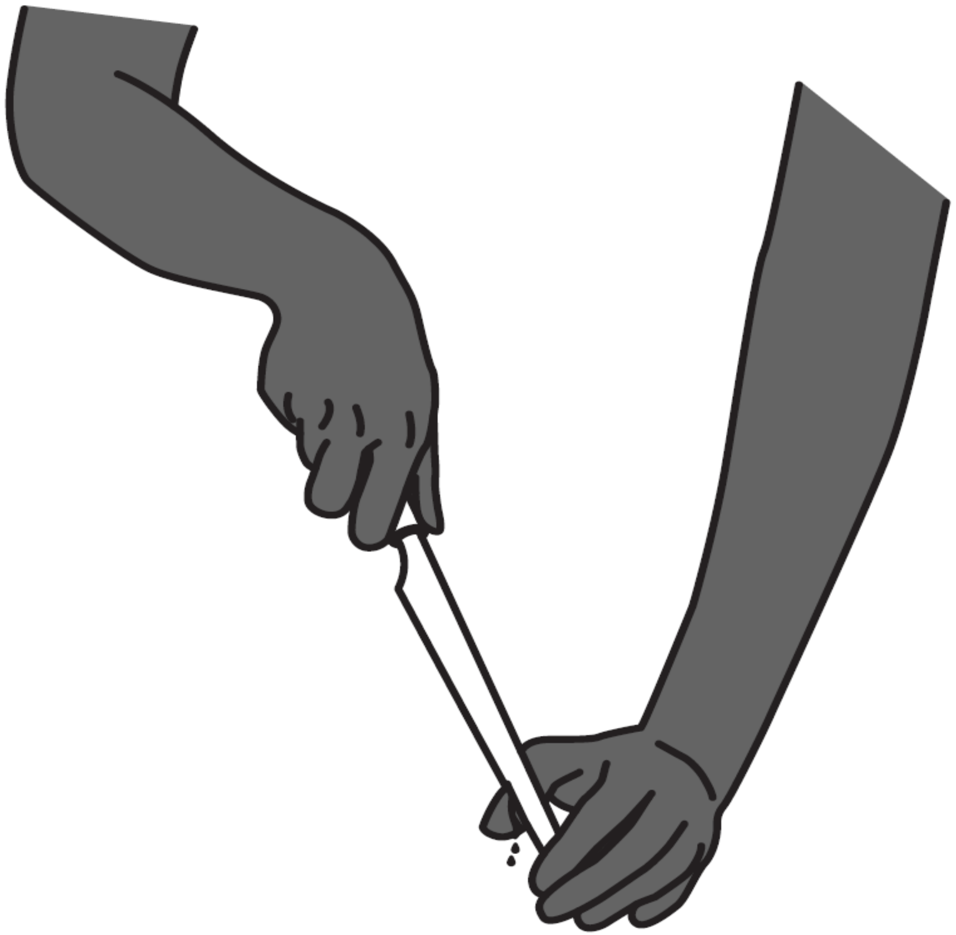

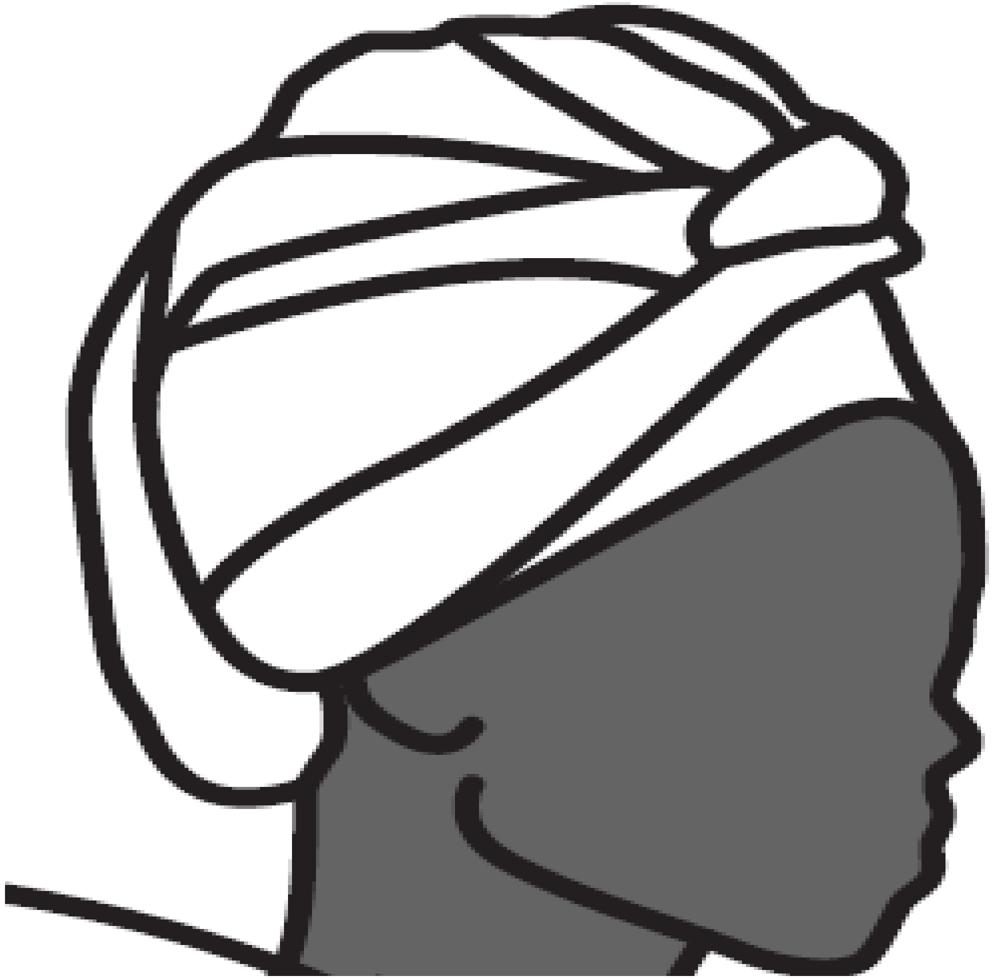

PAIN
(Experience)

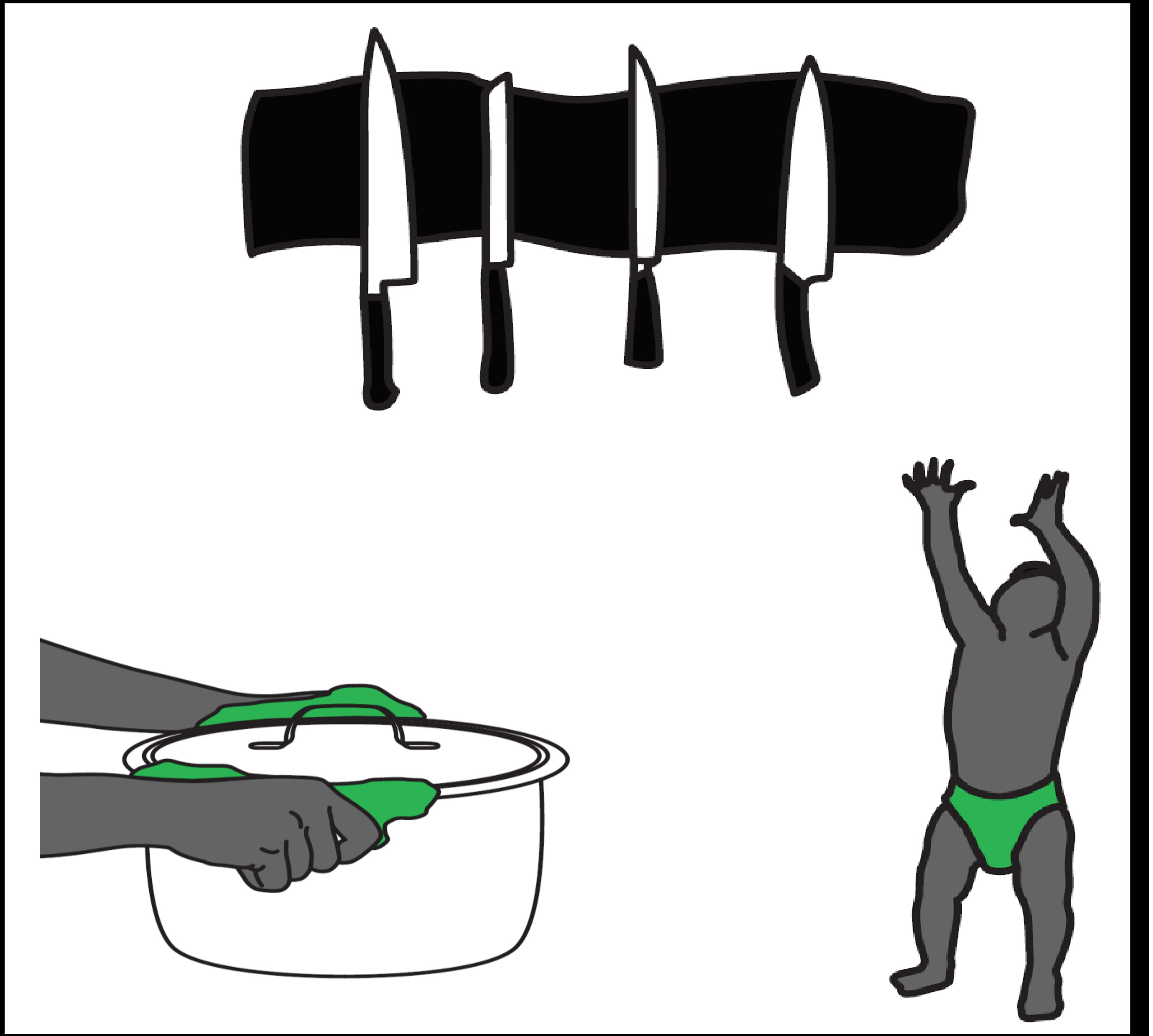

What is PAIN?

Pain: natural way of protecting body

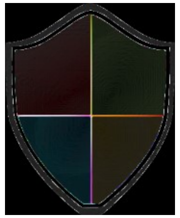

Alerts the body of potential danger

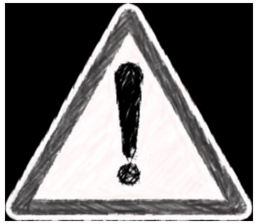

Body responds

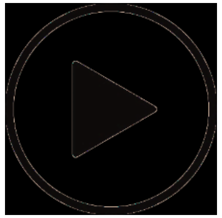

SURVIVAL

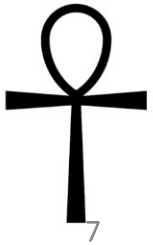

PAIN IS AN ALARM TO THE BODY

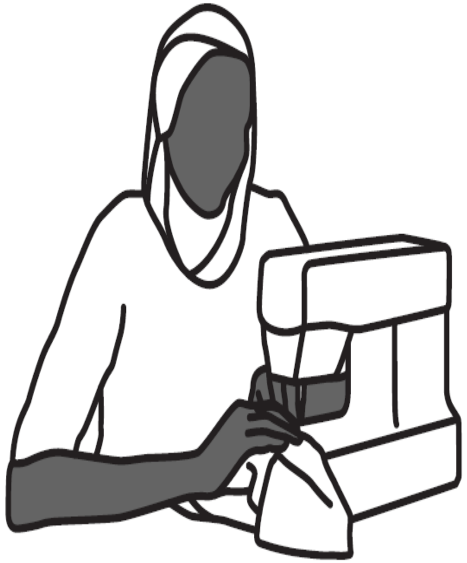

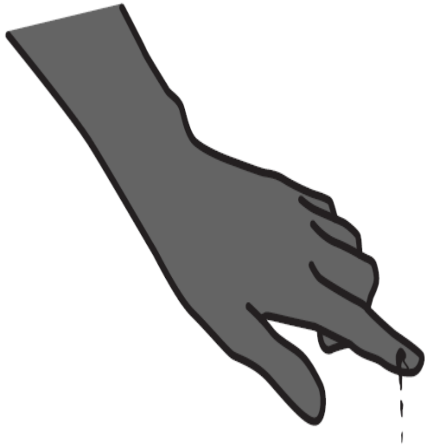

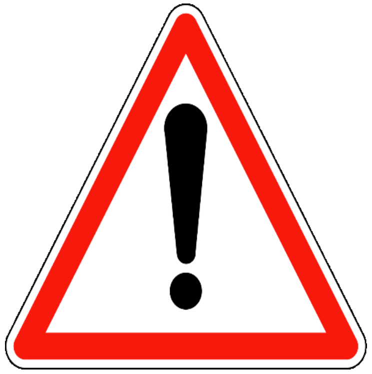

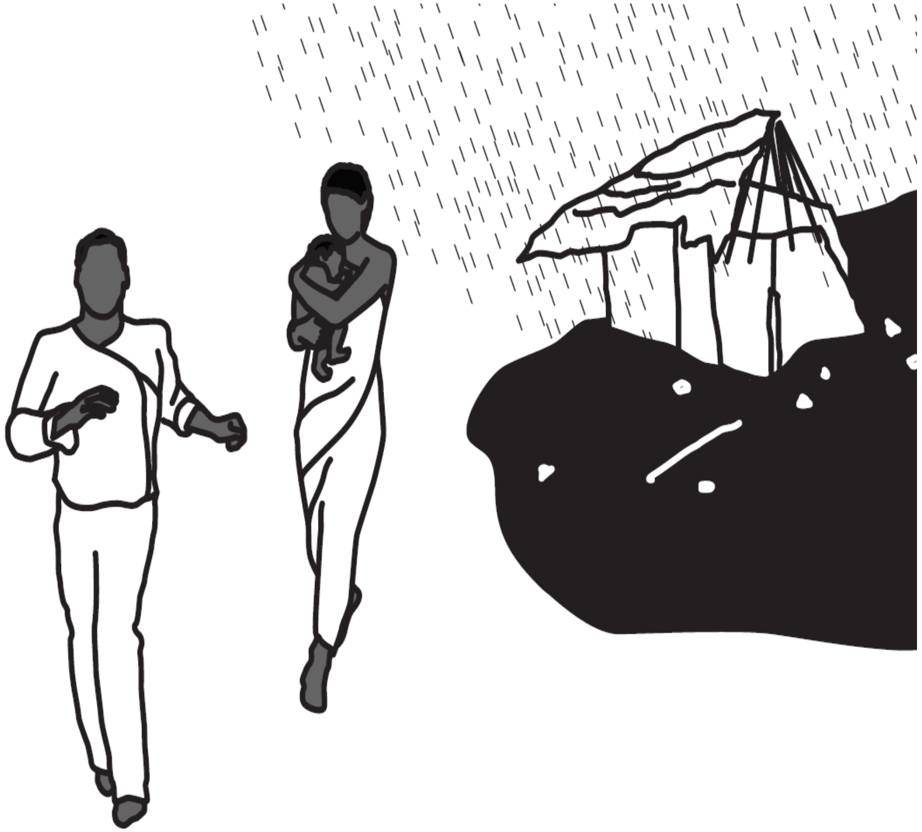

PAIN
alerts us

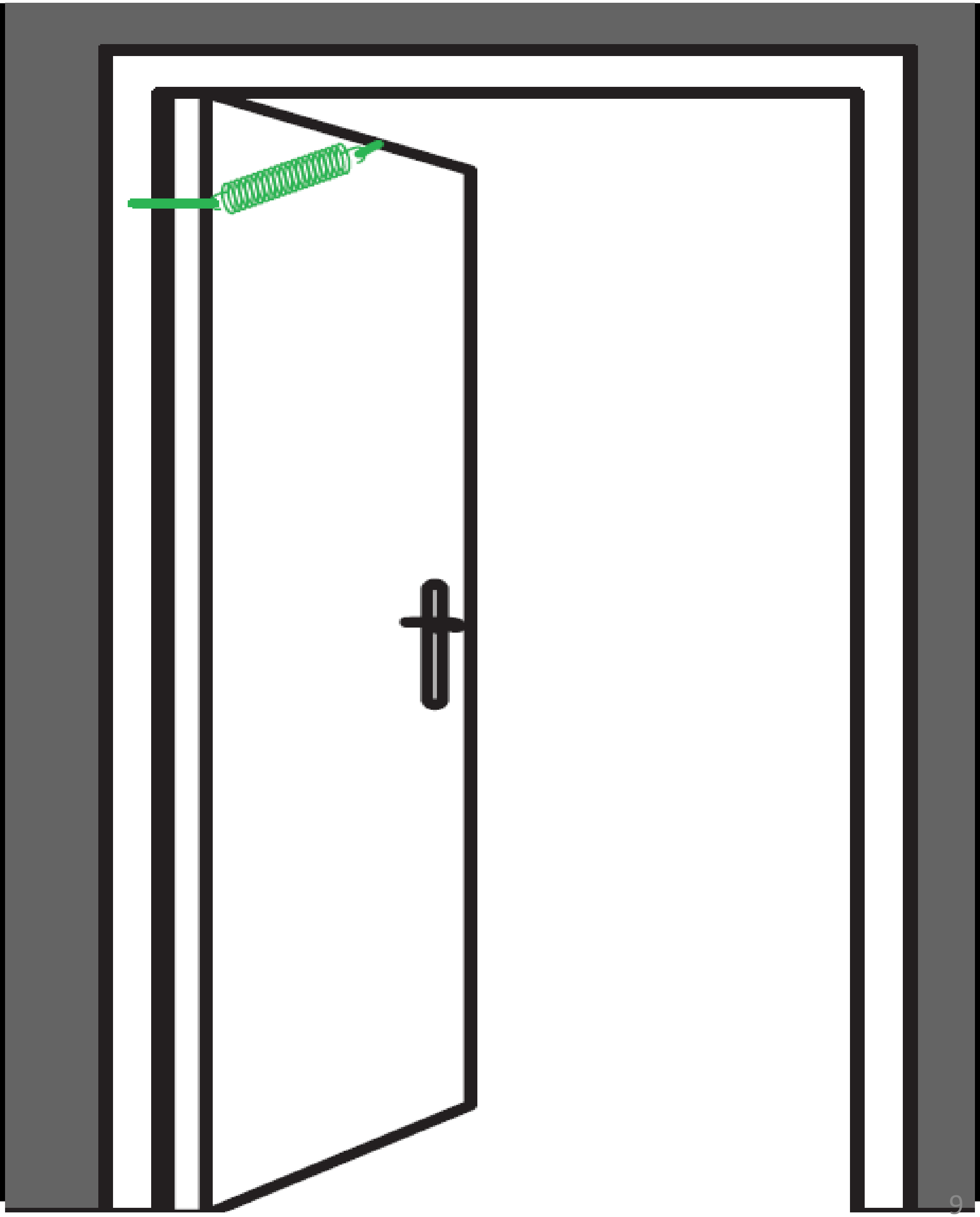

PAIN HELPS

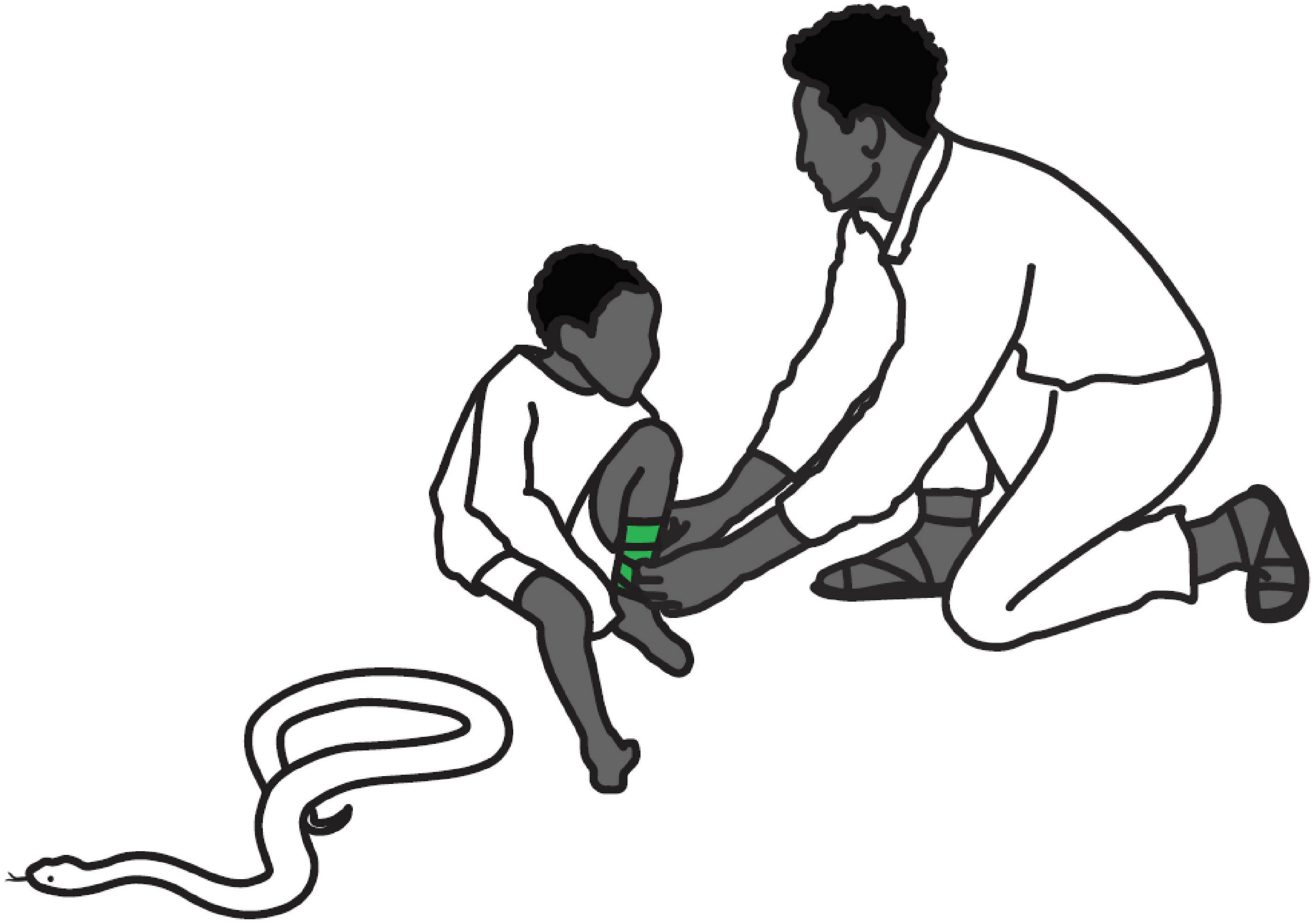

DANGERS OF NO PAIN

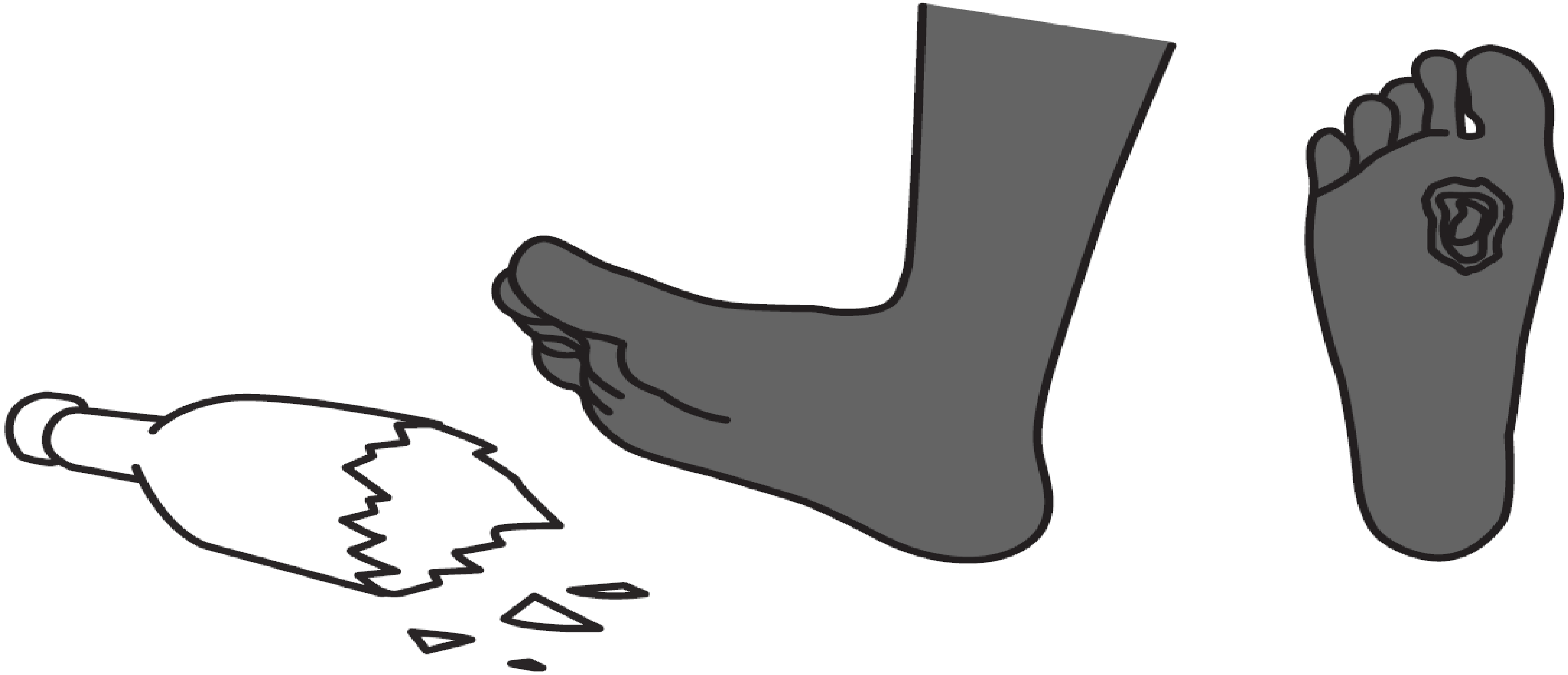

PAIN CLASSIFICATIONS

ACUTE

(NEW)

CHRONIC

(PERSISTENT)

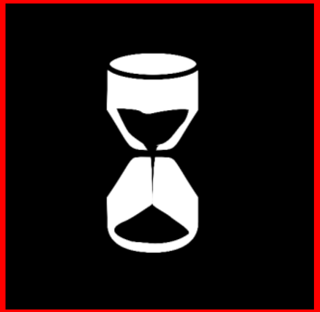

ACUTE PAIN

- ✓ Mostly have a clear cause
- ✓ Pain following injury

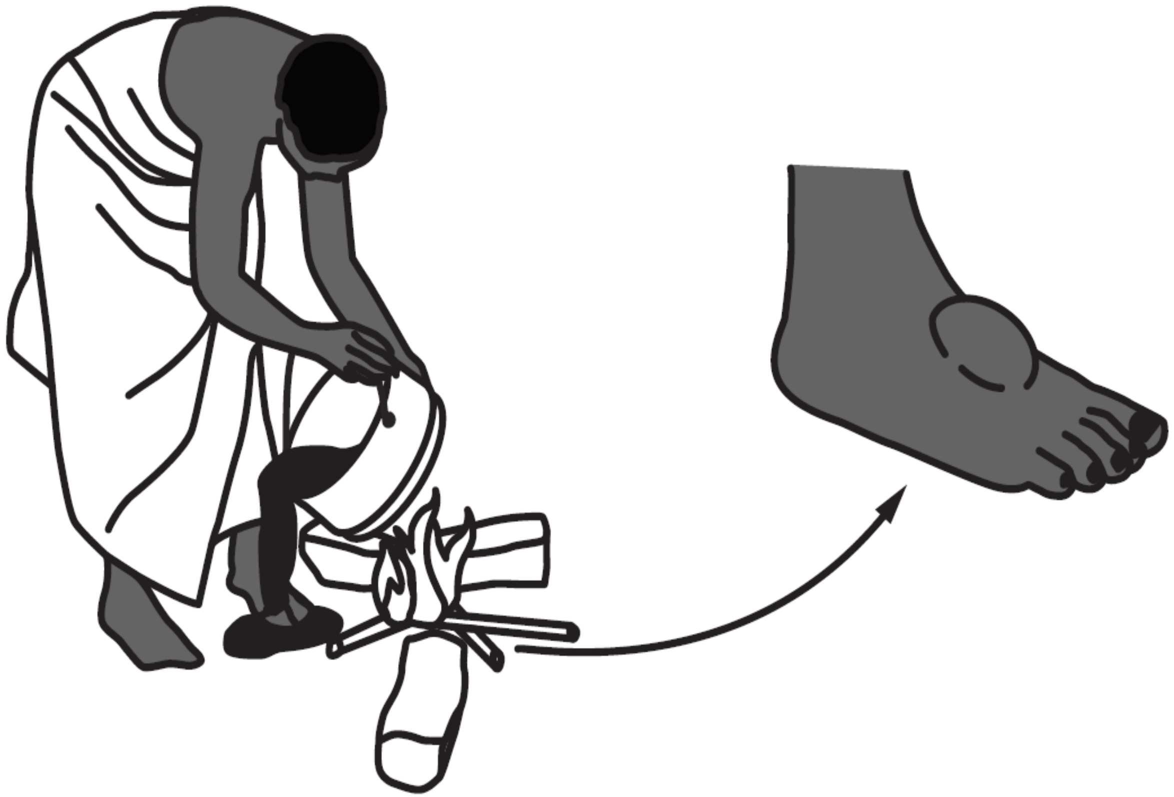

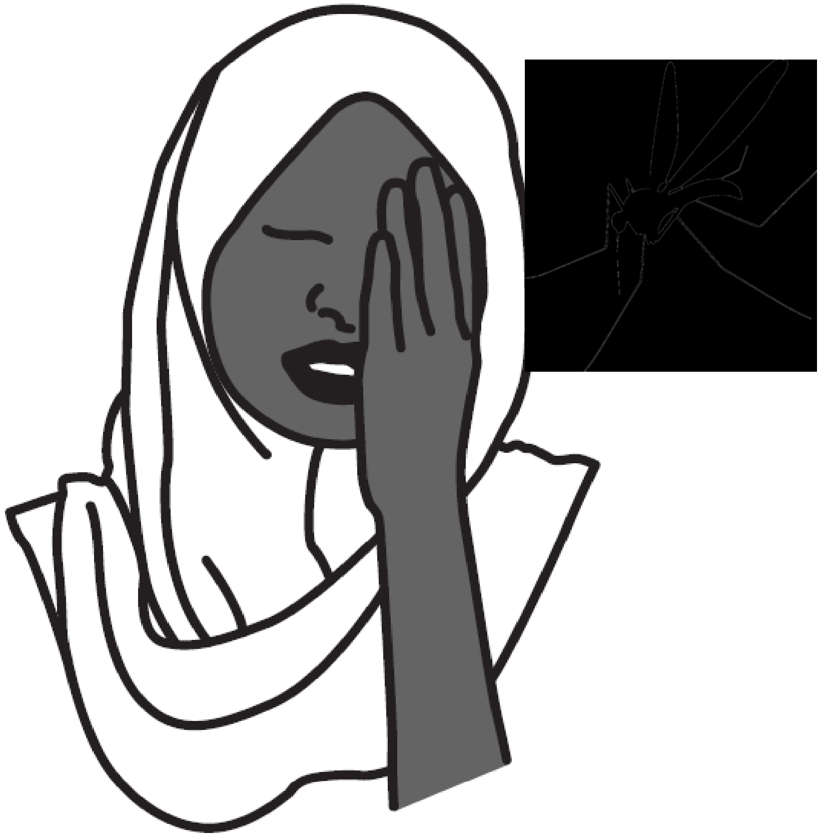

NERVOUS SYSTEM

Nerve cell

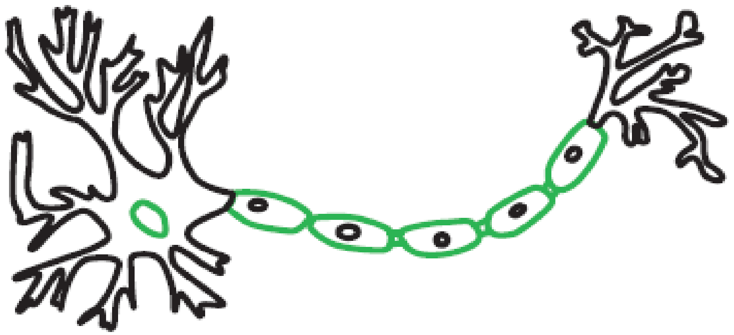

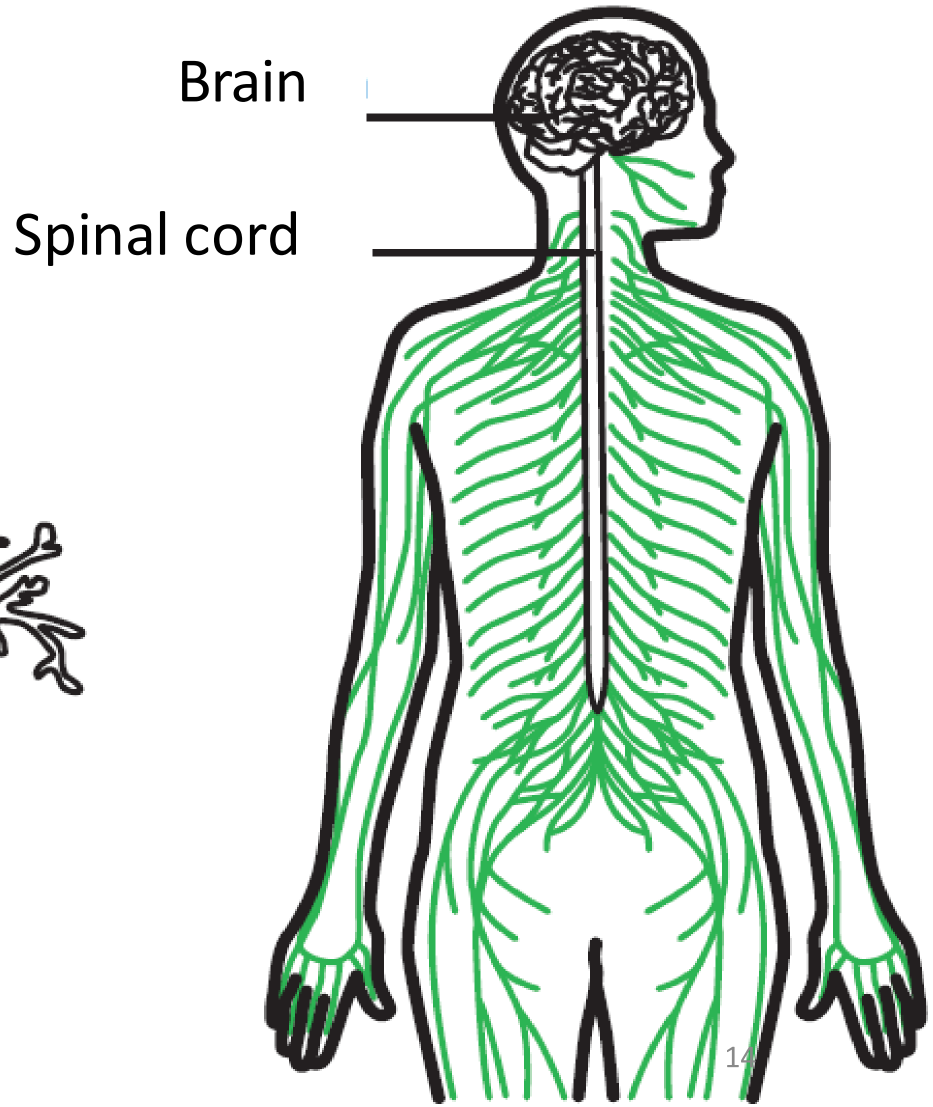

RECEPTORS

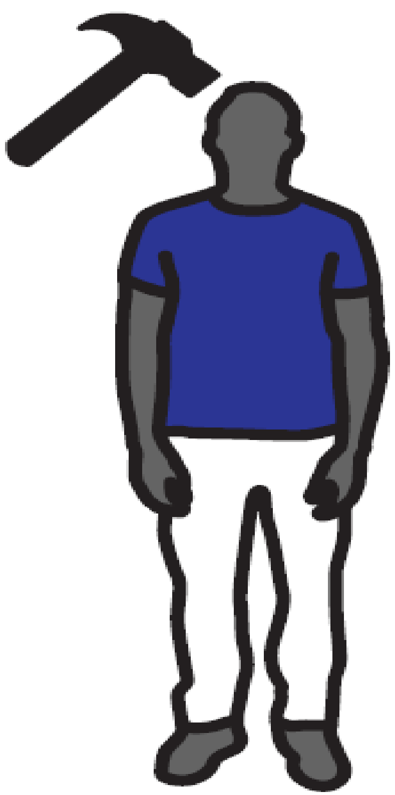

Danna

Mechanical

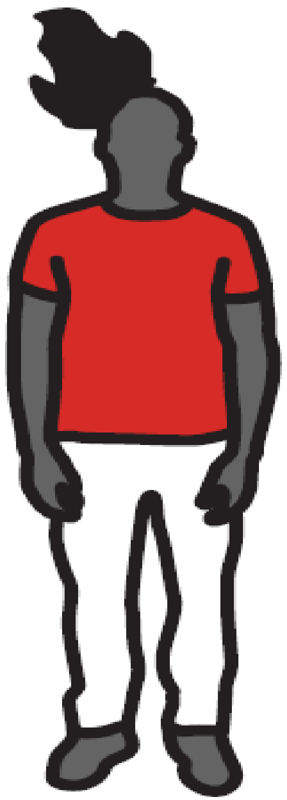

Zafi/Sanyi

Temperature

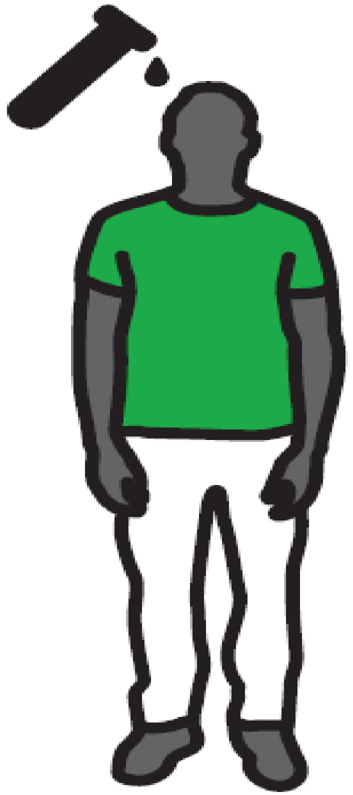

Sinadari/Asid

Acid/Chemical

RECEPTORS ON NERVES

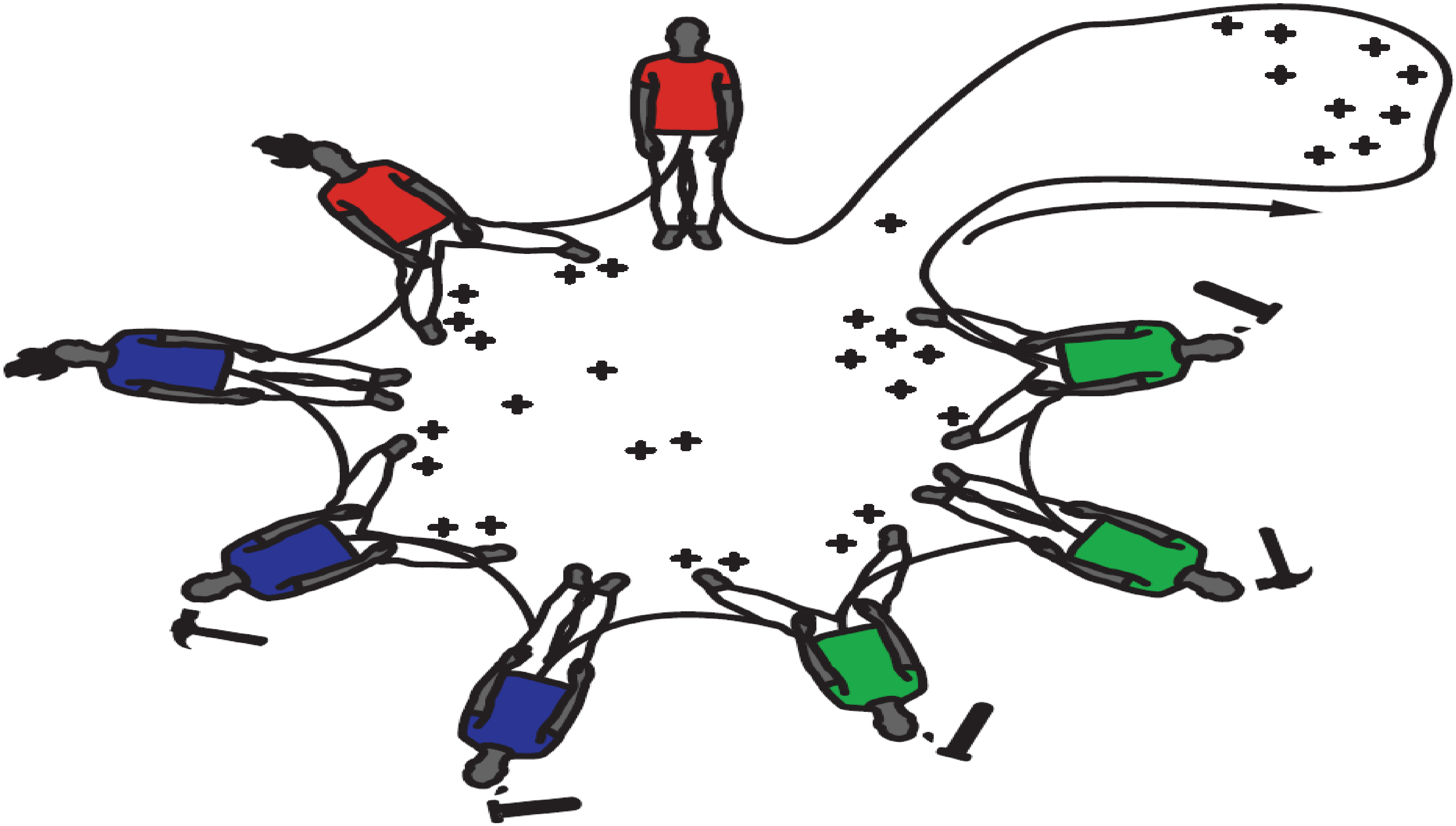

ACUTE PAIN MECHANSIM

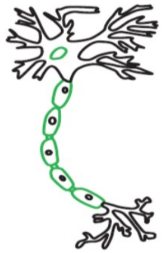

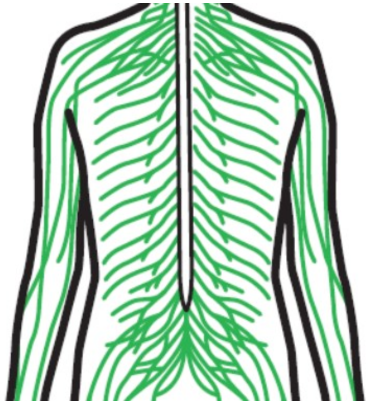

Receptor

Sensory nerves

Spinal cord

- Brain
  - PAIN
- Motor nerves
  - Reflex

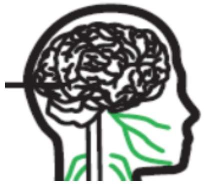

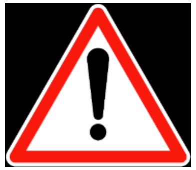

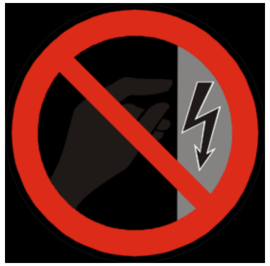

Pain ≠ Tissue damage ⟷ Tissue damage ≠ Pain

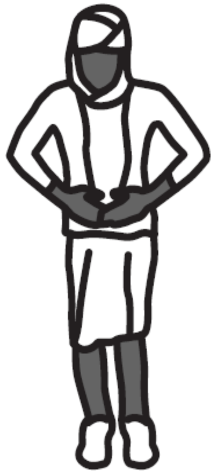

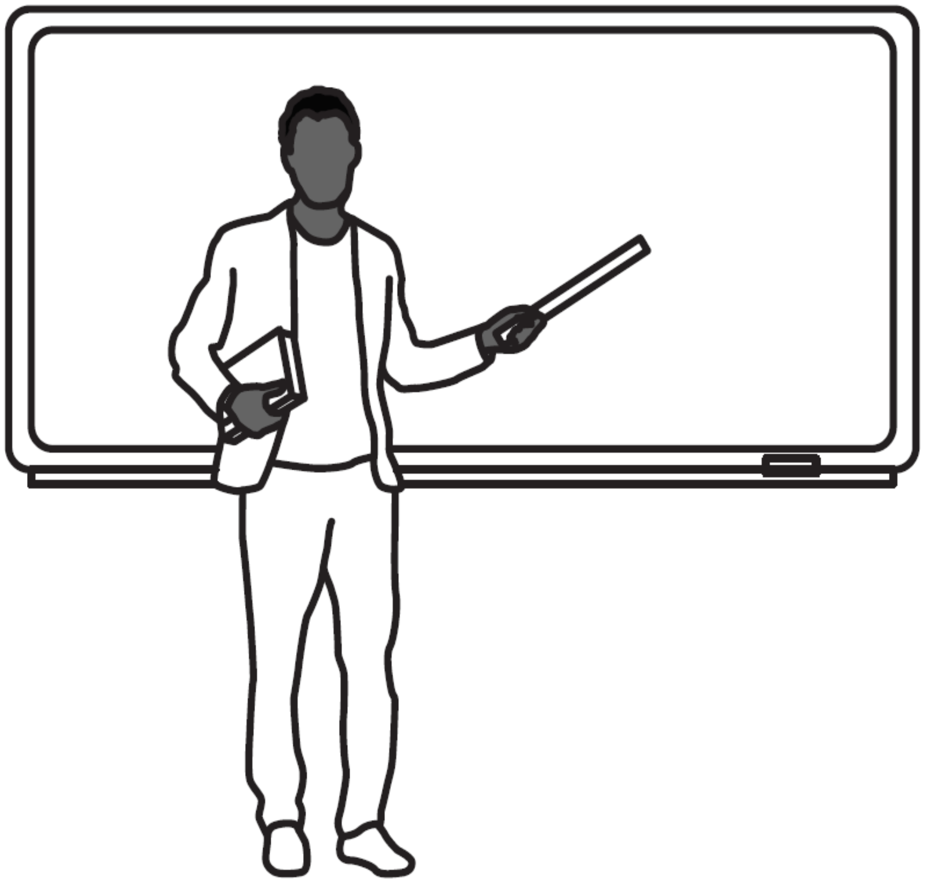

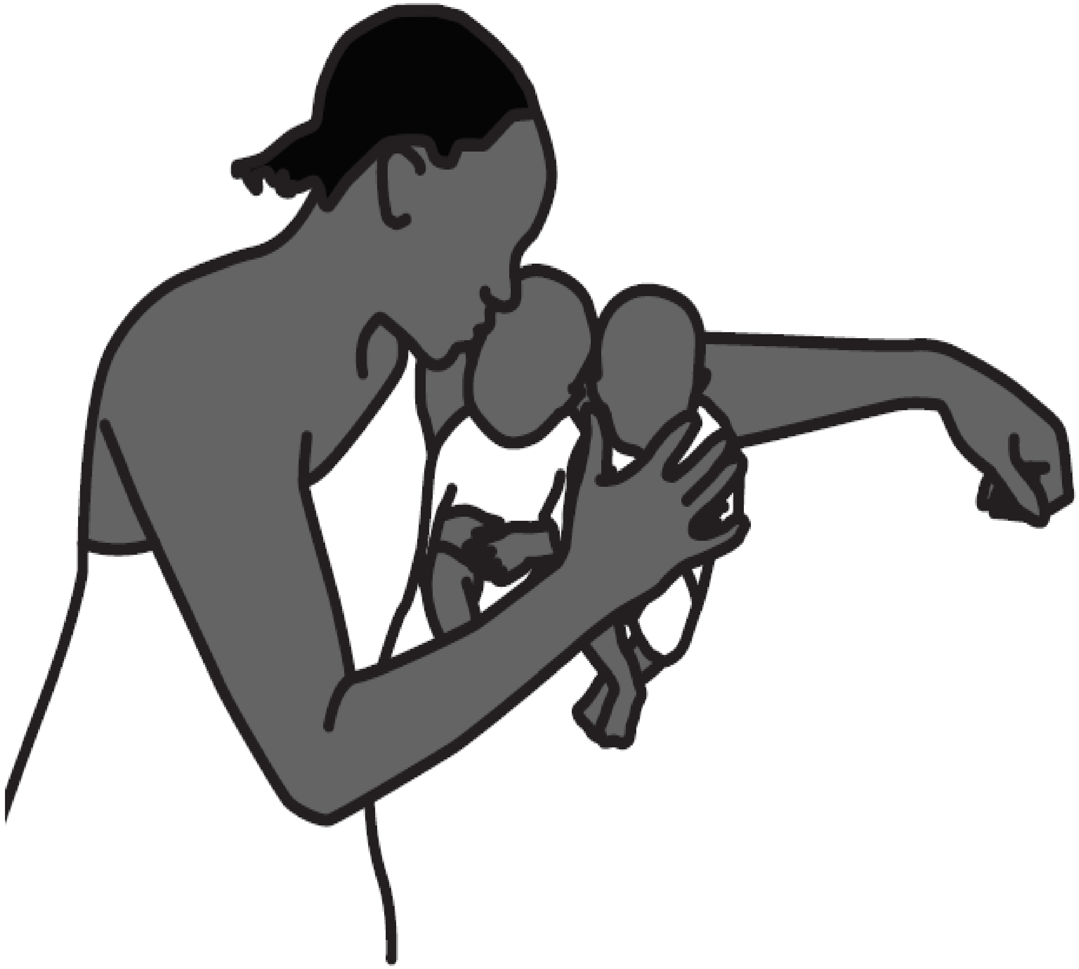

DAMAGE ≠ PAIN

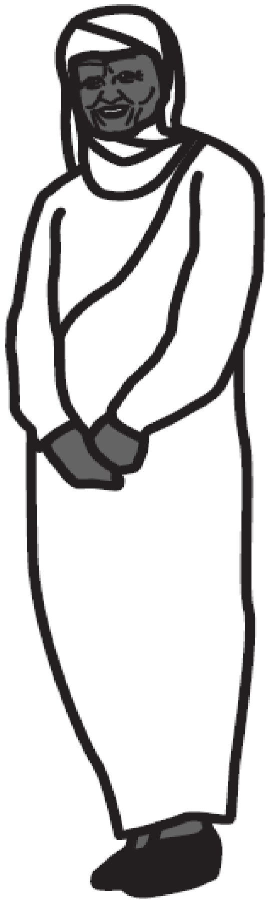

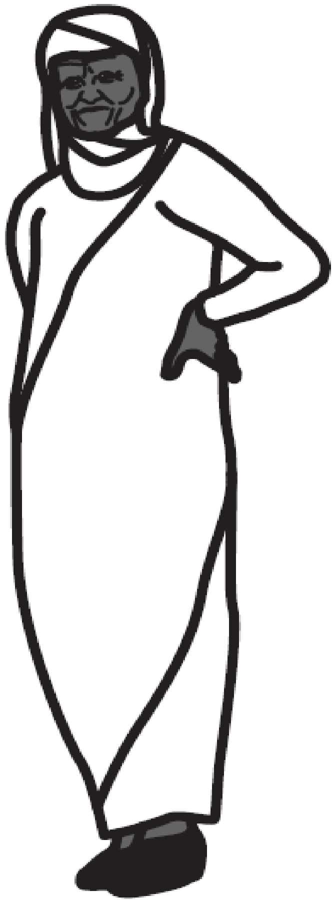

Age (years)

Imaging finding	20	50	80	Pain
Disc degeneration	37%	80%	96%	X
Disc height loss	24%	56%	84%	X
Disc protrusion	29%	36%	43%	X
Spondylolisthesis	3%	14%	50%	X

BRAIN MODULATION OF PAIN

Inhibitory

Facilitatory

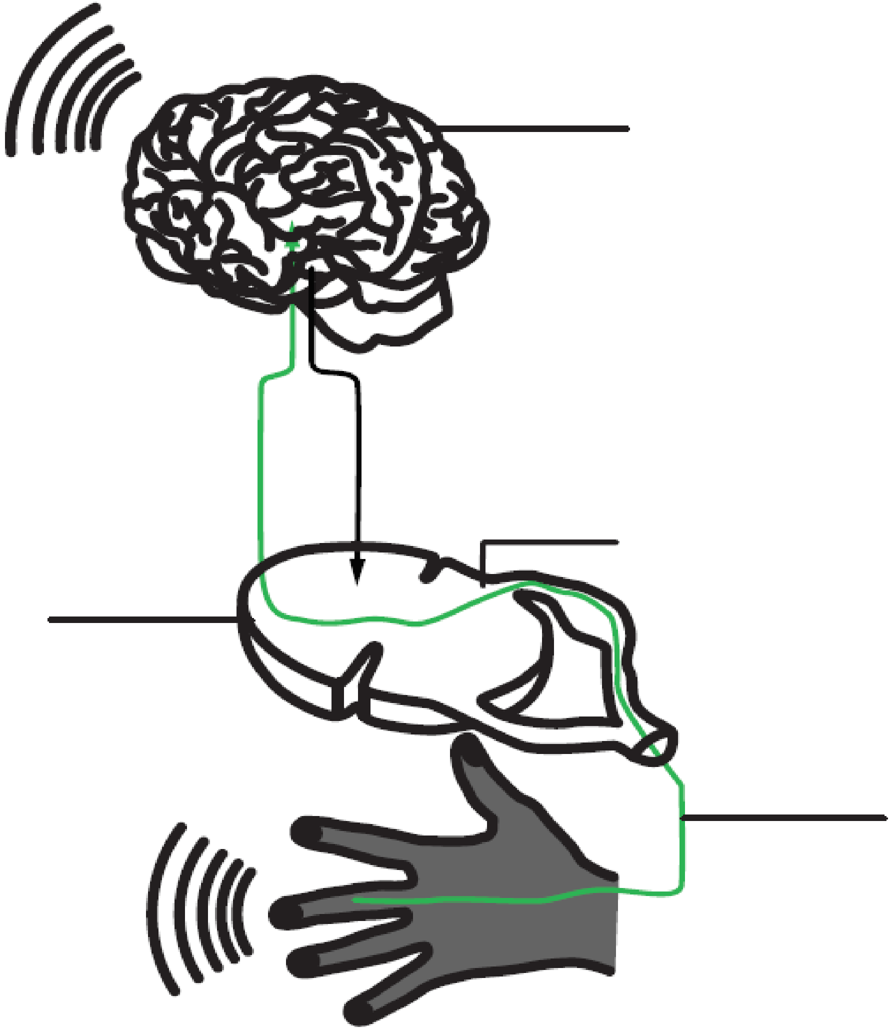

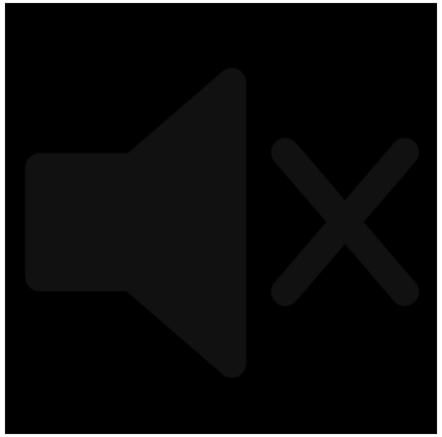

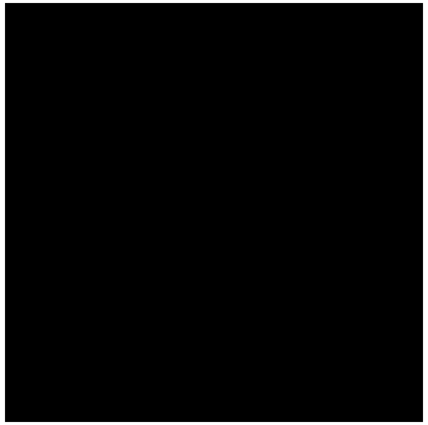

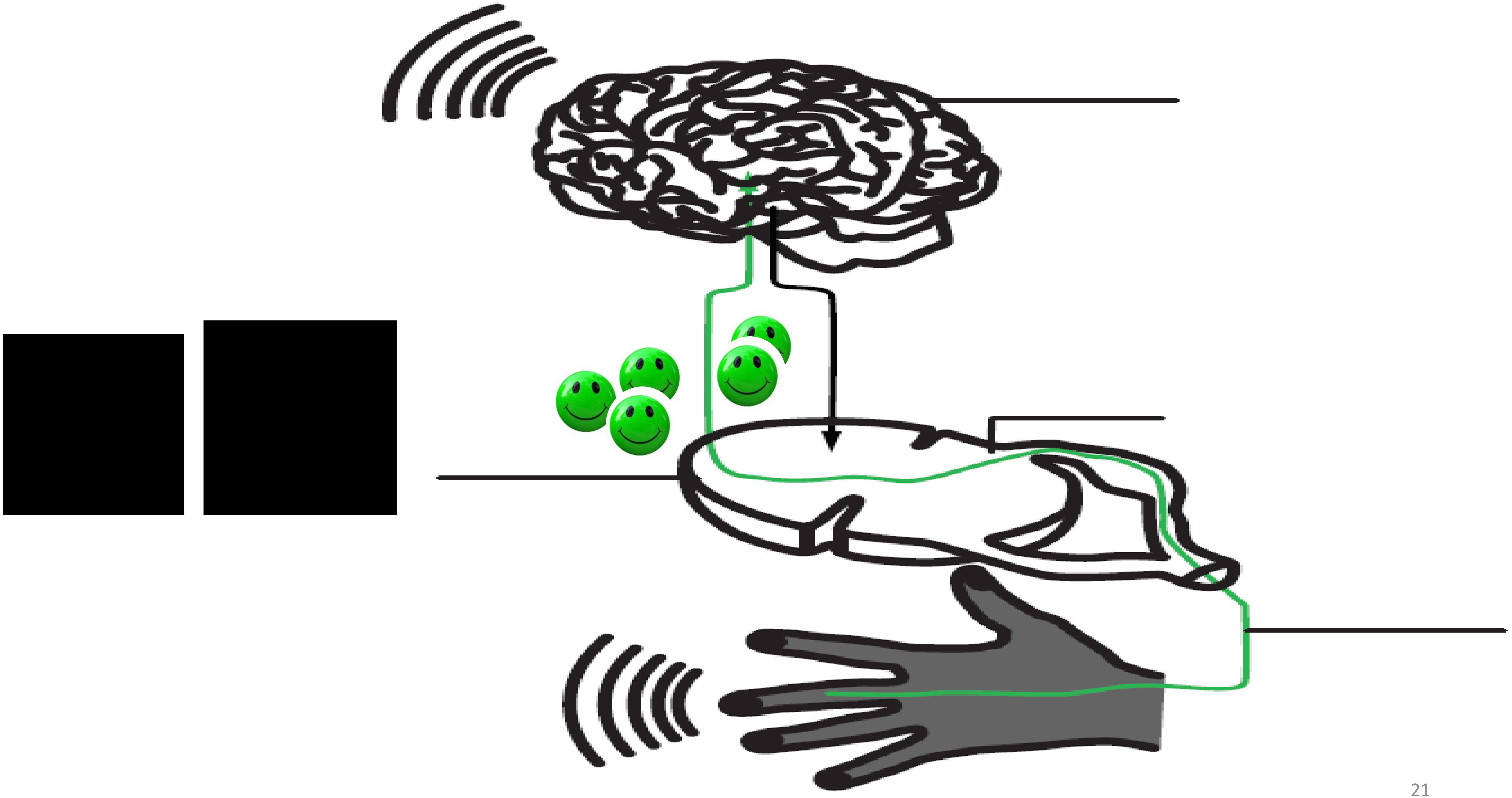

BRAIN: PAIN INHIBITION

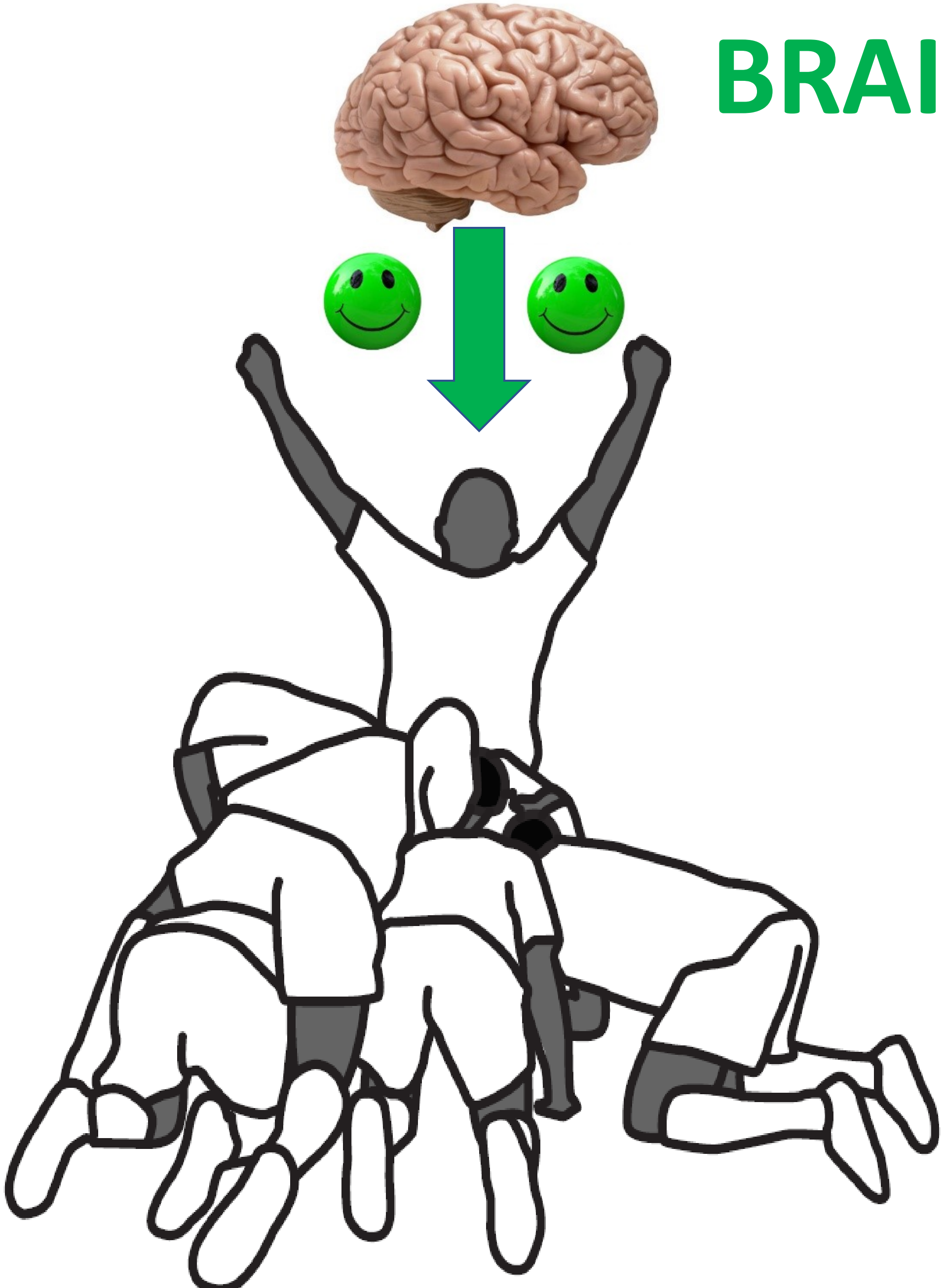

60x

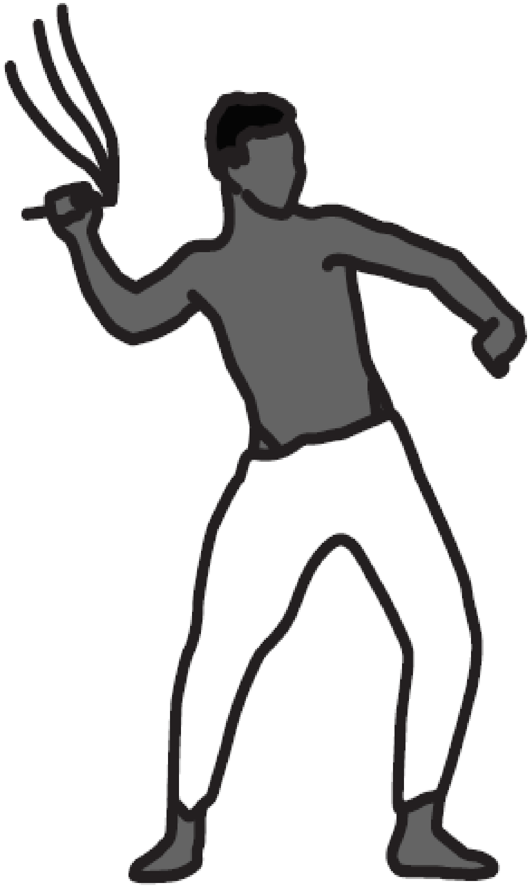

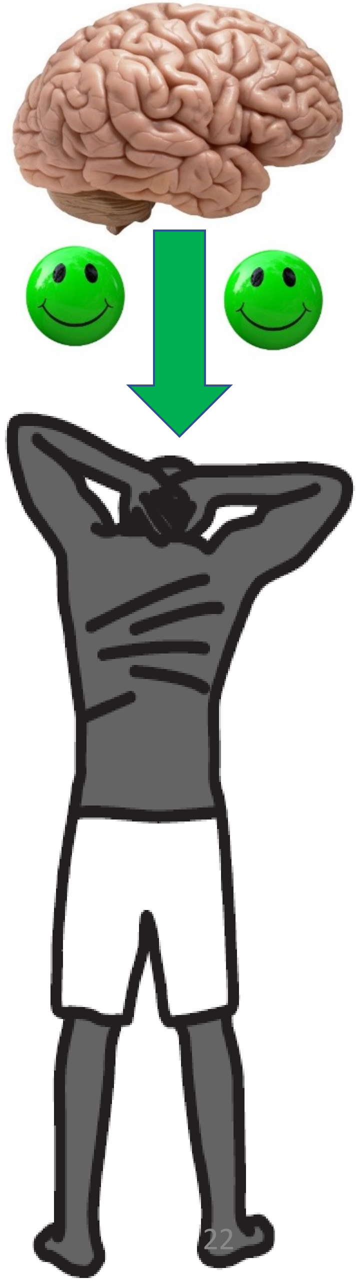

BRAIN: PAIN FACILITATION

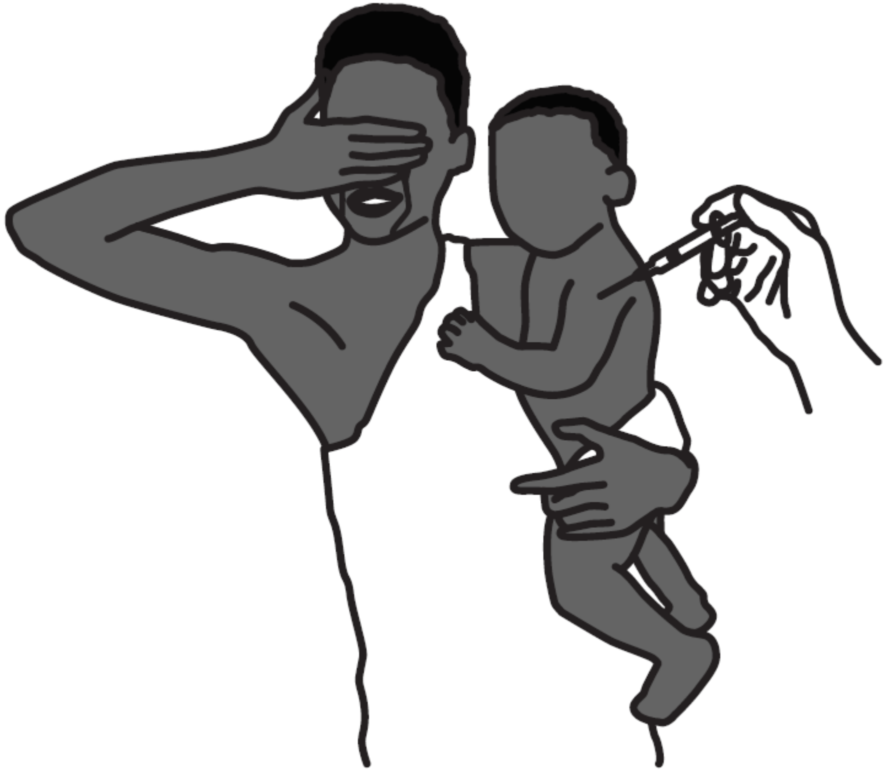

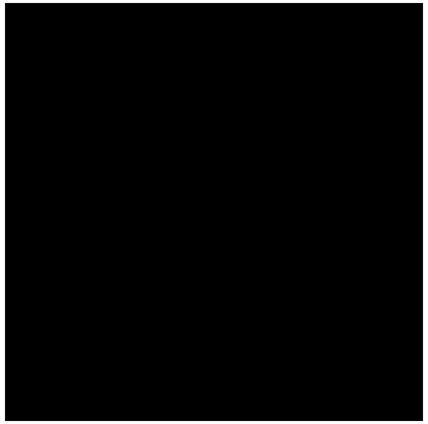

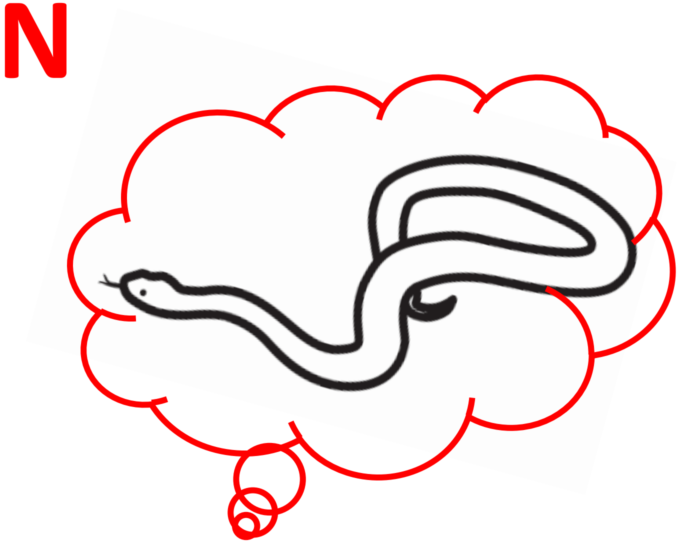

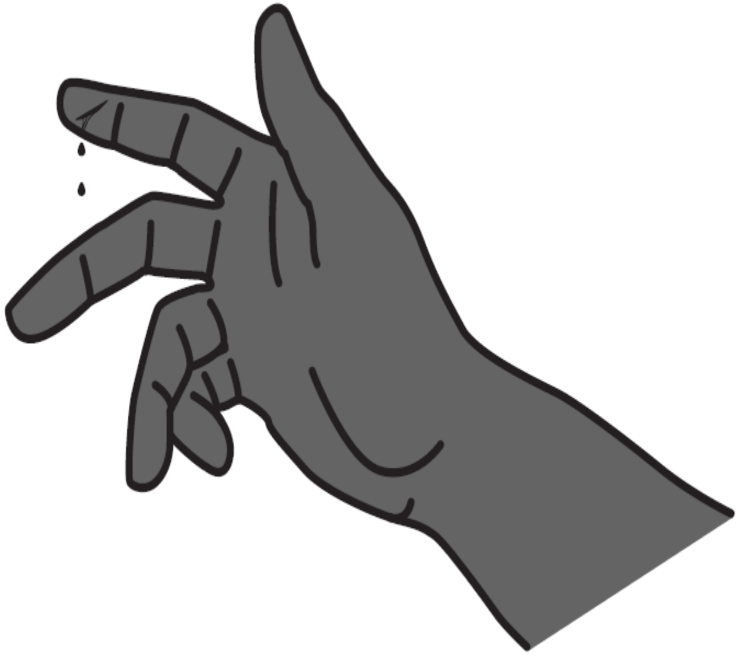

BRAIN: PAIN FACILITATION

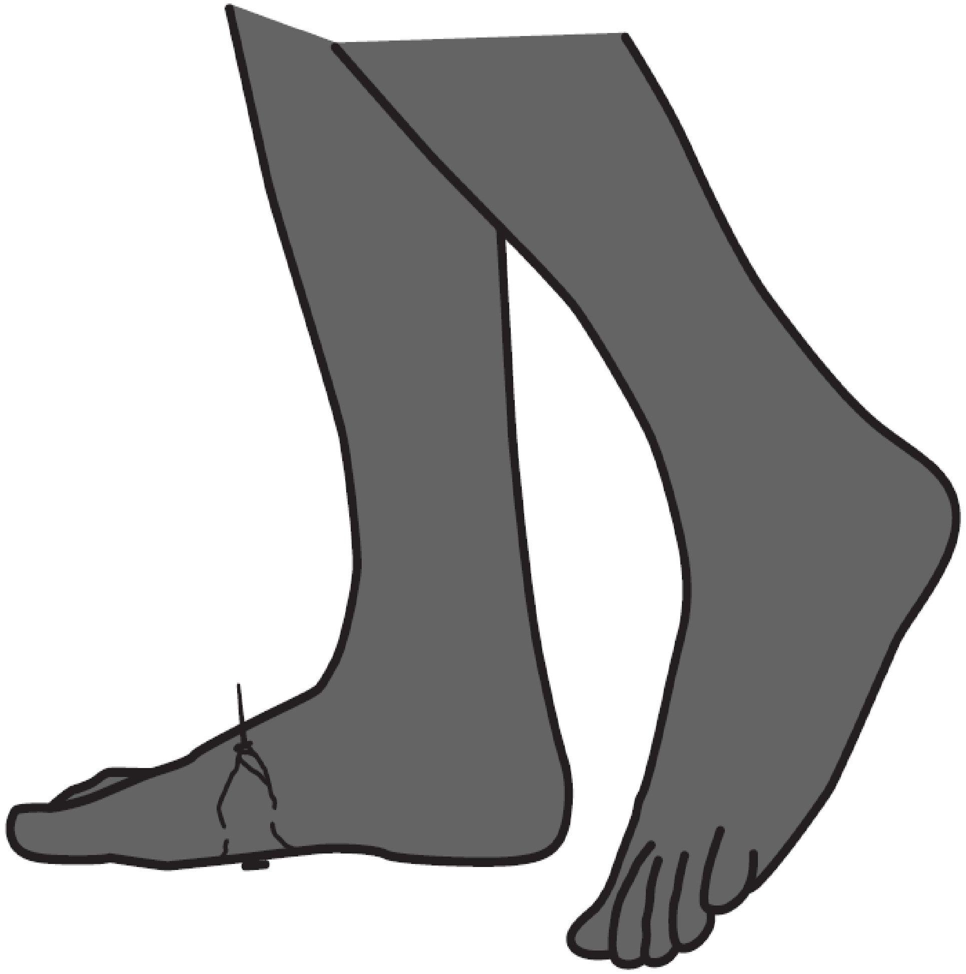

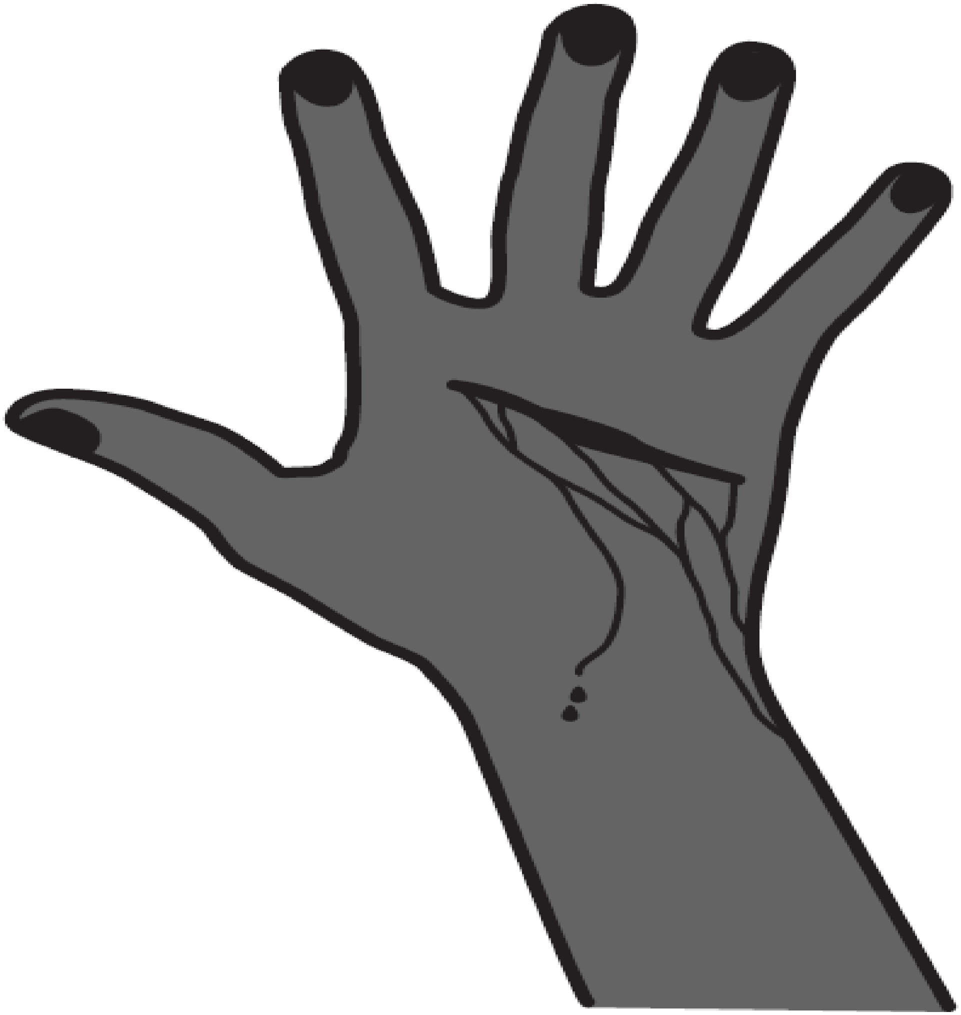

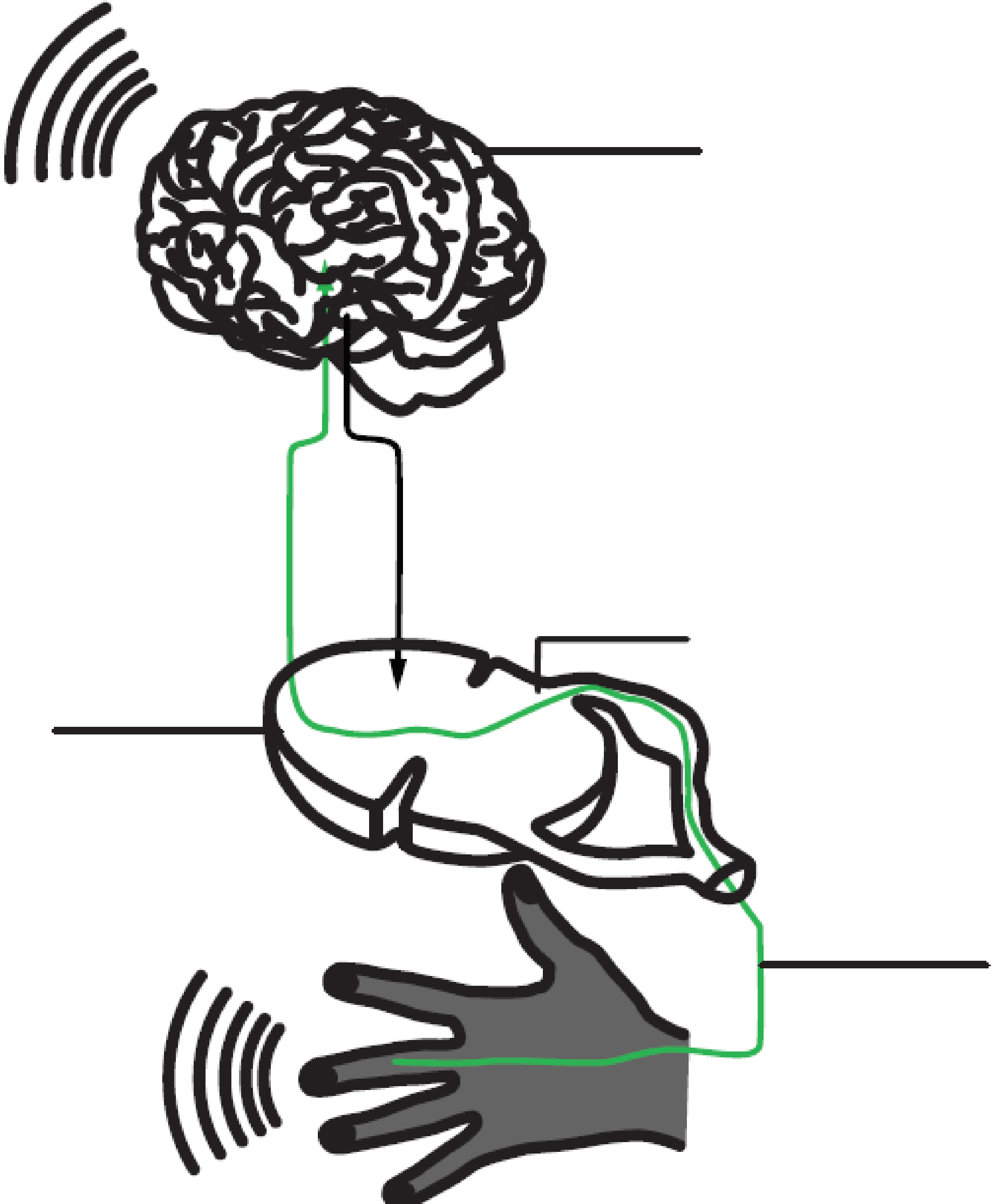

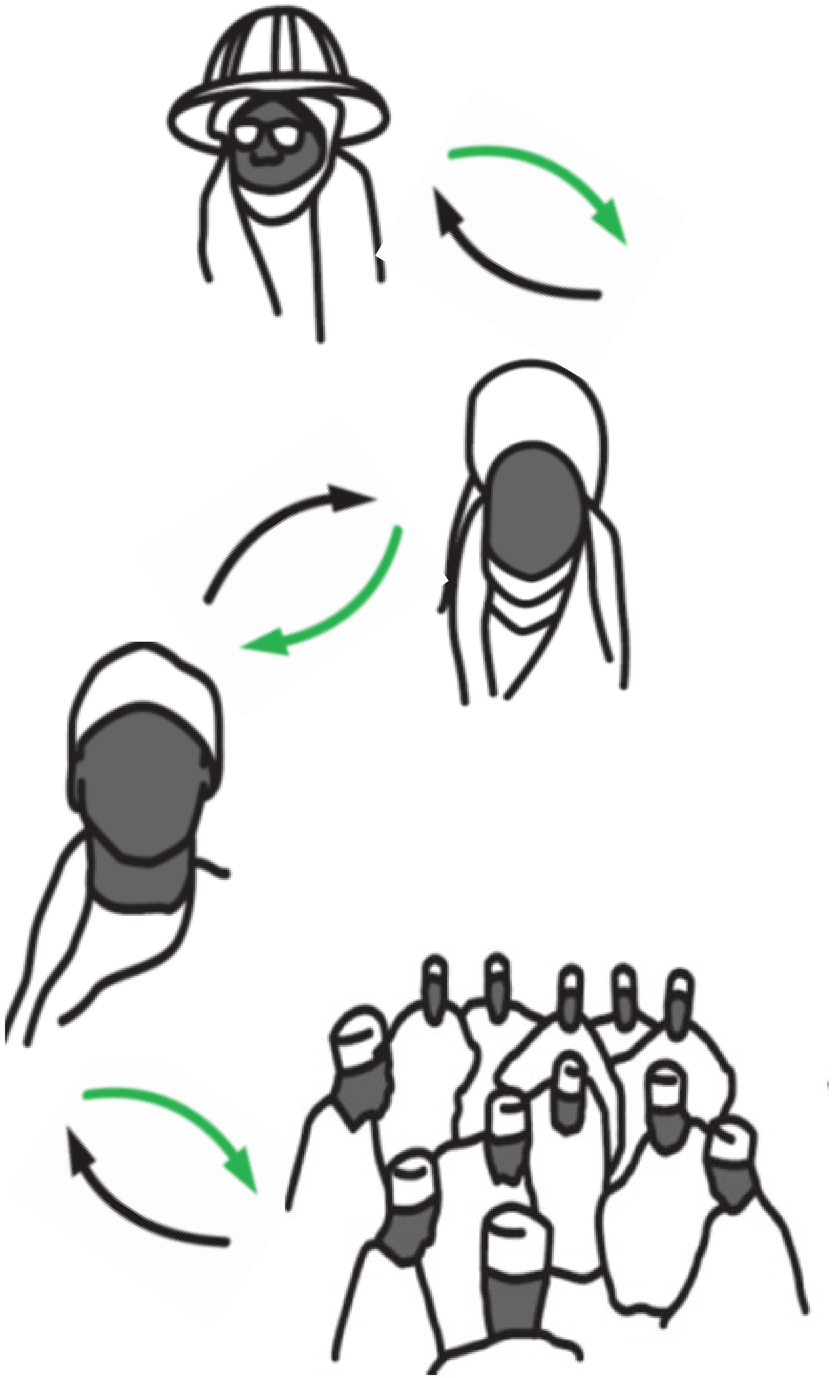

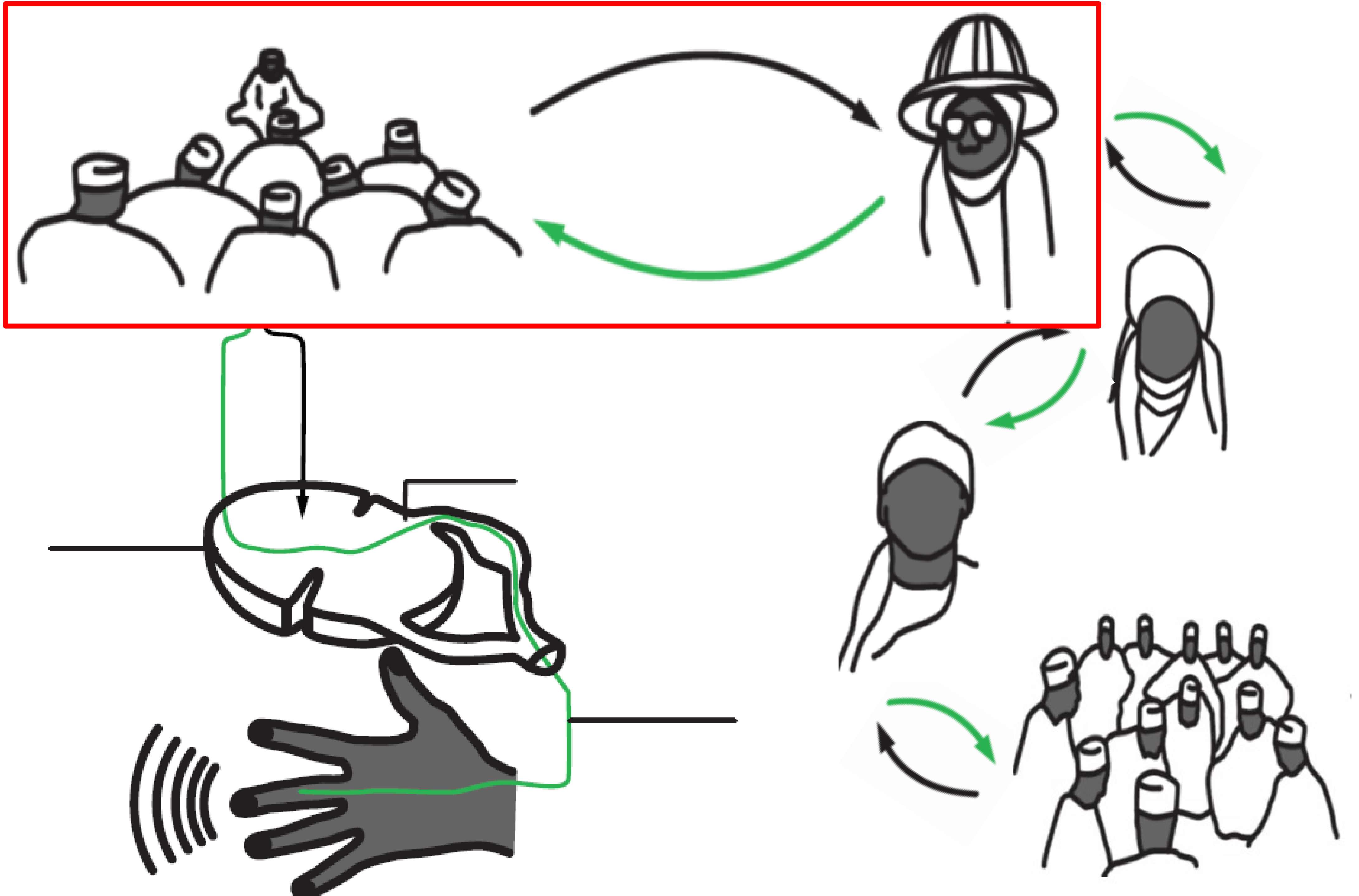

CHRONIC PAIN

- ✓ Pain > 3 months
- ✓ No clear definitive cause
- ✓ 1/5 of People

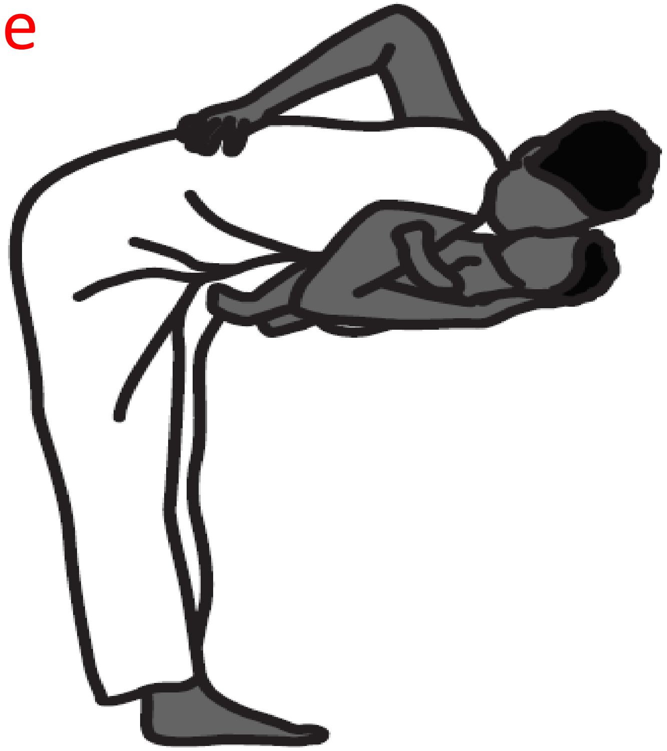

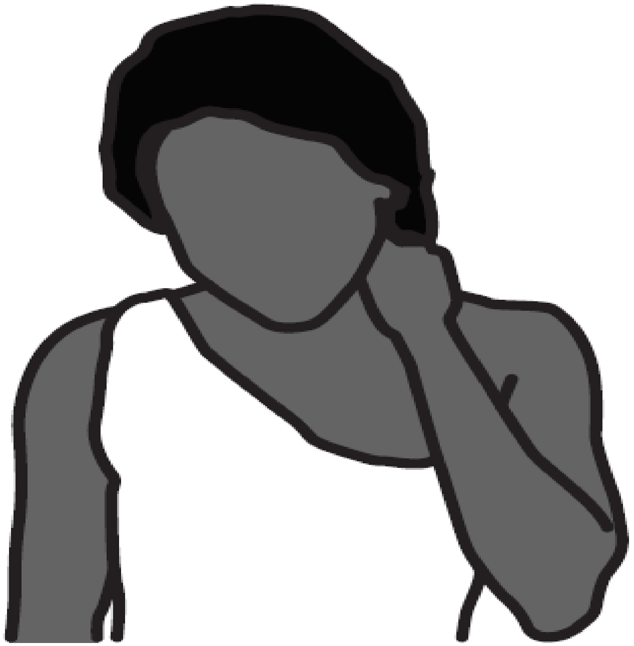

CHRONIC PAIN

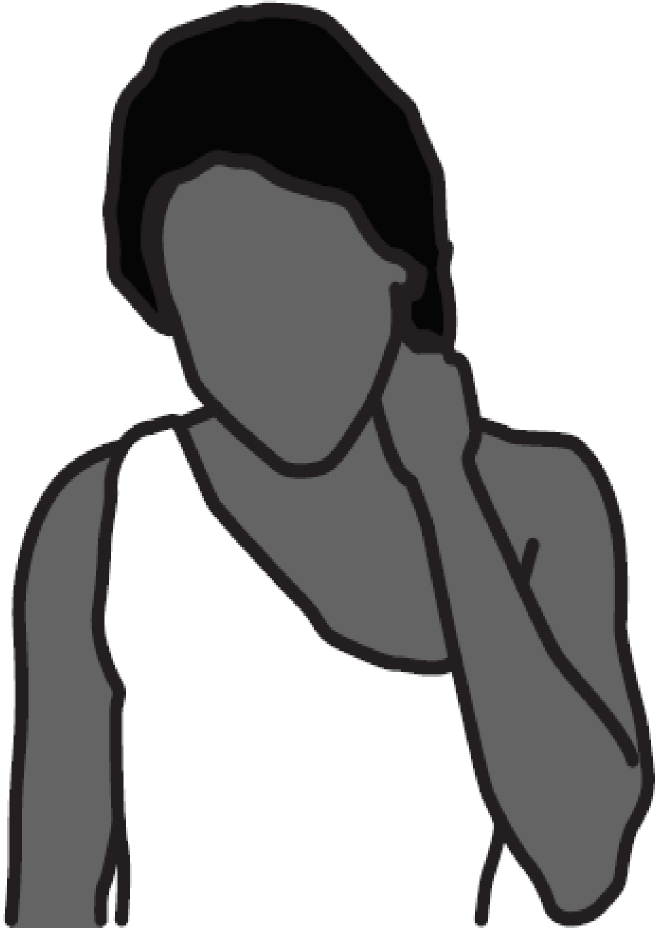

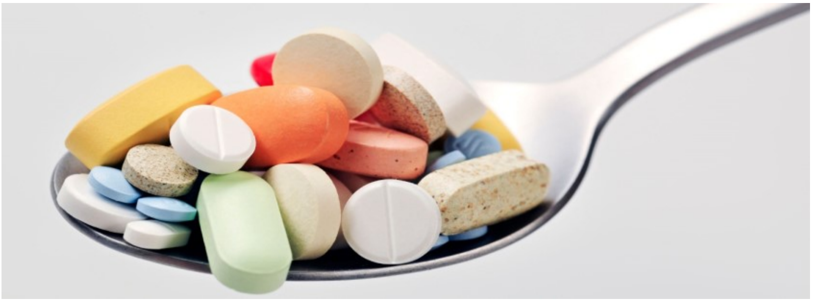

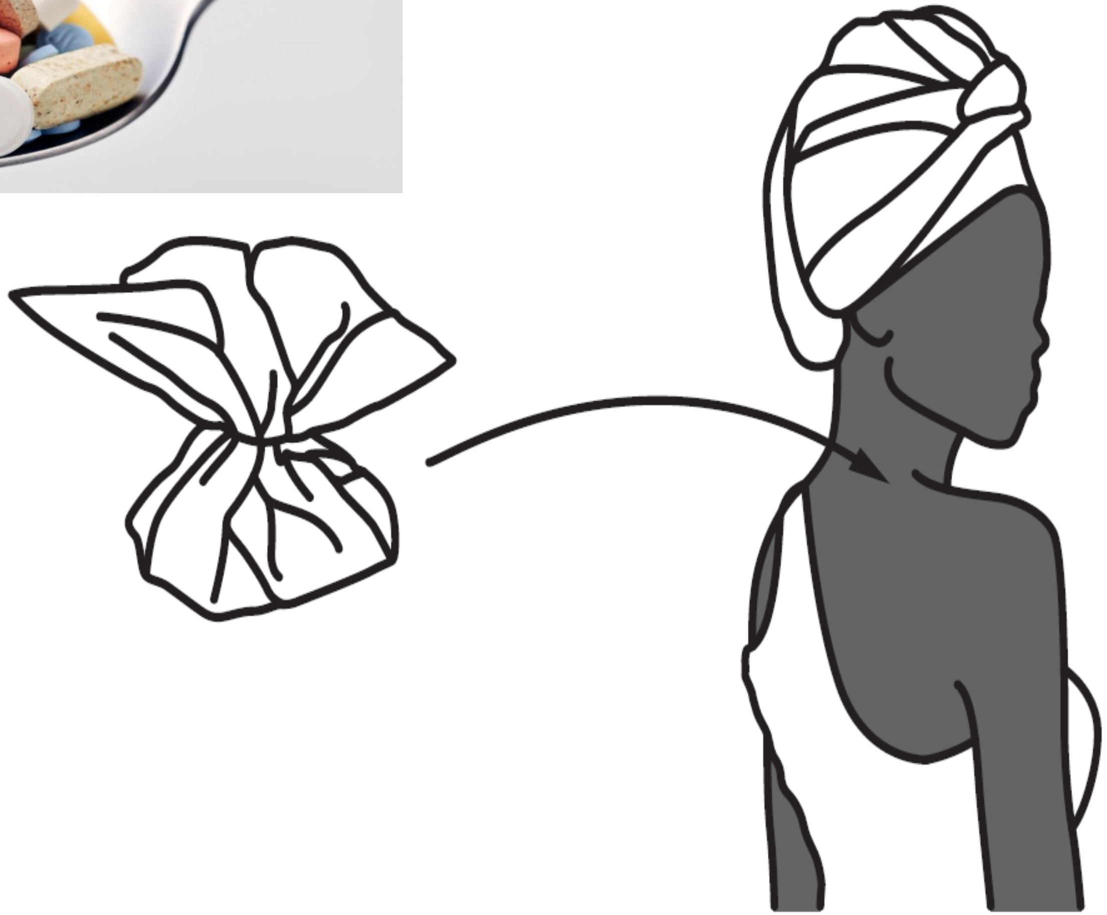

WHAT CONTRIBUTES TO OUR PAIN?

Activity

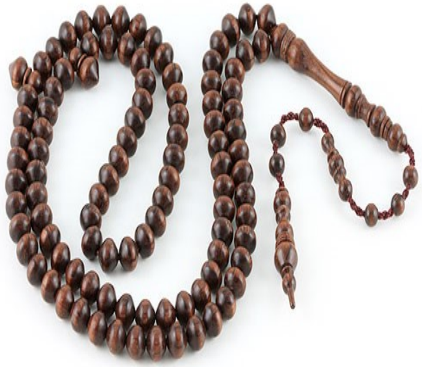

Damage

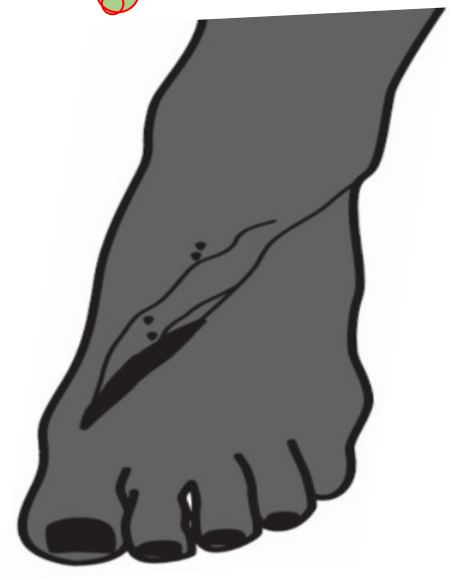

Religious

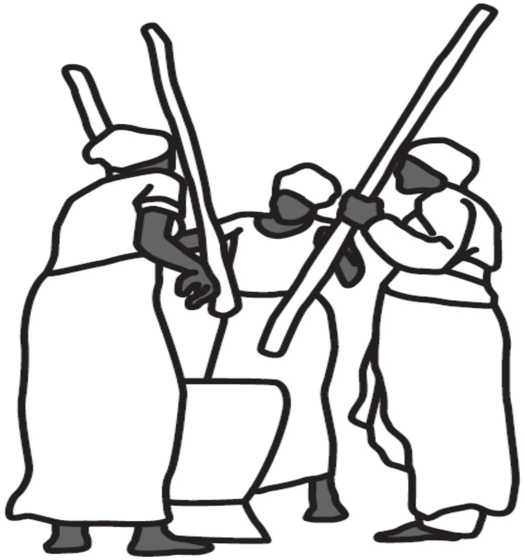

WHY HASANA'S PAIN IS NOT GOING AWAY?

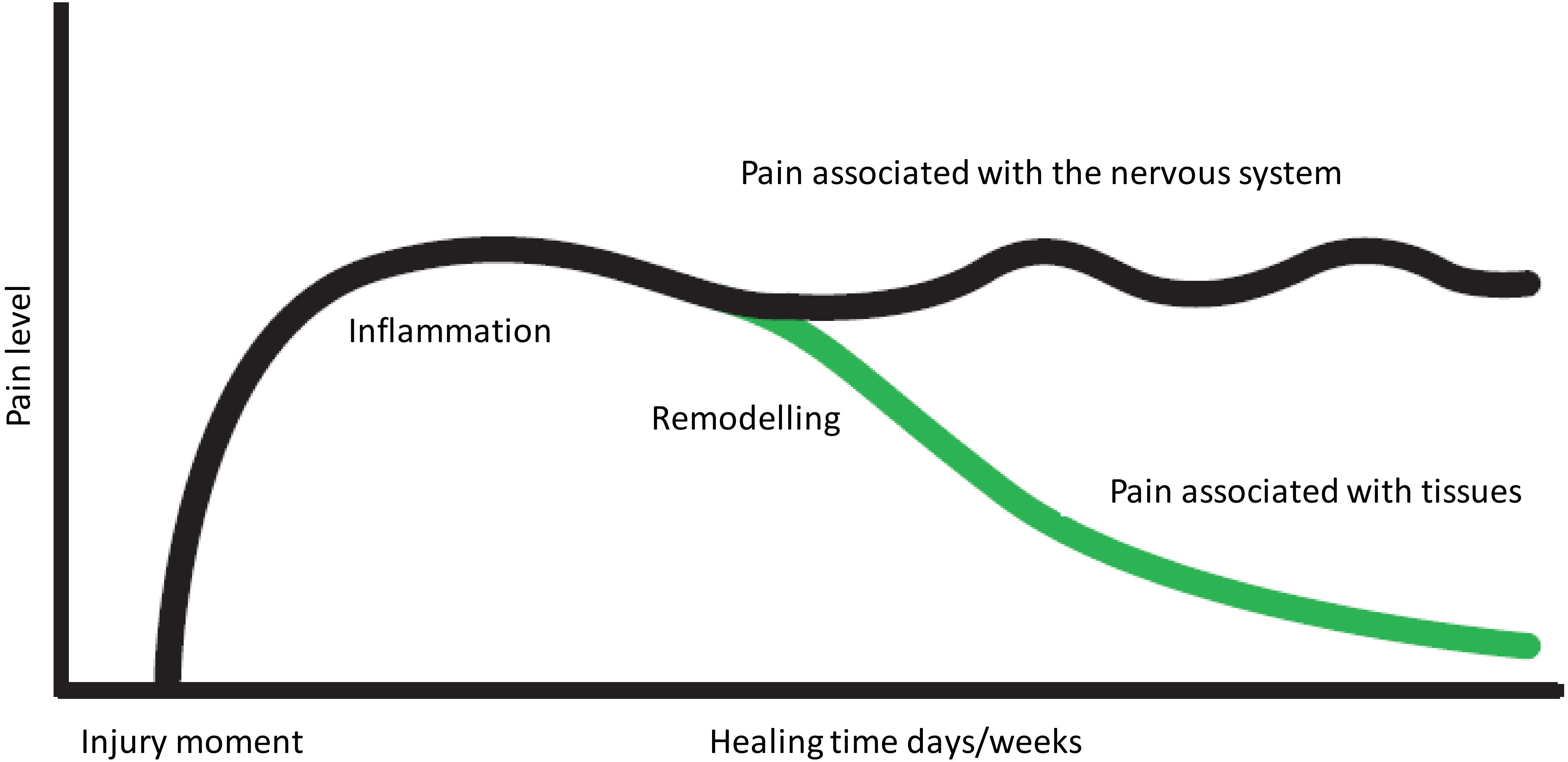

ADAPTATION AND FILTERS IN CHRONIC PAIN

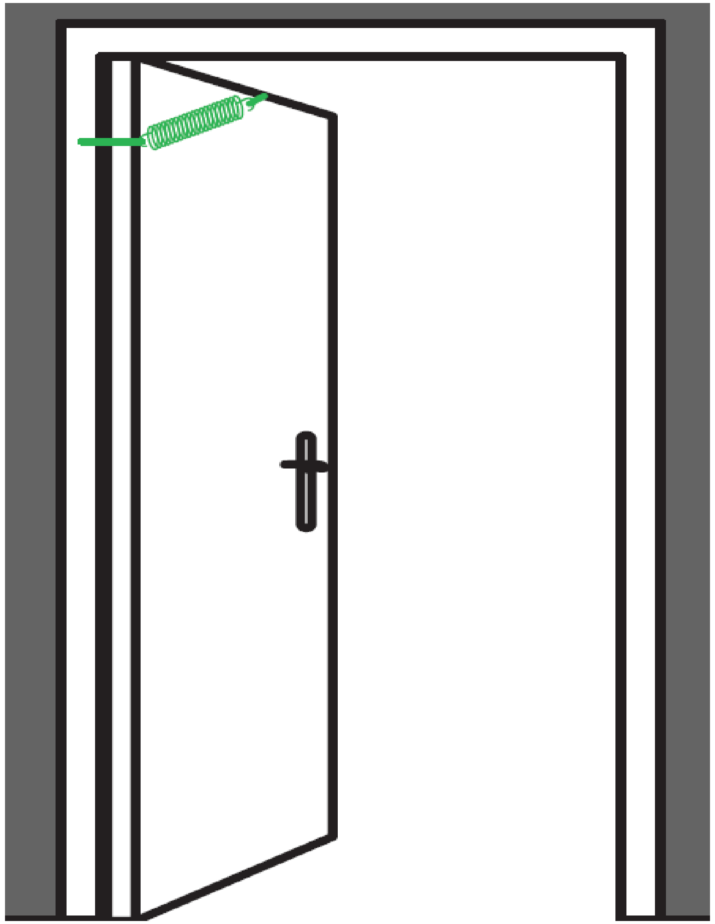

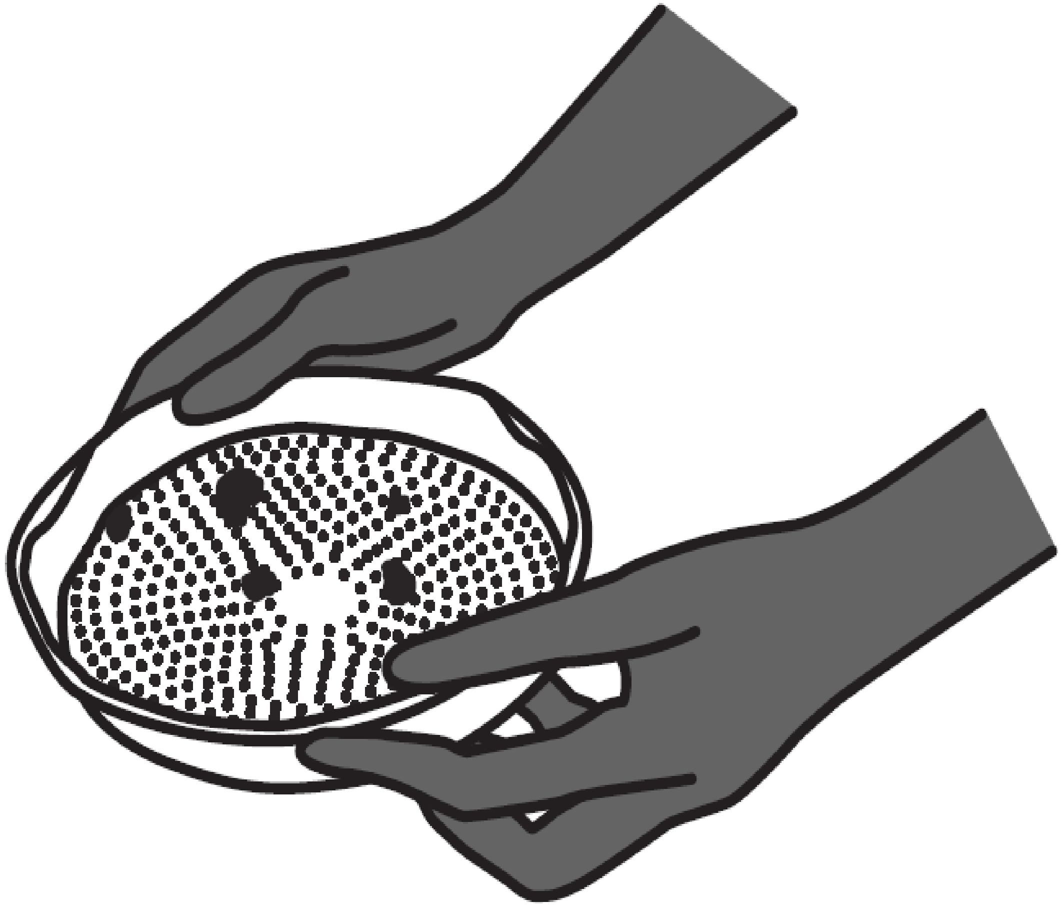

ADAPTATIONS IN CHRONIC PAIN

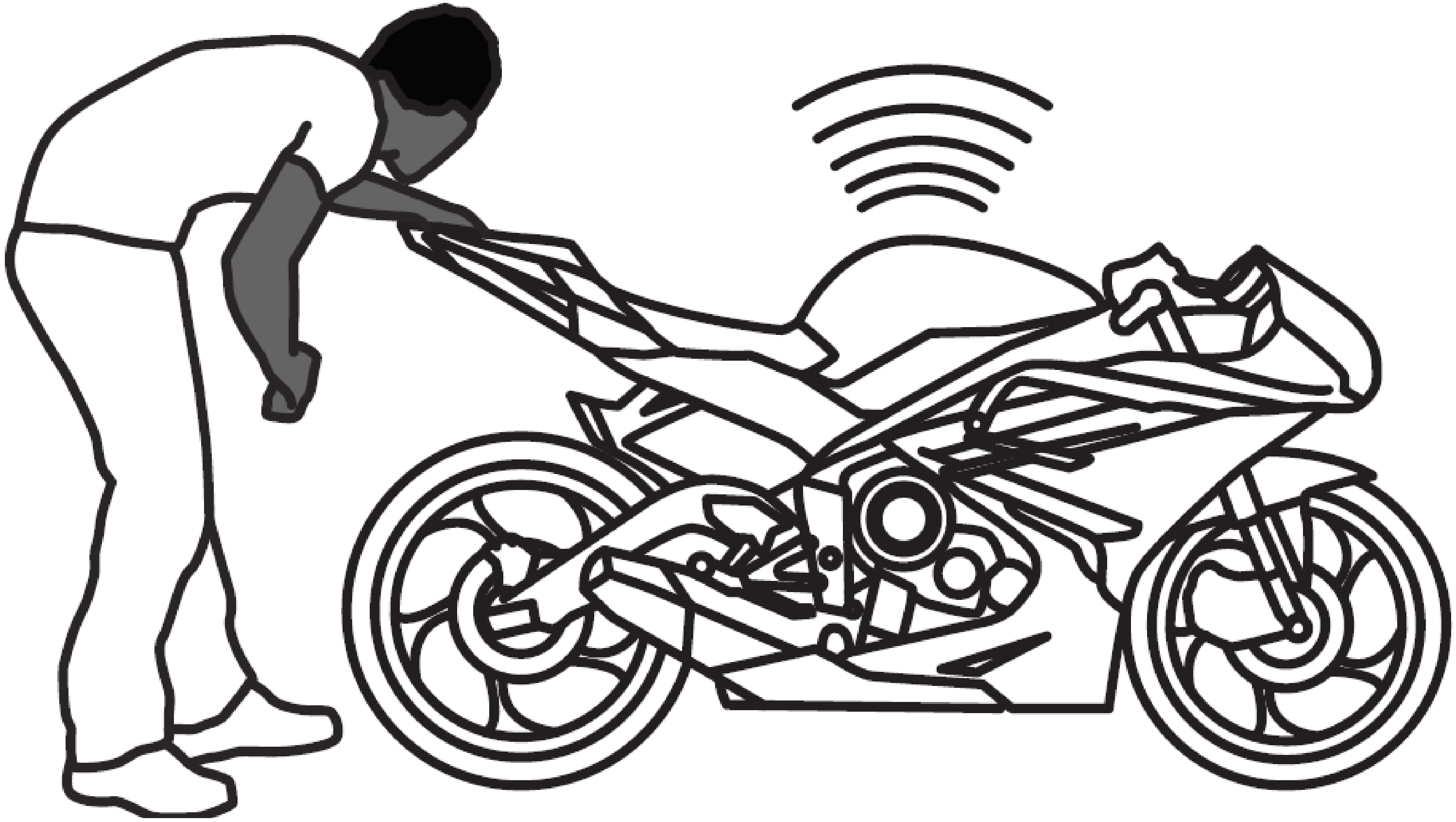

WHY DID IT HAPPEN TO HASANA?

Depends on

1. Amount of tissue damage
2. Genes
3. Coping behavior

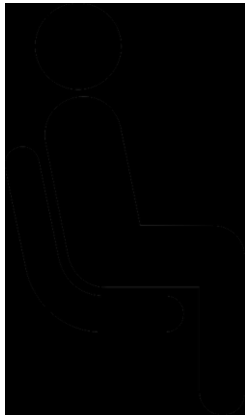

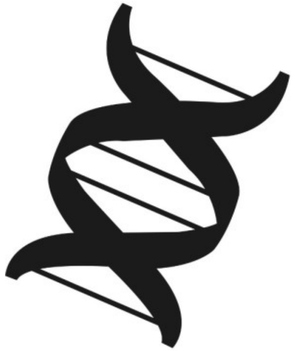

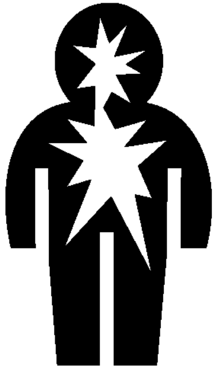

This is the only thing we can change

HOW DO I HANDLE MY PAIN

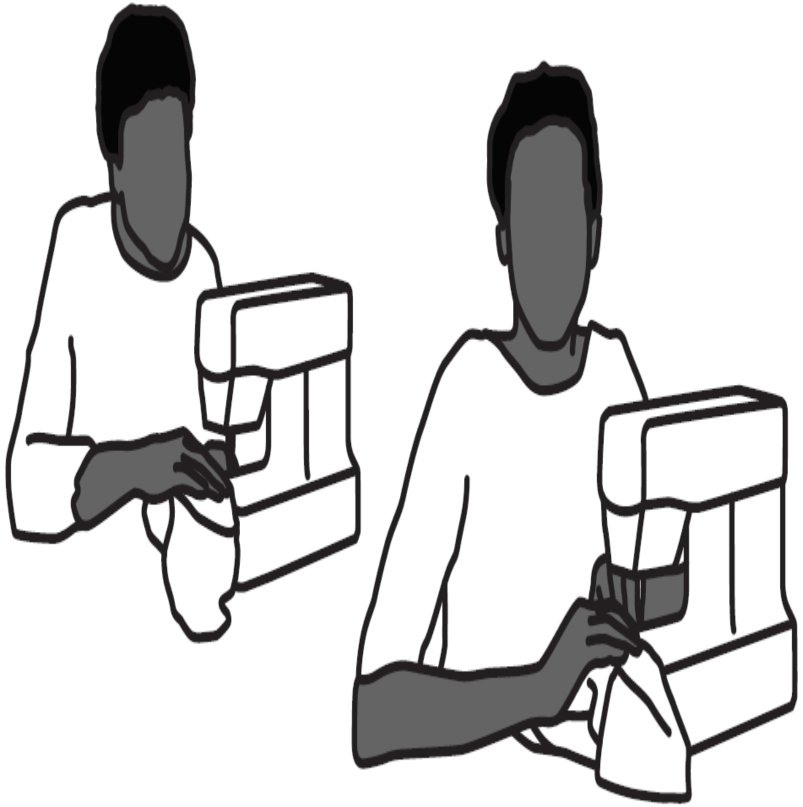

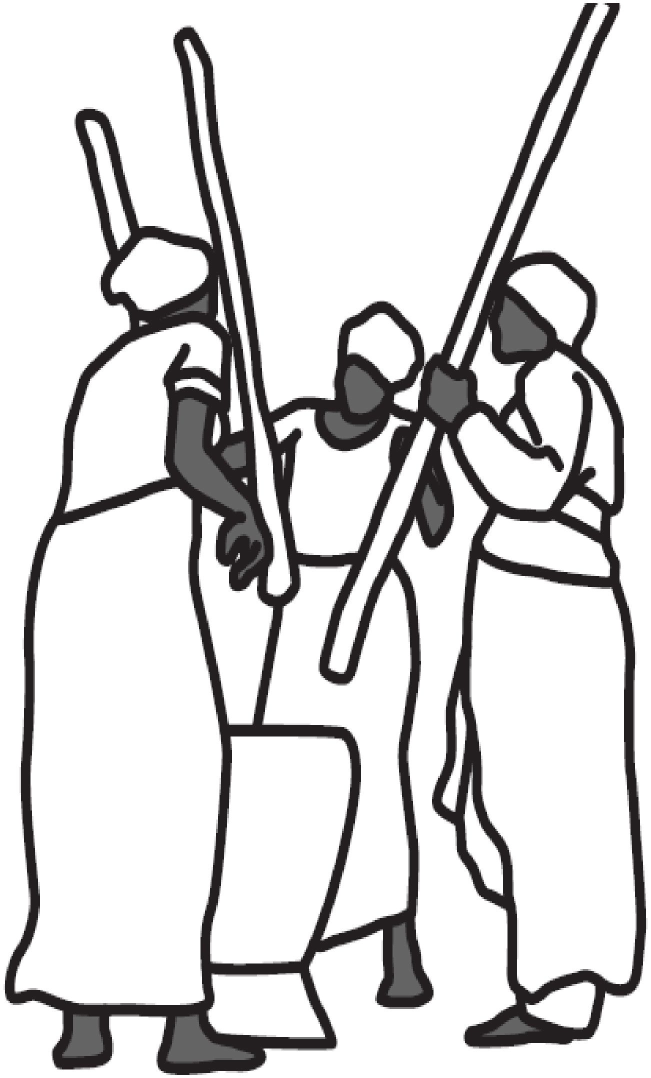

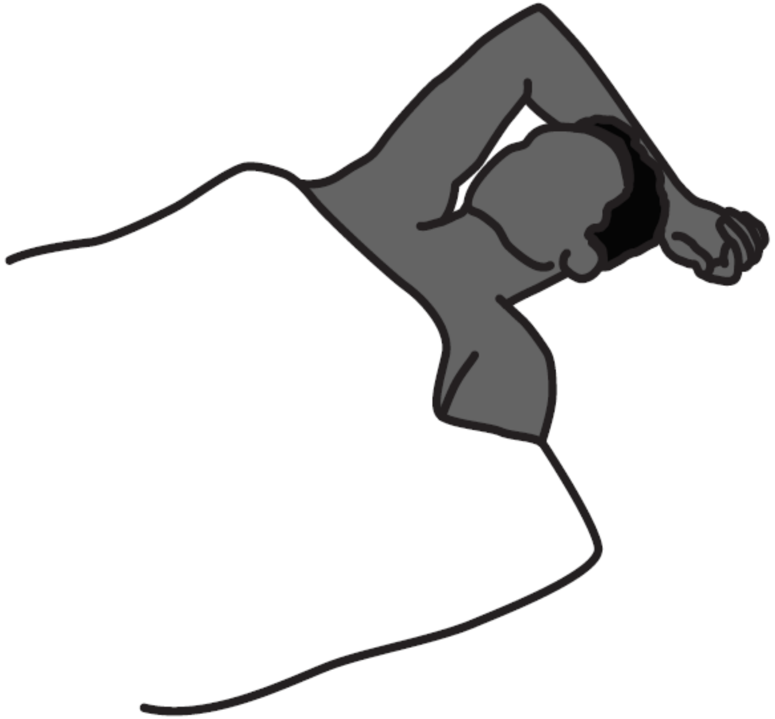

HOW DO I APPLY THIS IN DAILY LIFE?

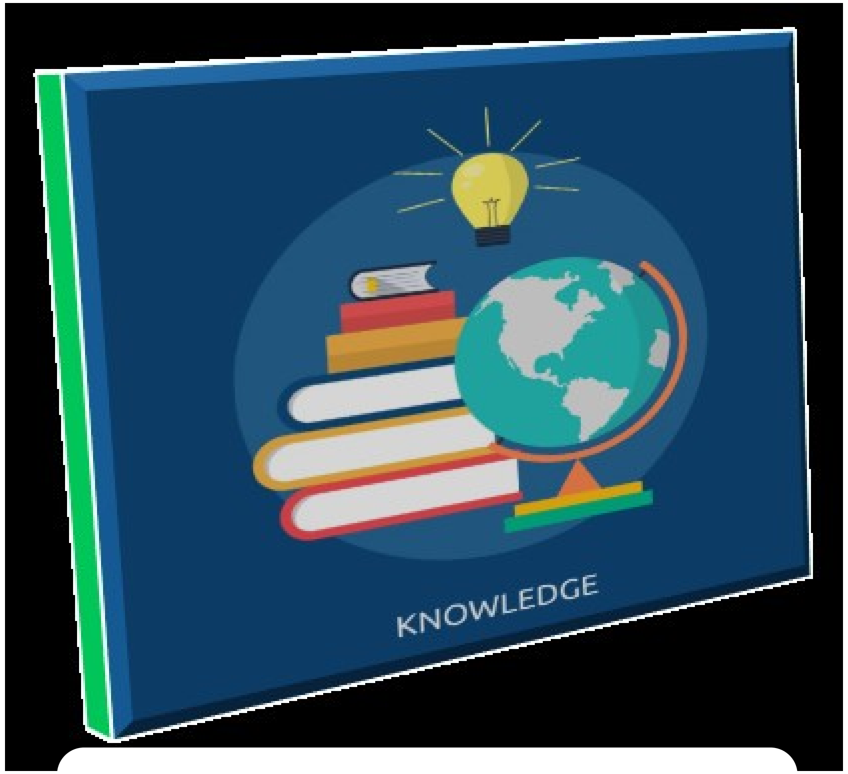

Get educated!

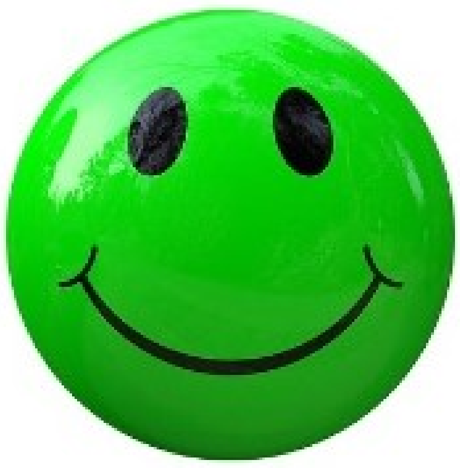

Worry less!

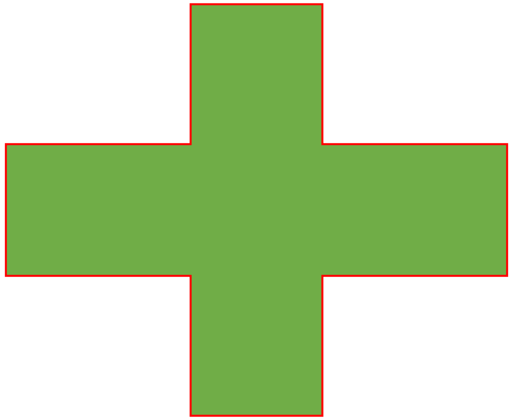

Be positive!

UPGRADE ACTIVITY LEVELS

Minutes of physical activity

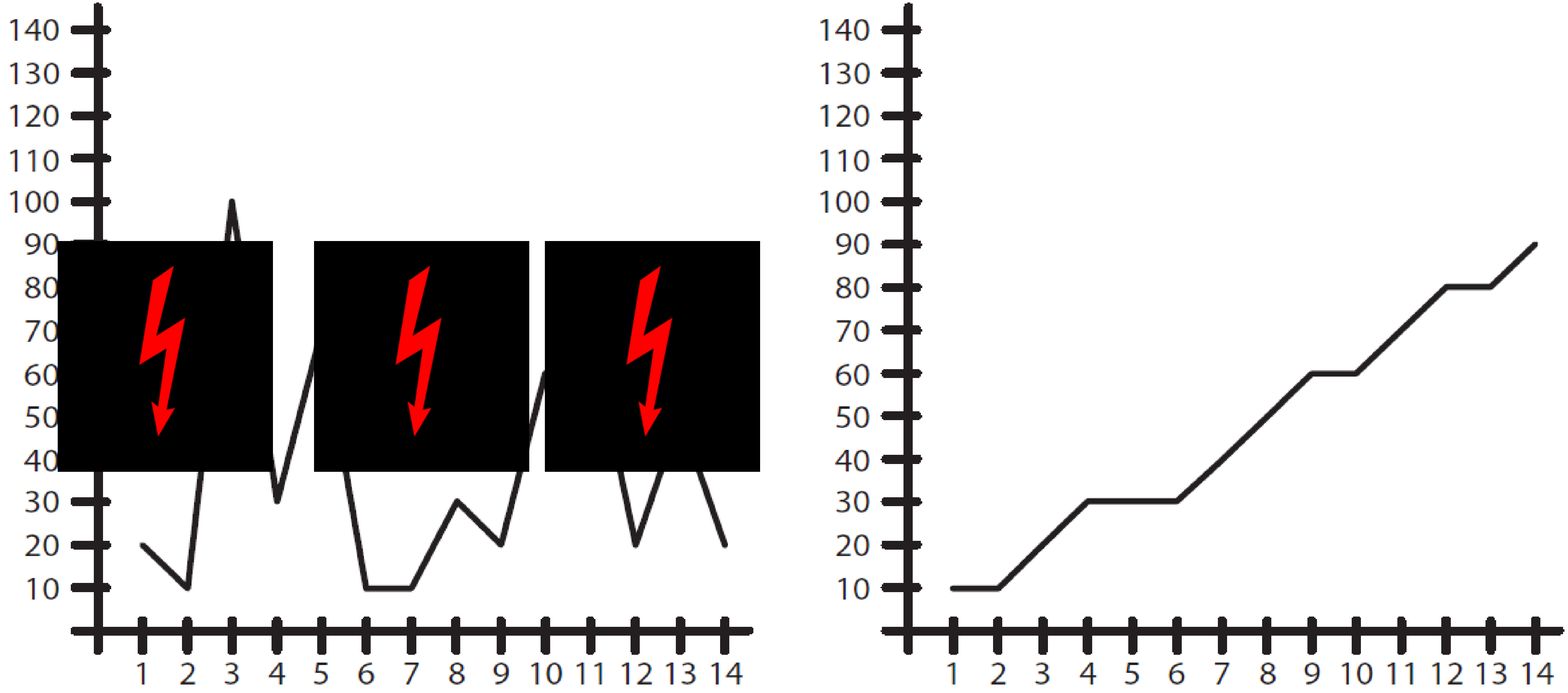

Days

HOME INFORMATION AUDIO

- Listen at the audio at home attentively
- Note down any question you may hav[illegible]
- Ask for clarification next session

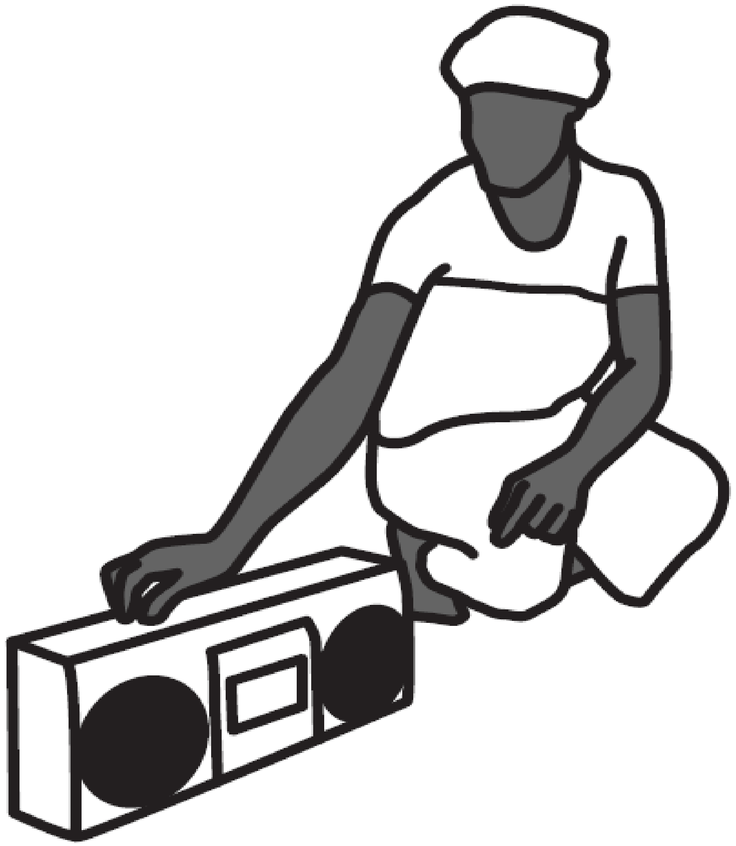

THANKS FOR PARTICIPATION

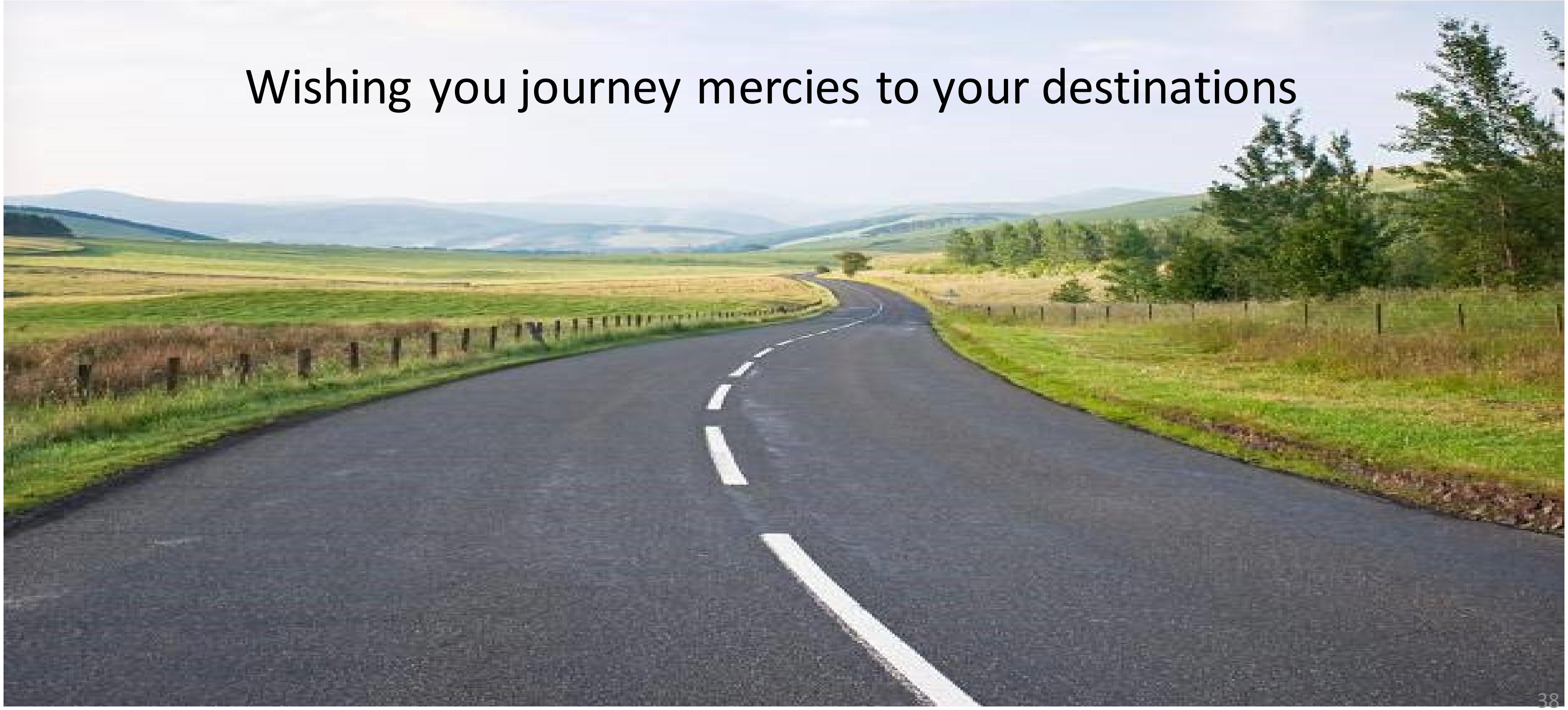

Supplement: S5 File — (PDF) [file pone.0253757.s005.pdf]
