# Supplementary material for: Development of culturally sensitive pain neuroscience education materials for Hausa-speaking patients with chronic spinal pain: A modified Delphi study
Source: PLoS One. 2021 Jul 2;16(7):e0253757. doi: 10.1371/journal.pone.0253757 (PMC8253446; doi:10.1371/journal.pone.0253757)
Supplement: S6 File — (PDF) [file pone.0253757.s006.pdf]

INFORMATION AUDIO FOR HAUSA PATIENTS WITH CHRONIC PAIN

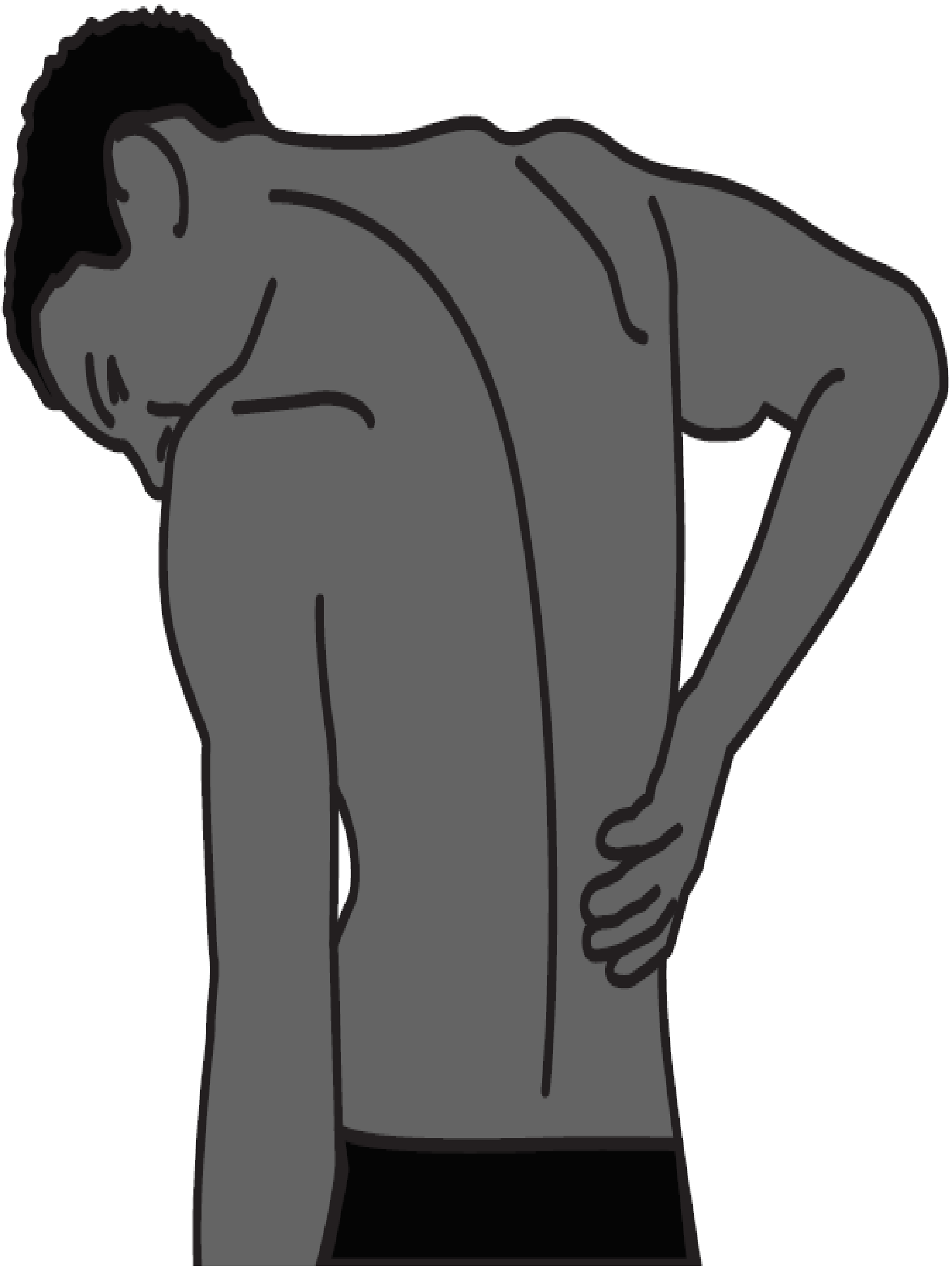

Assalamu alaikum, our respected listeners you are welcome to the second aspect of our interaction on pain, my name is Malam Ladan (ML), with me is your resource person in person of Dr Bala (DB), I wish you a happy listening

ML: Dr, you had a related previous session with the listeners, why the need for this additional audio? Is the previous session not enough?

DB: This is an important question. If you want someone to understand your lesson well, you need to give it in different forms for better understanding. The previous session was in the hospital and the listeners may not be very comfortable there, but now they are at home, they can listen at their own time and place of convenience.

ML: Who are your target audience/listeners?

DB: The primary target are the participants of the previous discussion, because they have attended the first session and therefore they may understand it better, but any other interested listener is welcome!

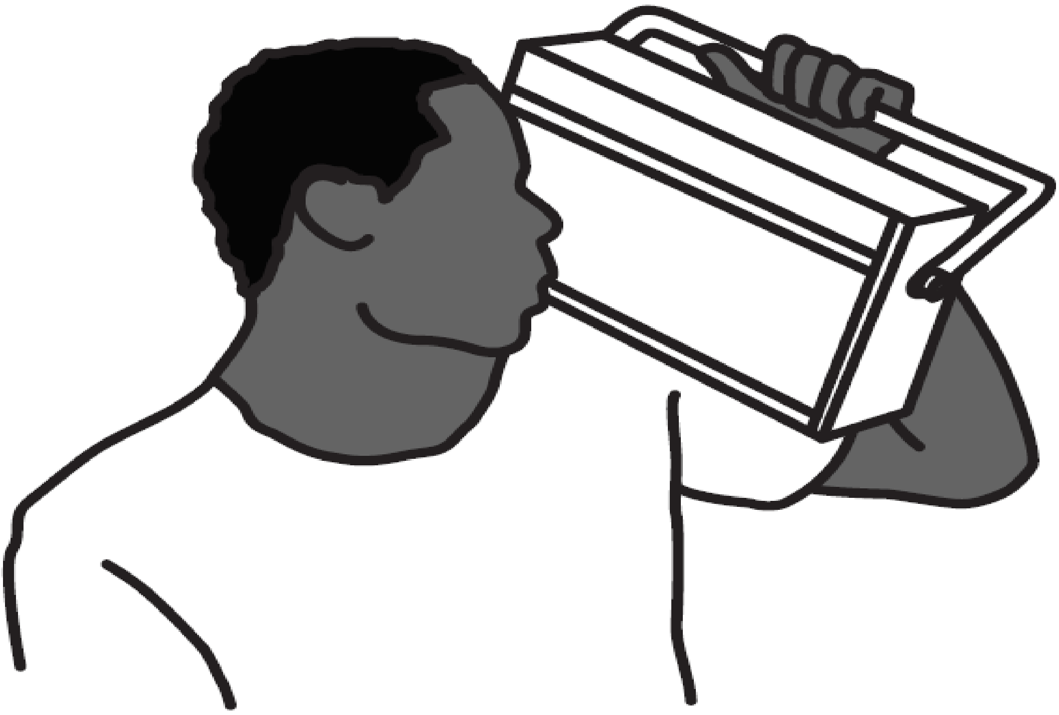

ML: Dr, you mentioned that the discussion is related to pain. Pain is a serious problem and I think we all want to live a life without pain?

DB: I totally understand, but it is good to know that pain is normal and even functional, but it does not mean you should live in constant pain.

What I mean is that pain is a vital part of our life. Everyone has pain experiences (young or old, male or female, rich or poor). However, it doesn't mean you should live in constant pain.

All pain experiences are the result of a process in the brain. After injury or when you are in a threatening situation, it is normal that you feel pain.

In case of damage or threat, pain alerts you to protect your body. Pain makes part of the healing process.

However, it is also possible that your brain decides that you are in danger and produces pain even if there is no problem or damage. In that case your brain is taking a foolish decision and the pain is not functional or protective anymore.

There are many myths, misunderstandings and also unnecessary fears about pain. We want to explain to you how your pain system works with this Audio. Understanding pain may help you to deal with it effectively.

ML: Since the discussion is on pain, why don't you start by explaining to us what is this pain about and why is it functional? I think it will be good to have that, right?

DB: Yes you are right! Pain is a natural way of protecting our body, it is very important in our life. Everyone feels pain. We would not survive without experiencing pain. Pain leads to safe behavior and taking care of problems in our body. I give you some examples:

Pain helped **Abdullahi** to protect his hand from a very hot charcoal iron he is using to iron clothes to earn for living.

Additionally, **Abdullahi** as a carpenter, was able to protect his finger while hammering a nail because of pain.

Even more importantly, **Abdullahi** and his family escaped from severe injuries or even death due to the benefit of pain. (Did I say benefit of pain? Yes! Benefit of pain). Their room was about collapsing, the blocks of his room started falling on them and the pain wakes them from the sleep and finally realized the danger of their building collapsing. Although they may not notice pain while escaping because their body has suppressed their pain for the purpose of escape, but the initial pain they felt helped them to realize they were in danger. Thanks to pain!

If not because of pain, venomous snakes would have killed many people unknowingly but thanks to pain, people will rush for medical attention after snake bites.

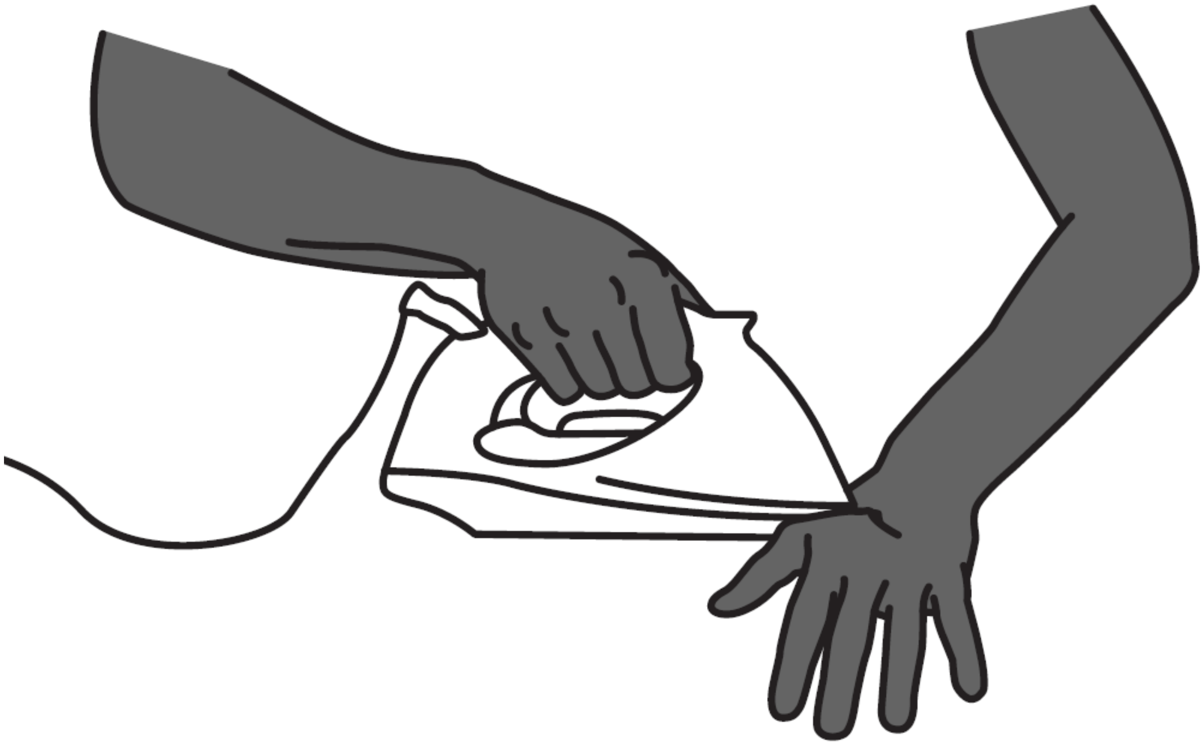

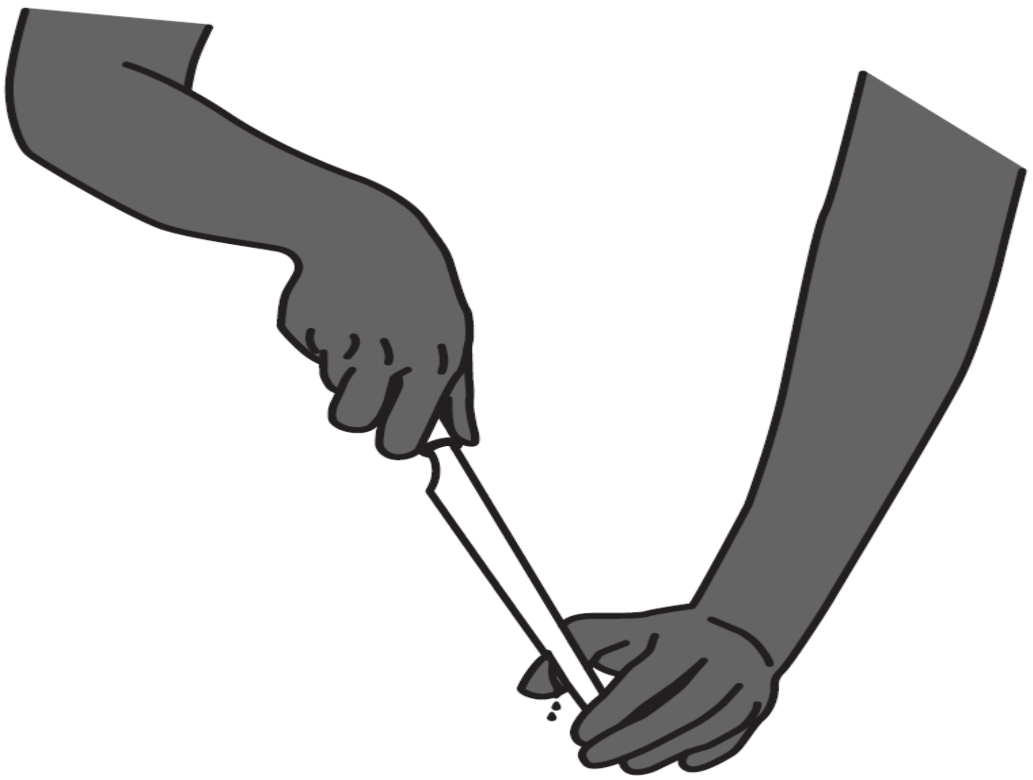

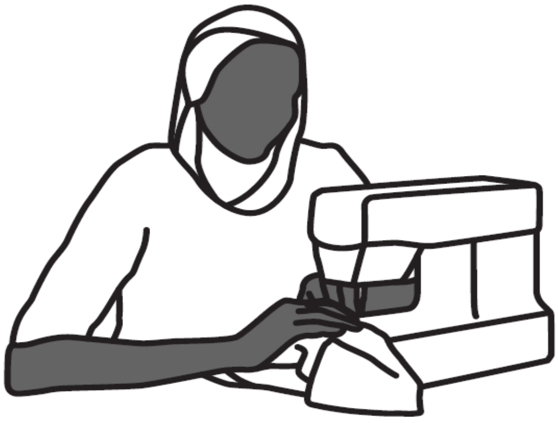

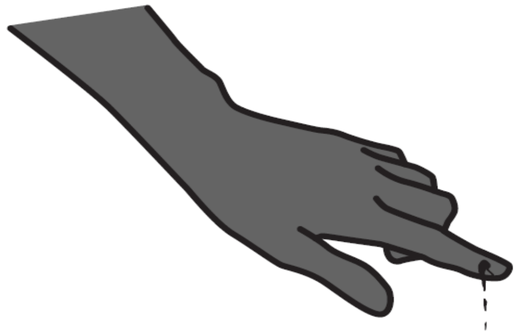

ML: Dr, if I get you right, this pain is like an alarm, is it?

DB: Certainly! Pain is an alarm that lets you know about a danger! Alarms help us to wake up. Dogs’ barks tell us about a possible intruder, likewise pain tells us about a potential danger.

If we don’t feel any pain, we never learn to avoid dangerous things.

Our listeners can remember **Abdullahi** who is diabetic and sustained a wound at his foot sole unconsciously because he could not feel the pain because of sensory loss at his feet due to the diabetes and in the end he lost his foot to amputation. He wished he could have felt pain at his feet.

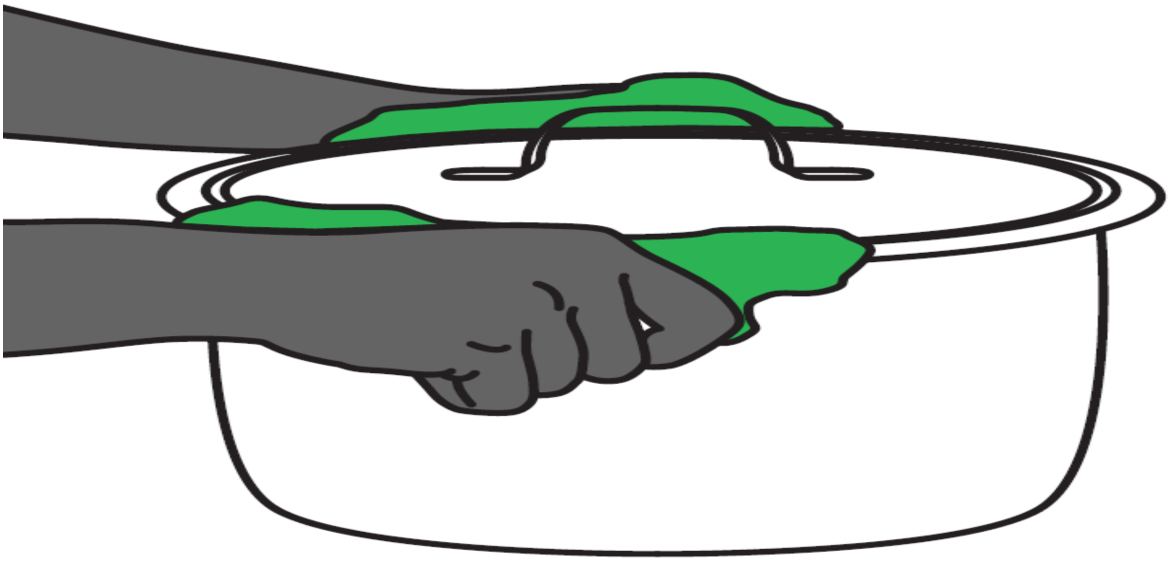

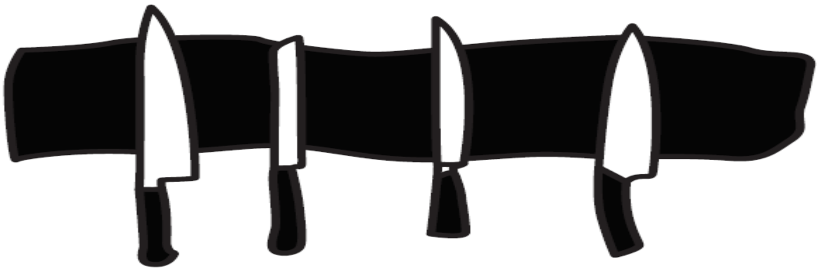

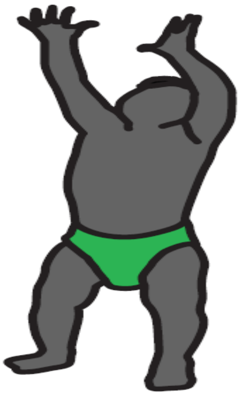

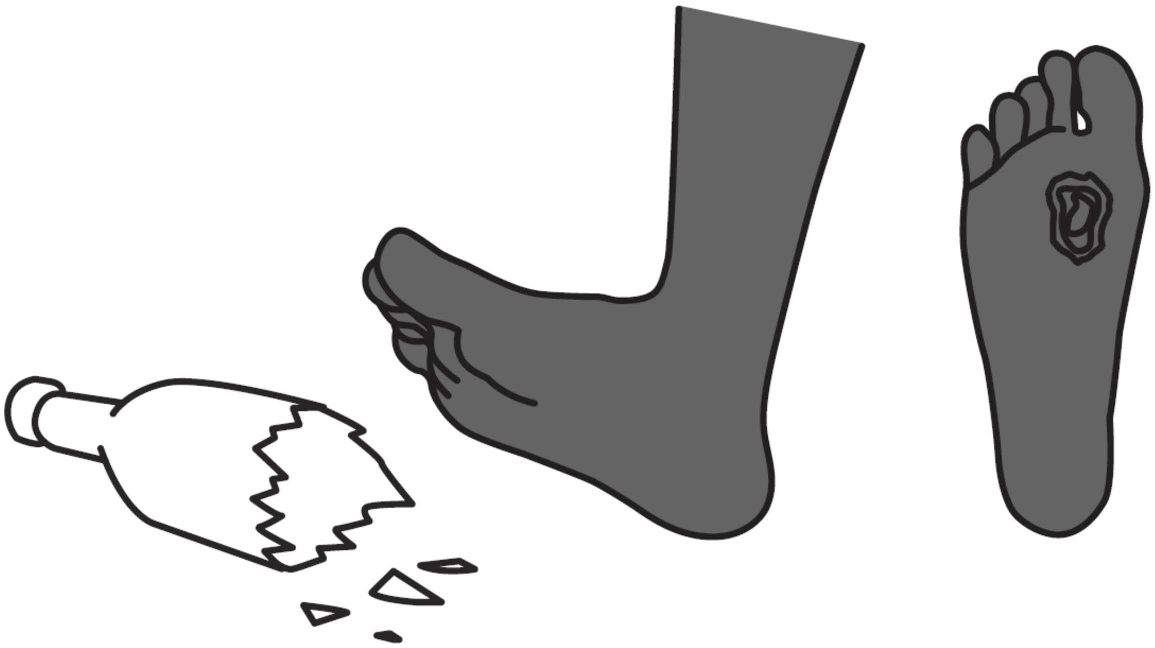

ML: But Dr, I hope I did not cut you short, some of us may ask: ok pain helps us to know if there is danger, why won't the pain go immediately after the injury?

DB: A very interesting question indeed! **After injury, it is normal that you feel pain.** Pain makes part of the healing process. After **Rakiya** injured her finger, the nerves in her fingers will become more sensitive for a while. Her finger will become more sensitive, leading more easily to pain. Pain will remind her to be extra careful with her finger so that she will not be reinjuring her finger and thereby delaying the healing.

After an injury, temporary damage causes temporary pain. It is normal and necessary to let us recover and survive. This short-duration pain of **Abdullahi** and **Rakiya** and any pain similar is called acute (new onset) pain. We can easily say what is the cause of our acute pain and conventionally such pains don't last longer than necessary for the healing.

Acute pain is usually recent and of a known cause, like the **Rakiya's** knife cut, **Abdullahi's** hammer injury and headache due to malarial fever, etc

ML: So in brief, what is an acute pain?

DB: Acute pain is the one of which we most of the time easily know the cause and it usually lasts a shorter period of not more than 3 months

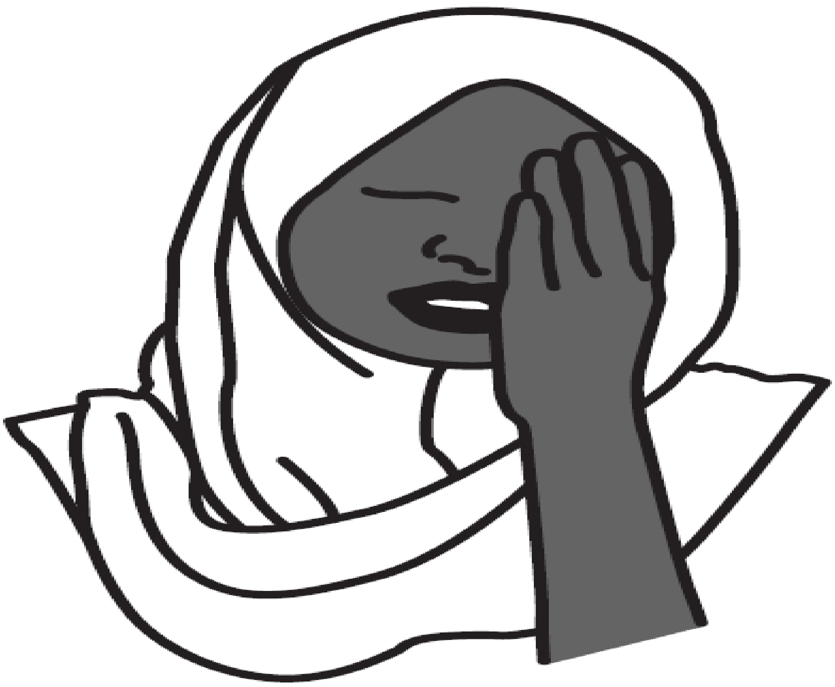

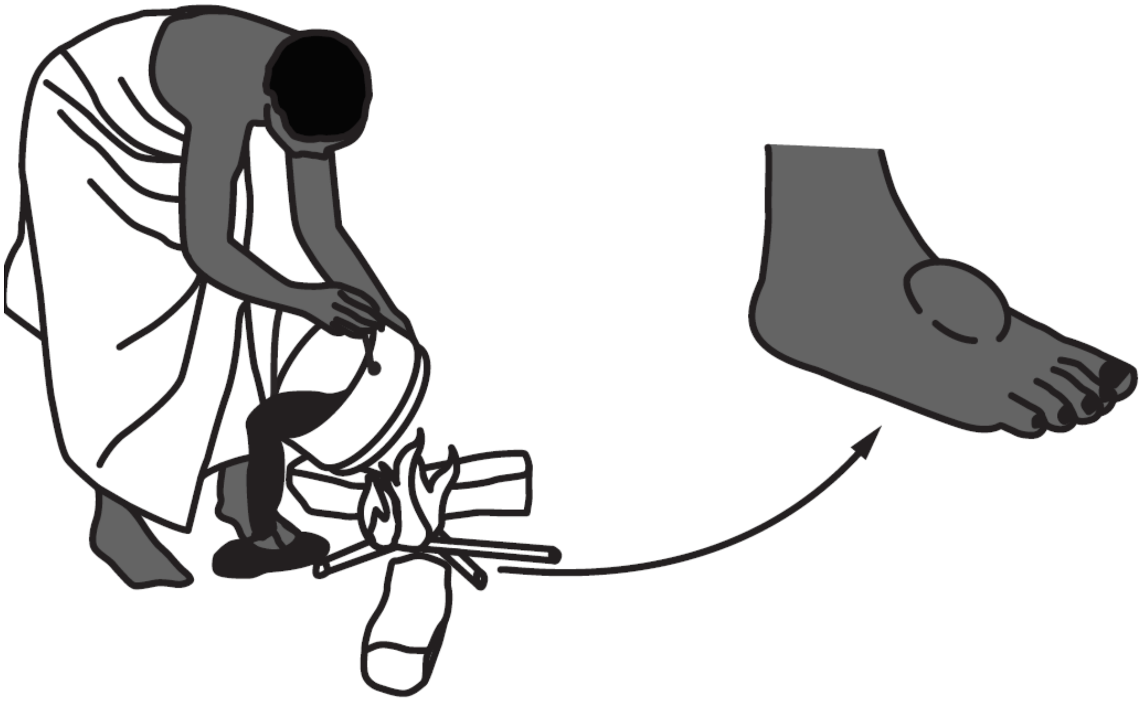

ML: Dr, is it like this our body has a special way or a system of handling pain?

DB: God is wonderful Malam Ladan! Our body has many systems with different roles. When we get food, we chew it in the mouth and then swallow it through the oesophagus and it gets to our stomach where some are filtered out and finally excreted through the anus or the useful ones absorbed in the intestine for our benefit, this is a digestive system.
Pain has also a peculiar system in which information is gathered, filtered, processed and actions are taken (where necessary). Based upon the information the brain decides when we are in danger, so we may call it our "Danger alarm system".

Sensors, nerves, spinal cord and brain are involved in the Danger alarm system.

The Danger alarm system has a network of nerves, like electric wires, covering all parts of the body to gather information everywhere. The nerves' ending have sensors (for mechanic stimuli, temperature/heat and chemicals), they can detect sources of our acute pain, from a pressure/compression/rupture as in knife cutting **Rakiya's** finger, or from temperature as in the boiling water pouring on **Rakiya's** foot or chemicals as the case of **Abdullahi's** acid injury to the hand in the tanning factory.

These sensors/receptors sit at the end of the nerve cell and any information detected is taken and then transported to the spinal cord through the nerves. They are very sensitive like a magnet in the sand that can attract tiny metallic objects so they can easily detect any potentially painful stimulus.

All the nerves in the body are connected to the spinal cord, and the spinal cord to the brain, where information is filtered and processed. The brain is the command centre of the Danger alarm system.

ML: Won't you say something a little more about this spinal cord Dr?

DB: Our spinal cord is a jelly like structure that extends from our brain (through the bottom of our skull) down to the end of our back through the back bones. The spinal cord serves like a gate-man in the houses of the rich or traditional rulers, it receives any messenger first and analyze the message brought by the messenger and decide whether the message should pass through to his boss or maybe it's not something big and his response can suffice or the message is not even worth handling and he will ignore the message, this is the same when a danger message comes, if it is strong enough the spinal cord passes it up to the brain while the weaker messages are handled by the spinal cord.

ML: Dr. sorry for that interruption, but I guess listeners will appreciate the explanation on the spinal cord and now you can continue on your explanations on the system our body has for handling dangers.

DB: ML never mind it was a very helpful interruption. As I was saying we have the danger alarm system medically called 'nervous system' which resembles our complicated electricity wiring. Looking at the wires from the outer part, we may get confused asking ourselves which wire serves which role and which wire goes to which house or comes from which house?

Because the wires may look similar and some are even twisted or overlapped, but to our surprise all the houses have electricity and things are working well, this means each wire is working as expected. That's how our nerves are connected to the spinal cord and brain although they may look complicated.

When **Rakiya** injured her finger with the sewing machine nail, sensors in her skin are triggered and gates will open (under normal circumstances the gates close back easily) with this stimulus. This creates an electric signal (that's why the speed of the whole process is very surprising!) in the nerve, and a danger message is sent to the spinal cord. Signals are filtered in the spinal cord. If the signals are strong enough to pass this filter, danger messages travel to the brain. In the brain, there is another relay centre that filters the signal and decides which signals will be finally analysed in the brain.

The danger message is processed throughout different brain regions. There is not only one pain centre in the brain. Many parts of the brain are involved simultaneously in analysing the danger signal. Just like the team work in community development works *'aikin gayya'* in which different people contribute to the success of the project, so also pain analysis.

An example of how brain works maybe like how the Traditional ruling council works, the Emir is the highest decision maker while channels are used in handling information, when there is an issue with the masses, the ward head is the first point of contact, some issues that are very simple are treated there and forgotten while some bigger/complex issues may be passed on from the ward head to the district head and from there if they are considered big enough they are forwarded to the emirate and different title holders will help the Emir in taking decision and the channel used in bringing the information to the Emir will be used in relaying back the feedback. So we can see many filters and how decisions are made.

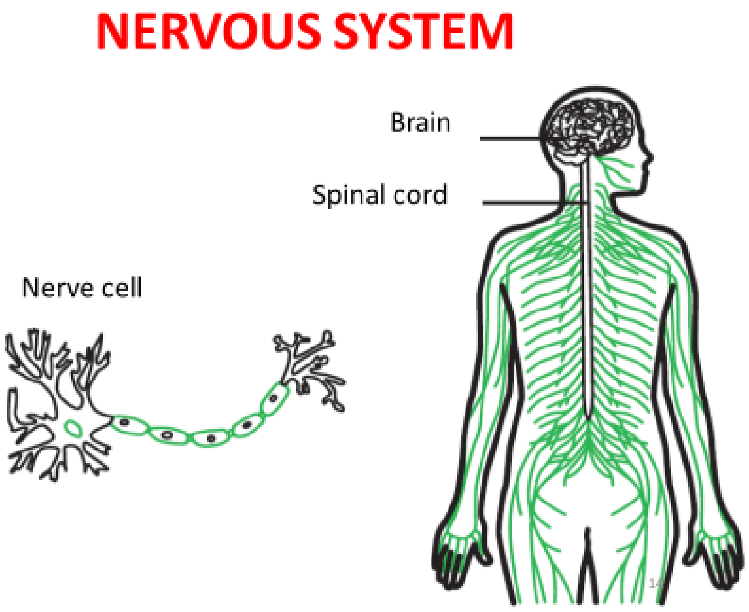

RECEPTORS ON NERVES

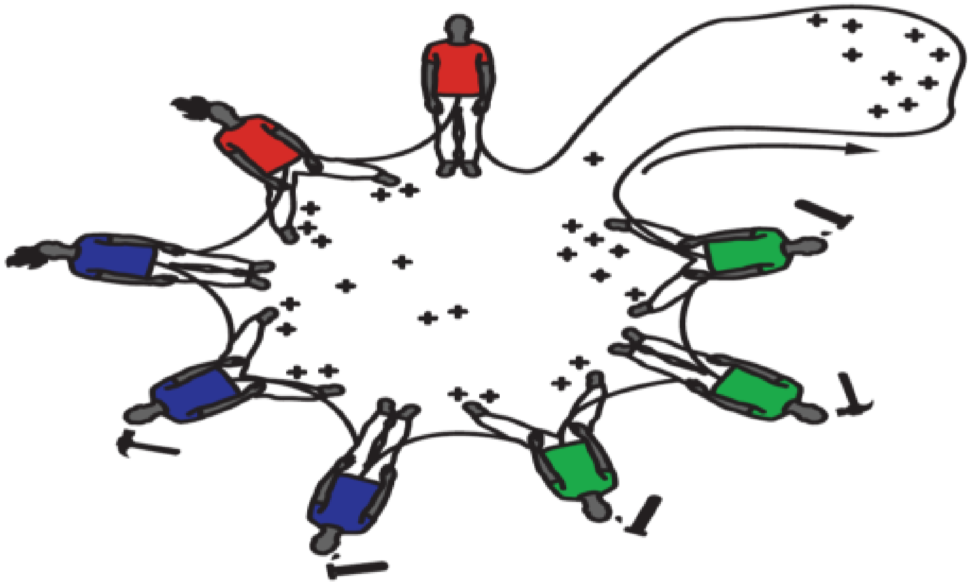

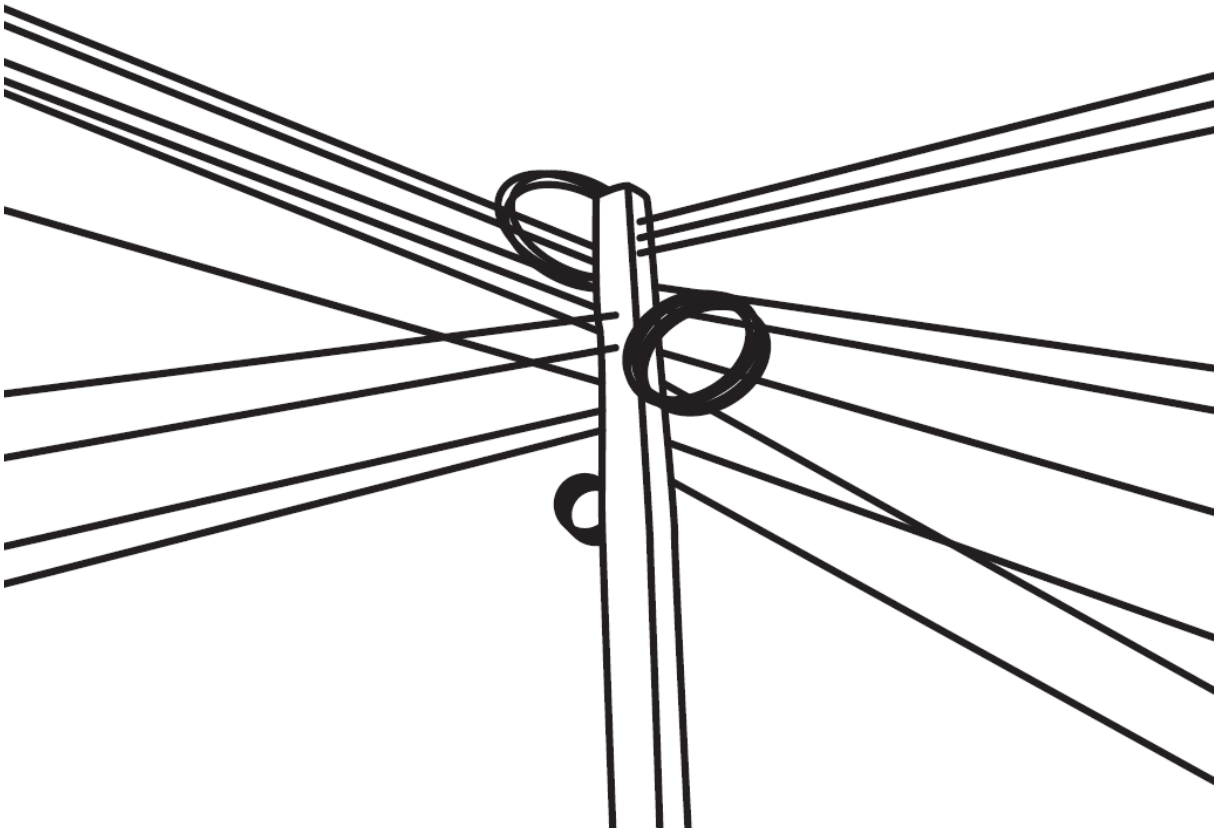

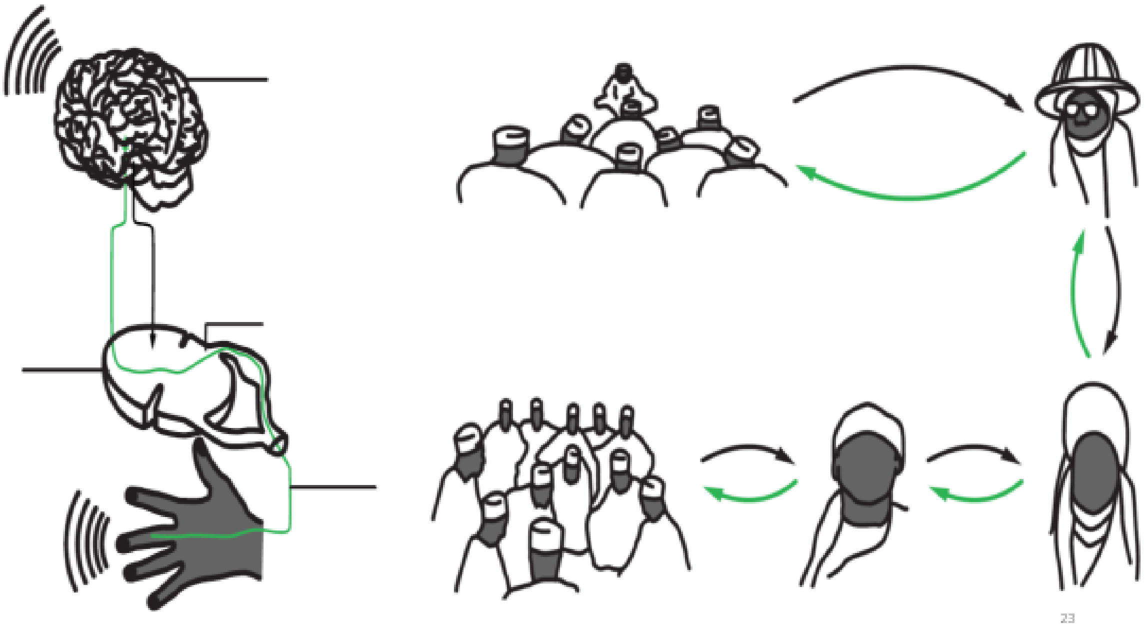

ML: What does the brain do after getting the information?

DB: When the brain gets the information, it responds in two ways, it can either suppress or facilitate the pain

ML: How?

DB: The brain can release happy hormones/body agents responsible for suppressing bad signals sent from the brain. They prevent to send further danger messages. This is our very powerful pain control system. It is 60 times more powerful than any pain medication. For example: warriors/hunters who have a severe injuries, e.g. leg amputees, sometimes report no or little pain. People that do *'Shadi/sharo'* are seriously beaten and even severely injured but they report little or no pain (they even celebrate afterwards). This is related to our internal pain control system.

Severely burnt mothers run back into burning houses and run through the fire to save their children. They feel no or little pain.

On the other hand, brain may release substances reinforcing the incoming danger messages. Due to this system a small injury can hurt a lot.

Rakiya's abdominal pain gets worse whenever her school mathematics teacher is in the class holding his usual cane. Some individuals get headache/heart pain when terrible accidents happen before their eyes. Even when **Rakiya** sees the injection needle for the vaccination of her son, she gets pain.
Although they experience no physical injuries themselves, the brain decided to produce pain.

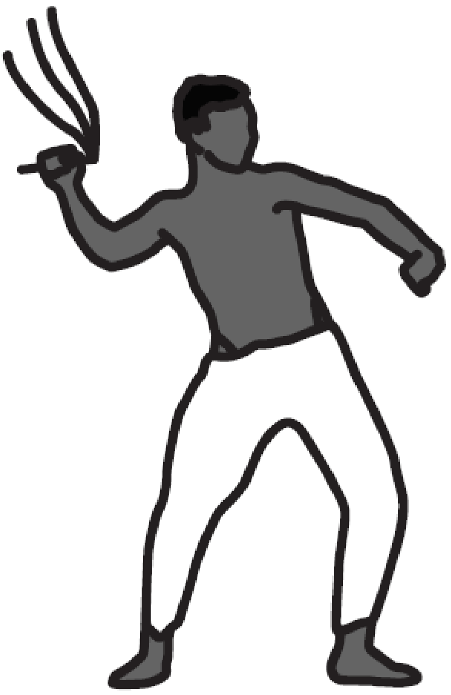

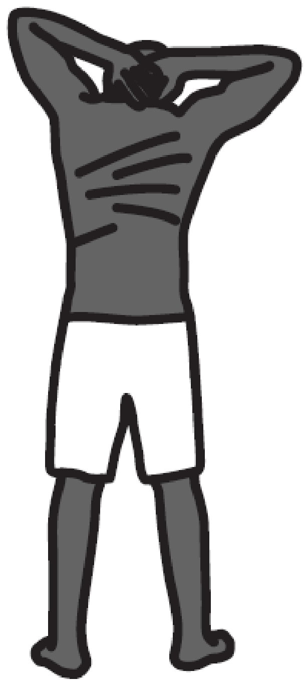

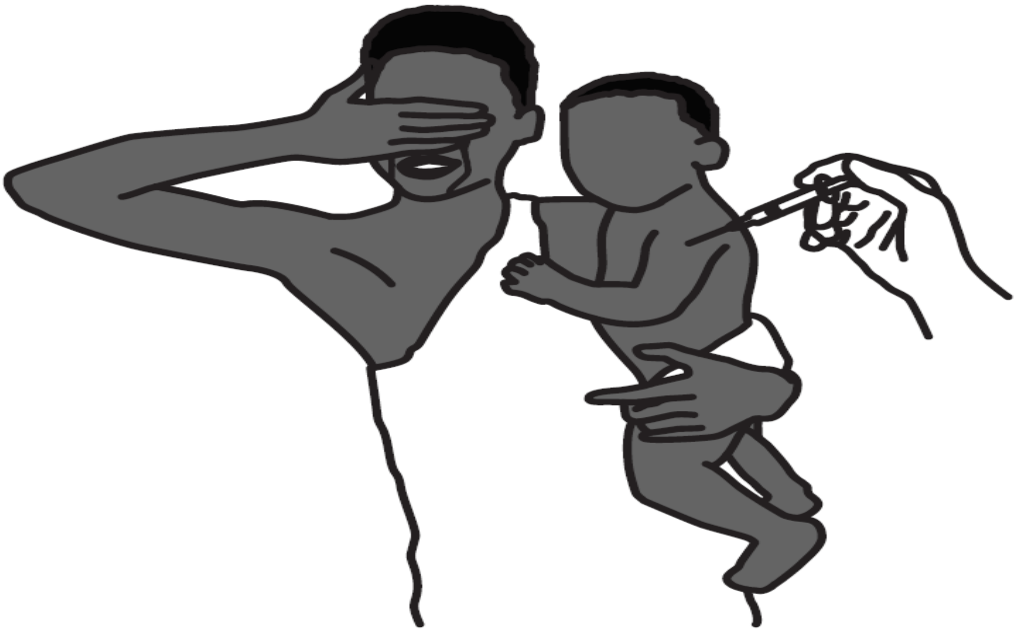

ML: So which lesson do you want to pass to the listeners?

DB: As I explained and we have seen, pain severity is not always related to the amount of tissue damage.

Small injuries can cause a lot of pain and severe injuries might not hurt. Understanding and appreciating this will be very important in handling our pains.

ML: Dr, you previously explained acute pain to us, does it mean we have another type of pain? Maybe non acute? if I maybe right, sometimes the pain persists for a long time after the injury?

DB: You are right! But instead of calling it non acute, we use the term chronic pain. **Sometimes, the pain system loses its control**

For example, there are situations in which the security sensor of a motorbike stops working normally and it makes too fast or too much sound even without anybody approaching it or just a mosquito flying by. This is similar with chronic pain: the brain produces pain and thus with little or no danger stimulus, pain is felt.

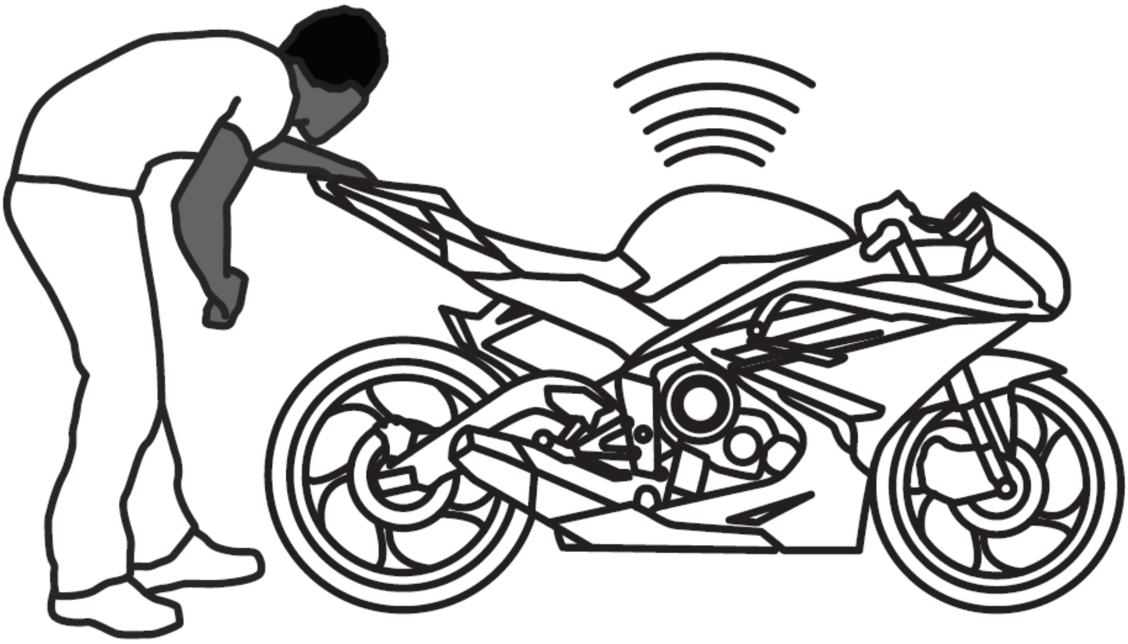

When pain persists for more than 3 months it is regarded as a chronic pain, it is estimated that, in every 5 individuals, 1 is having such a pain. Although the body ensures recovery after injury, pain may sometimes persist for longer periods even in the absence of a clear cause. Many individuals have persistent pain in the back without an understanding of the cause. **Rakiya** and **Abdullahi** have persistent back pains that stop them from doing activities. They have tried all the available alternatives of treatment but it doesn't work, why?

Sometimes instead of the pain sensors to stop sending information to the brain, they become more sensitive and even send more information. The gate in the spinal cord will open longer than necessary. Let me give some more examples here; when the spring of our door becomes loose, a little push will open the door and instead of the spring to close back the door after the push, the door will remain open for a longer period of time due to the weakness of the spring, this is similar to what happen to the gate in the spinal cord during chronic pain situations, stimuli that are not even strong enough will open the gate in the spinal cord and the gate will remain opened for a longer period thereby making more messages to enter the spinal cord.

When the messages enter the spinal cord, we still have a filter that determines which messages are sent to the brain or not, in chronic pain this filter may also be less effective and sensitive due to too much messages coming through the already loose gate and as such messages that are not supposed to go to the brain are allowed to go, this is similar to our sieve in the kitchen which due to overuse may have some bigger holes and therefore particles that are not supposed to pass through can now pass through. In the brain there is another filter and can also face similar problem in chronic pain situation and consequently brain receives too much messages and therefore may take wrong decisions by defining normal or less threatening messages as painful.

An example where wrong decisions maybe taken due to repetitive messages, just as the brain does in chronic pain situation is like in the traditional council in the Hausa land where a specific district maybe associated with too much complaints coming from the district head to the Emir and in some cases no thorough investigations will be taken by the Emirate council because they are tired with such kind of complaints and an action will be taken which may be wrong due to lack of investigation and wrong assumptions.

This is how our brain works in chronic pains. But the good news is that, just like we can repair the spring of our doors or our kitchen sieve to work well, so also our pain system can be calmed down and it can revert to a normal work.

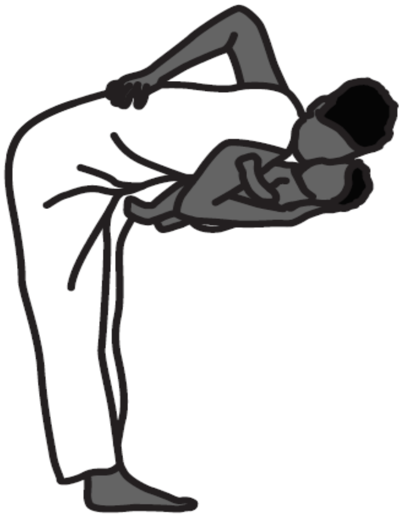

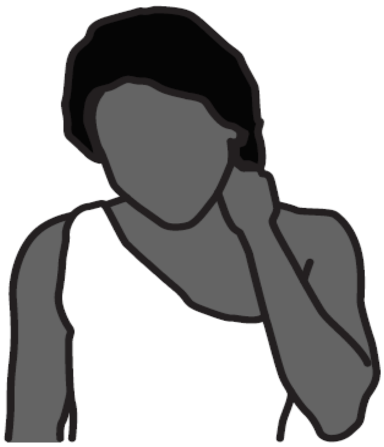

ML: But Dr, don't our worries contribute to our pains? Let me confess here, I also have a persistent pain but it gets worse when I am worried, what can you say?

DB: Malam Ladan, it is like I have to bill you for consultation (laughs). Other factors can also distort our pain system and consequently contribute to our chronic pains, these include our thoughts, beliefs, fears, worries, stress etc.

Abdullahi is the type that when he is worried he gets pain easily, little touch from his kid is painful, everybody knows this in the family and when he is in pain and at the same time worried his wife will usually calm him down and his pain gets better. Since **Rakiya** got divorced from her husband her abdominal pain and headache get worse, she personally knows that if she will settle with her husband her pains will be better.

But another good news is that, we can control these factors and as well our pain perception will be better and in that case we can calm down the hypersensitive danger alarm system ourselves

ML: In that case how do we calm down our pain system?

DB: Getting educated about pain is the key (that is why we are having this discussion), others include movements, positive thoughts, relaxing exercises and good sleep, all of which help in activating the filter in the spinal cord. After activating the filter, fewer stimuli enter to the brain, just like when the ward heads learn how to effectively handle trivial and less complicated issues thereby reducing the workload on the Emir and minimizing the rate at which wrong or inappropriate decisions are taken by the Emirate council.

These strategies also activate the pain control system in the brain that releases the happy hormones (a double advantage!).

ML: This aspect is very important, I am a potential beneficiary too, can you please shed more light?

DB: In a smart way you have finally gotten a free consultation, ok let me breakdown the items for better understanding;

Pain Knowledge!

Getting educated about pain helps a lot, for example, now that the listener understands that, pain might not be related to damage or threat, so you don't have to be anxious anymore. And when your fears decrease, the brain will produce happy hormones and nerves will immediately start to calm down.

Stay active!

Don't say because you are in pain that if you move it will hurt more. Movement is important for the health of all body systems. We can remember the leg numbness we encounter when we travel for a long journey without changing position, we feel as if the leg is not part of our body but when we stand and move around blood will circulate and gradually we feel normal again, so also the body needs movement. Muscles, joints and nerves love activities. Exercises such as walking, farming and riding a bike, pump blood and oxygen around nerves. Brain releases happiness agents and nerves start to calm down. Therefore, try to increase your physical activity levels gradually, don't do too much on one day and nothing on another day, spread your works over the week days.

Remember that some more pain during exercise is not a sign of damage, it is your brain that is probably too sensitive for the information coming in through movements and a sign that your tissues were in need of some movement and reconditioning.

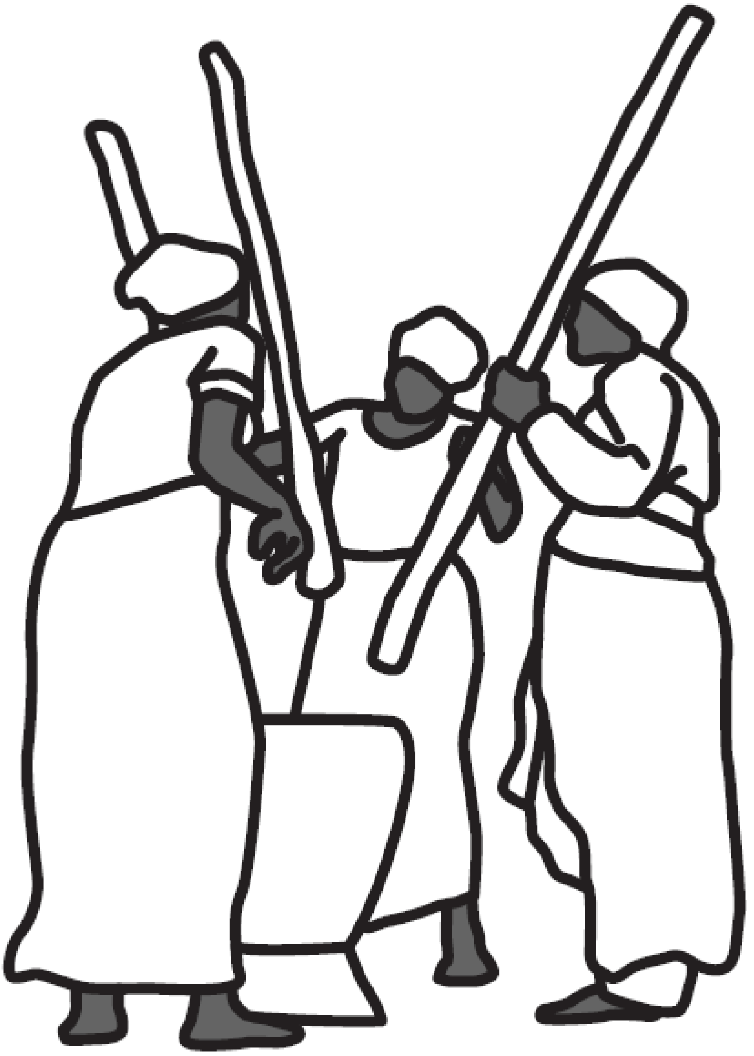

Take control!

Take control of your pain and let life continue. At every stage of life, you make decisions for many things some were even harder than the pain you are experiencing. This is your life. You must take control of your pain as well as your life.

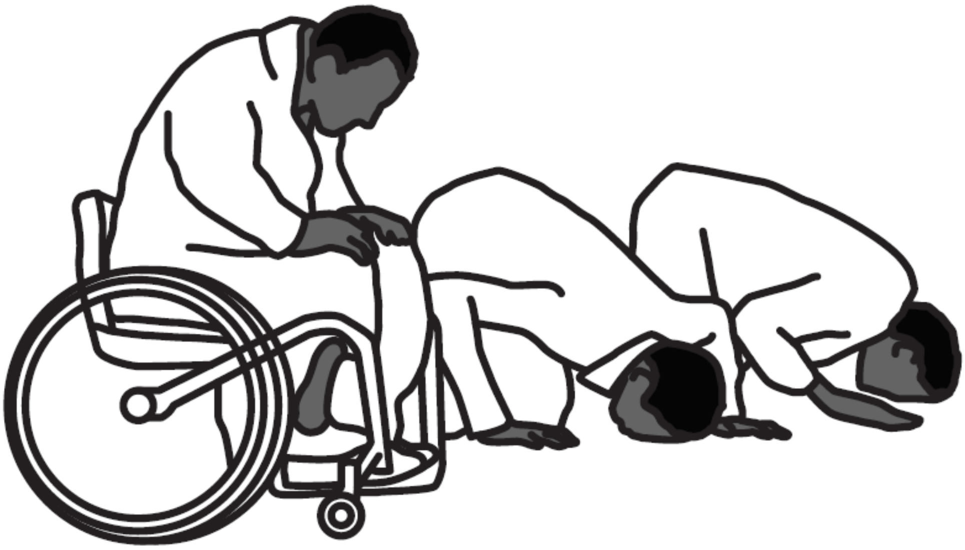

Relax and rest!

Sufficient rest and relaxing exercises will help to refresh your body. Relaxing helps to reduce stress level.

Stay positive!

You are not punished because of your pain, the best people you can think of, have also passed through pains maybe even stronger than yours. Don't blame yourself/your neighbour/colleague/spouse due to your pain. Try to avoid negative thoughts and beliefs and less worry about pain. When you stay positive, happiness agents will increase.

Sleep well!

Sleep quality and duration is also important for your health. Pain and sleep closely affect each other. If you sleep well, you will feel more energetic and have less pain the next day. Inversely, a bad sleep will make you to feel unrefreshed and worried, thereby making you to have more focus on your pain and subsequently ends up with more pain.

ML: Dr we have spent quite some minutes discussing this topic, I think we have to stop here, what will be your last message to the listeners?

DB: *Amfanin alimi aiki da shi* "the purpose of having knowledge is putting it into practice"

ML: Lafiya uwar jiki (Health is key to your wellbeing), may we benefit from the discussion.

I leave you in good health

Ma'assalam
