# Supplementary material for: Development of culturally sensitive pain neuroscience education materials for Hausa-speaking patients with chronic spinal pain: A modified Delphi study
Source: PLoS One. 2021 Jul 2;16(7):e0253757. doi: 10.1371/journal.pone.0253757 (PMC8253446; doi:10.1371/journal.pone.0253757)
Supplement: S1 Data — (DOCX) [file pone.0253757.s007.docx]

Delphi round 1.

Questionnaire link: <https://docs.google.com/forms/d/e/1FAIpQLSeJXbhx2PNE1eVxMgOxFhsqN3C3oodKaIRIW2CF2f6Jkj1dtg/viewform?usp=sf_link>

Responses obtained:


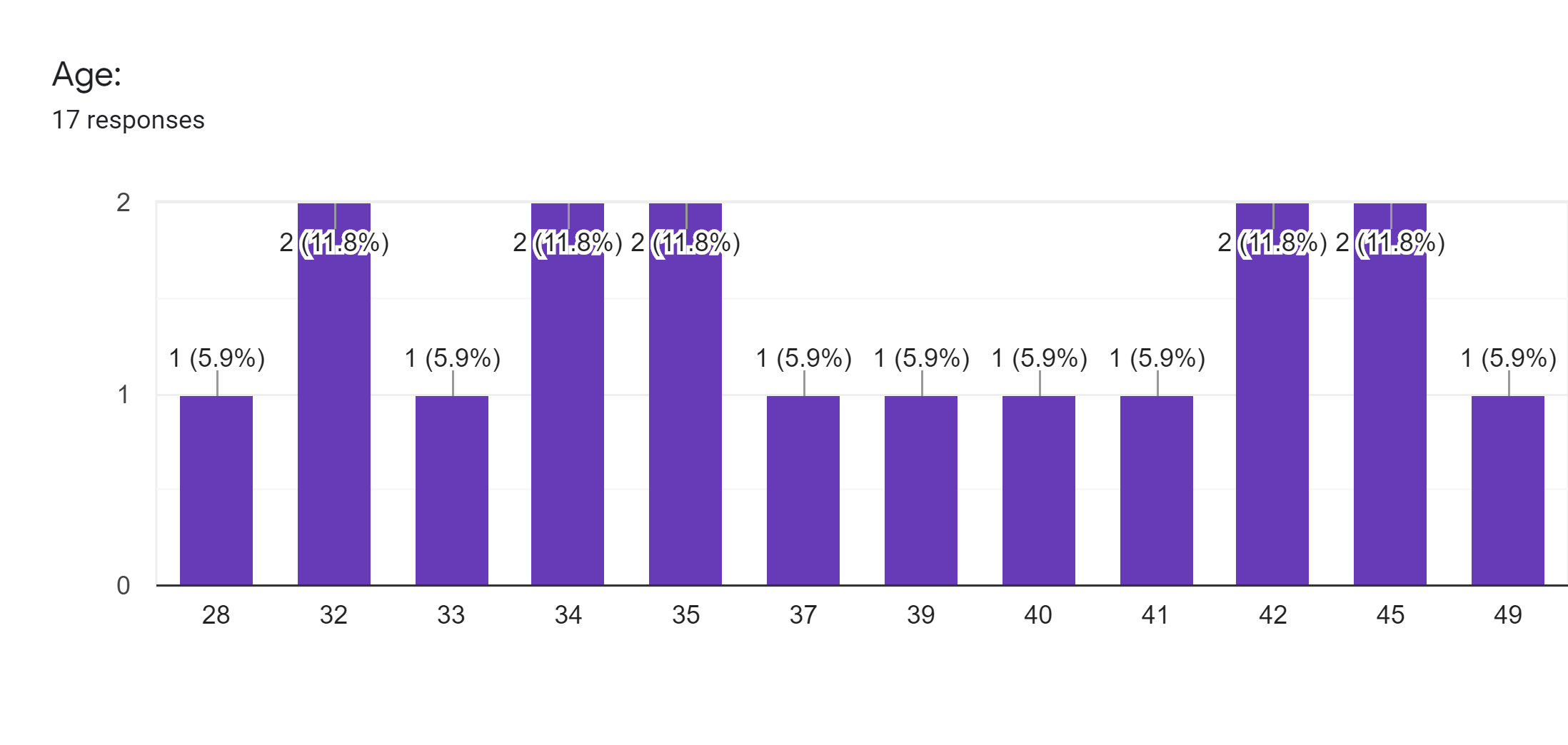


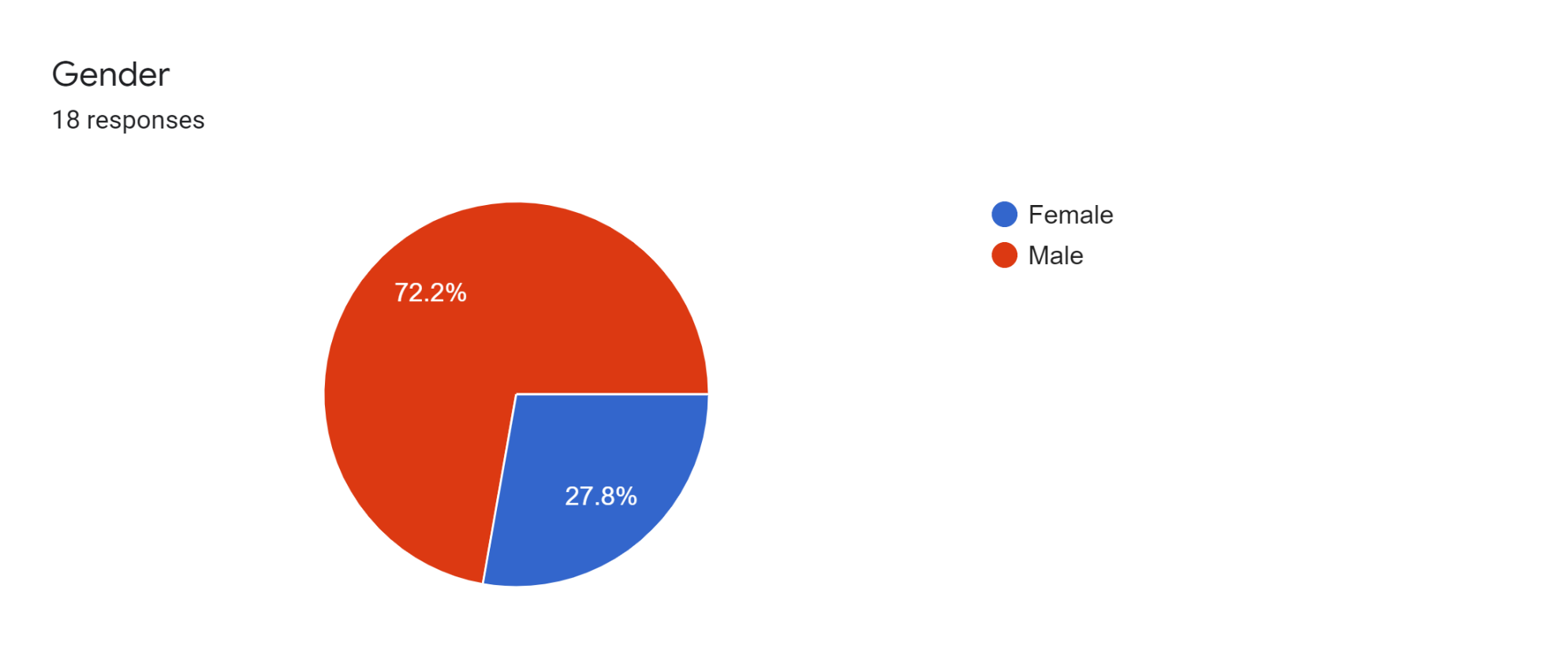


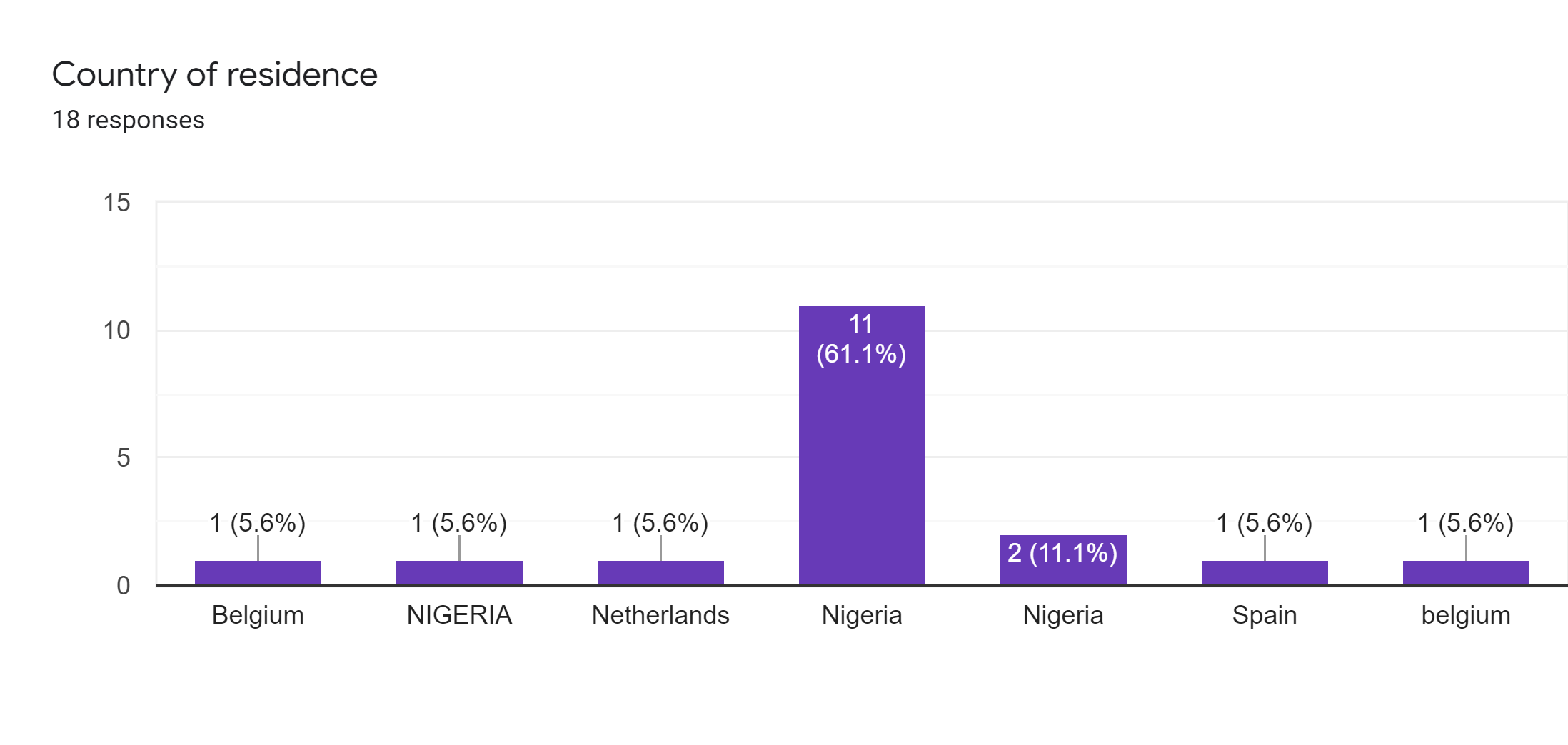


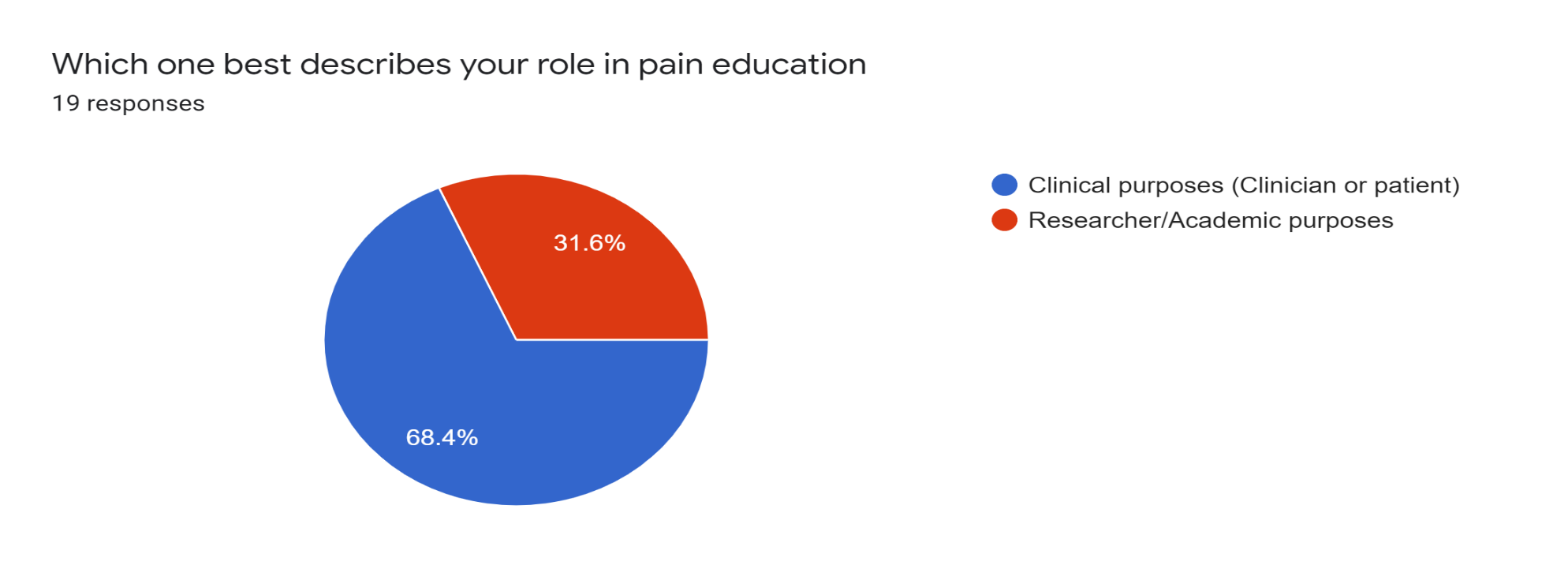


What is your experience with pain education?

19 responses

Non-existent 21.1%

Heard of it 15.8%

Familiar with it, <1 year 10.5%

Familiar with it, 1-5 years 15.8%

Familiar with it, 6-10 years 15.8%

Familiar with it, 11+ years 21.1%


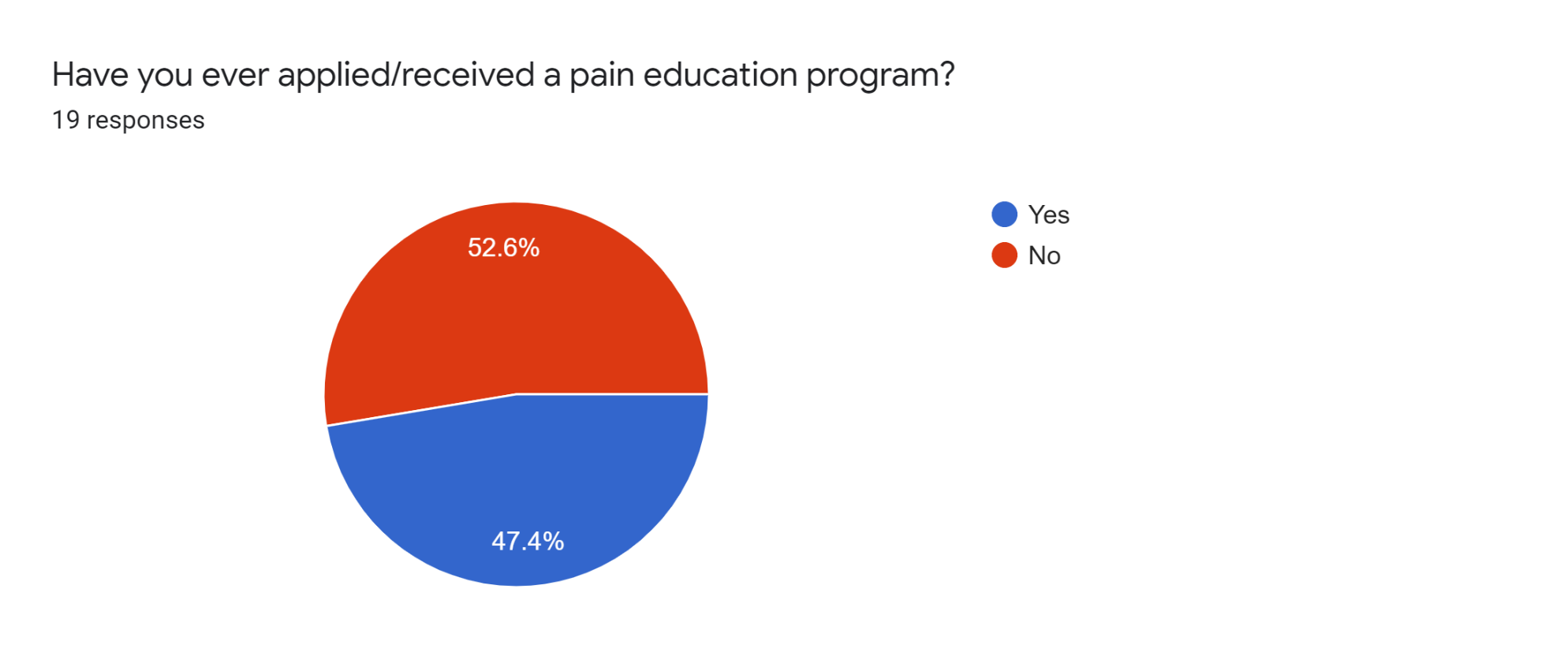


Acute Pain


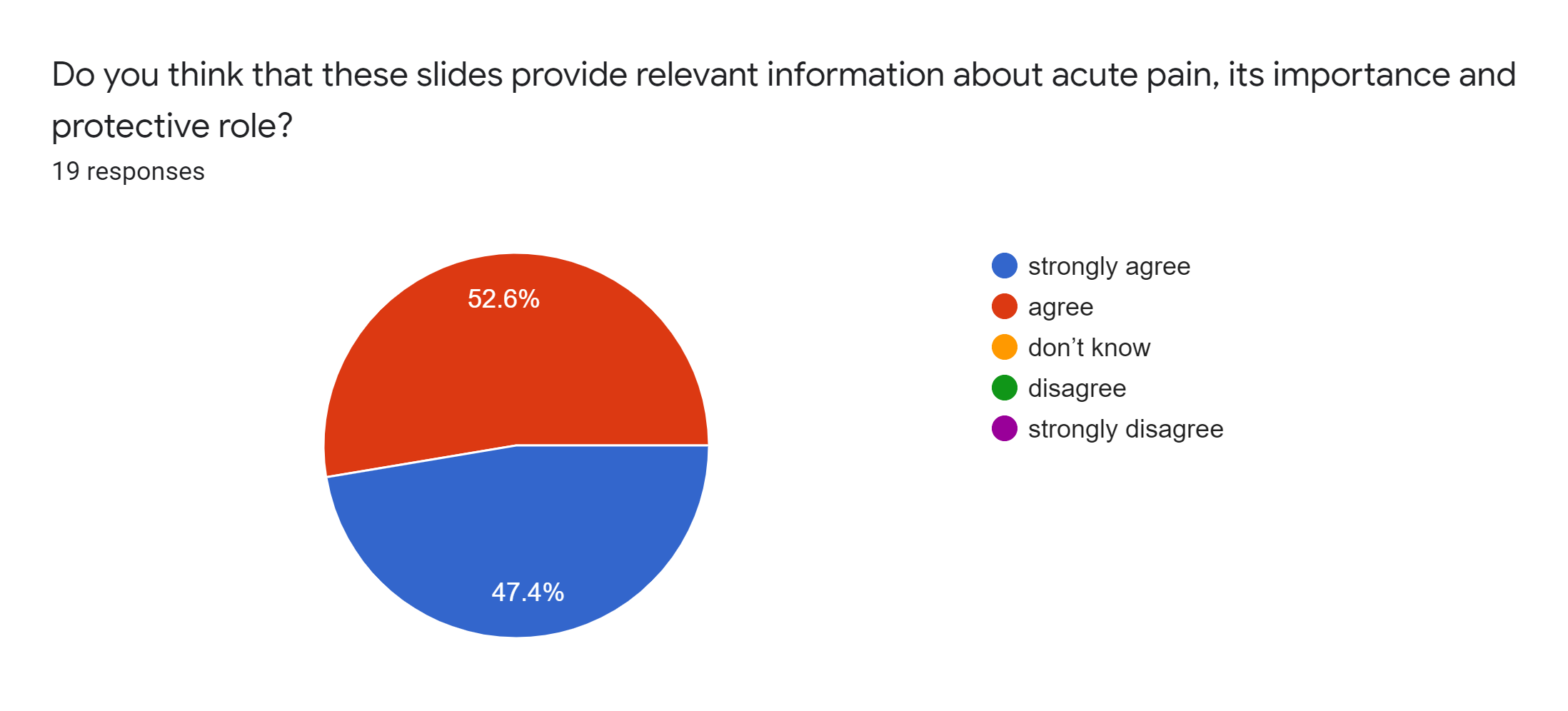


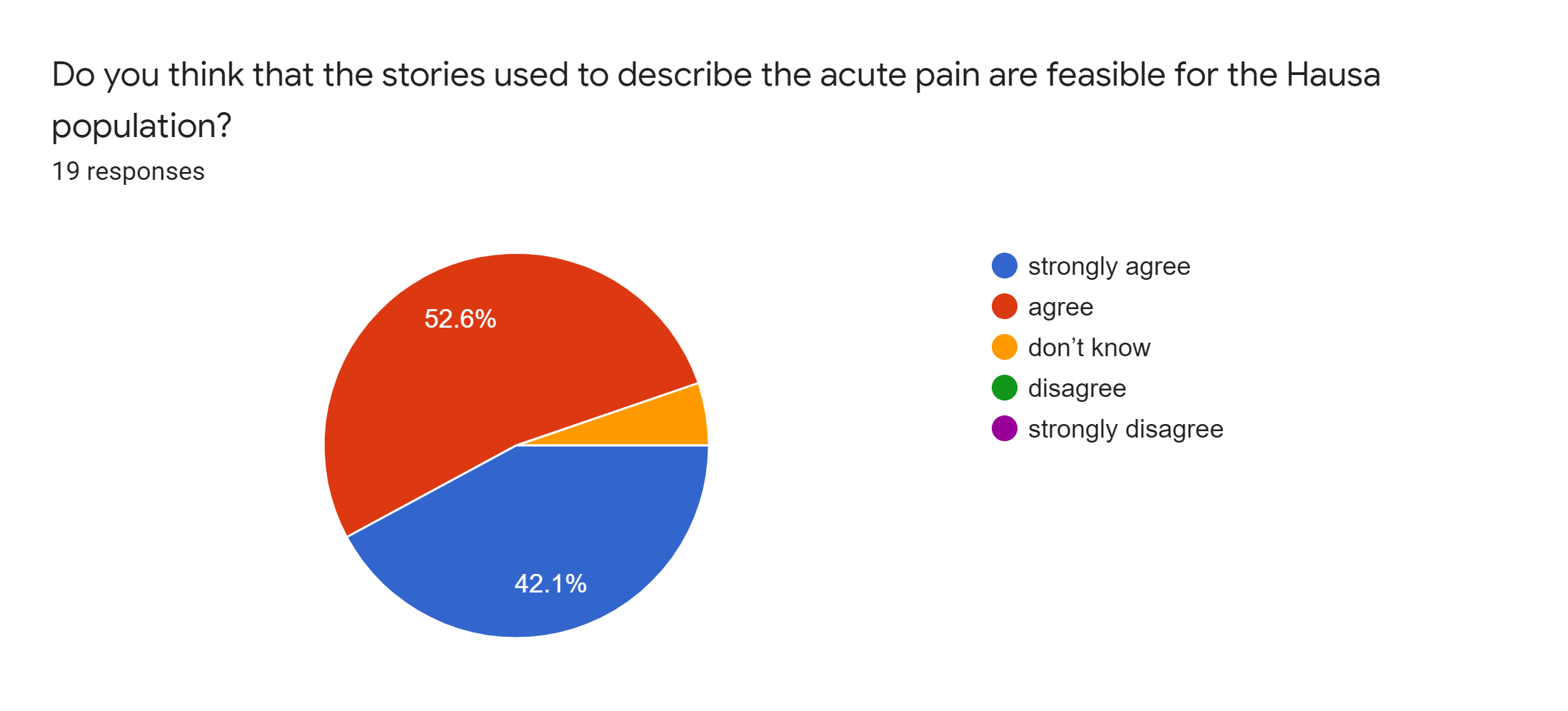


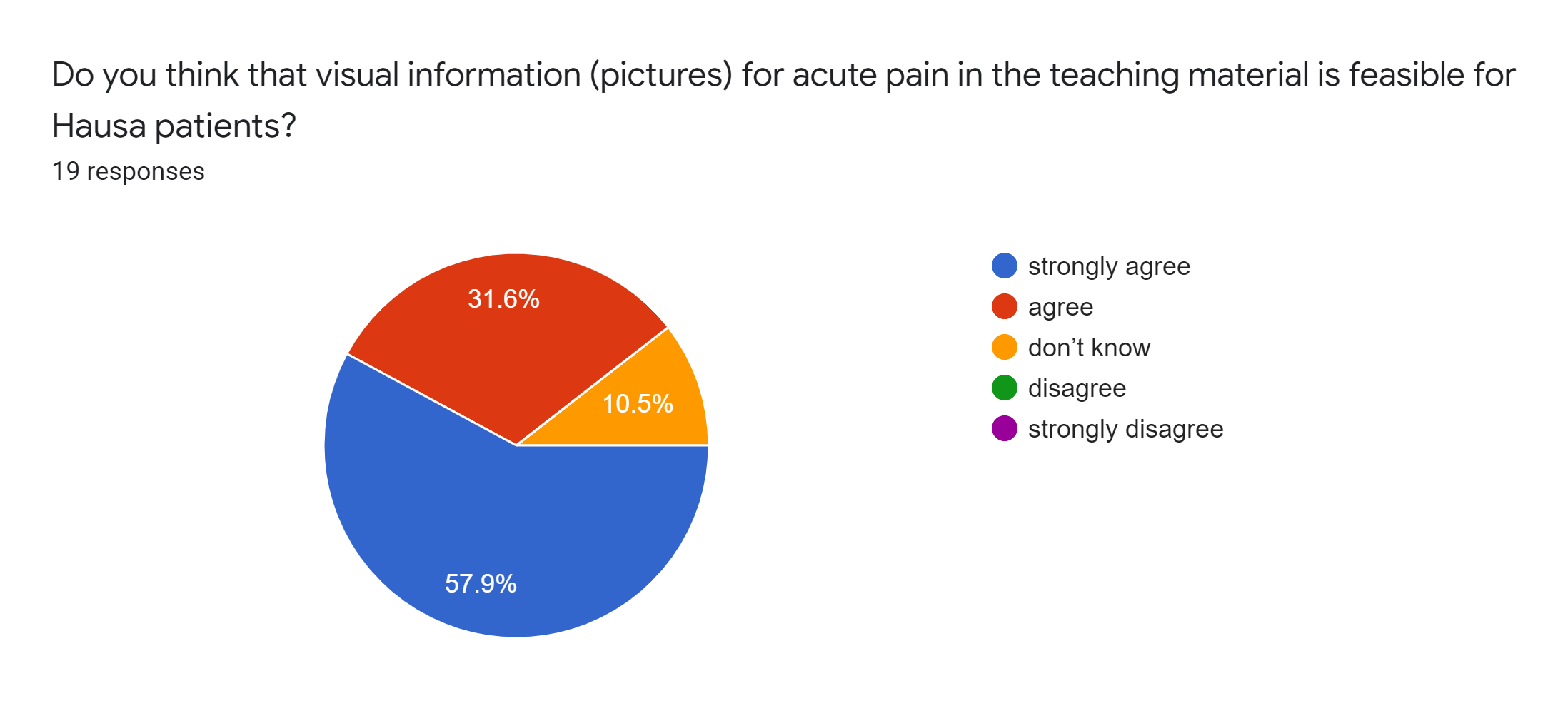


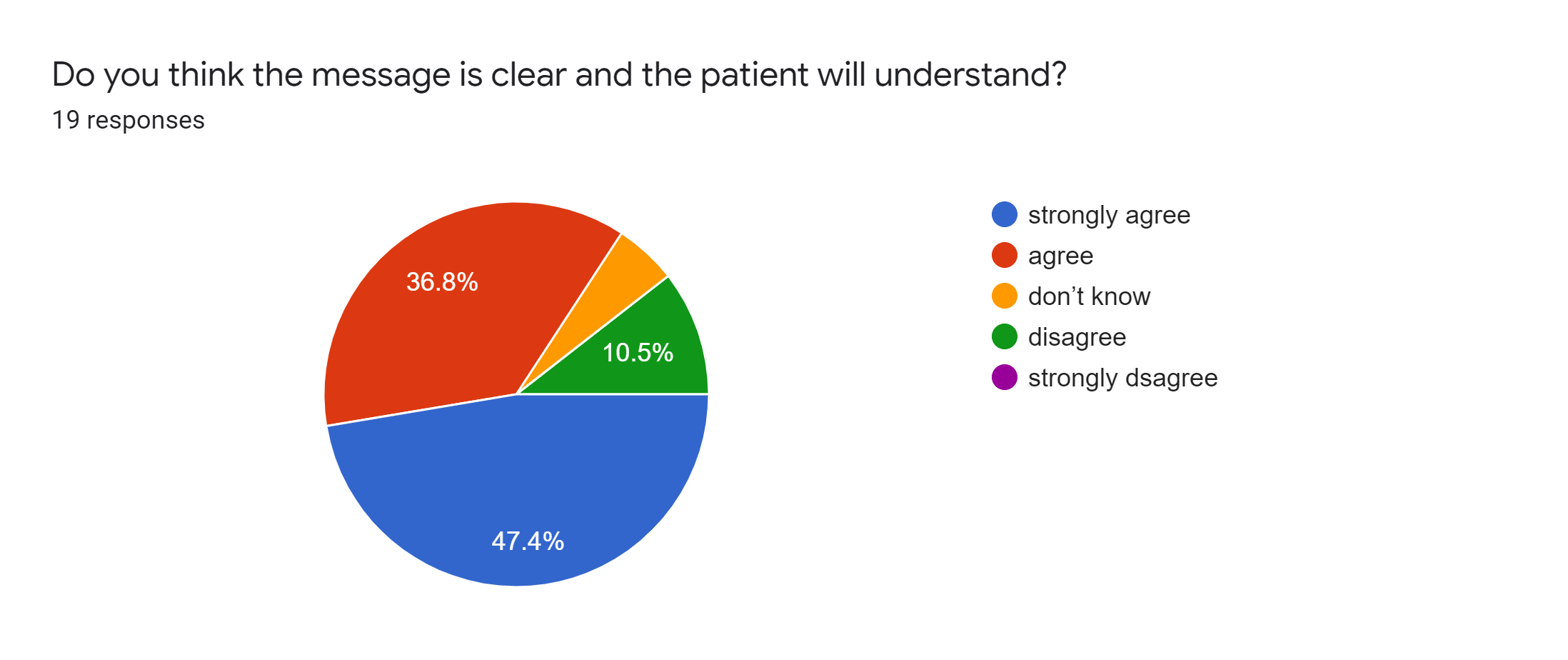


If you have any suggestion(s) regarding the description of acute pain, please write them in the box below.

9 responses

Regarding your example about the injury made with the hammer, I personnally prefer the example of an ankle sprain. Regarding the example with the collapse of the room, I am not sure it is a good example...as in such survival situation, people will tell you that they may not feel pain despite injury to survive the situation (and I am not sure it is what you want to illustrate here). Furthermore, will the people understand a sentence like "by special receptors through nerves to the spinal cord and then to the brain in return stimulus (iron touch)". Is it not necessary to remind/explain first what spinal cord is ? Regarding the metaphor with the door's spring, ins't it easier to use the metaphor with the alarm/security system?

-not sure whether yhe example of an iron is applicable to a major part of the population

I think the translation of some words need to review e.g. Dadadi I think it should radadi

You should have an cultural adapted version, I am not aware of how the Hausa patients wil respond or which cultural specific perceptions they will have an if these examples are meningful, why show somebody with low back pain in an acute pain picture, is back pain common in Hausa people?

Nil

Acute pain is sudden and it can cause a learning effect

None

Make it more educative

No

Pain biology
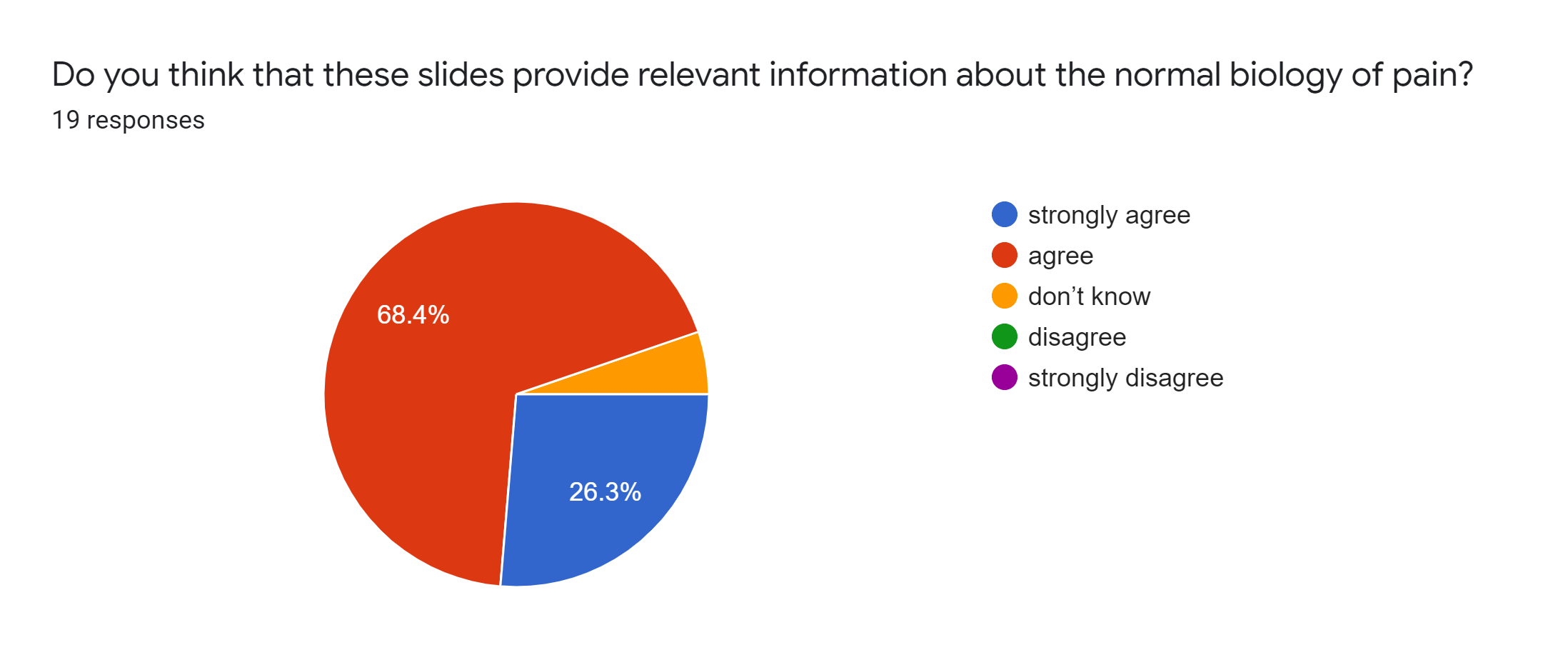


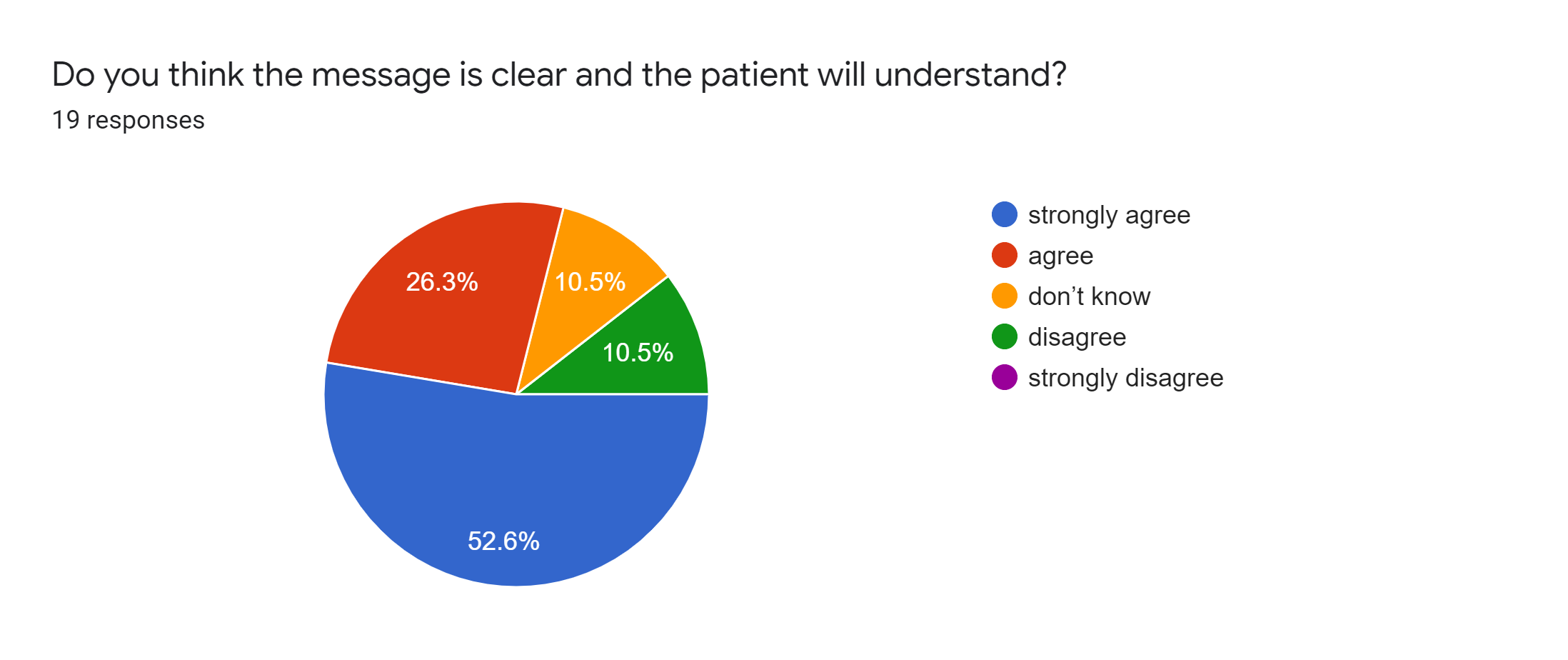


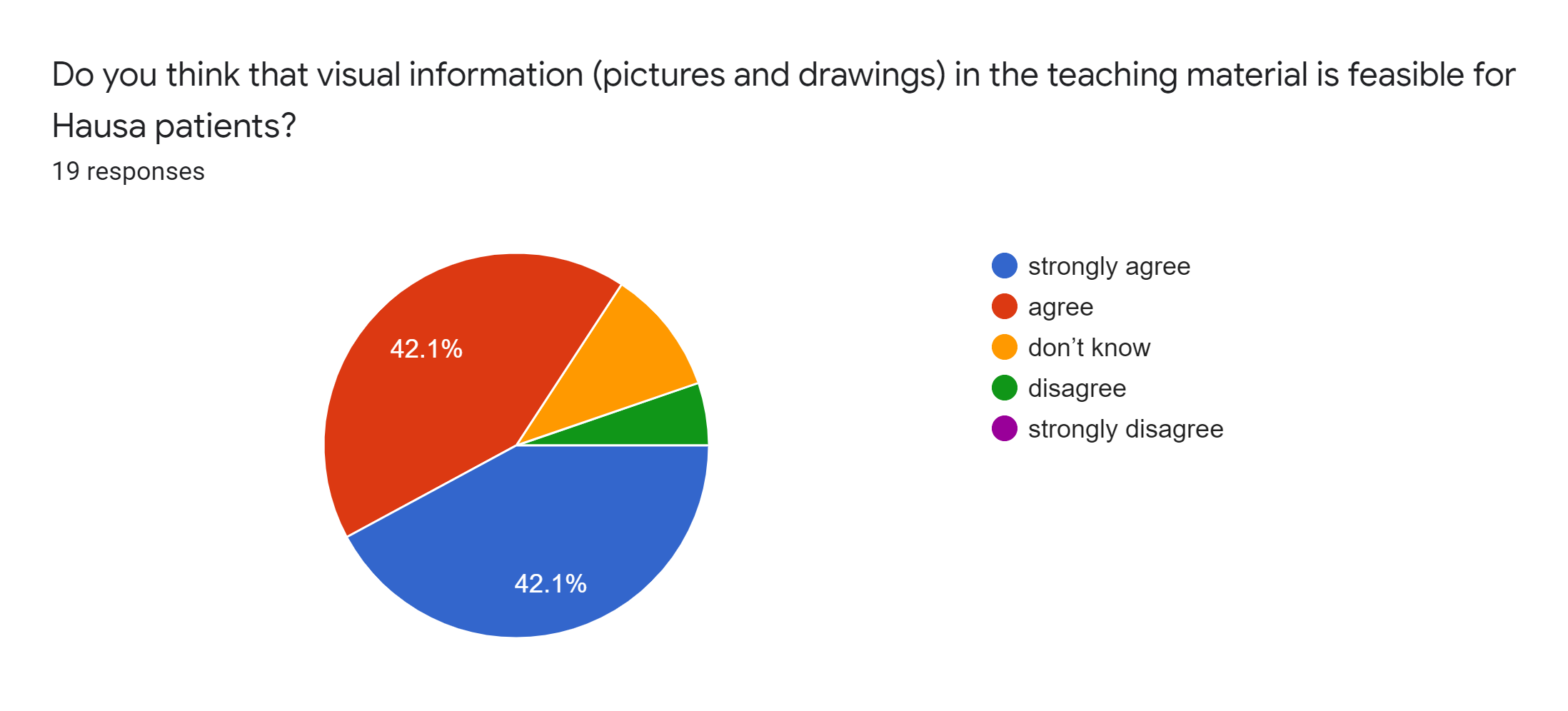


If you have any suggestions regarding the explanation of the normal biology of pain, please write them in the box below.

12 responses

none

The pictures are not clear enough according to me and I am not sure the text below them will enable people to understand the pictures. Can you really state that "sensors detect painful stimulus"? (what is a "painful stimulus" ? Indeed an identical injury can cause pain or not cause pain depending on the context). Will people understand "...transported to the nerves through the action of chemicals...". Not sure they will understand the picture present in the slide 16

-not sure whether the drawings and information of receptors etc will be understood, depends of level of education -how are you going to translate receptors, sensory nerves ,.. ?

Give additional information regarding the role of the spinal cord as an amplifier of pain

I have problem with some words

I am not aware of the cultural context it seems logical ans easy ti explain, although maybe add some more cultural specific examples

Nil

The biological images used are not much, you can add more next time.

Addition of more pictures that will explain pains

None

Health-workers should be responsible for educating patients about pain biology

Pain Modulation


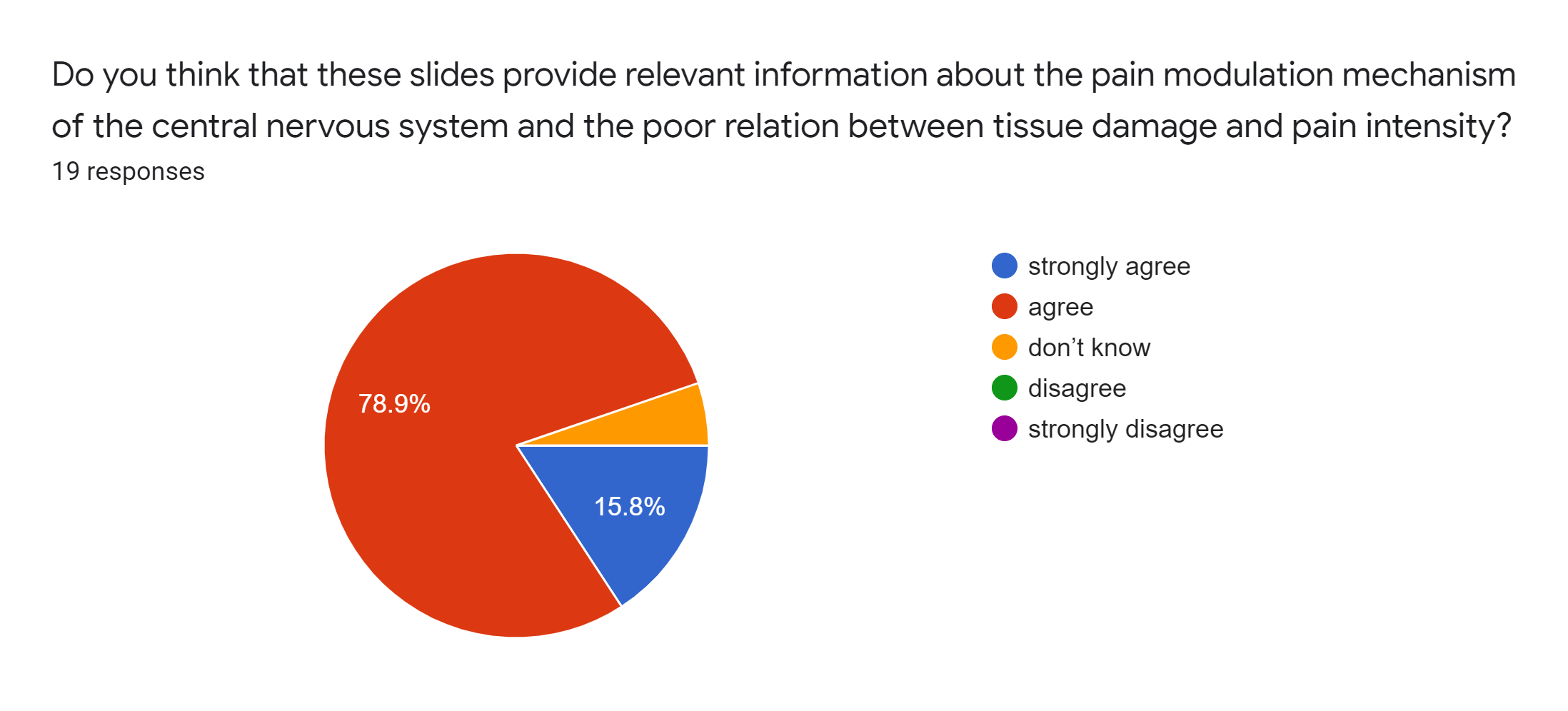


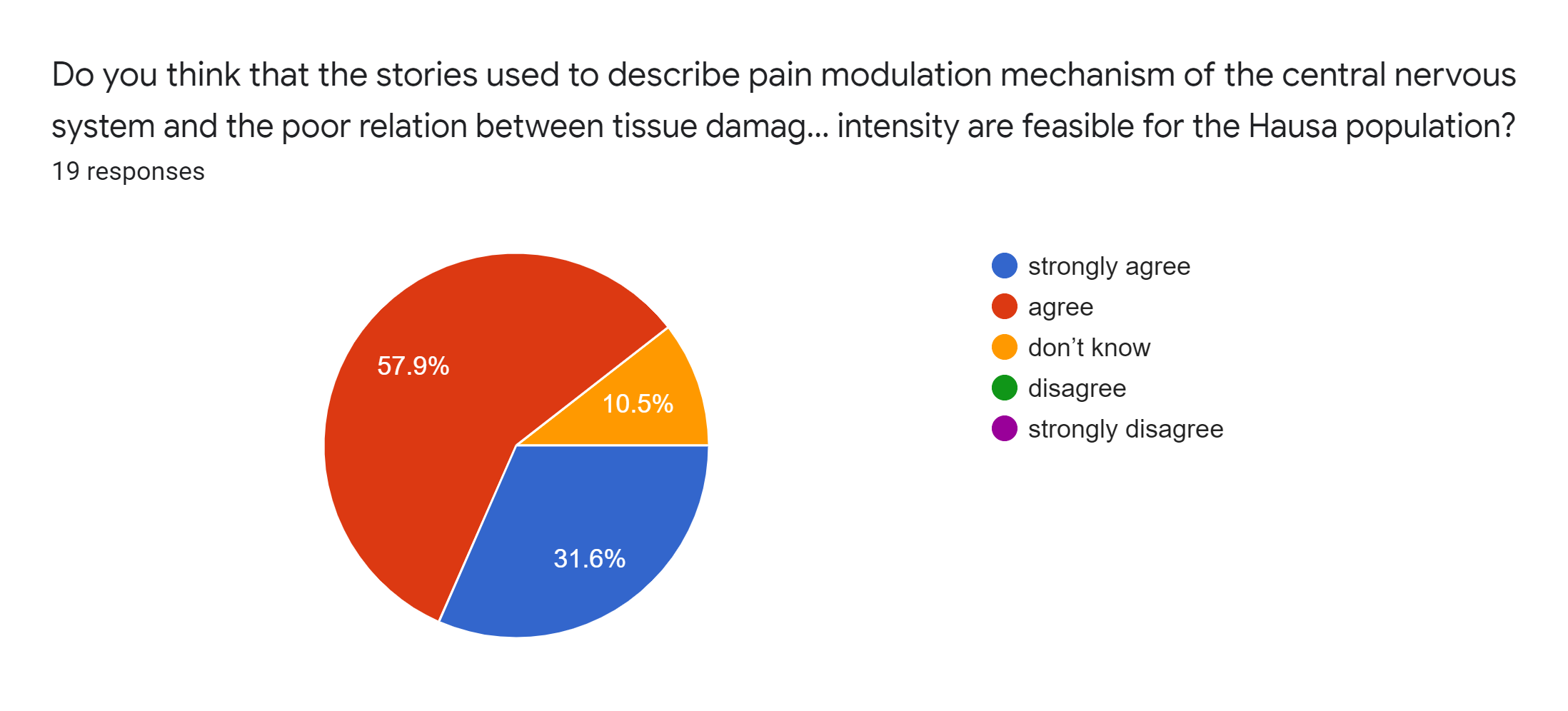


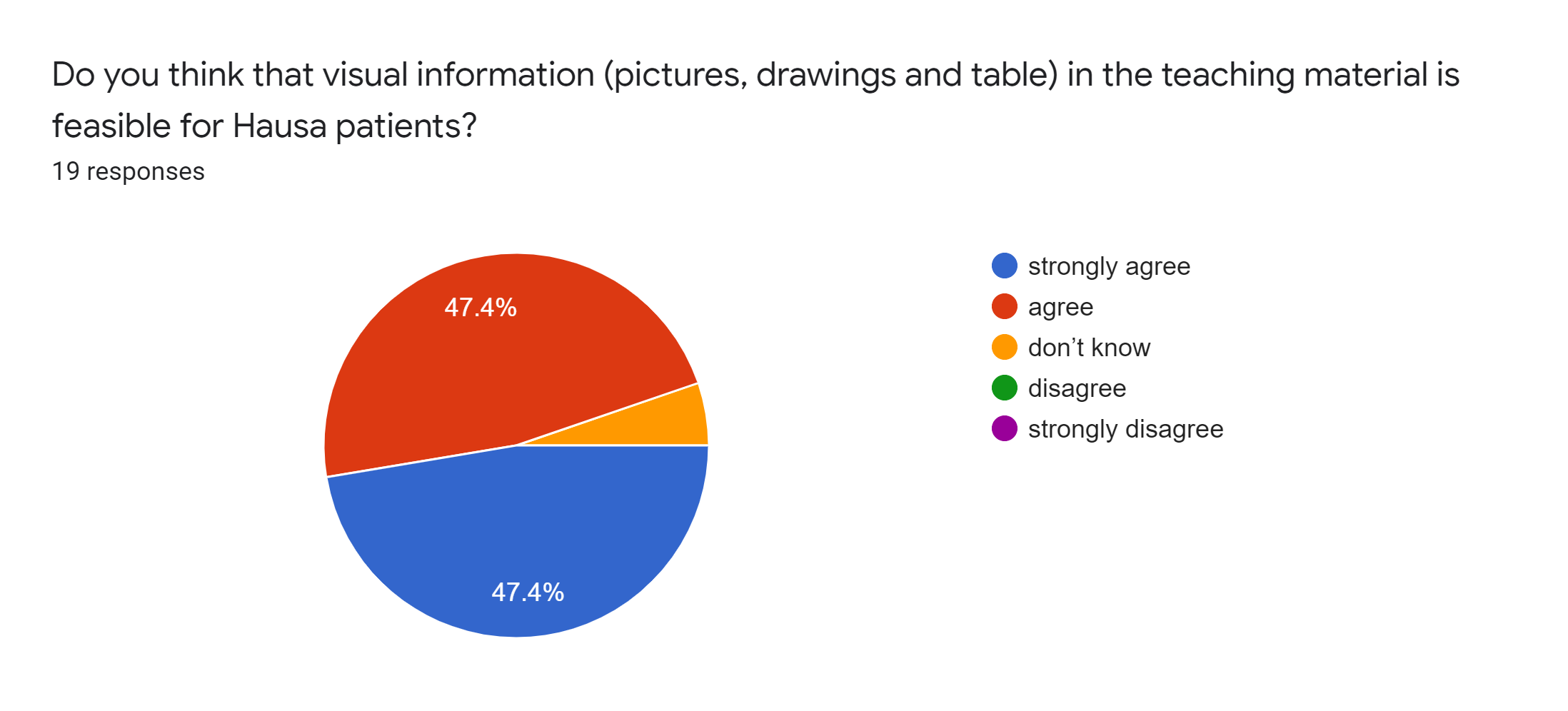


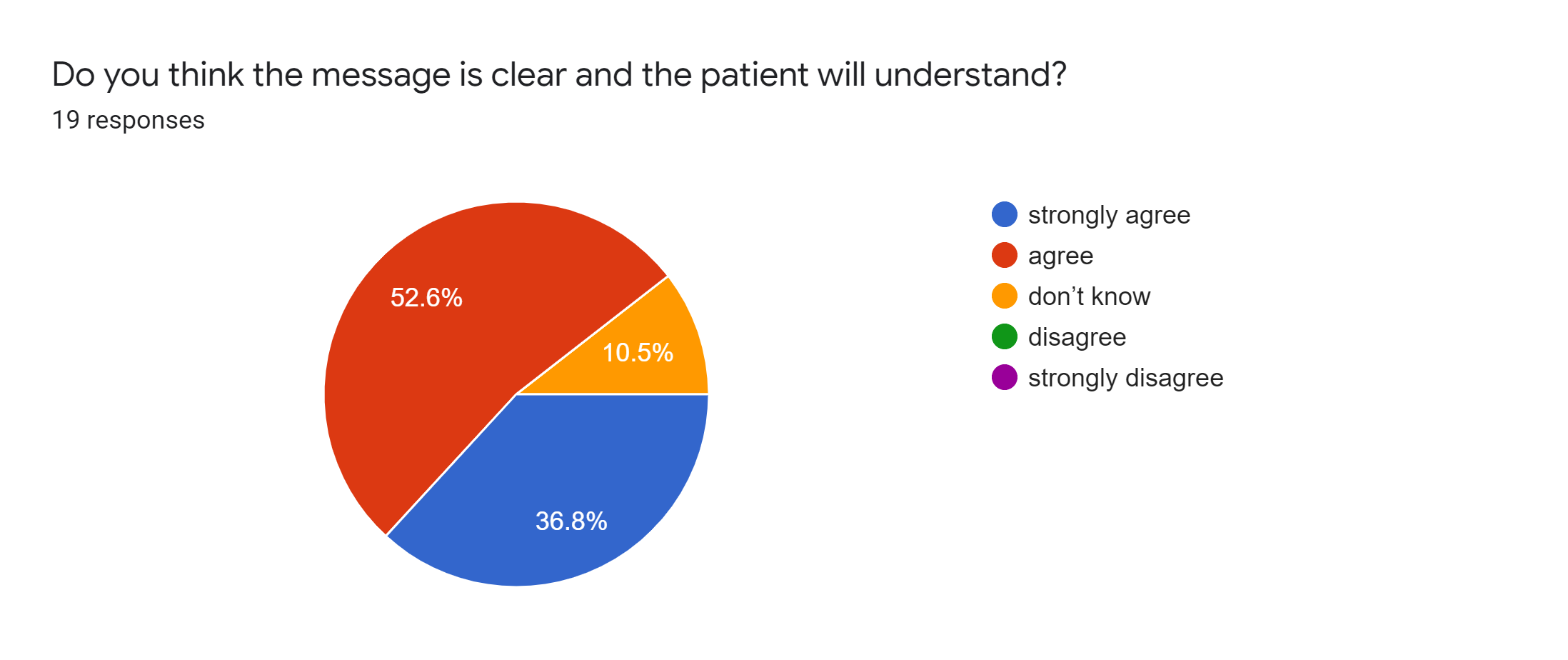


If you have any suggestions regarding the explanation of the pain modulation mechanism of the central nervous system and the poor relation between tissue damage and pain intensity, please write them in the box below.

10 responses

None

Slide 18: Abdullahi is upposed to have headache, not low back pain (see picture). Can you really state that people doing shadi competition do not have any pain? Ii is sure that it will be less painful than if I was participating and that they can manage not to express any sign of pain but are you sure those people say afterwards that they did not feel any pain at all ? Slide 19: I am really not sure the people will understand the table. Are the sentences below the table questions or affirmations ? Slide 23: the foot injury looks severe on the picture...you should make it much less severe

-not sure whether the drawings used ,like spinal cord cut, and sound sign))) will be understood

Give additional information regarding the role of psychosocial factors (i.e. cognitive emotional sensitization) in chronic pain

Common metaphors related to Hausa people's culture should be used to provide clear and simple explanations of pain modulation mechanism of the CNS and explanation of "harm is not equal to hurt"

slide 19 also use other examples not only back examples f.i. headache or stomic pain

Upgrade of pictures

Pain matrix


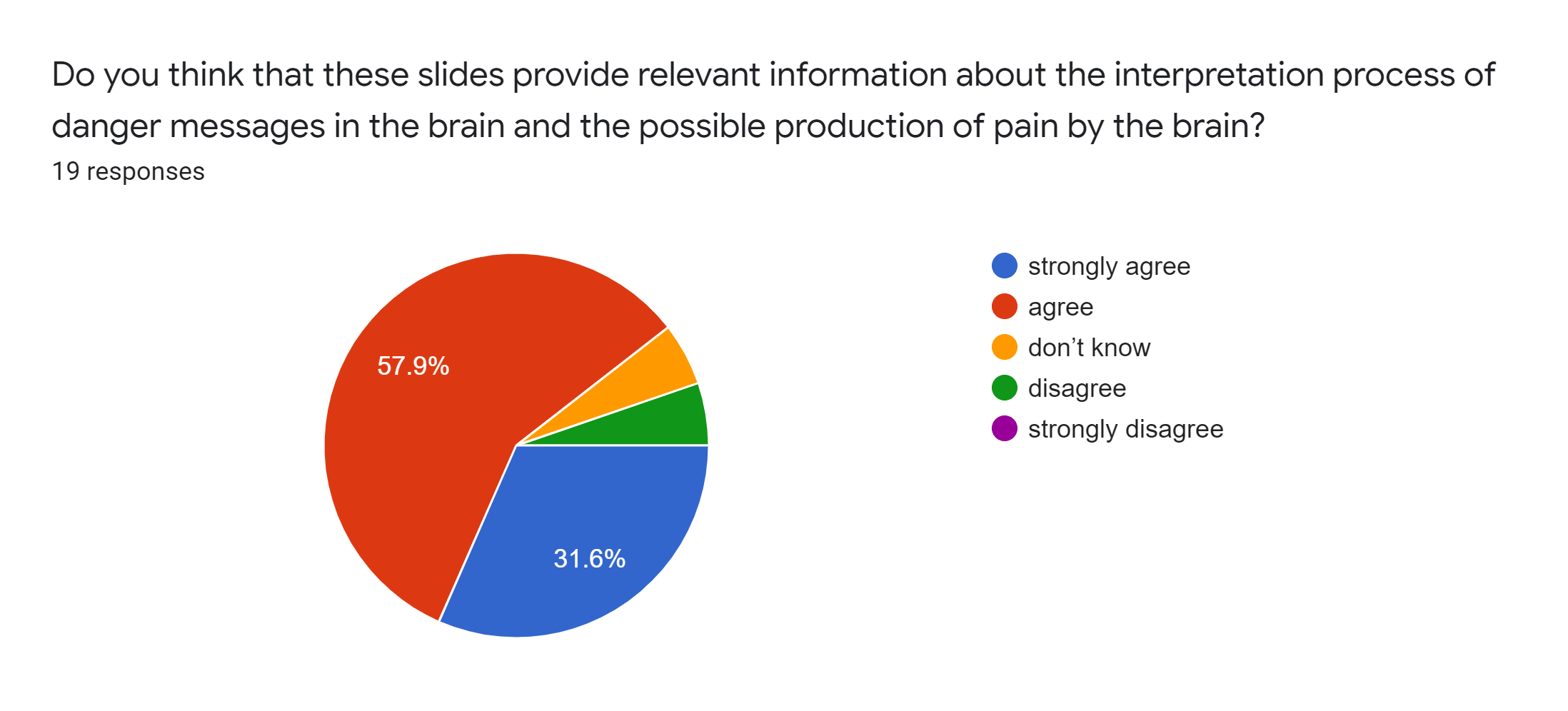


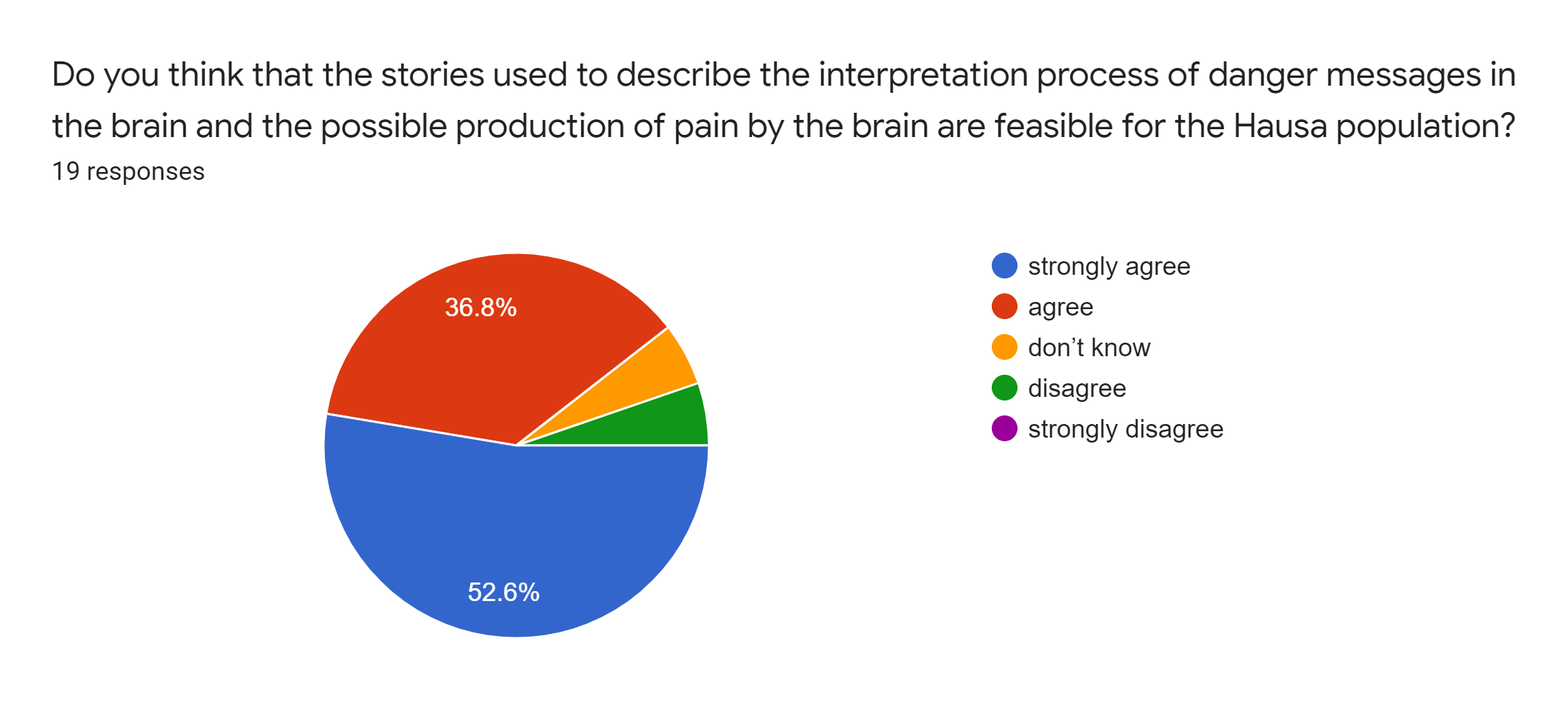


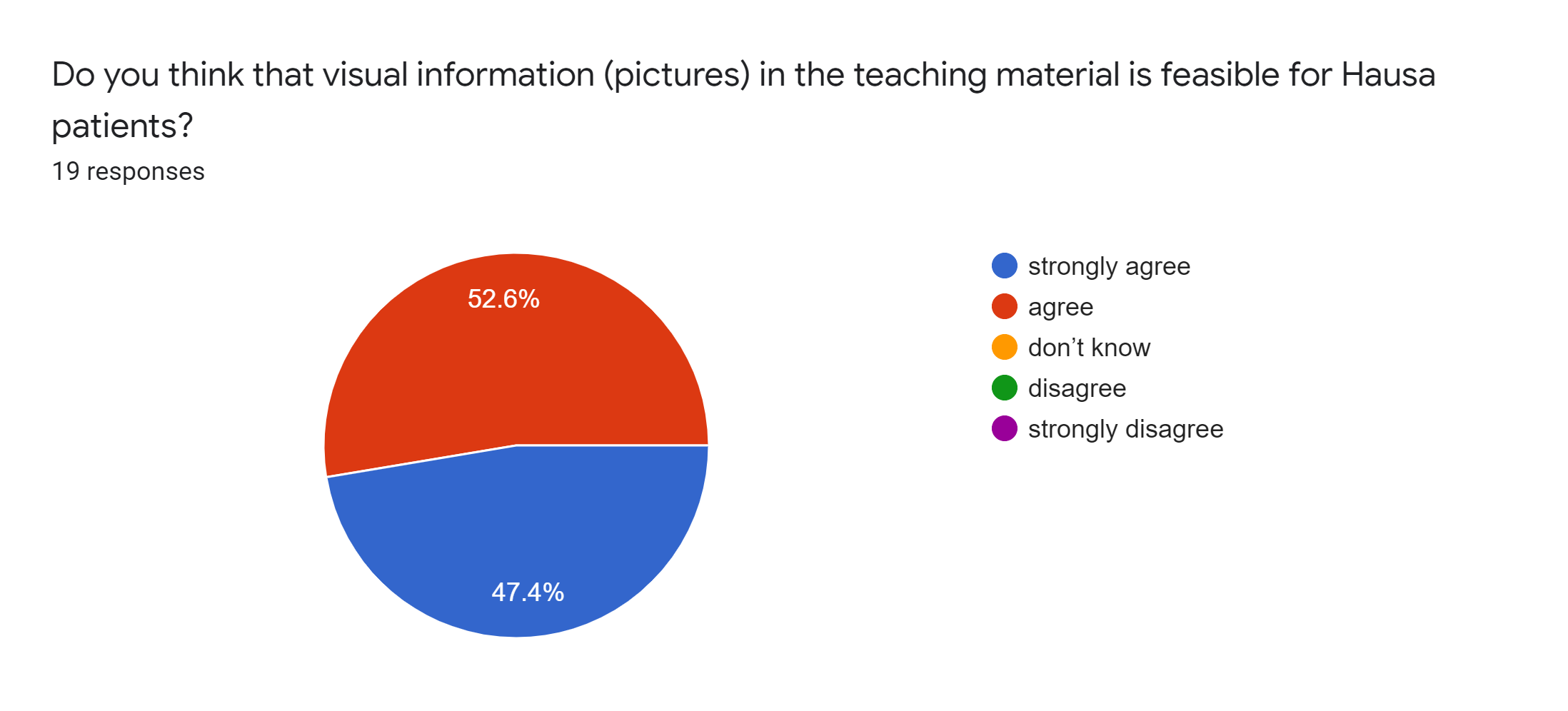


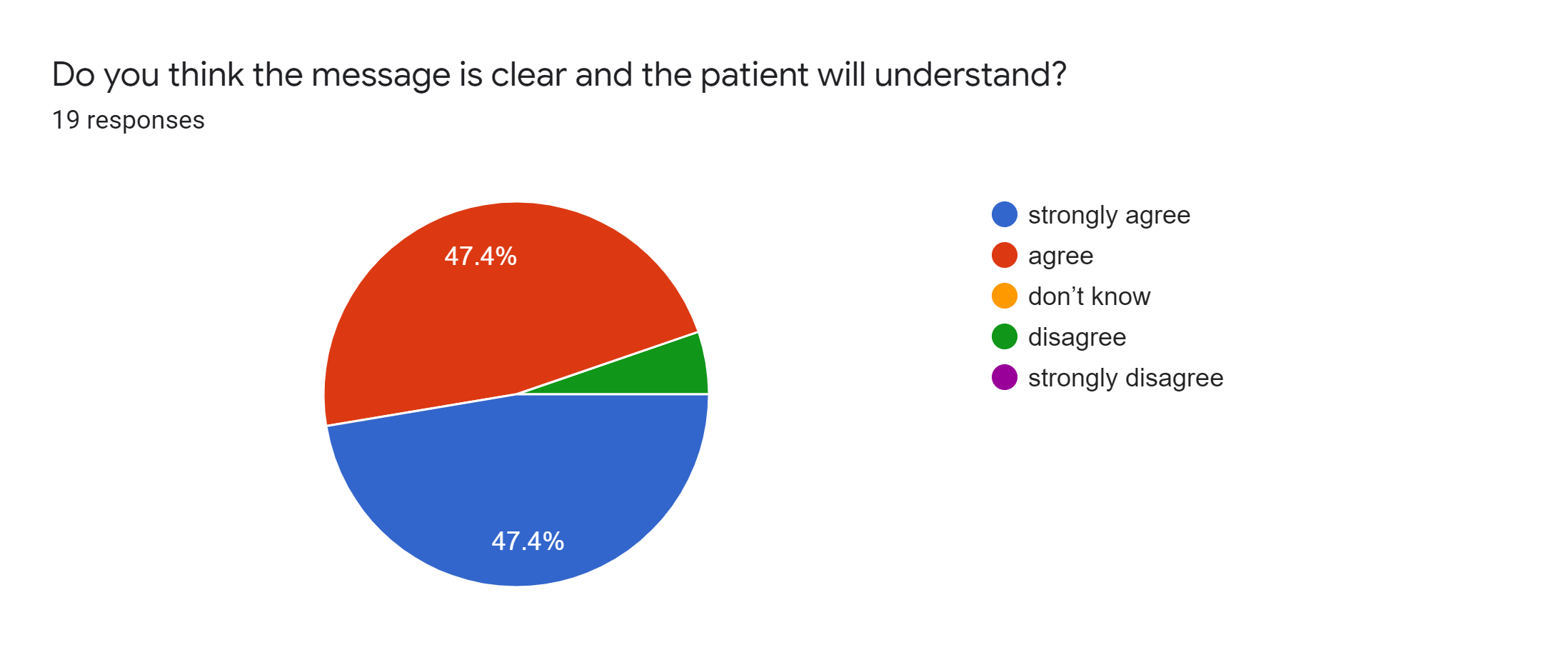


If you have any suggestions about these slides that explain interpretation process of danger messages in the brain (pain matrix) and the possible production of pain by the brain, please write them in the box below.11 responses

None

I think that you should better explain in the text that, as for how the traditional council works, danger messages are filtered at several points and that only some messages arrived at the brain. But it might be relevant to state that in some circumstances, the brain (chief) might be willing to get ALL the info....or to be NOT disturbed (because of a more important thing to do: e.g. to try to survive)

-

Give additional information regarding the role of specific brain áreas involved in pain

Some words need to be review with some patients who has experience of pain

nice cultural examples important

Nil

The pictures and interpretation should be expanded.

There is need to educate patients about how to avoid these injuries (electric shock, sharp objects etc)

No

Chronic pain


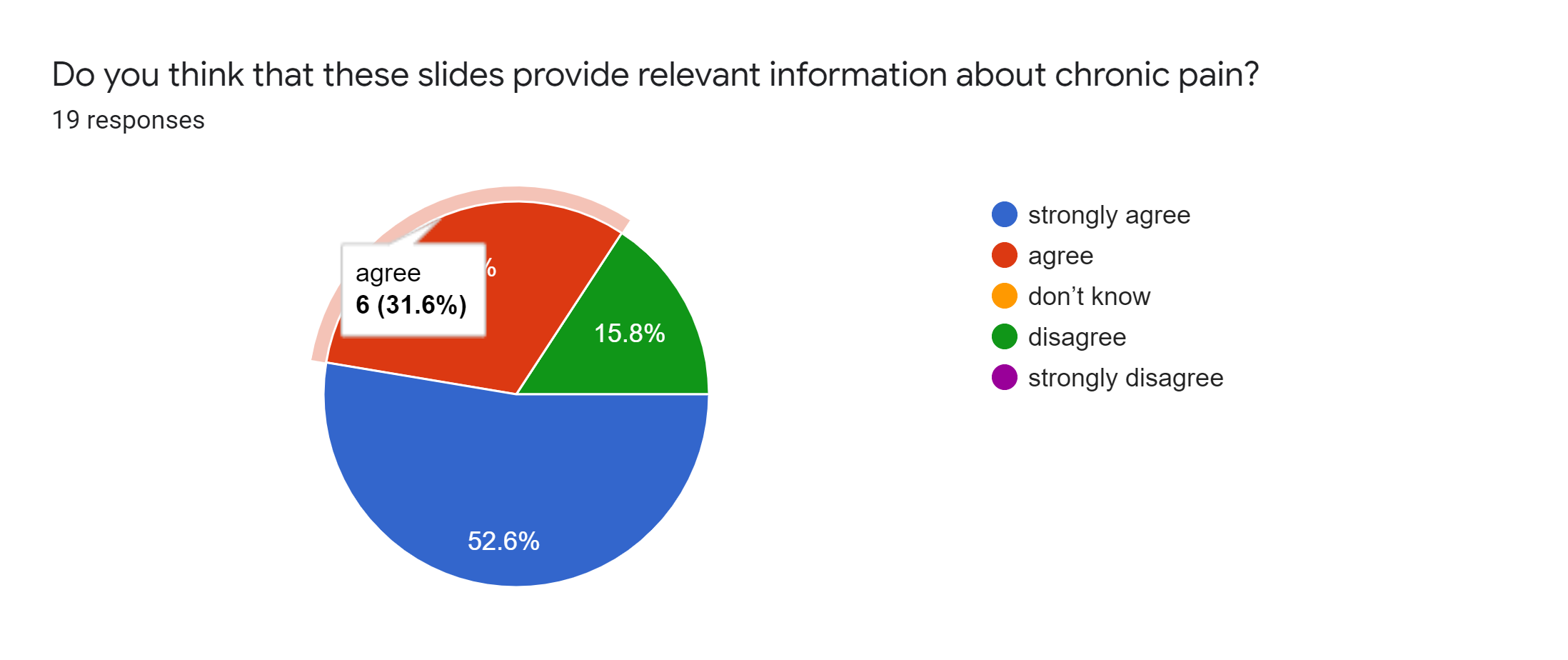


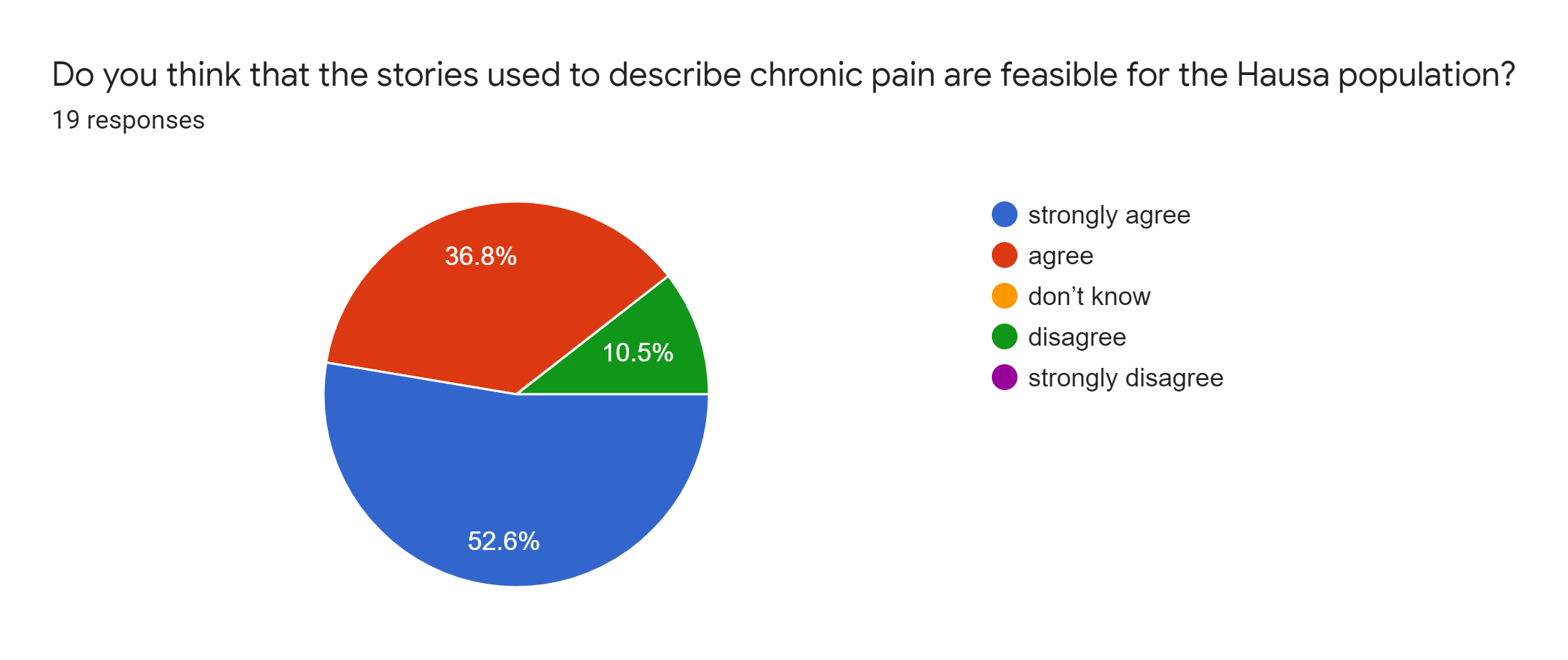


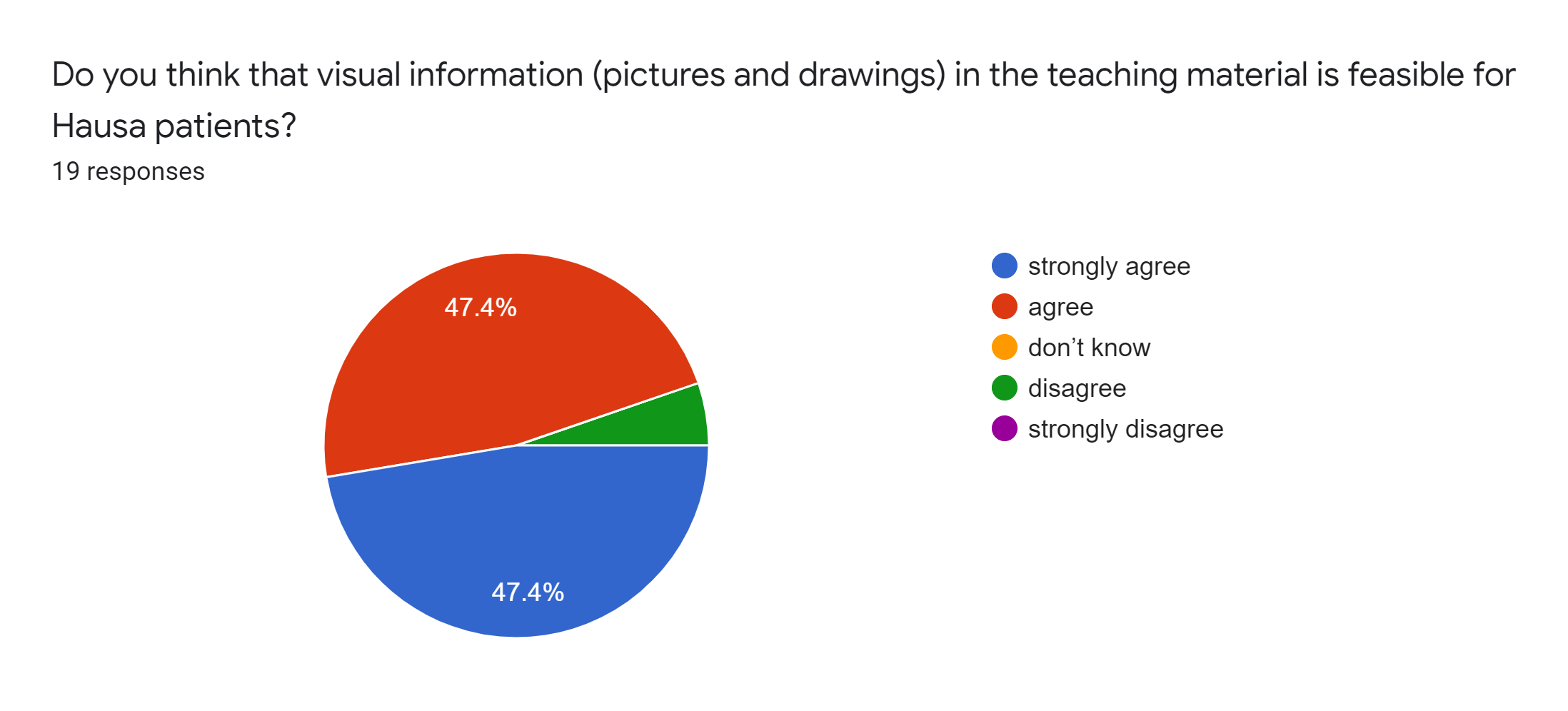


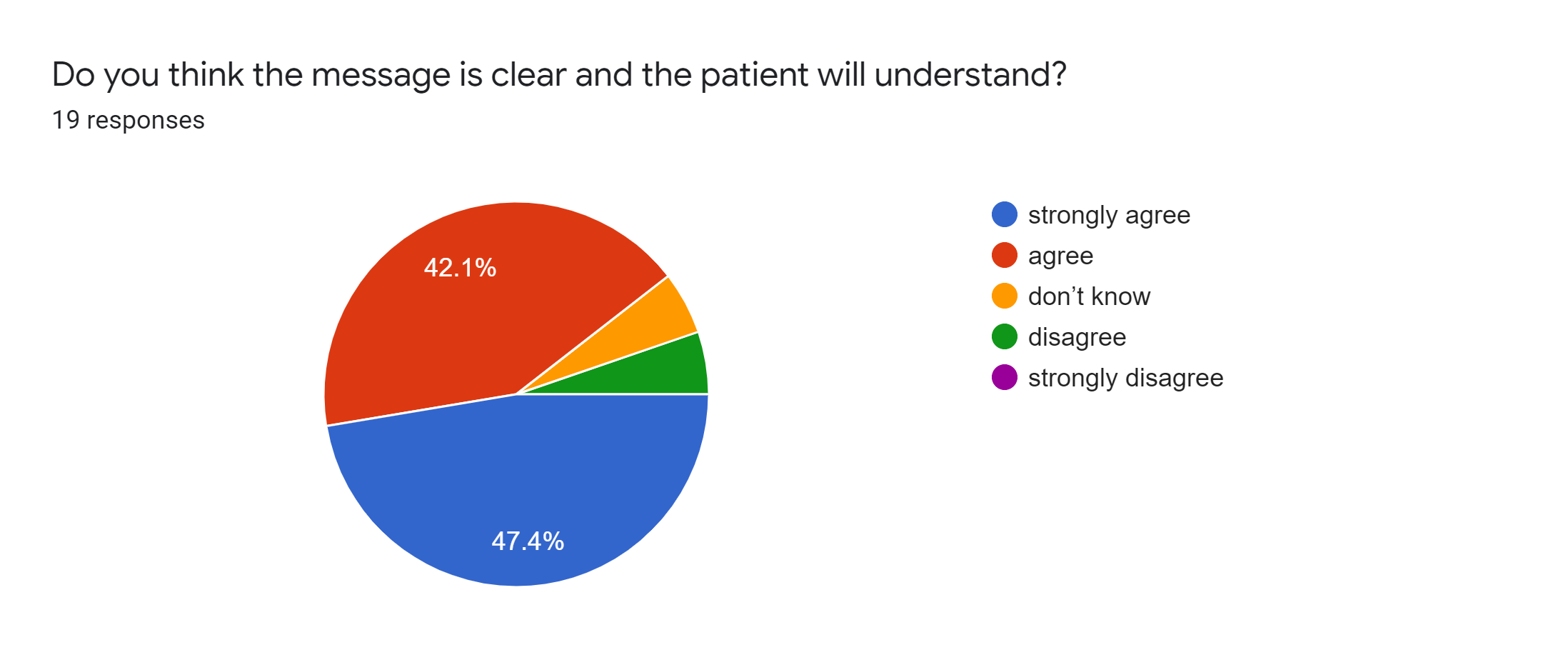


If you have any suggestions regarding the description of chronic pain, please write them in the box.10 responses

None

Some of the pictures are not clear to me (e.g. slide 28, what are the two treatments on the left of the picture?). Slide 27: not sure they will understand what you mean by "not clear definitive cause". Maybe some of them will think: for me it is a disc problem, facet osteoarthritis,...

-

Please reviews the questions with patients

please integrate psych-sociale issues these can also be important in the existence of pain make them cultural specific

Nil

More explanation is needed.

No

Psychological aspect of chronic pain should be emphasized

Beliefs, thoughts and behaviours


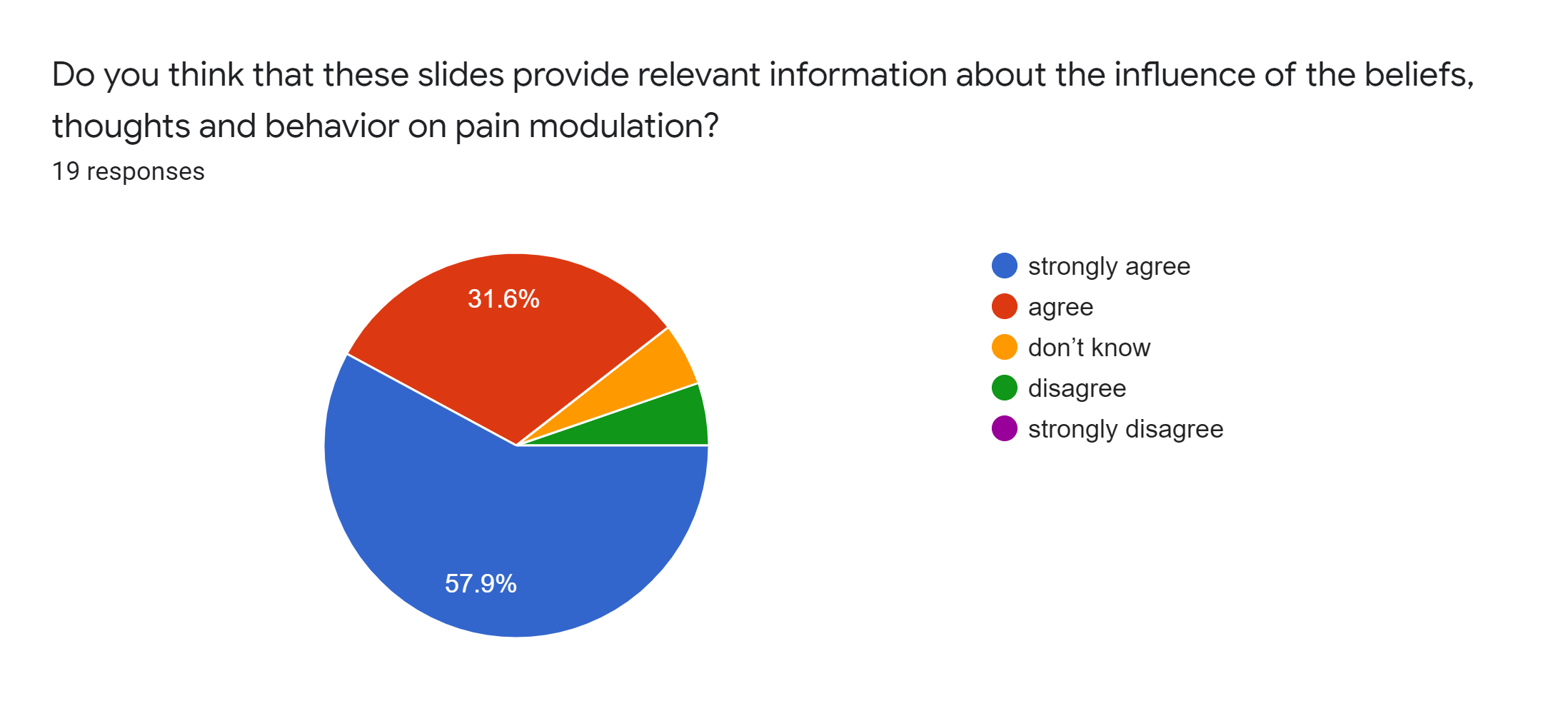


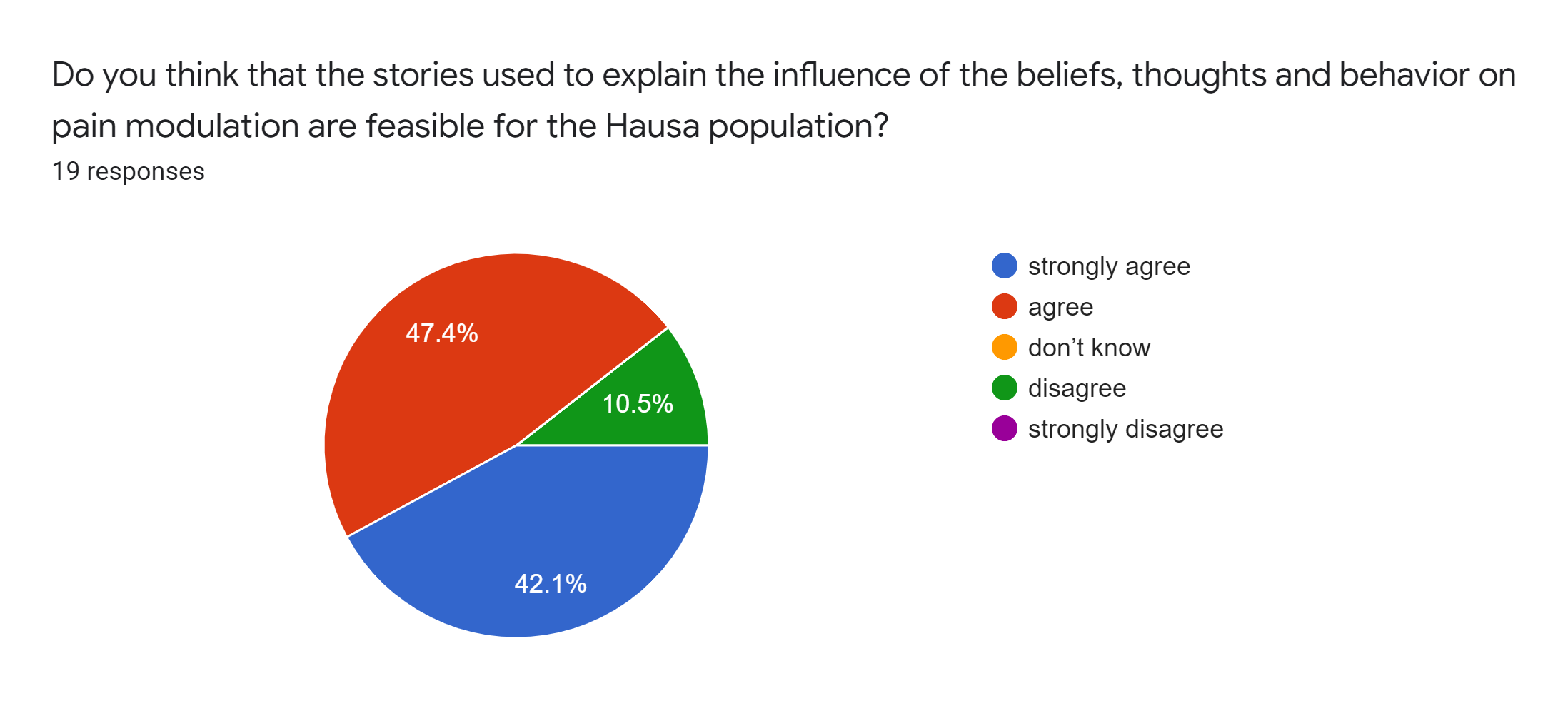


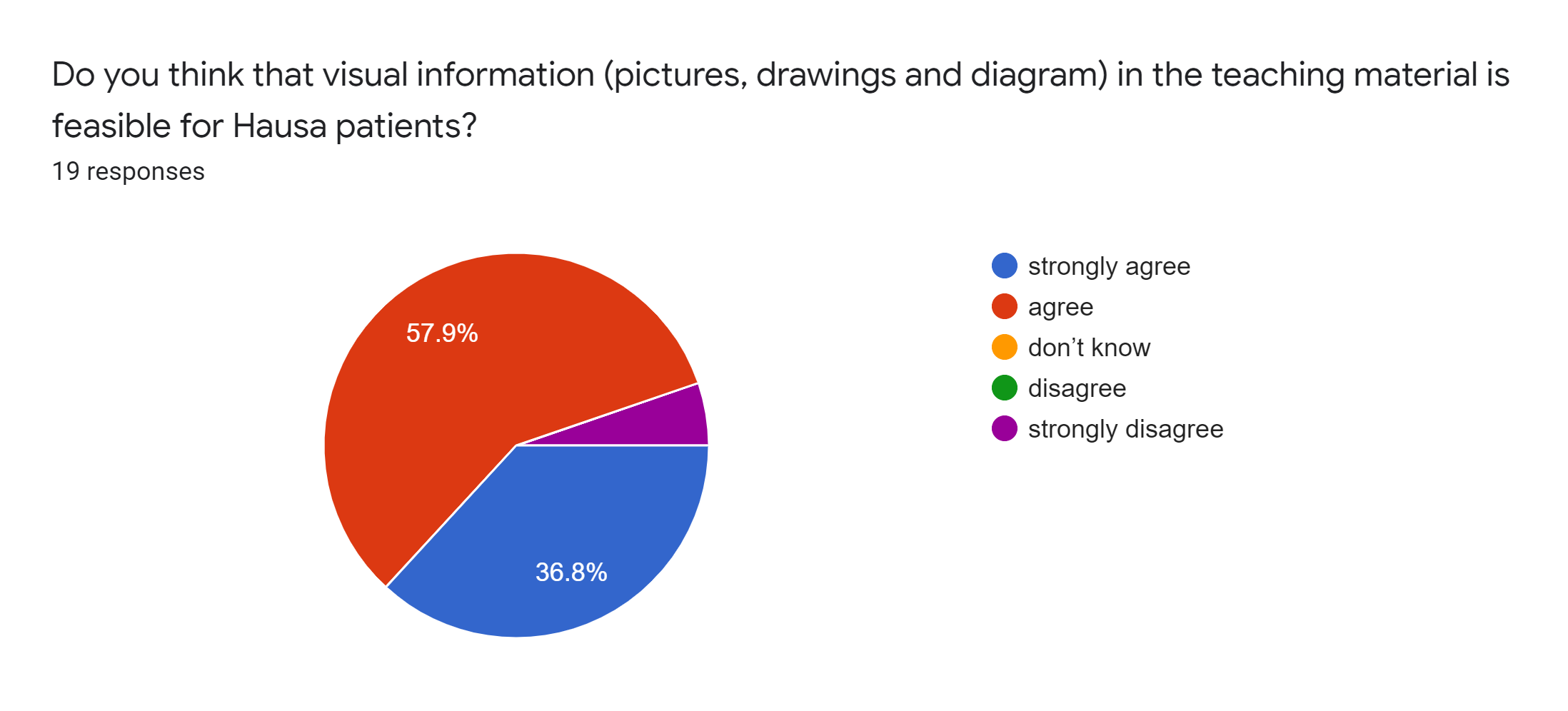


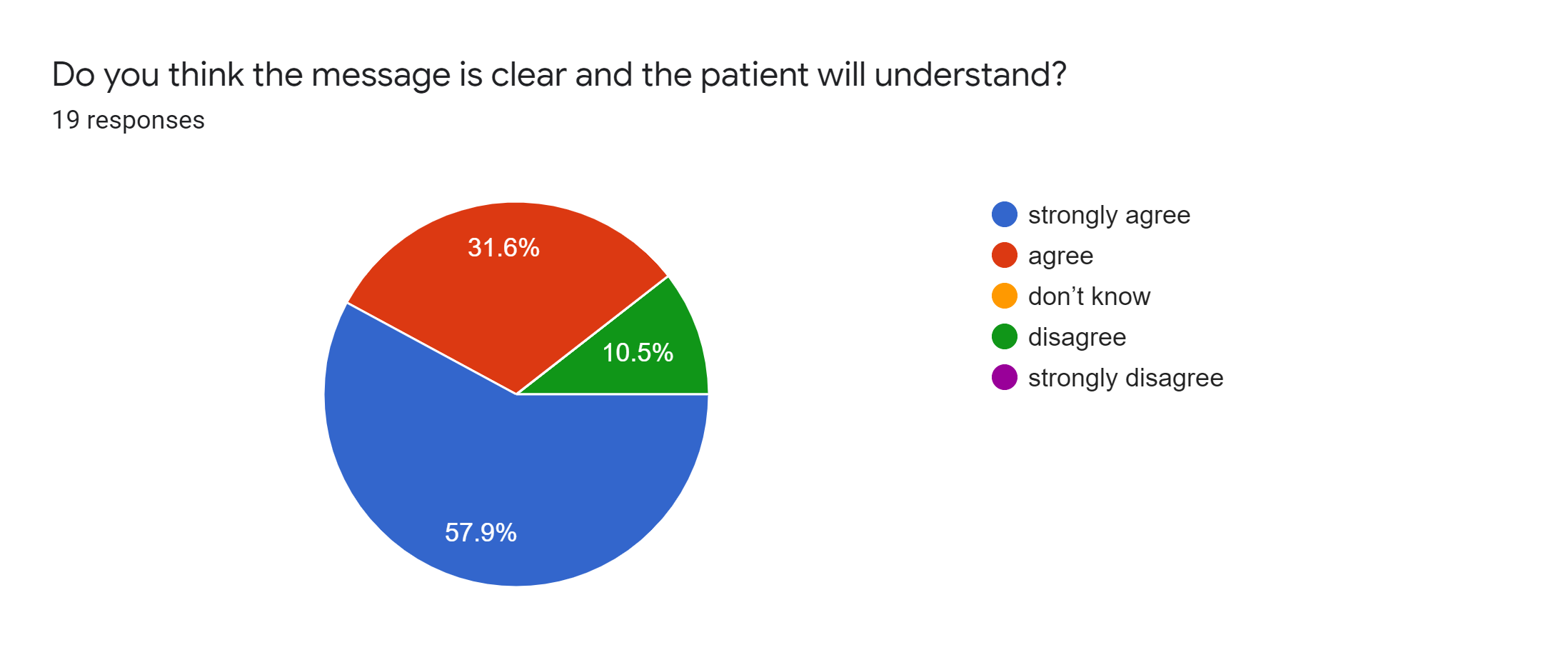


f you have any suggestions the influence of the beliefs, thoughts and behavior on pain modulation, please write them in the box below.11 responses

No

I think that other factors influencing pain experience should be reported (e.g. sleep, stress...). Regarding the pictures, I do not think that illustrating the foot injury you used to explain acute pain is a good idea. The text below the figure is not clear (are the text affirmations or questions for the patients?)

-

Give additional information about maladpative beliefs acquired by patients about pain due to bad information provided by many healthcare practitioners

The pictures are clear, but more work need to be done on translation especially for patients

are the more important traditional elements of importance? religous is good

Nil

The slide is clear.

Pain can make people to abnormal things. eg Someone can become more vicious

None

Central sensitization


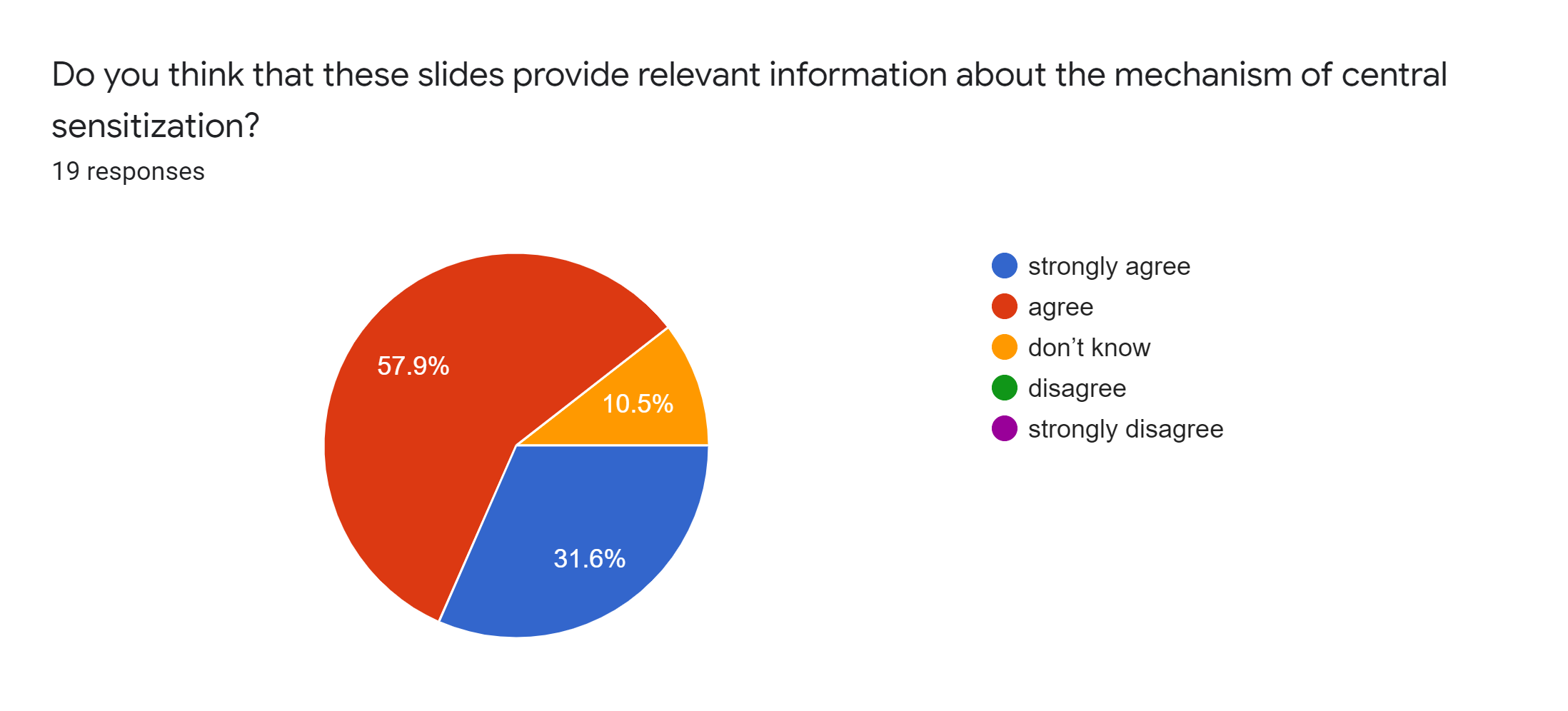


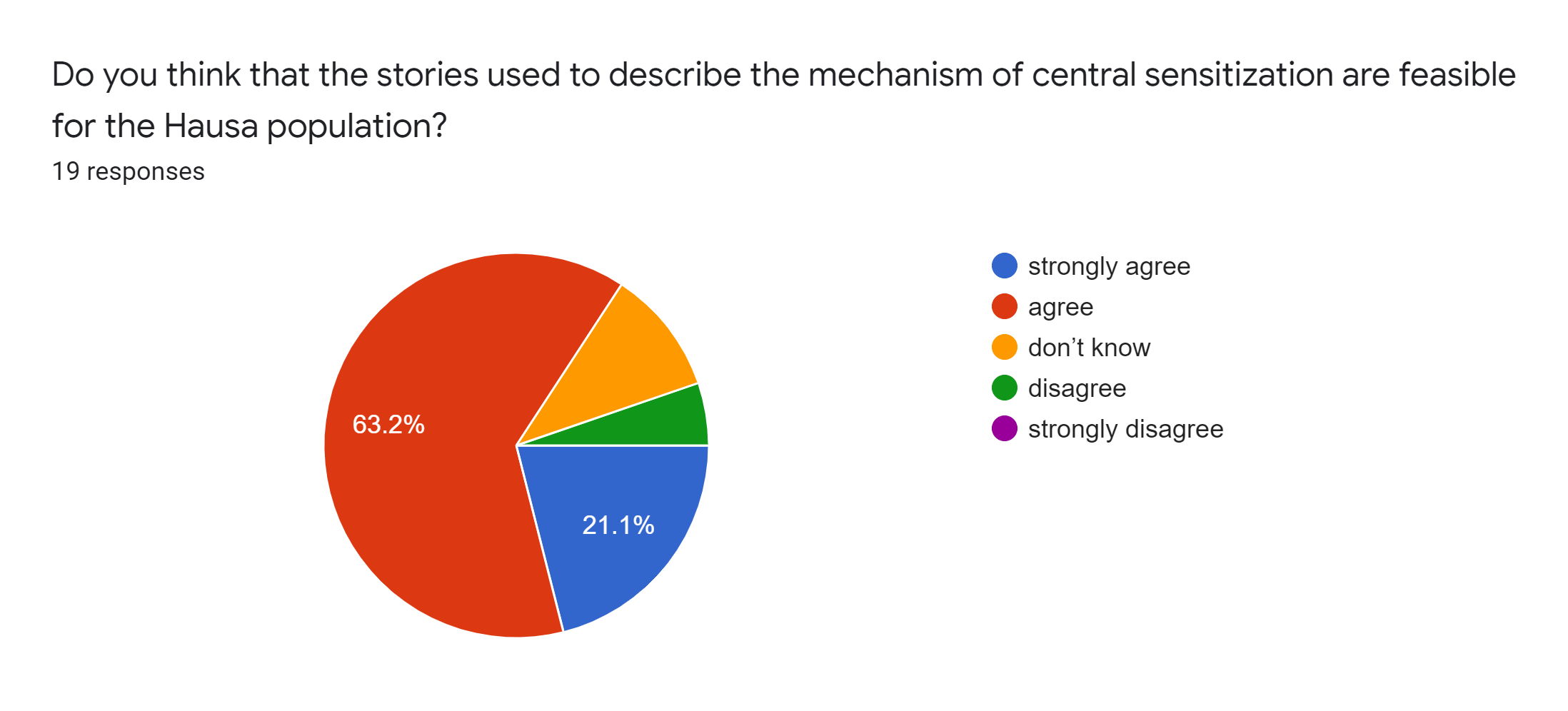


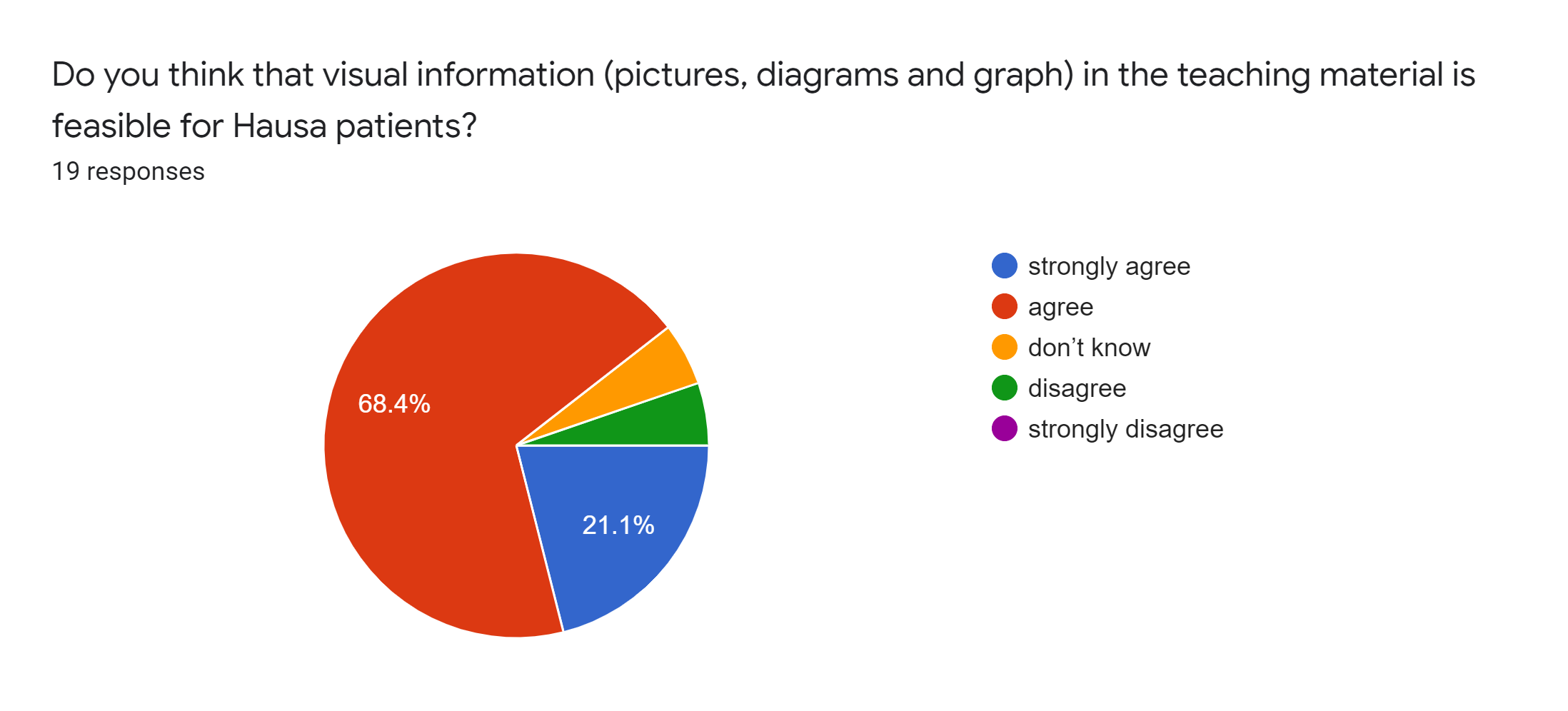


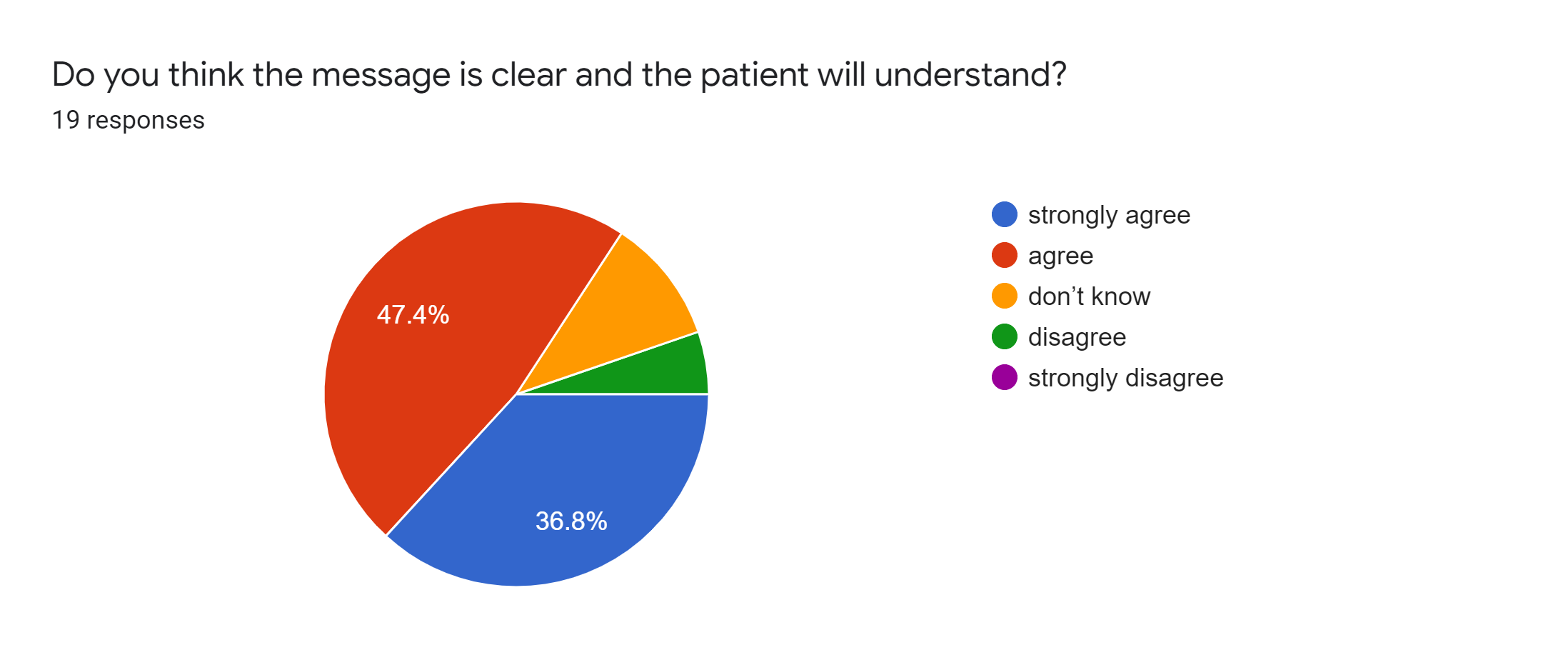


If you have any suggestions regarding the mechanism of central sensitization, please write them in the box below.10 responses

None

Slide 30: not sure the people will understand the figure. SLide 32: you state: with chronic pain, the brain becomes too sensitive. However, regarding patients with chronic low back pain, most of the patients do not suffer from a "central sensitization"...

-

Simple description should me made as much as possible because many Hausa patients are low literate

As explain earlier

Nil

Not every patients will understand the graph.

No

What has been written is enough

Implications


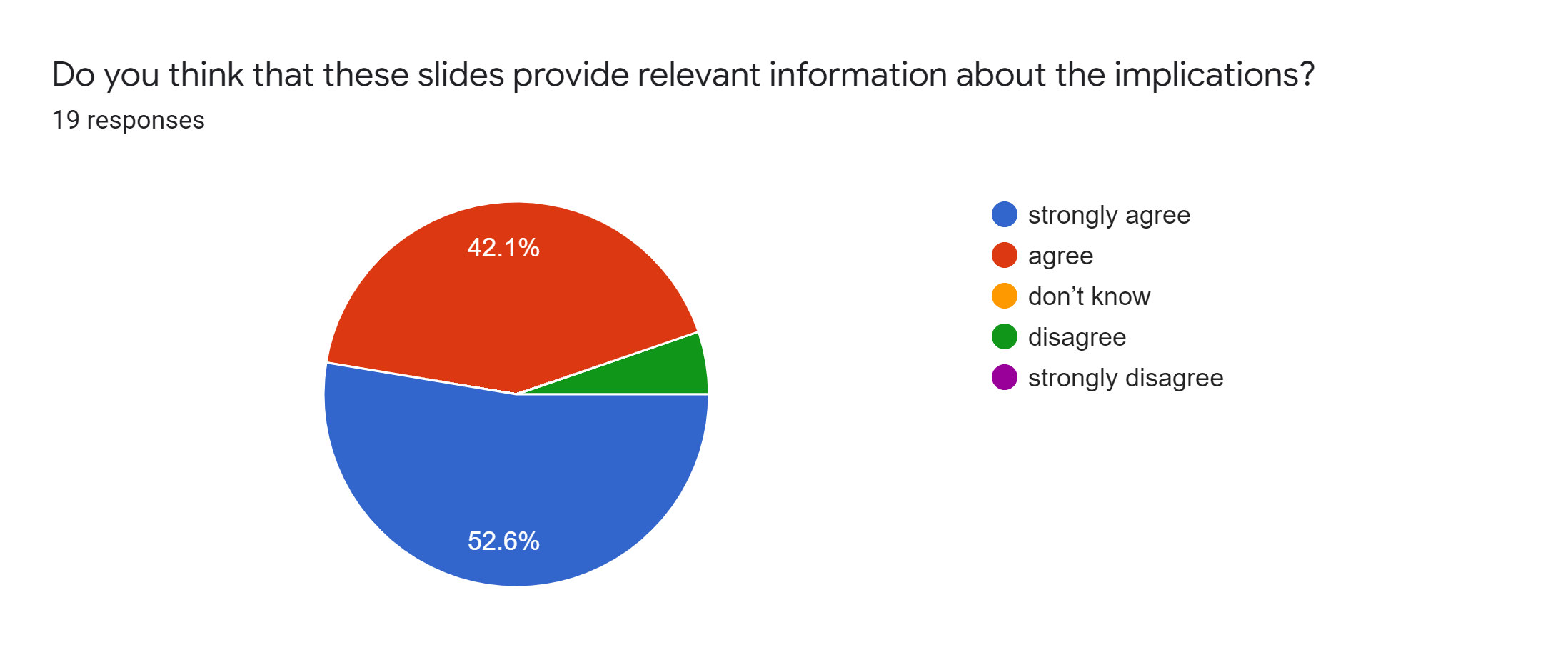


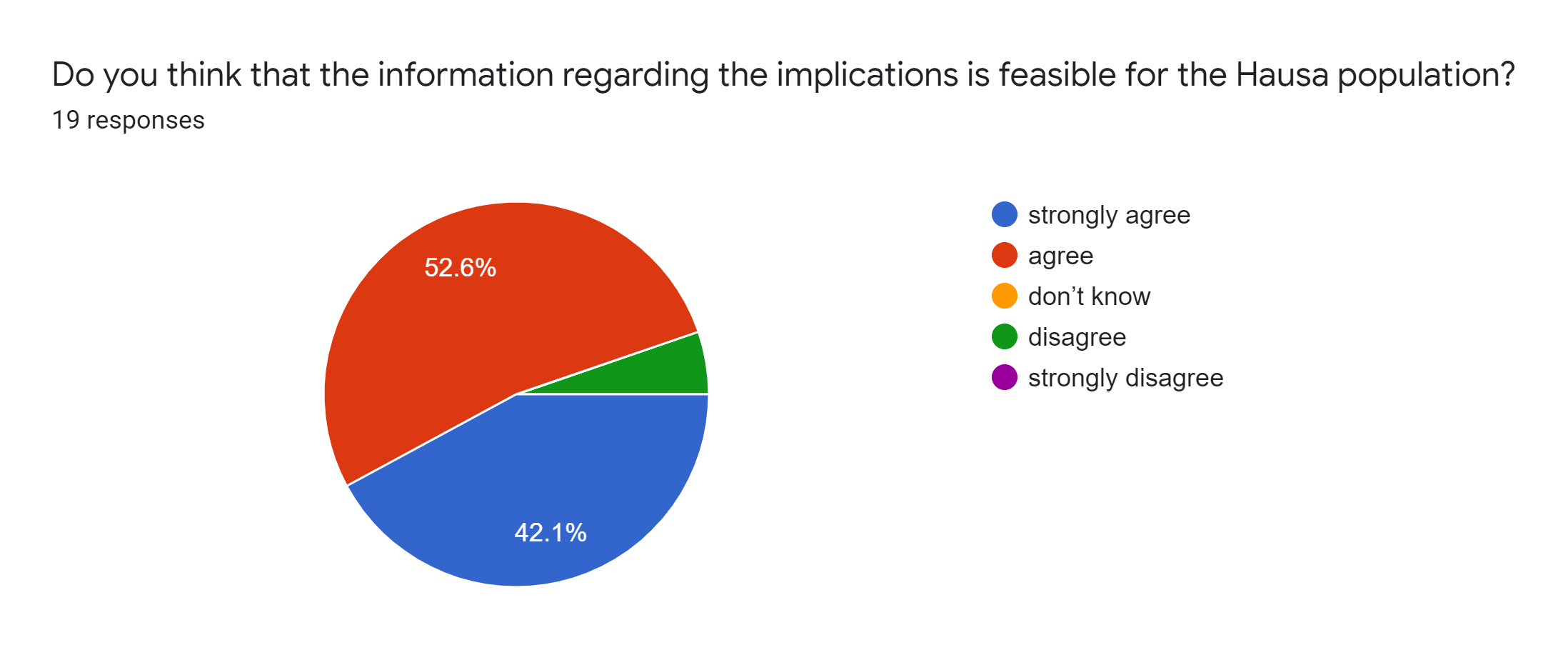


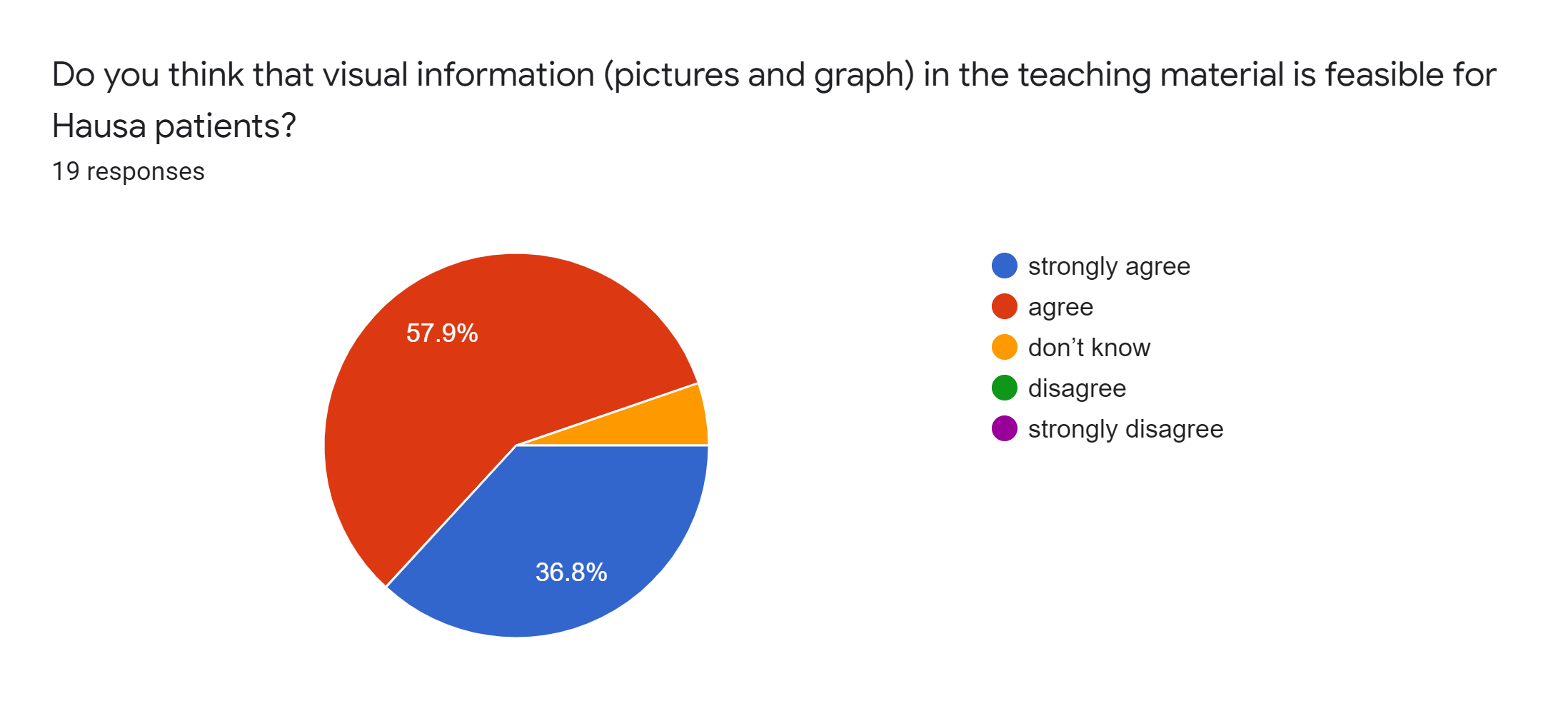


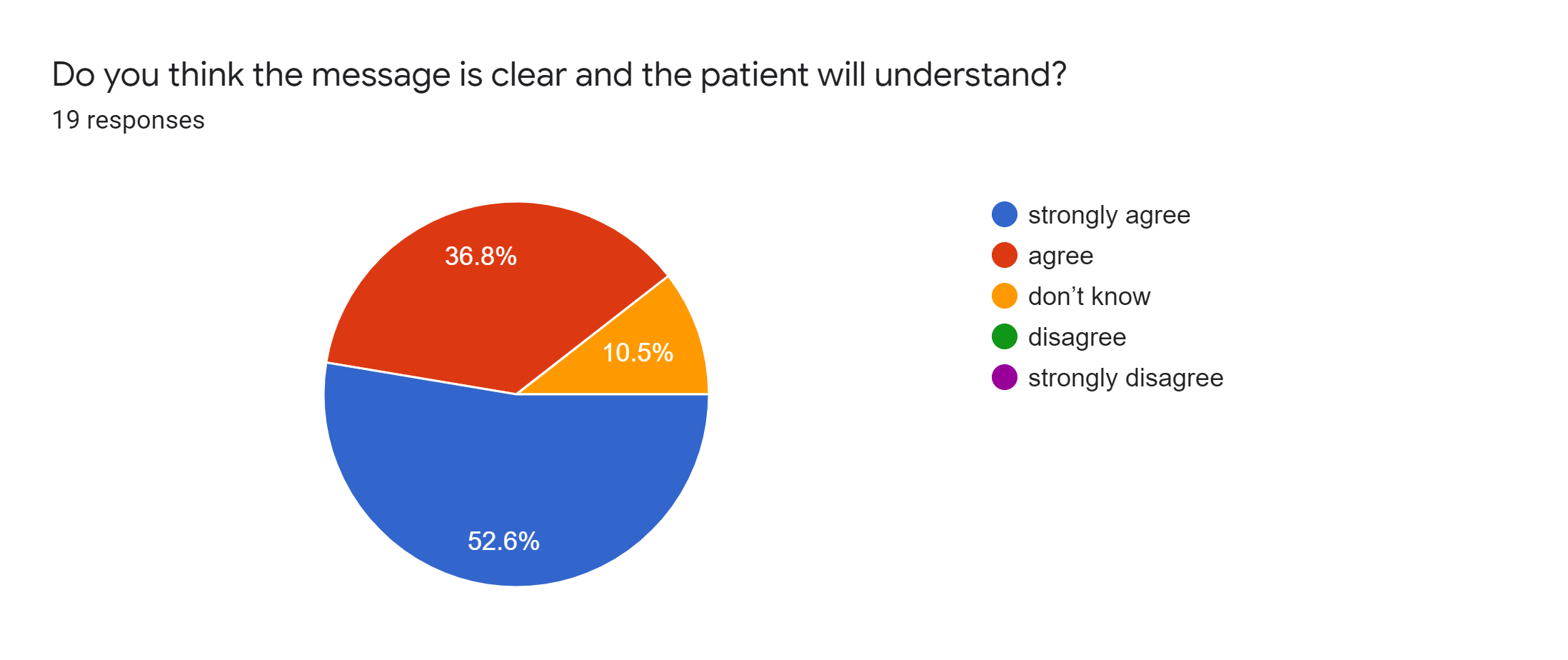


If you have any suggestions regarding the implications, please write them in the box below.

9 responses

None

Slide 33: can you really state that the one who has his leg broken will logically have more pain than the one with the toe injury.... it depends on the context as you stated earlier. The second sentence is not clear. Slide 34: you state "do not stay inactive". Do you state that because you think that in this population people become inactive as soon as they have pain ?

-

Too general recommendations are provided. Give additional information about strategies for management of chronic pain

more maybe pray? talk to your family or grandfather, be in contact with nature (?) stop taking bad medication etc!

Nil

More examples may be provided

GENERAL QUESTIONS

1. What do you think about the order and the concept? Is it understandable, logical?

19 responses

Understandable

I think that the content would need to be improved

well, it needs some subheadings I believe , for the reader to be more clearly you're shifting from acute - chronic - adaptaions - implications

Yes orderly and sequential

Yes, it follows an standard PNE teaching session

Yes, it is very logical

It's understandable and logical

The order is well arrange

agree although I would be very interested first to know the cultural context which you shoul incorporate more

Both understandable and logical

Yes it is

It is understandable , it explains all in detail.

It is both logical and understandable

Yes it is both logical and understandable

It is very meaningful and logical

Yes, it's really understandable

It is very understandable

It is understandable and logical

2. General remarks?18 responses

None

I'm sensing it might still be building upon previous knowledge: so it might be still to difficult for lay people

Will be helpful to patients

The program is well organised and provide all the necessary information on pain neuroscience education.

Interesting and informative

But you need to work on the translation of some concept

nice pictures!

A feasible project and relevant to the population of interest

The flow of the questions, is good for understanding to both clinician and patient

This will help a lot of people understand when to visit a physician.

1.The pictures are self understandable even without explantion. 2.The additional of explanations or interpretation make it clean and clear that every pt at all level of education capacity will understand.

More explanations and examples may be provided

Simple and educative

An excellent approach to explain both acute and chronic pain in Hausa

The slides are well packaged

very educative and interesting about pain education

I wish you success in your research

3. Further suggestions?16 responses

None

see above

I would advice the researcher to have a component where by the participants will be allowed to tell thier stories about the experience of pain. Using this information would be useful in addressing any unhelful beliefs and thought about pain. Secondly, is gud to have an assessment questionnaire like the Hausa version of Revised Neurophysiology Pain Questionnaire (NPQ) which can be used to assess participants' understanding of biological process of pain (pain conceptualization).

The translator and expert need to sit down and agree on some words

succes

Nil

I suggest more images should be used, as some people understand better through looking at images than listening to explanations.

Some slides needs additional or more explanations e.g. slide 16, 19, 20, 28 and 30.

Other forms of pain (eg extremities) could be incorporated

More and more of visual images while explaining in the future

Slides should be produced in the form of a pamphlet or book format

The questionnaire need to be cross culturally adapted into different languages

There is need to provide more educational resources for patients

This questionnaire should have also been translated to Hausa language
