# Supplementary material for: Development of culturally sensitive pain neuroscience education materials for Hausa-speaking patients with chronic spinal pain: A modified Delphi study
Source: PLoS One. 2021 Jul 2;16(7):e0253757. doi: 10.1371/journal.pone.0253757 (PMC8253446; doi:10.1371/journal.pone.0253757)
Supplement: S2 Data — (DOCX) [file pone.0253757.s008.docx]

Data for Delphi round 2 (A and B)

Questionnaire link for 2A: <https://docs.google.com/forms/d/e/1FAIpQLSd9XPSyQEhaeDveQy1jT6qYaCezOLfkfh8WQfiQtwVa-at65Q/viewform?usp=sf_link>

Responses:


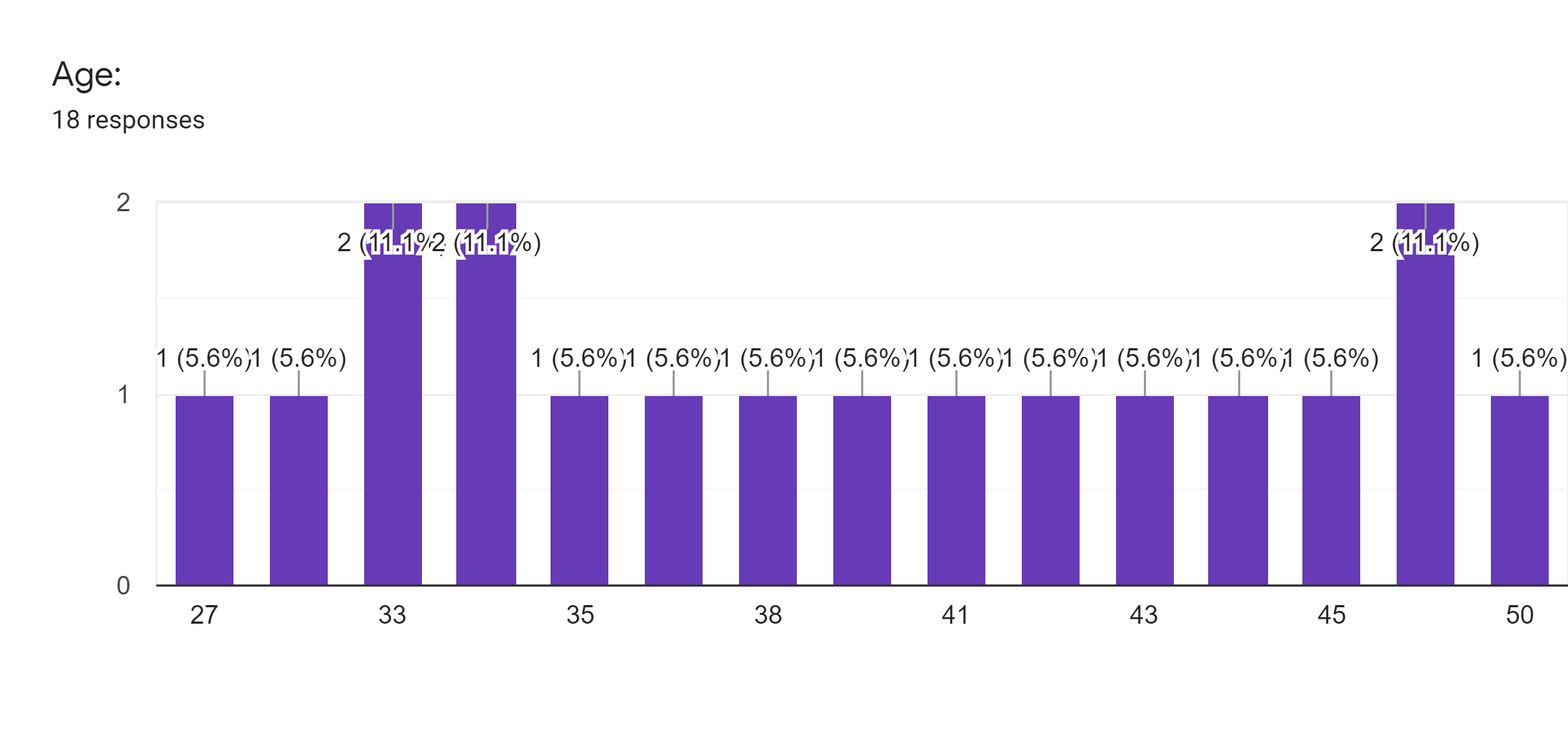


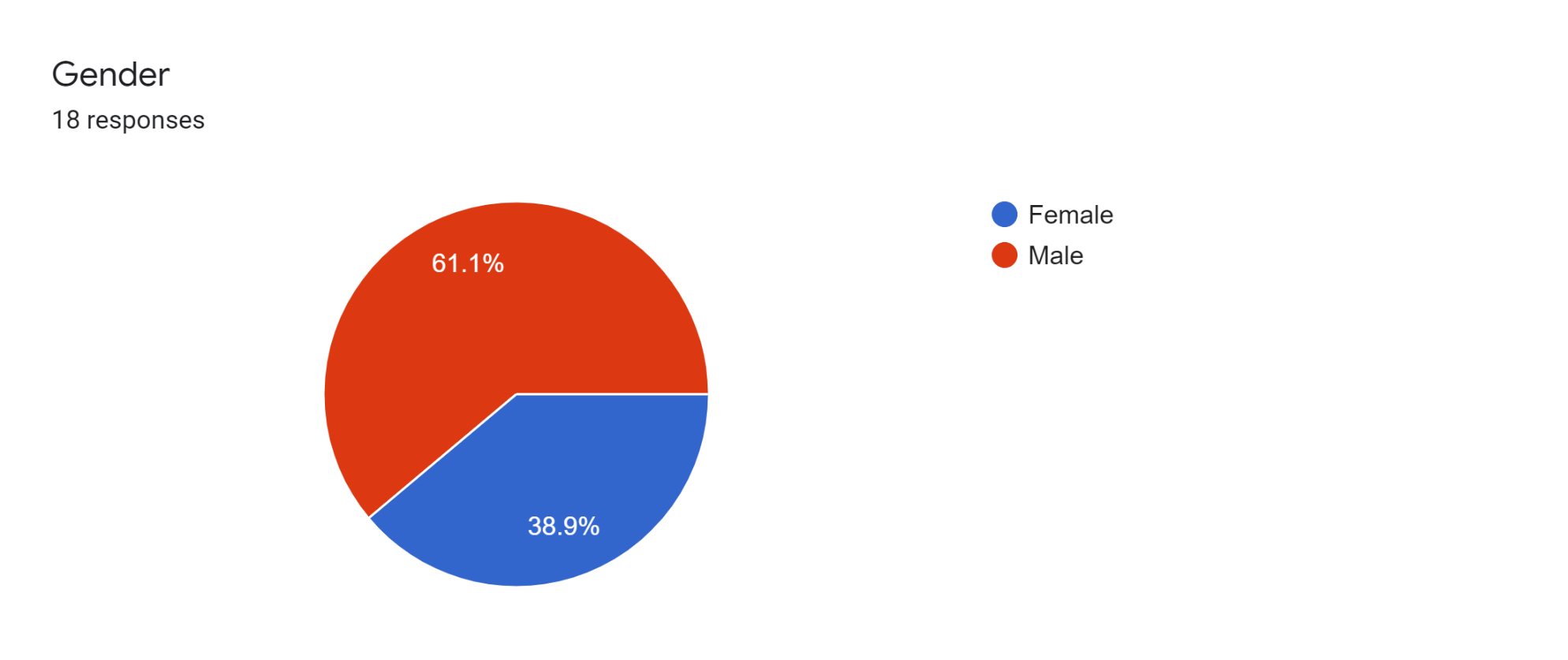


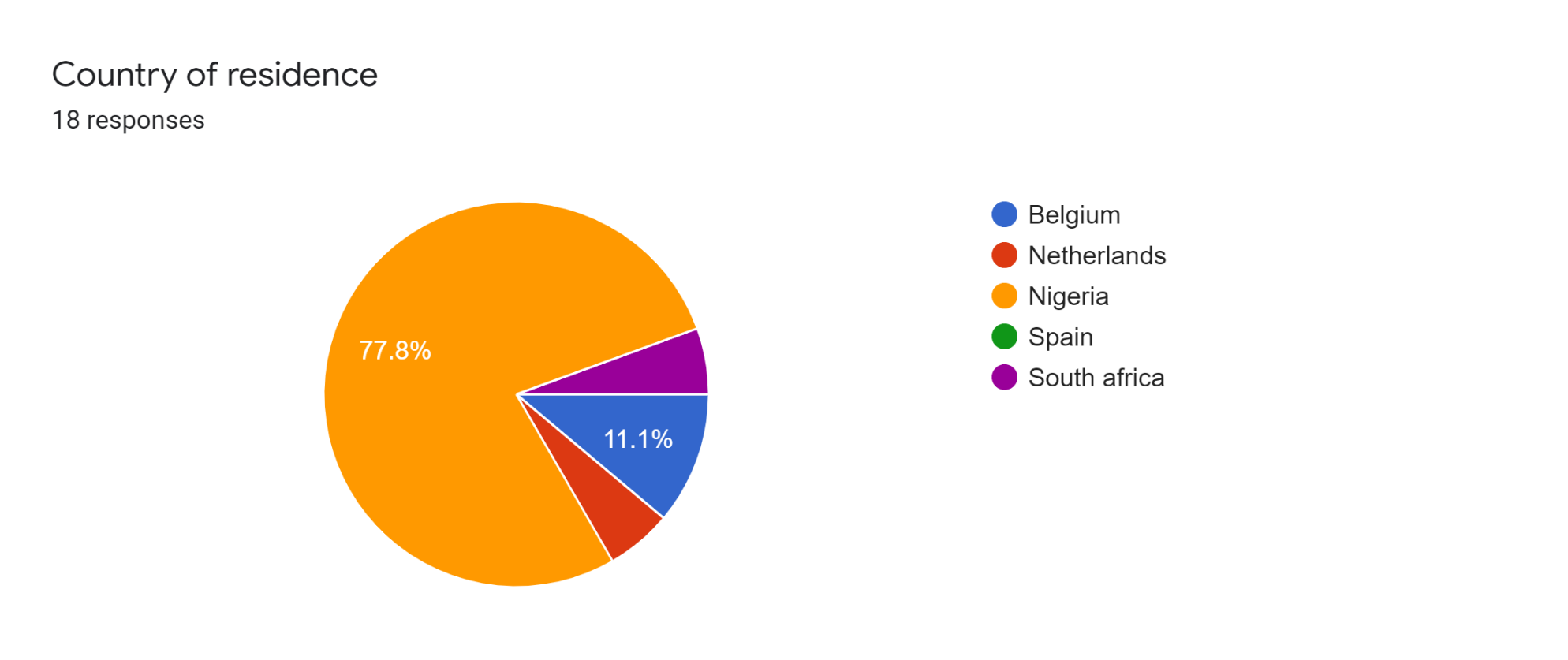


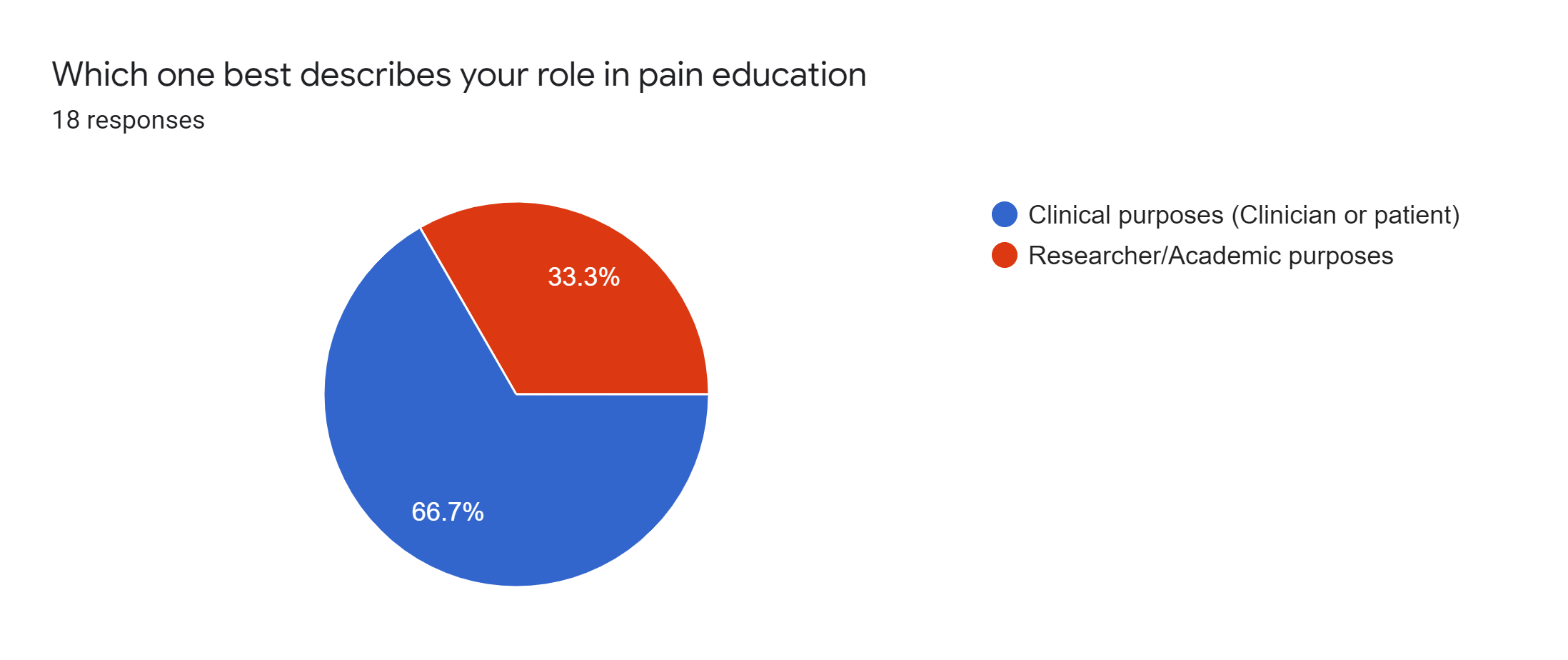


What is your experience with pain education?

18 responses

Non-existent 11.1%

Heard of it 5.6%

Familiar with it, <1 year 16.7%

Familiar with it, 1-5 years 33.3%

Familiar with it, 6-10 years 22.2%

Familiar with it, 11+ years 11.1%


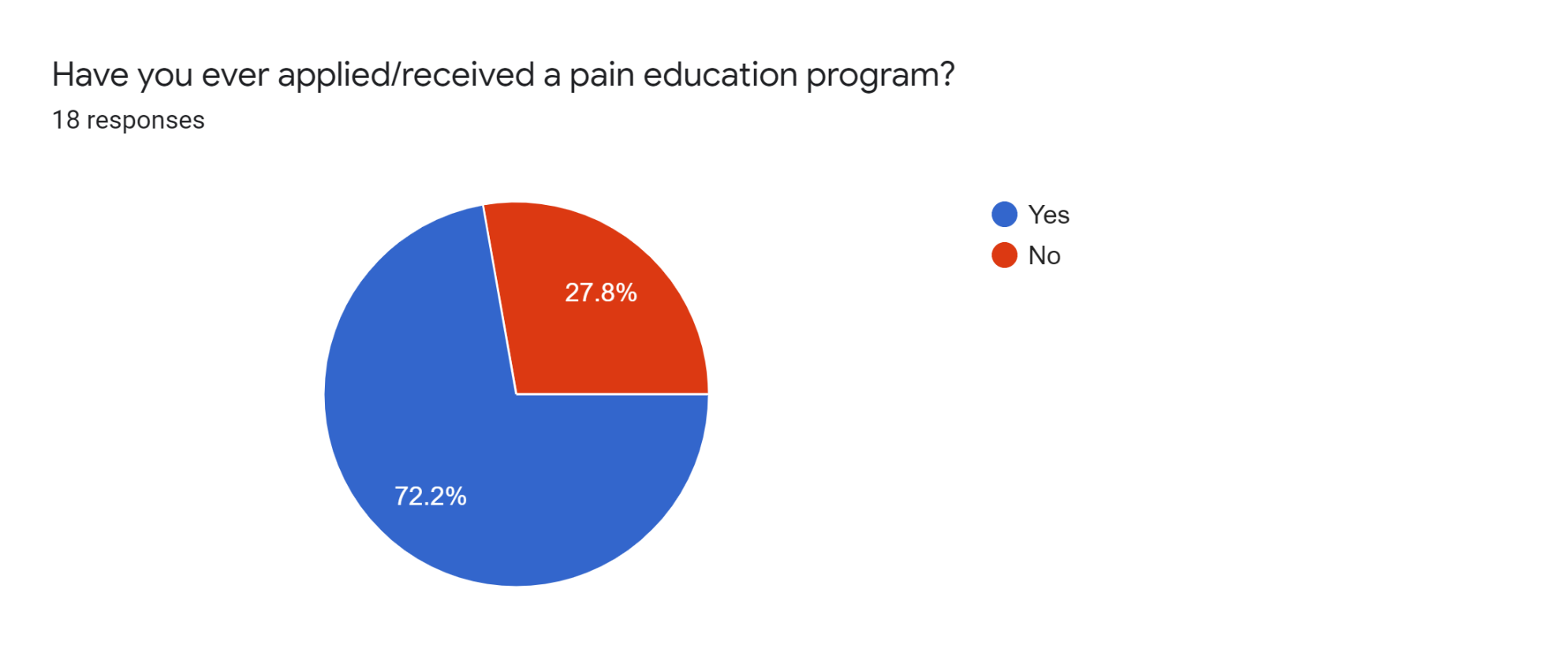


Acute Pain


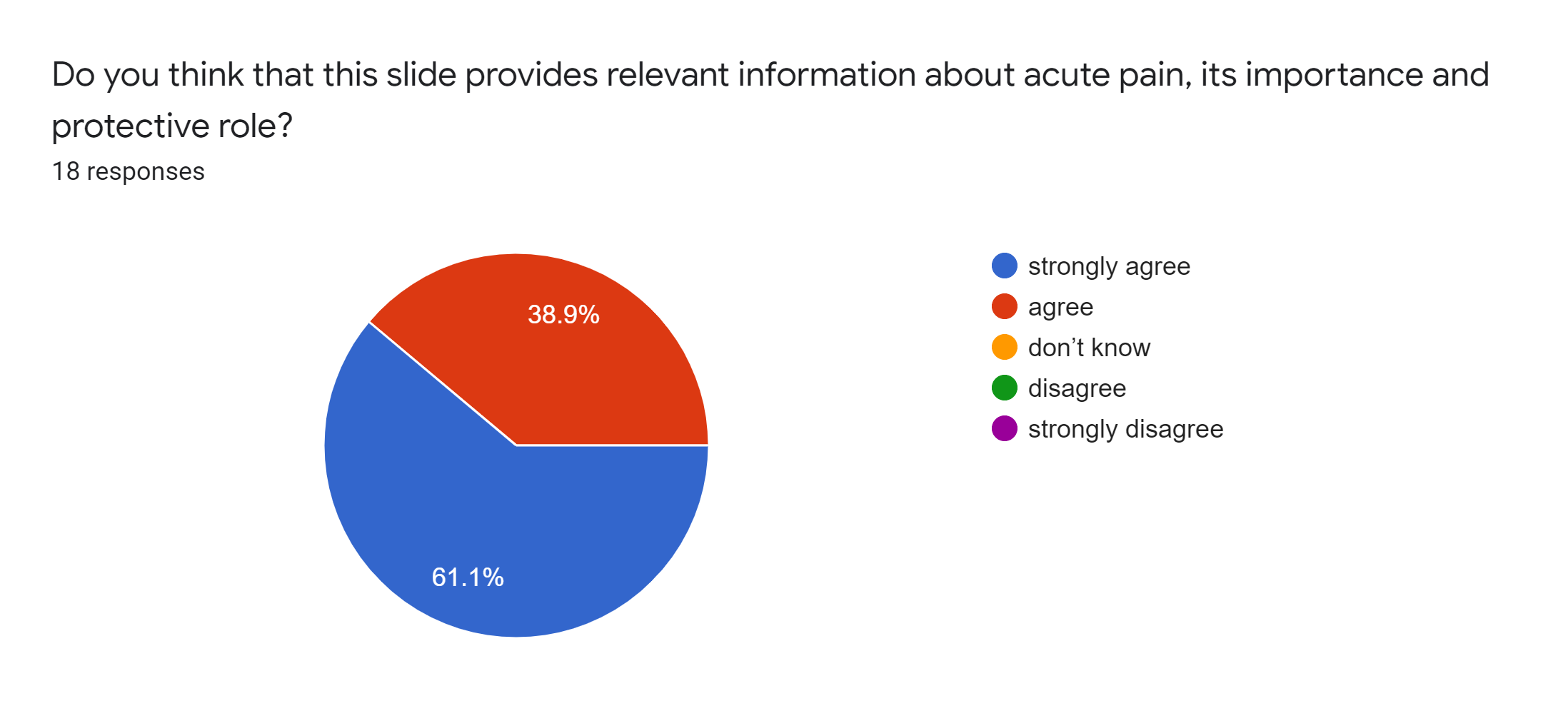


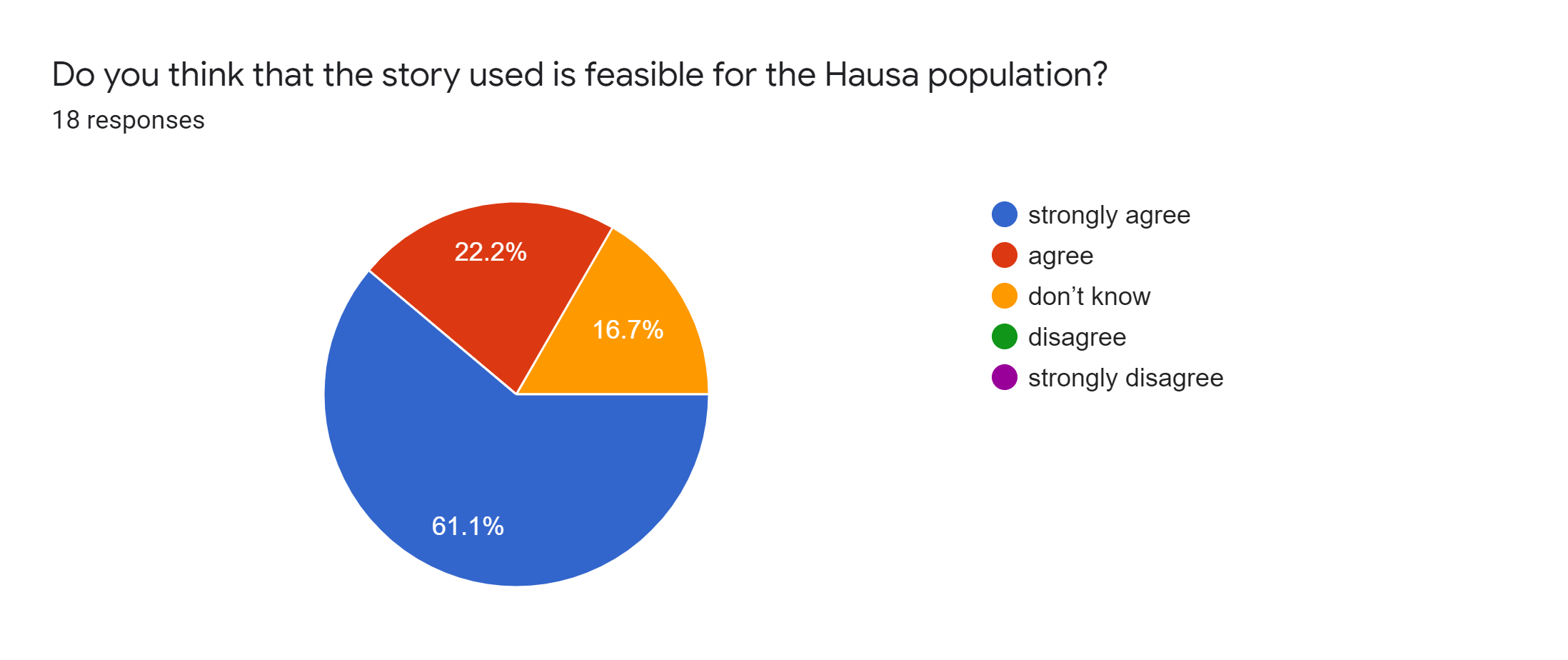


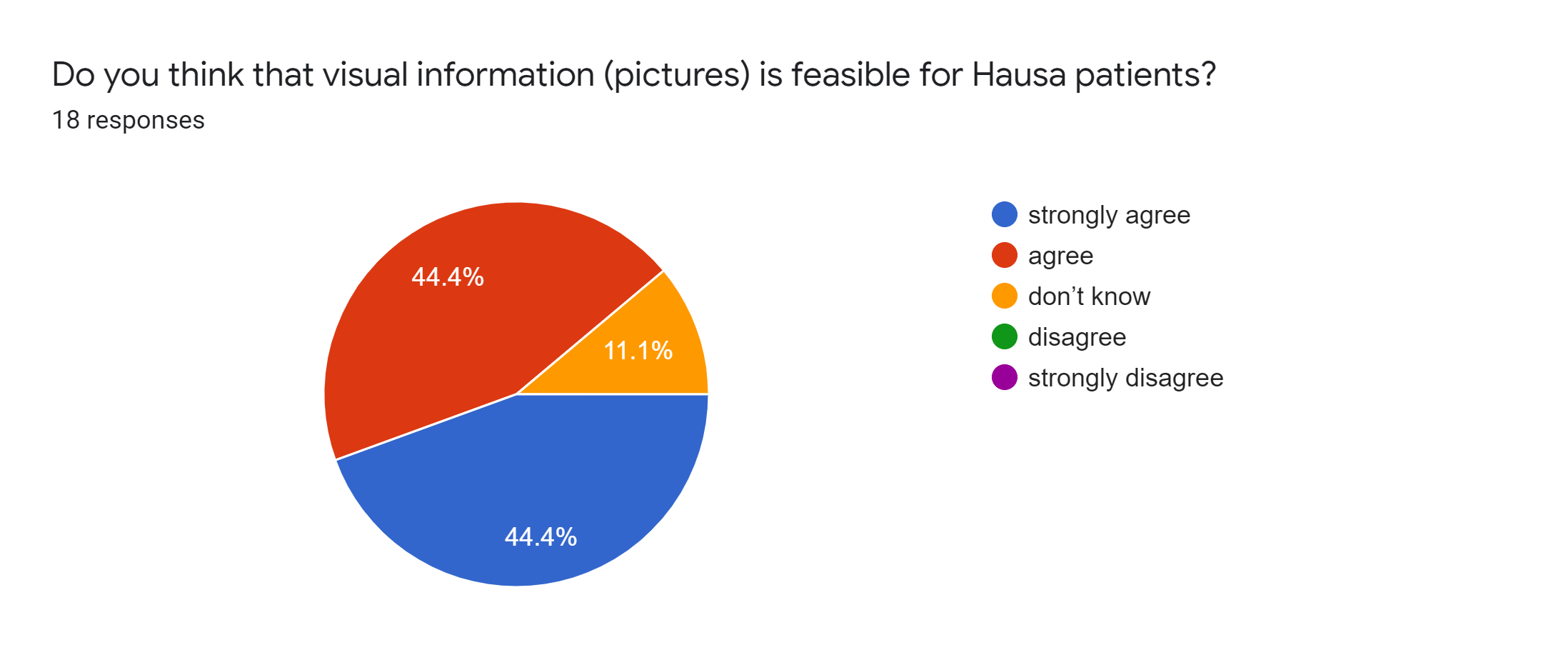


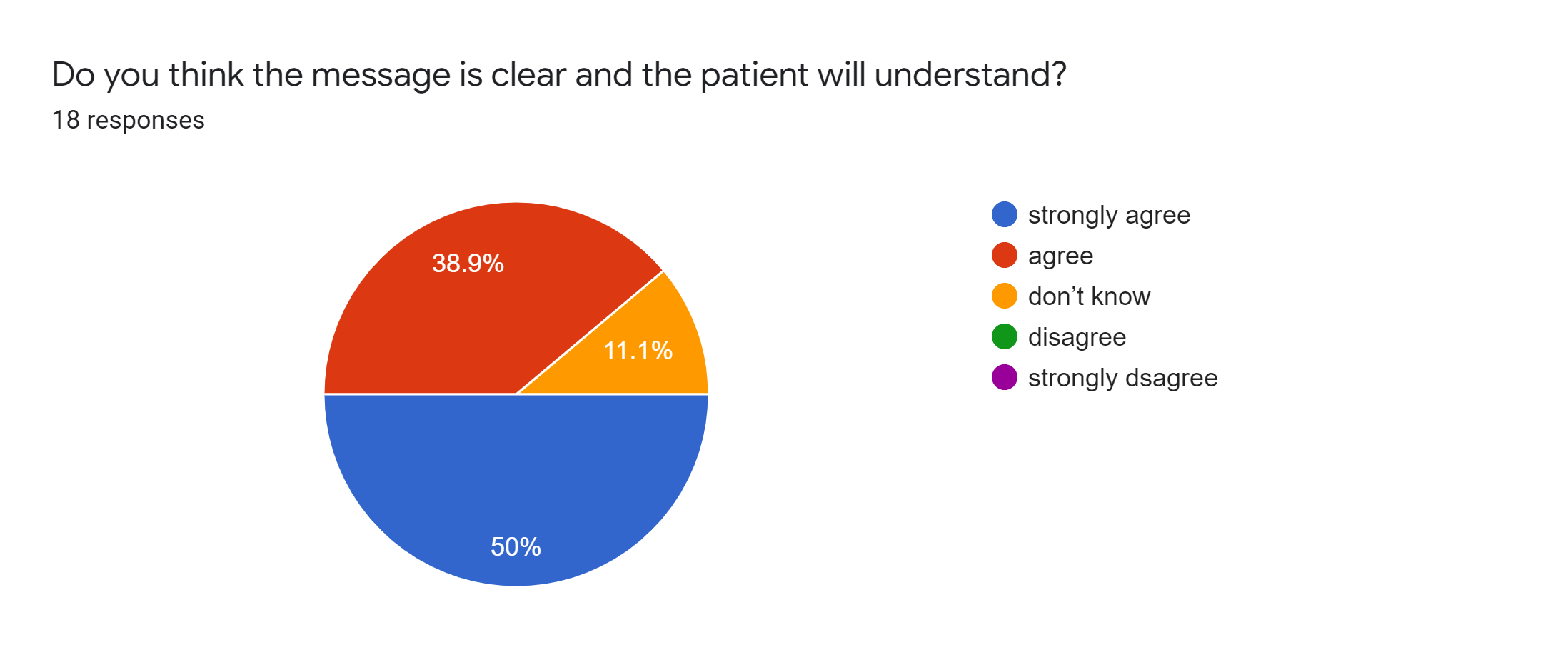


If you have any suggestion(s) regarding the description of acute pain, please write them in the box below.9 responses

I dont know if the picture slides could be of animation..so that they could be played in various areas such as hospitals and other healthcare facilities.i think it could me more interesting.

No any suggestions

Some patients believe in traditional medication

none

NO

sorry my knowledge of the cultural context is not well enough to give more specific answers

Nil

I am not sure about the explanation that pain alerts the body of potential danger. I would say that pain alerts the person to potential danger

Its apt

Pain biology


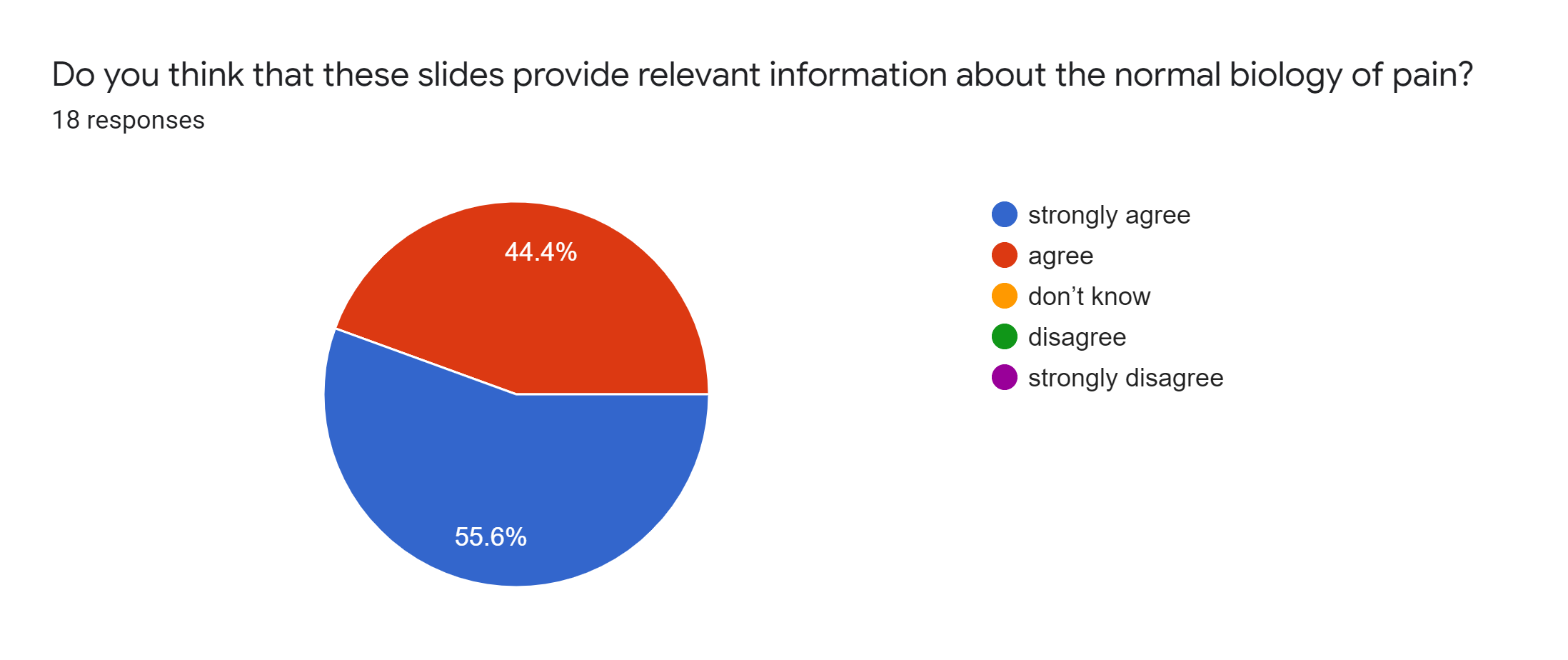


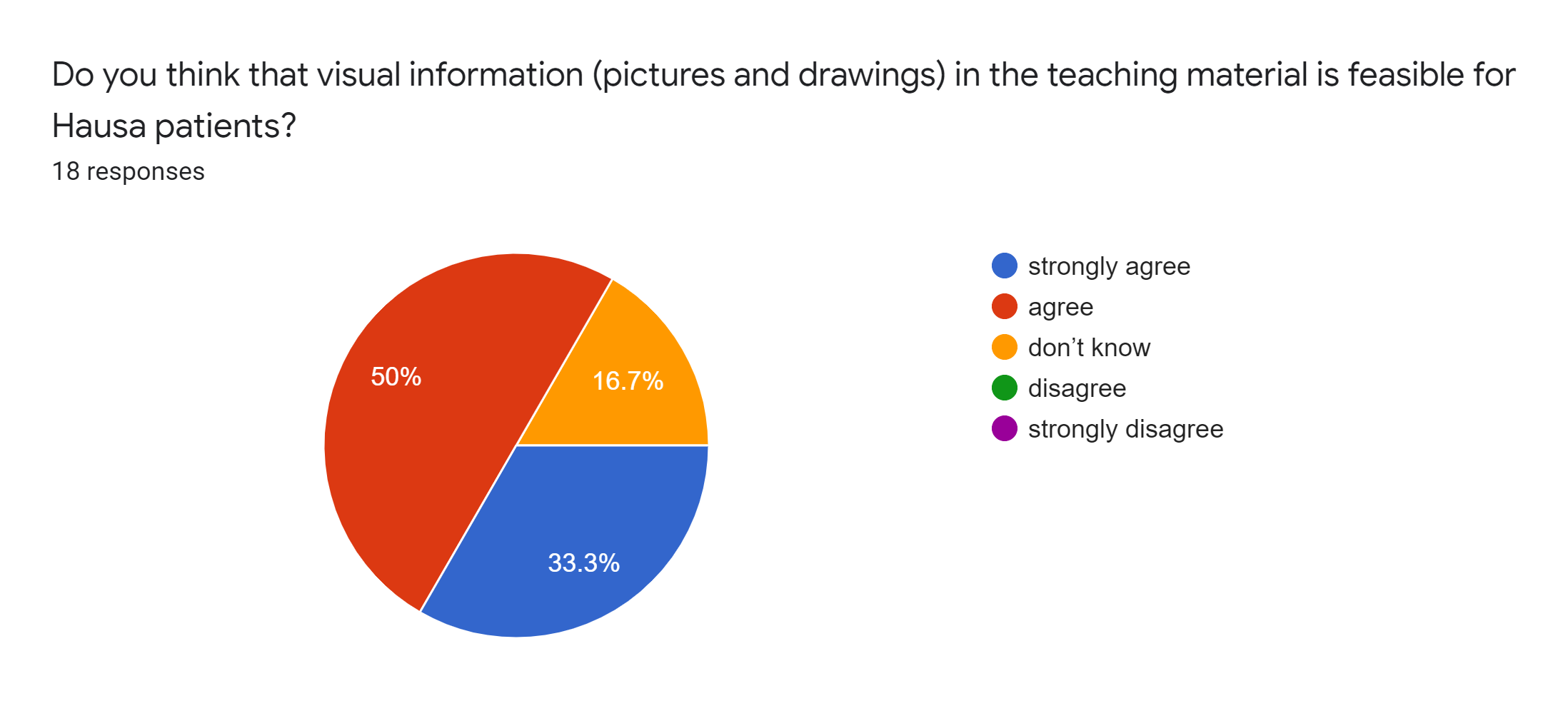


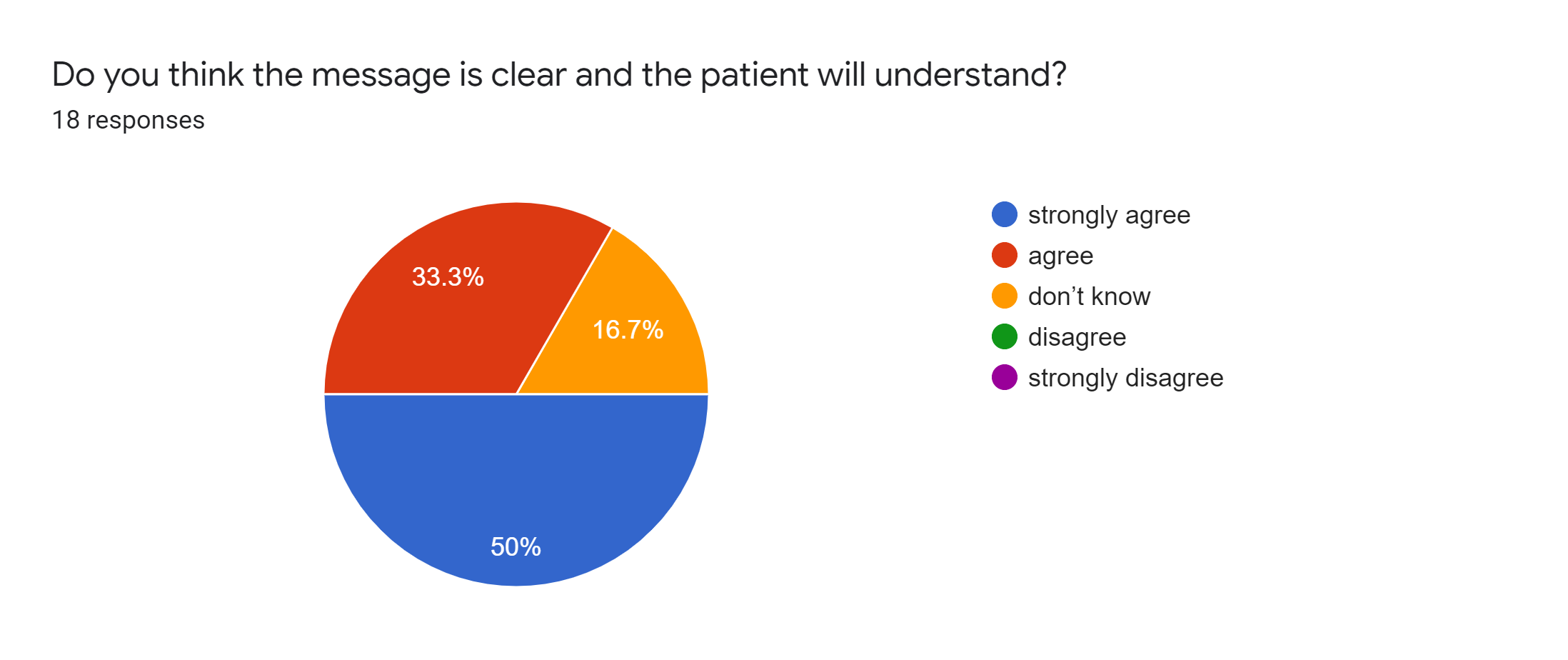


If you have any suggestions regarding the explanation of the normal biology of pain, please write them in the box below.9 responses

I think those drawings(neurons)may confuse some.i feel its better to give clearer pics so that lay men can understand too.

Everything is well explained.

E.g road accident and electric shock

no additoinal information

NO

I don't like the receptor drawing, like receptors are also black??

Pictures should be more of hausa

I believe the designs are much biased by paramedical investigators: don't whether the hausa population is familiar with receptors like this (same remark as last time)

Clear and precise

Pain Modulation


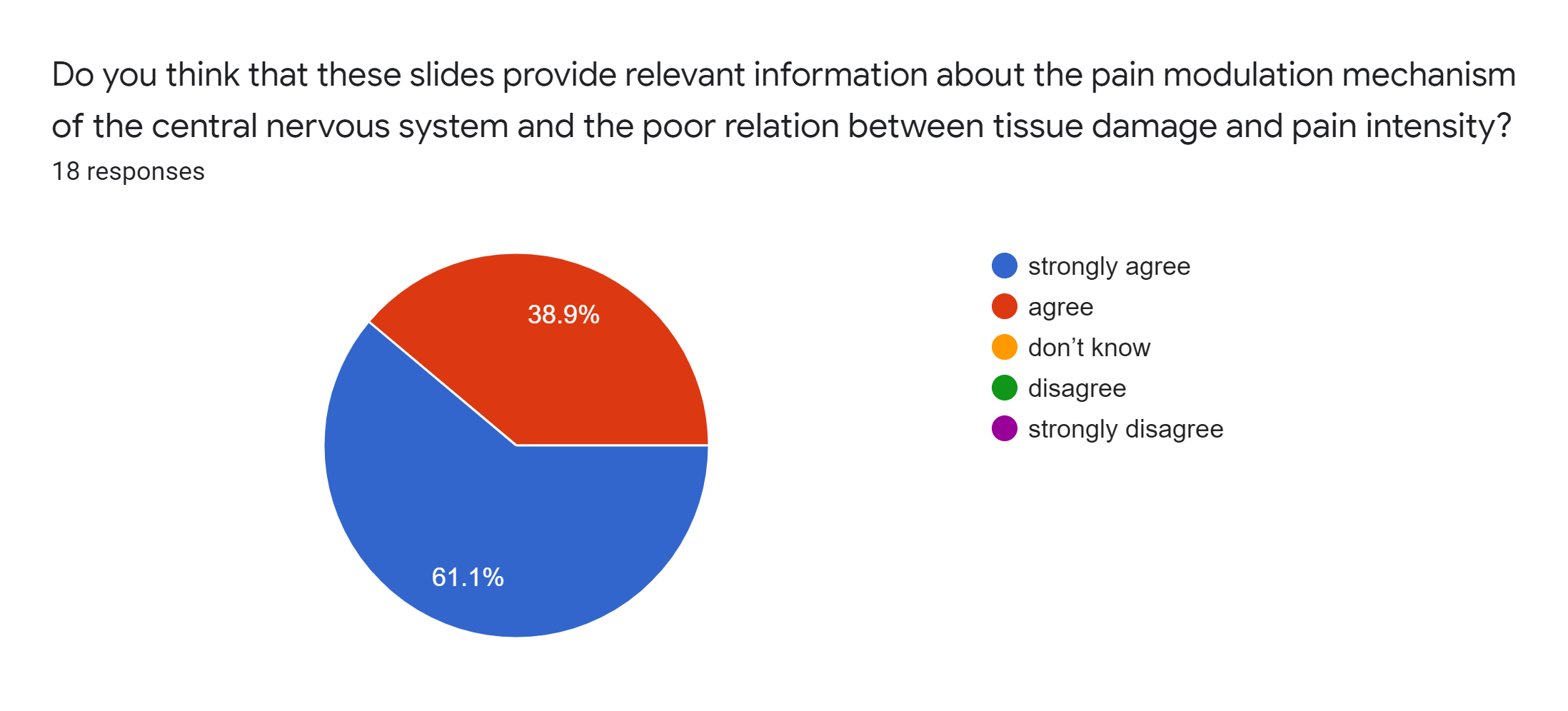


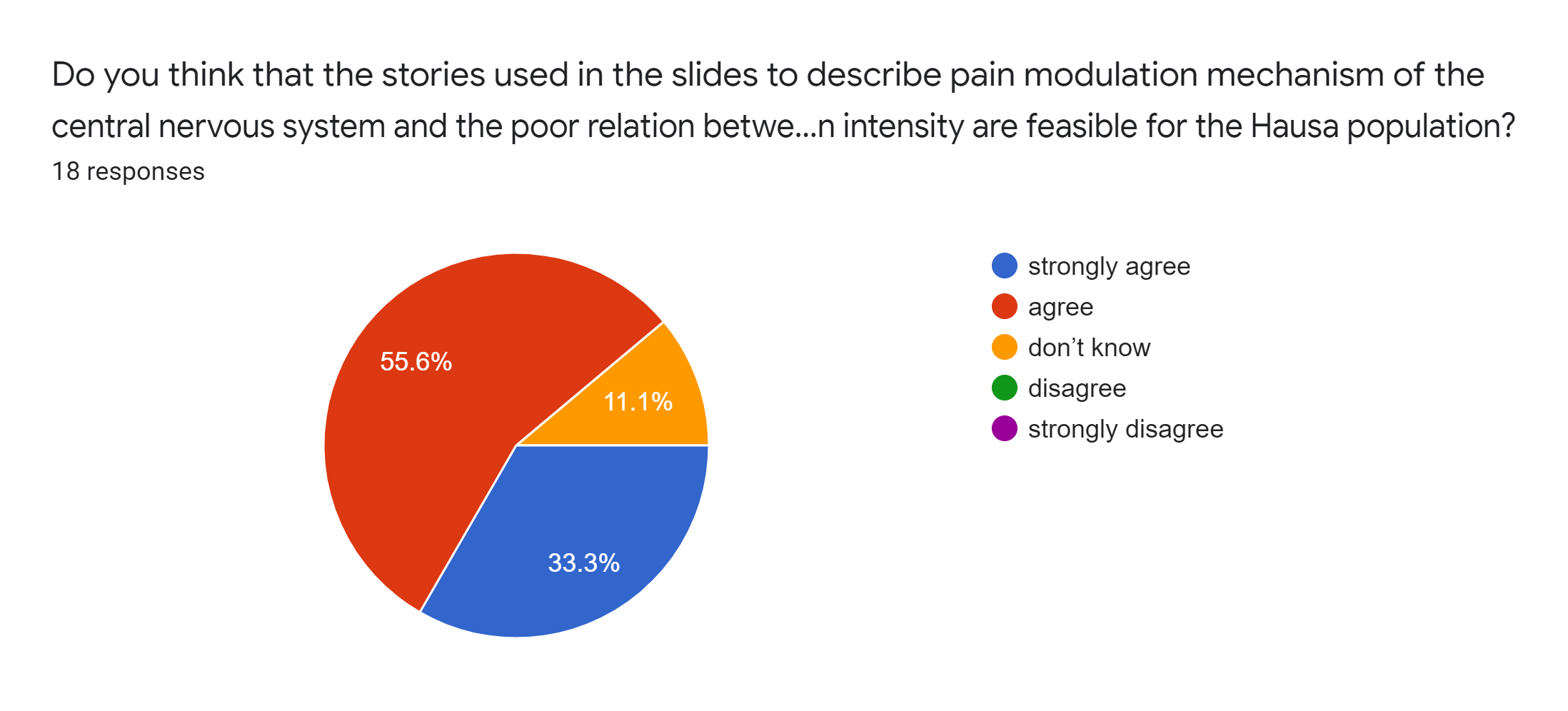


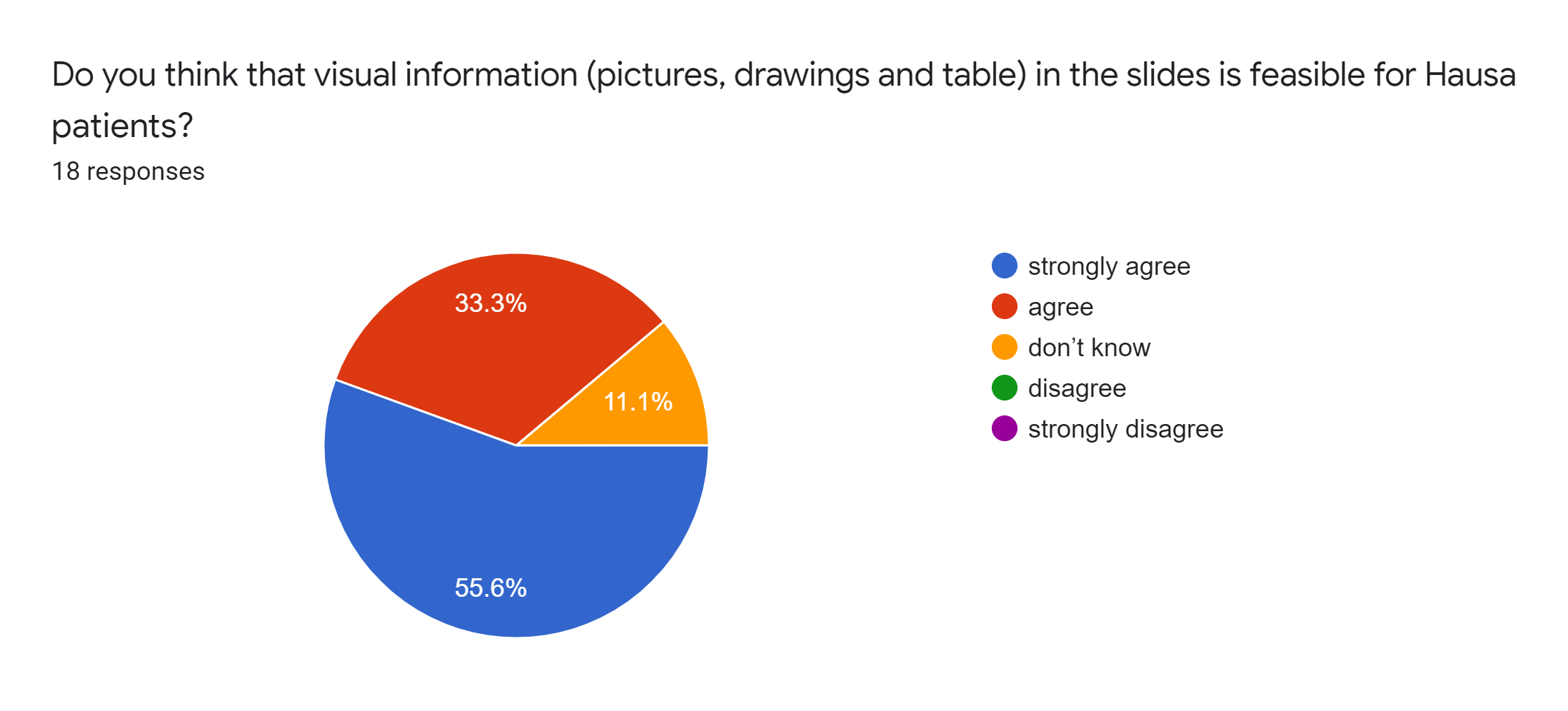


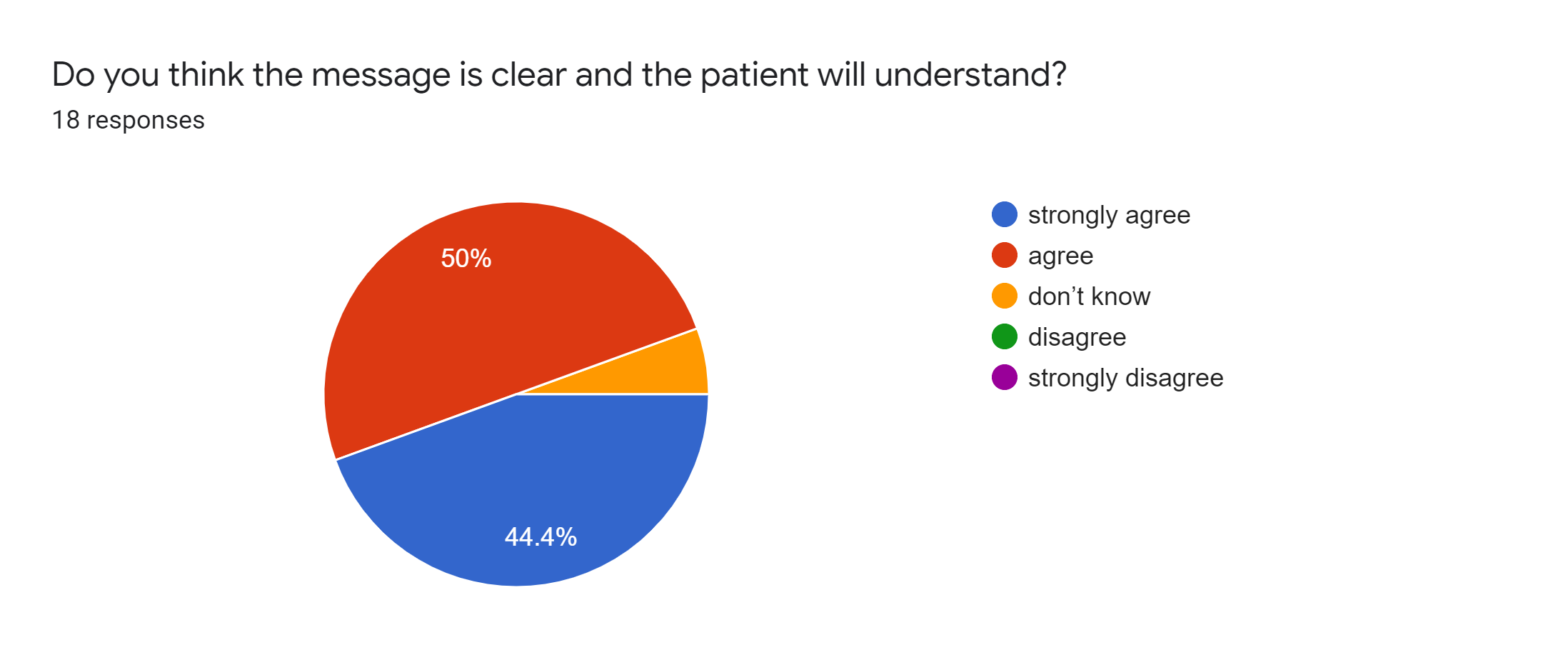


If you have any suggestions regarding the explanation of the pain modulation mechanism of the central nervous system and the poor relation between tissue damage and pain intensity in those slides, please write them in the box below.7 responses

Why not use few names in the stories instead of using various names?

Simplifying this relationship using clear and simpler term through provision of health talk

no

NO

shadi / sharo explain this is niciceptive pain, in the also pain thresholds are contextual driven

Nil

figure 18 I. Does a lay person understand disc degeneration ? I would make a drawing of é 75 year old's: 1 wirh pain, the other wirthout, but with the same lower back

Beliefs, thoughts and behaviours


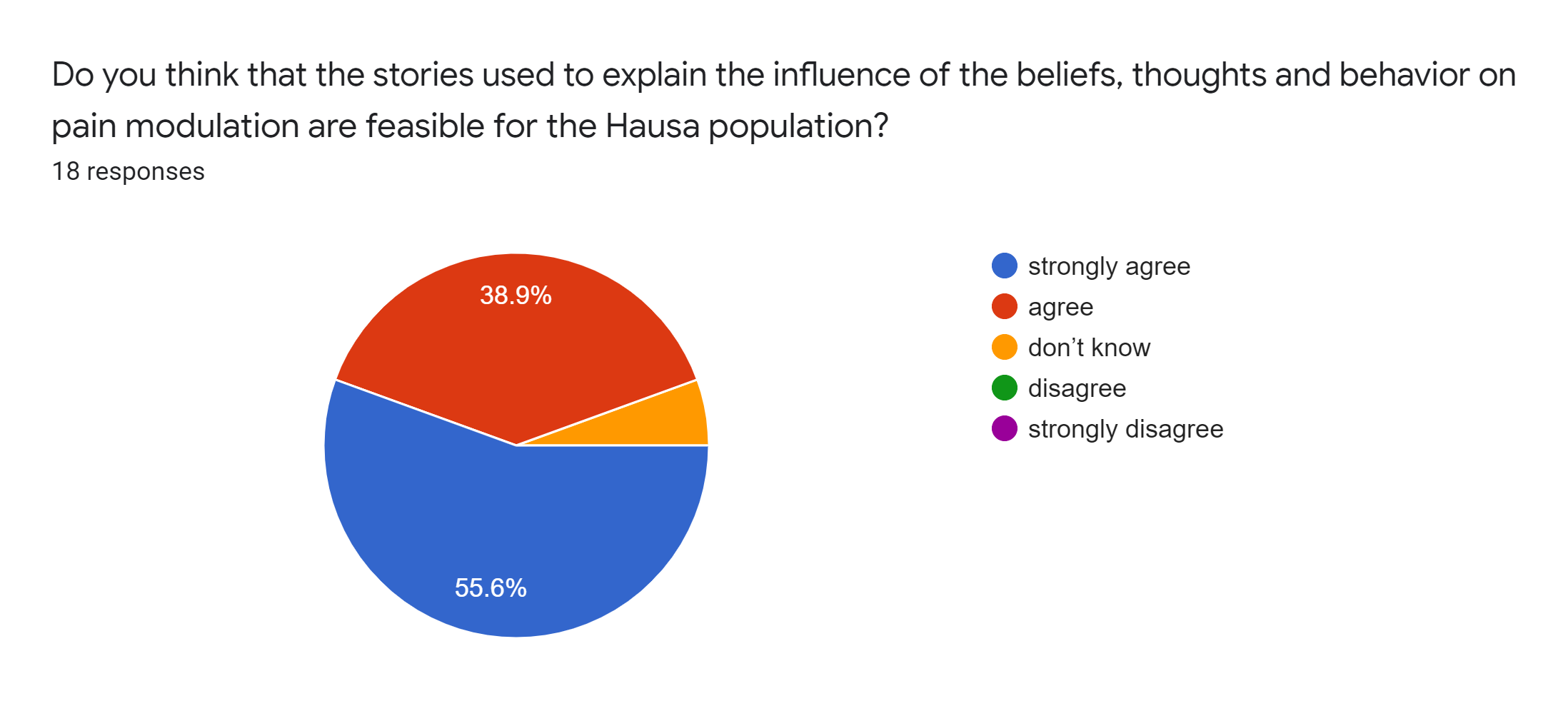


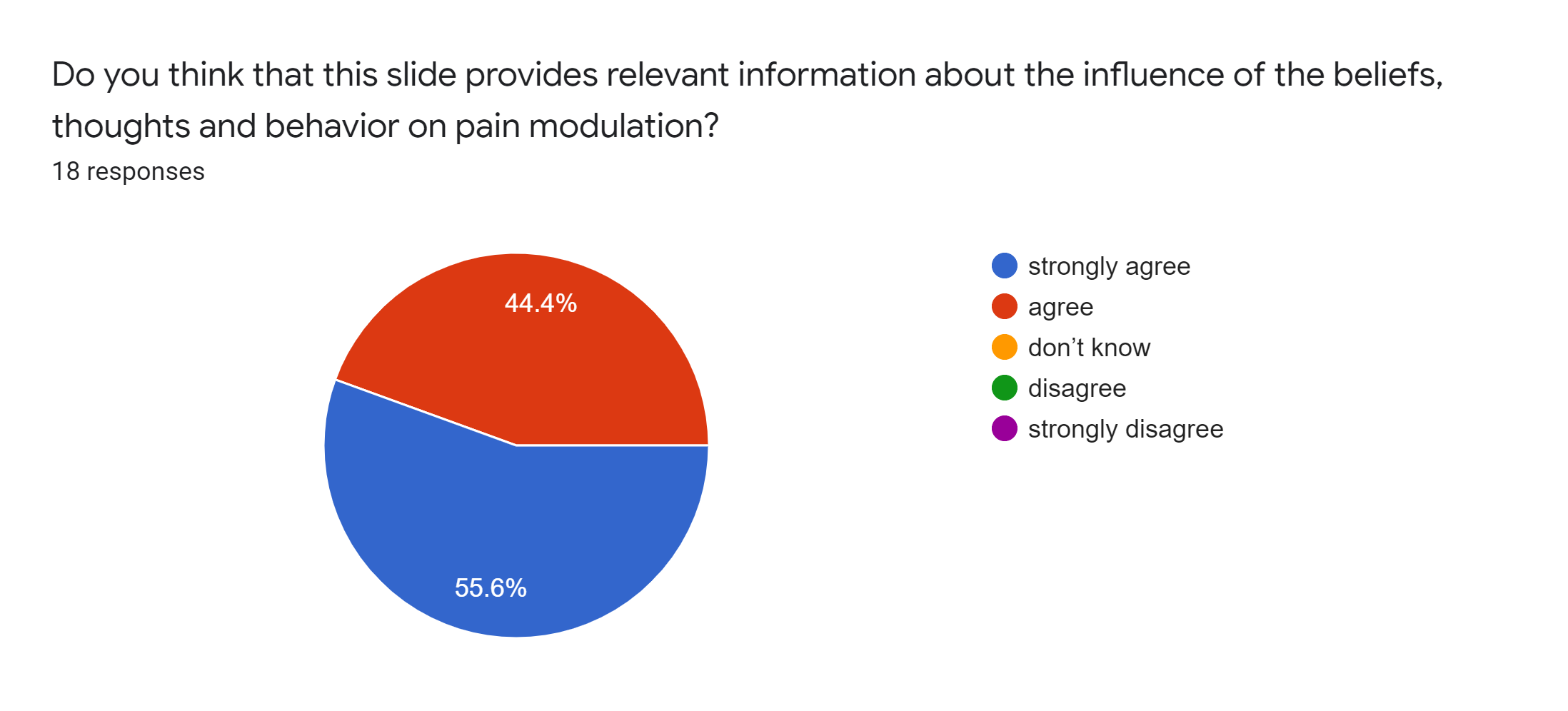


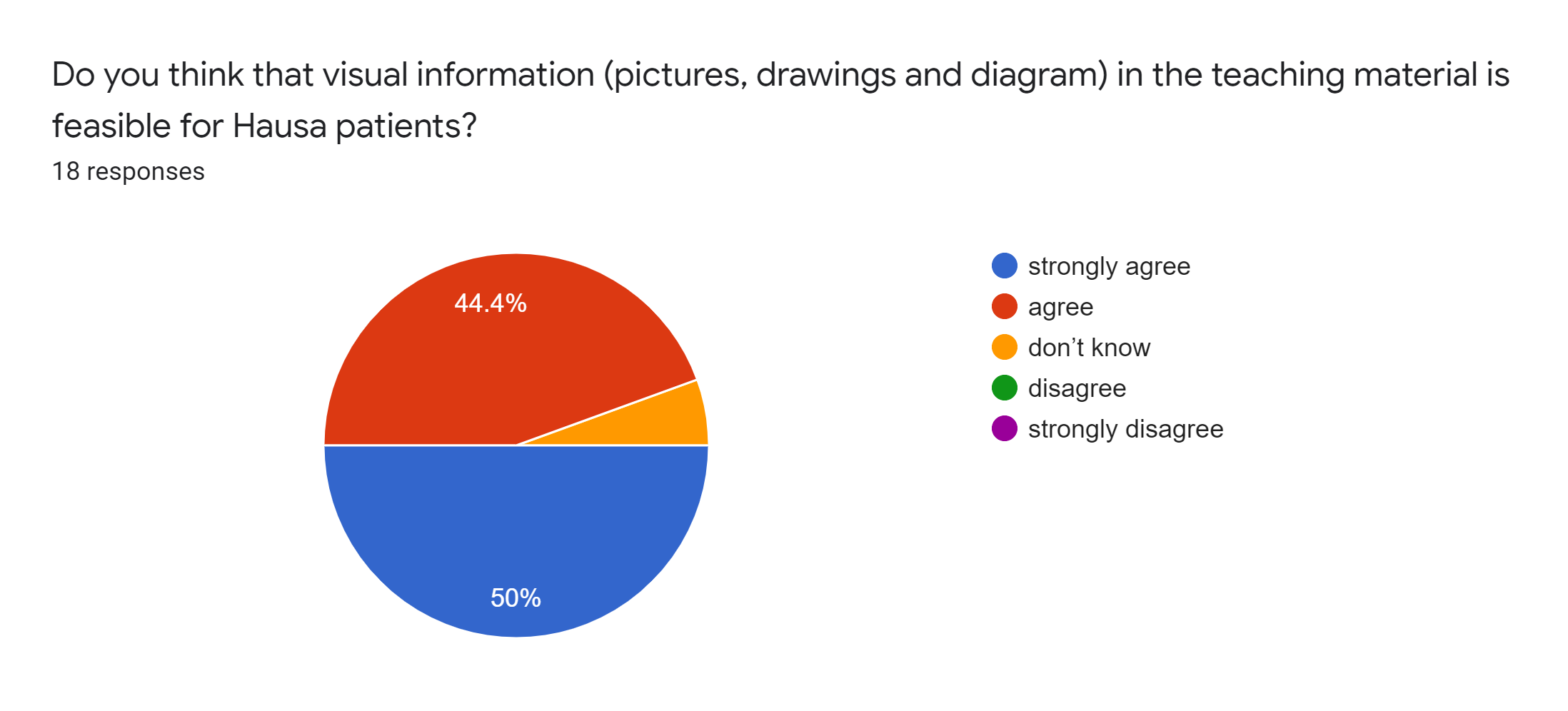


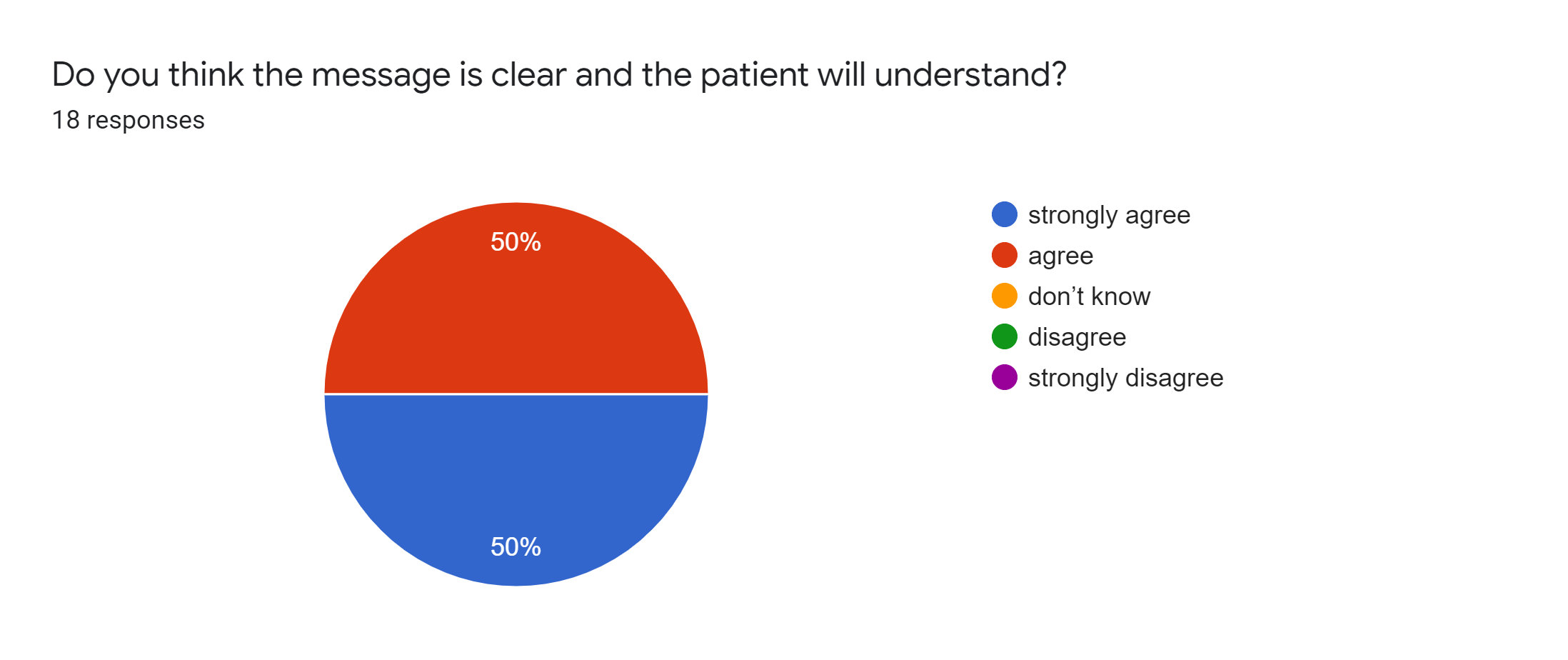


If you have any suggestions the influence of the beliefs, thoughts and behavior on pain modulation, please write them in the box below.

5 responses

No

None

NO

religious could also be mentioned as fear, fear for religious punishments etc

Pictures

Implications


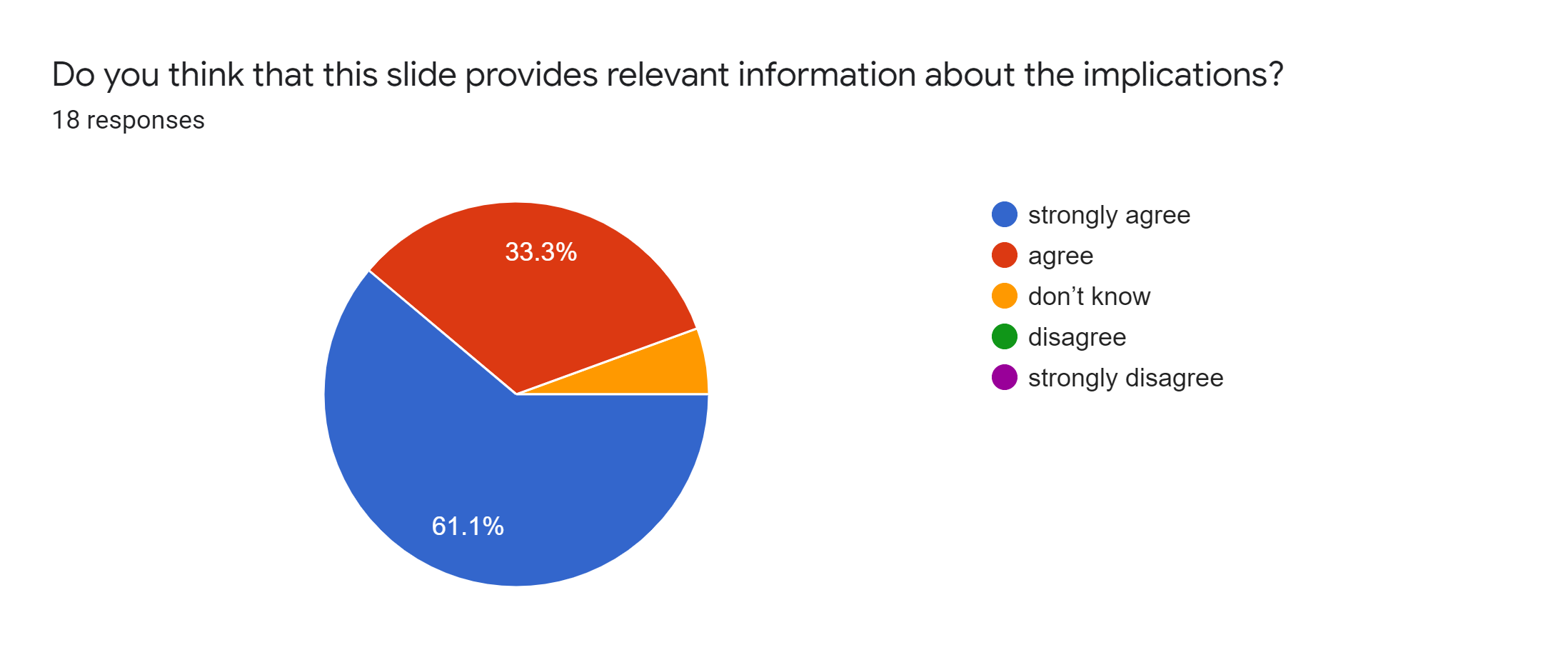


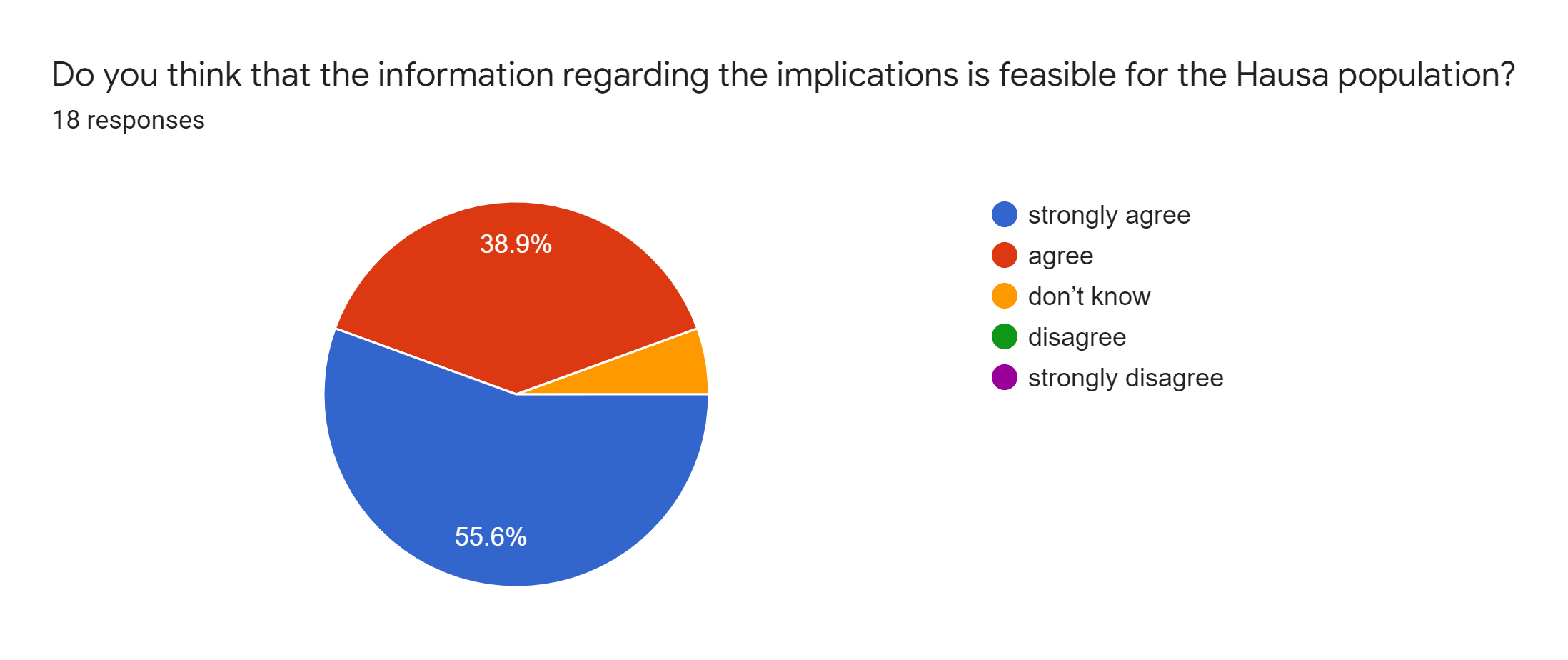


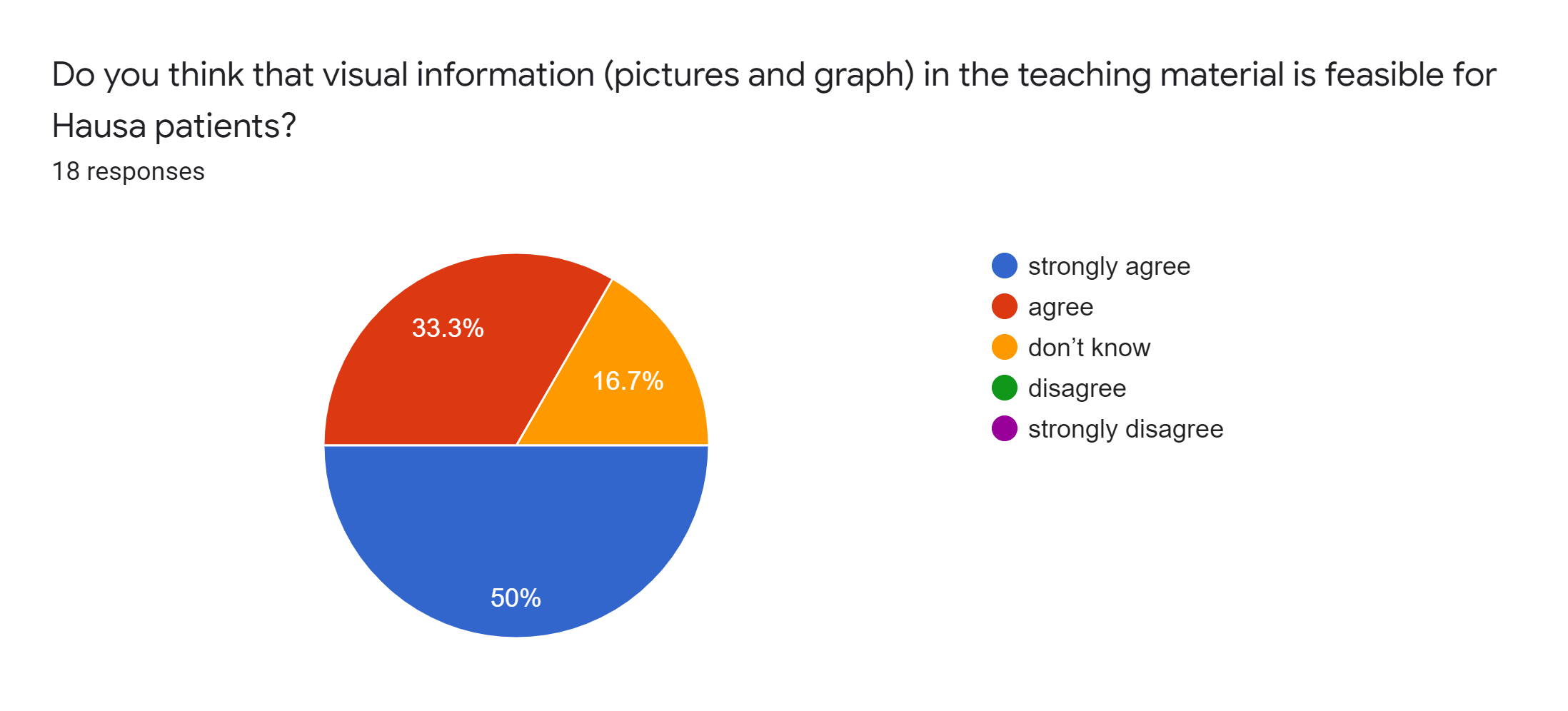


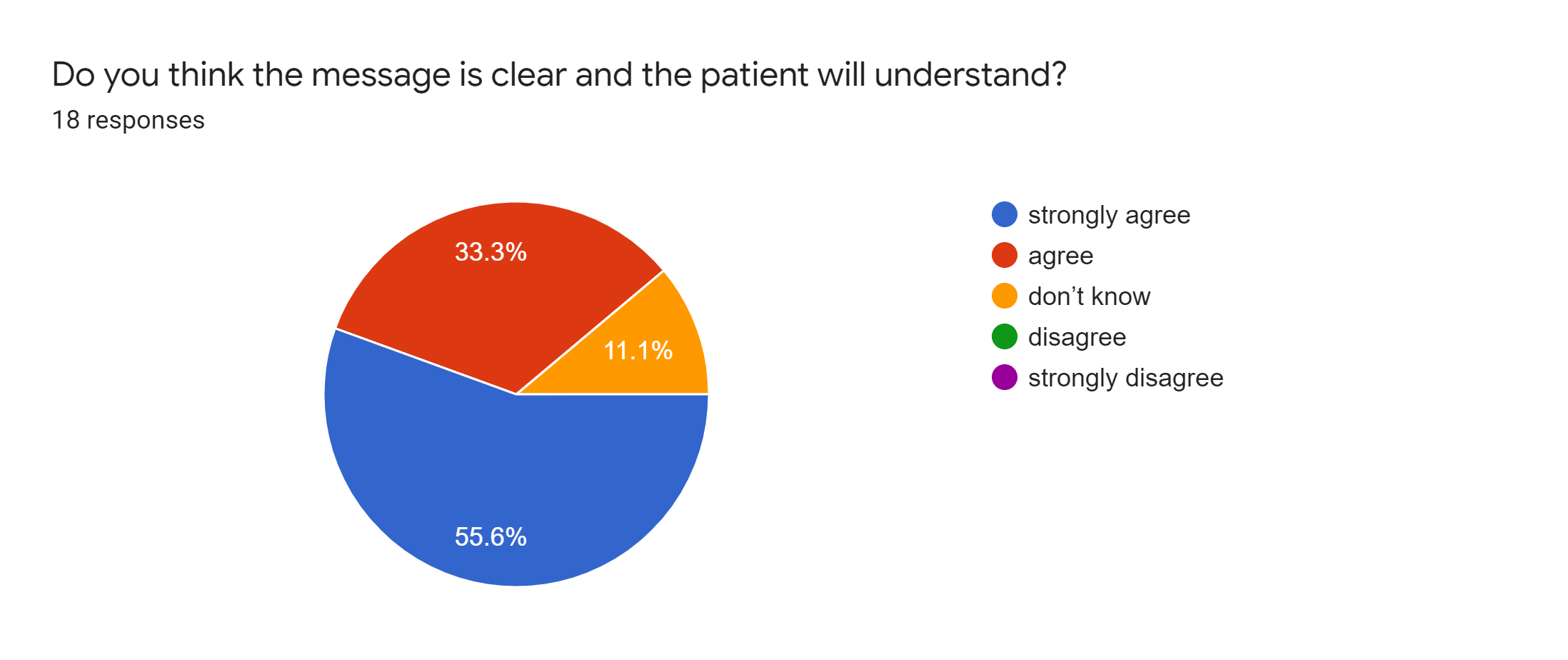


If you have any suggestions regarding the implications, please write them in the box below.5 responses

None

This slide is well modified and reviewed.

NO

Nil

Concerning the whole teaching material after the reviews and updates


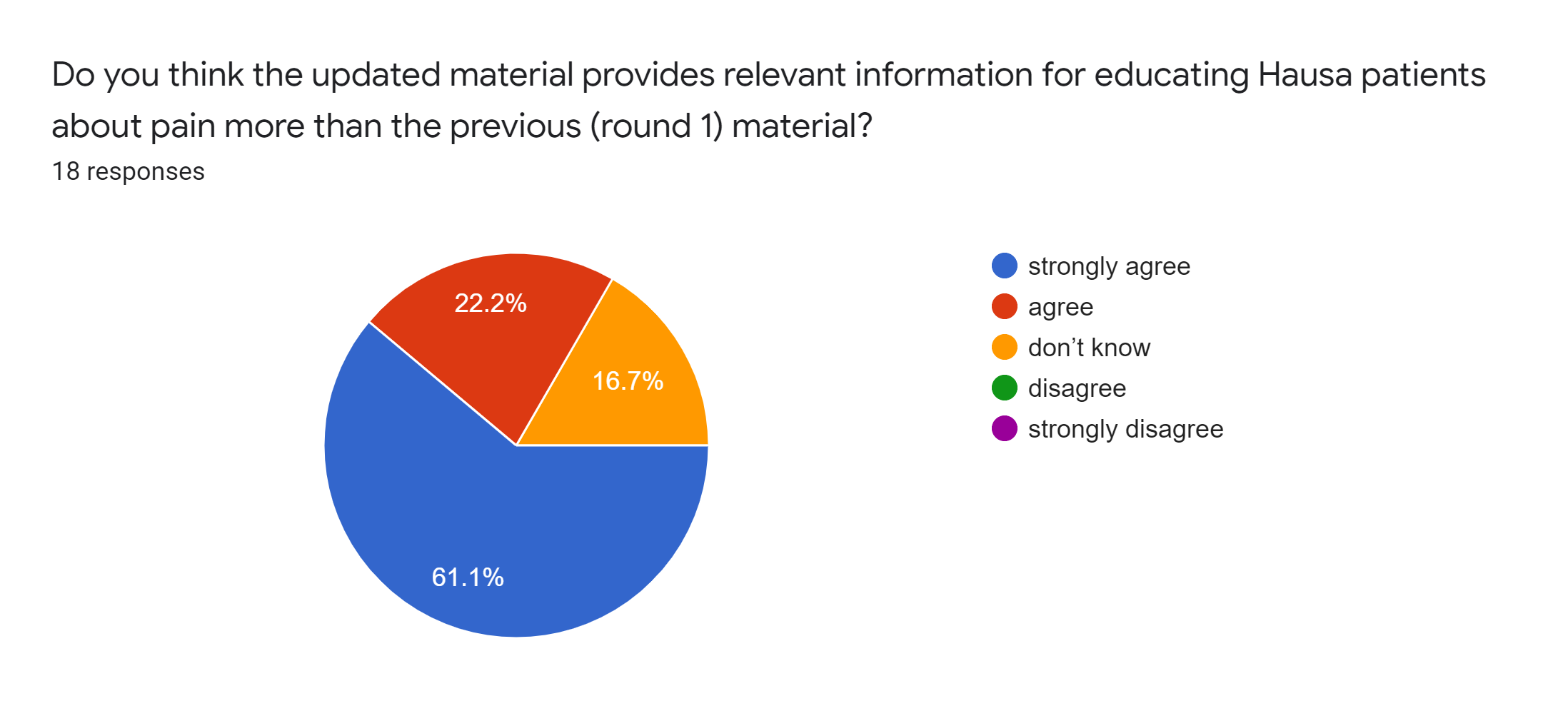


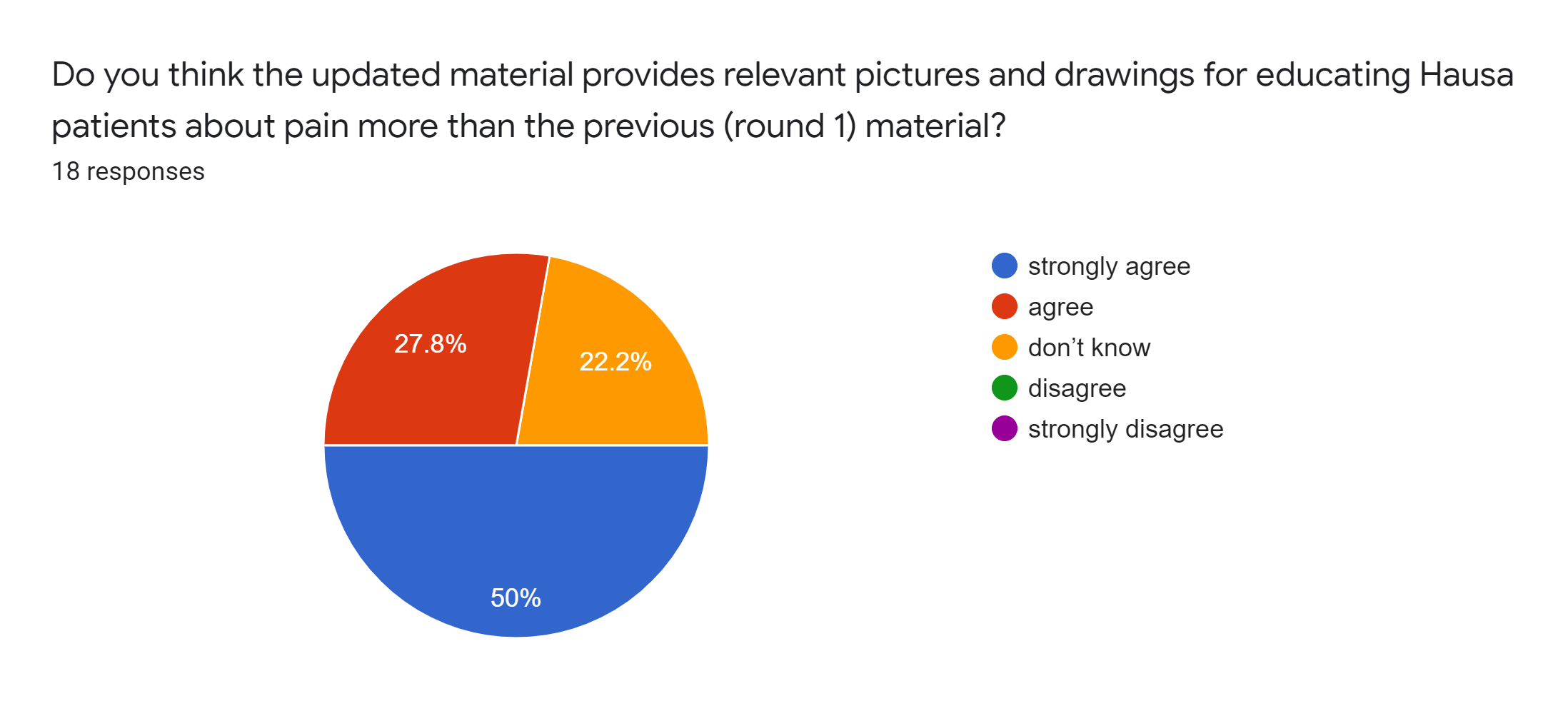


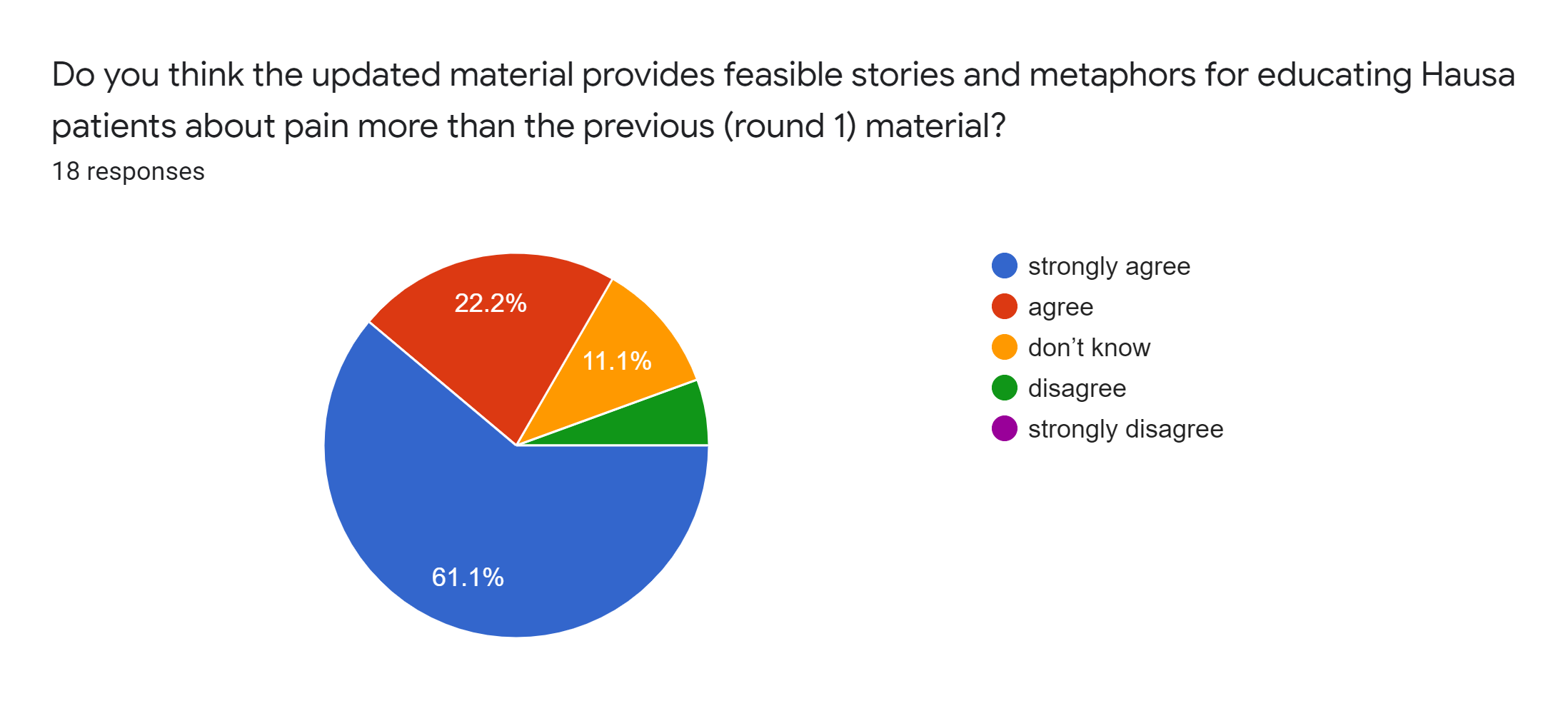


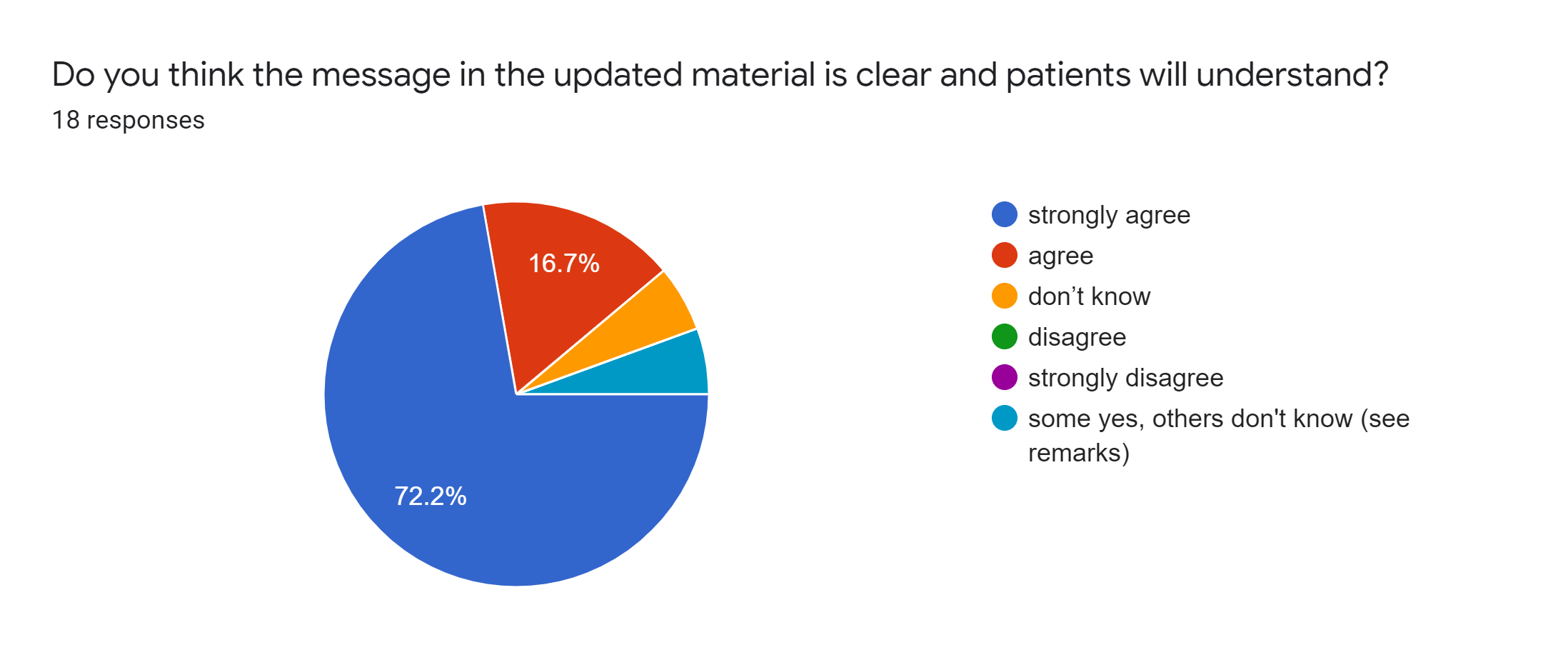


GENERAL QUESTIONS

1. Is there anything you would like us to add to this teaching material?15 responses

No

no

Short video clips(3D animations).many are not fond of reading but are good at watching videos which sticks to their heads

All most everything is include regarding to pains and it's mechanism.

Everything seems ok

See attached file

NO

again the cultural context is difficult to understand for me, but I really like the way you are trying to set this up

Please you can add story on sub-acute pain in Hausa

The pictures should be more of hausa people in outfit

I feel its explanatory and rich enough

2. Is there any specific thing you would like us to modify or simplify again in this teaching material?14 responses

No

Names in the stories are many.if its possible let's limit it to avoid confusion

To add a story or to have an interview with a person that has this problem.

I think every aspect of the education is well addressed

no

NO

traditional healers something to say about that to?

It's okay

Nil

see remarks

No,youve captured all necessary information

3. Further suggestions?8 responses

Continues researching about pains and it's related

No

No additional suggestion

It's wonderful, excellent. Well understandable. Thank you

None

Nil

More simpler way of explaining pain modulation

-

Questionnaire link for 2B: <https://docs.google.com/forms/d/e/1FAIpQLSc7gIbdaytTXq7dFJ4JZcjJ12emZlDlc60ugjRzFMlR0BL8RQ/viewform?usp=sf_link>

Responses:


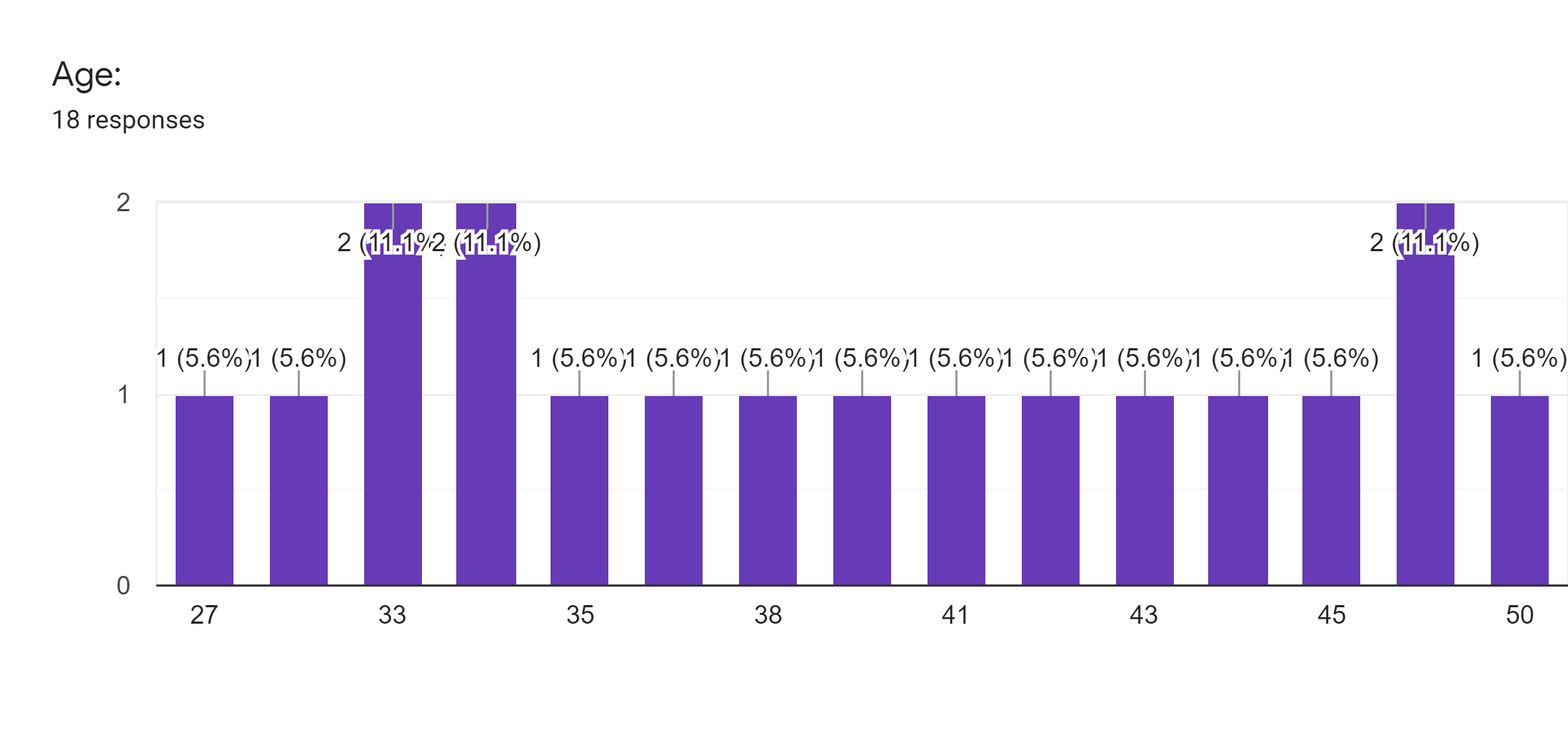


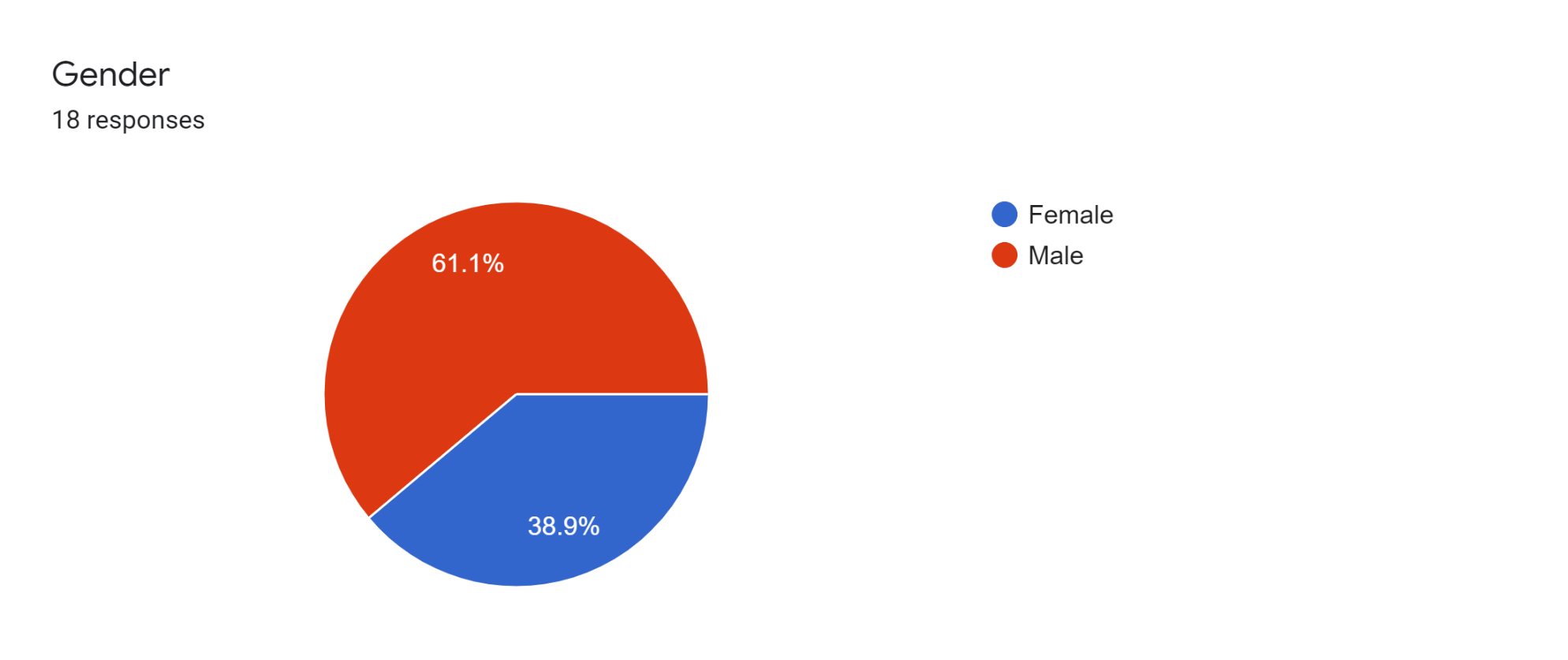


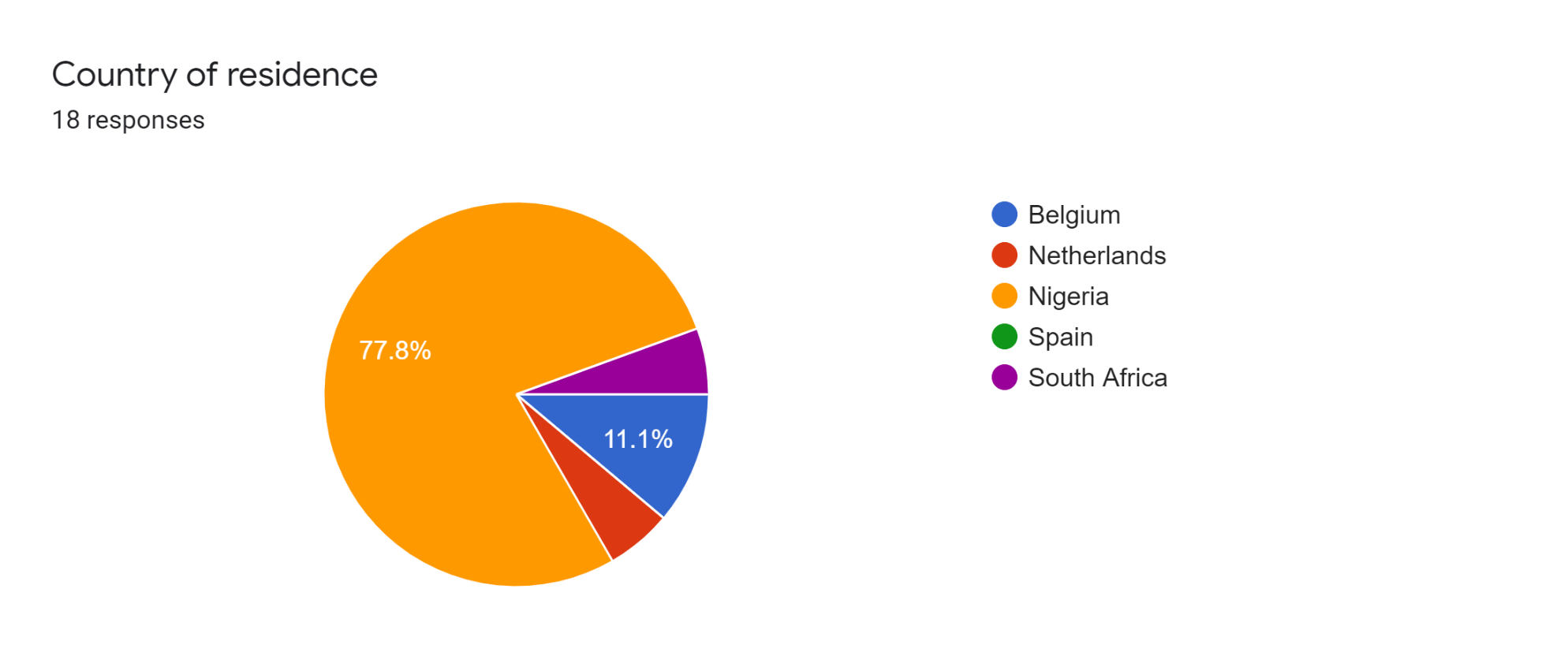


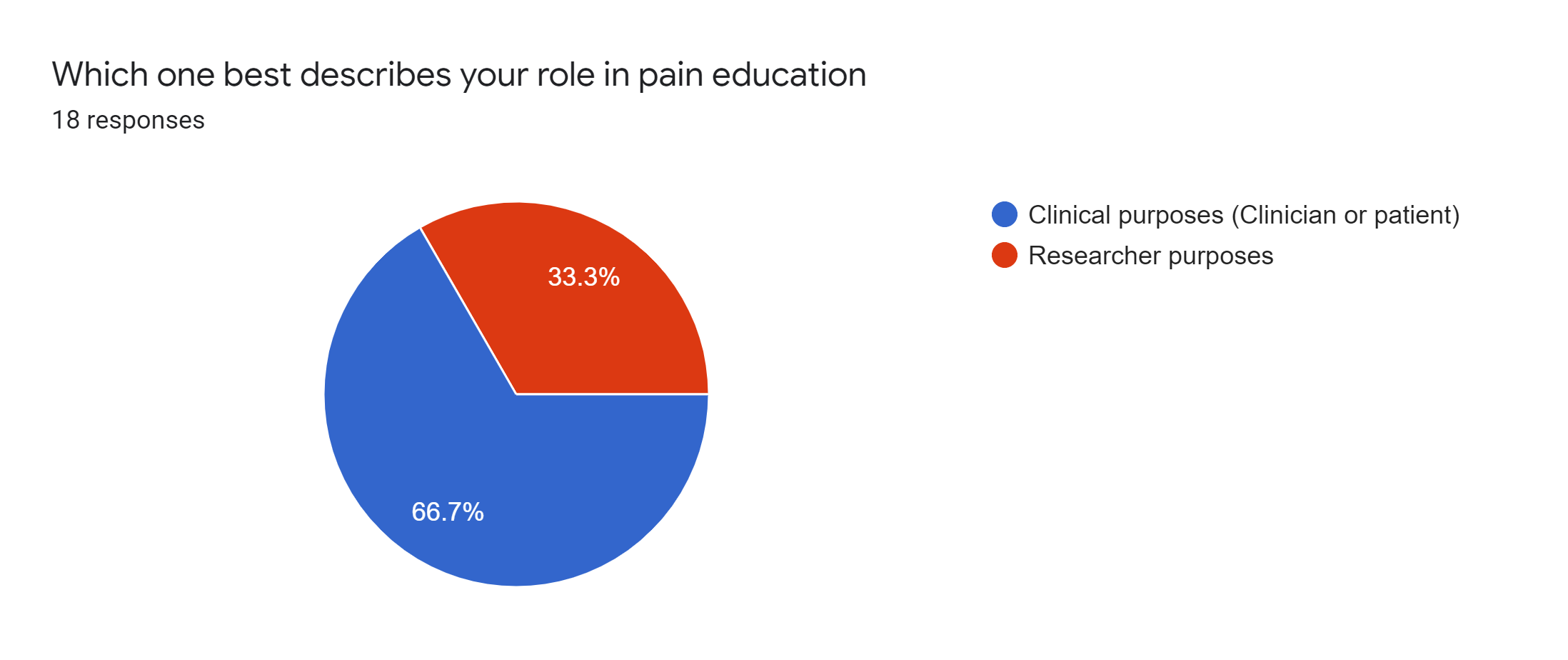


What is your experience with pain education?

18 responses

Non-existent 16.7%

Heard of it 5.6%

Familiar with it, <1 year 16.7%

Familiar with it, 1-5 years 33.3%

Familiar with it, 6-10 years 11.1%

Familiar with it, 11+ years 16.7%


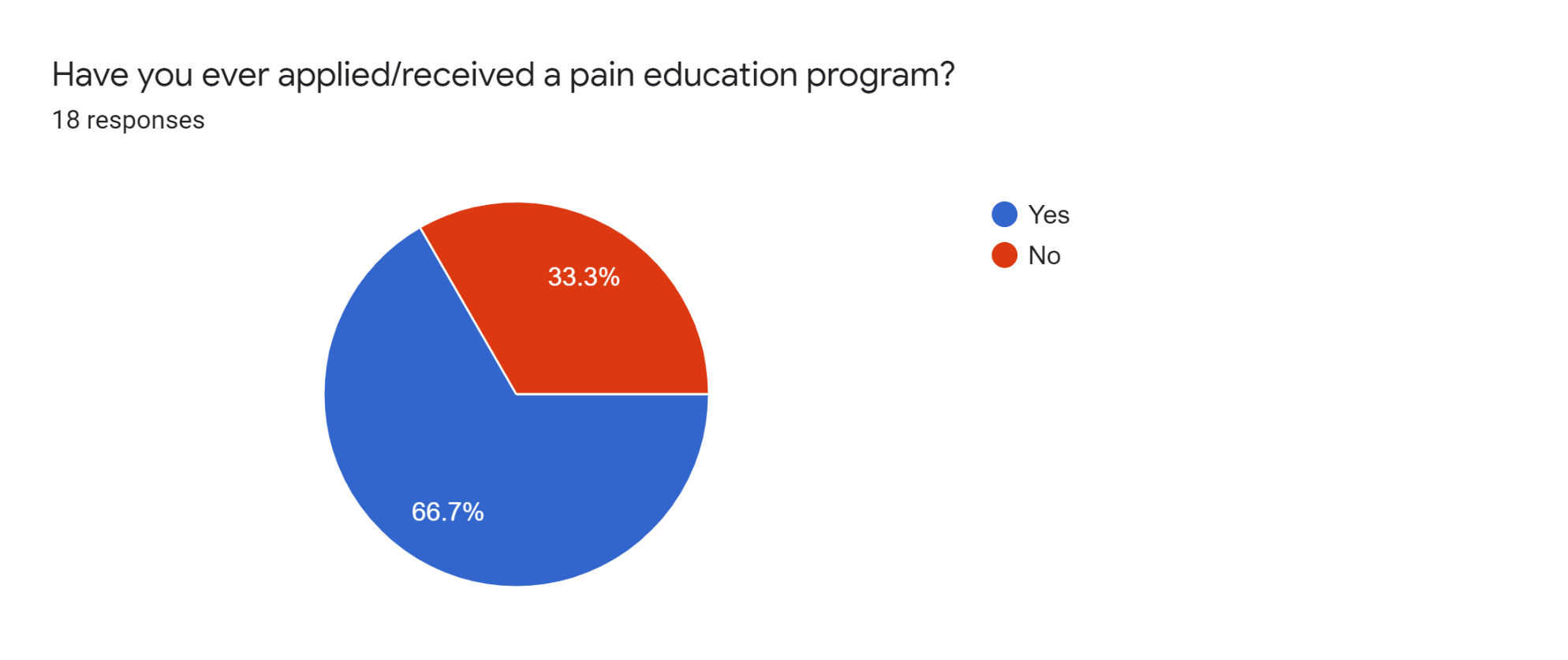


Introduction


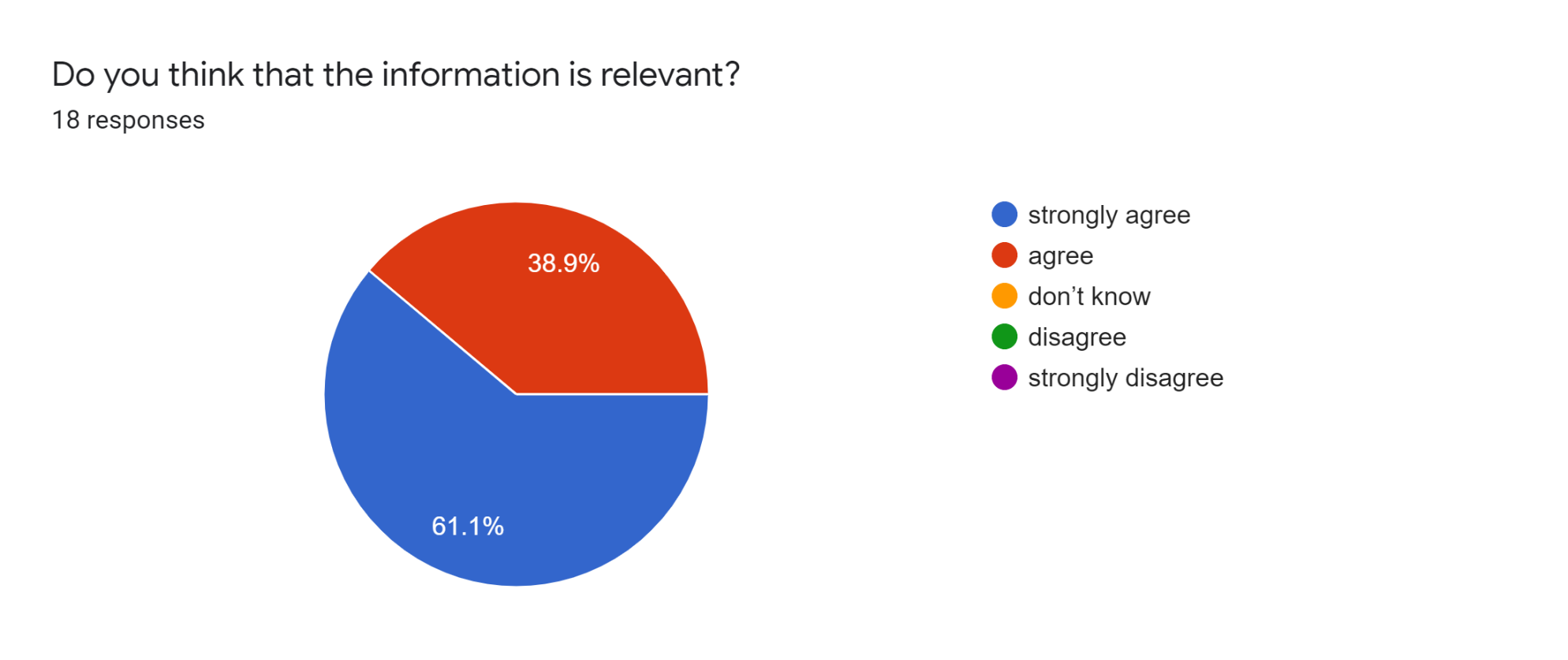

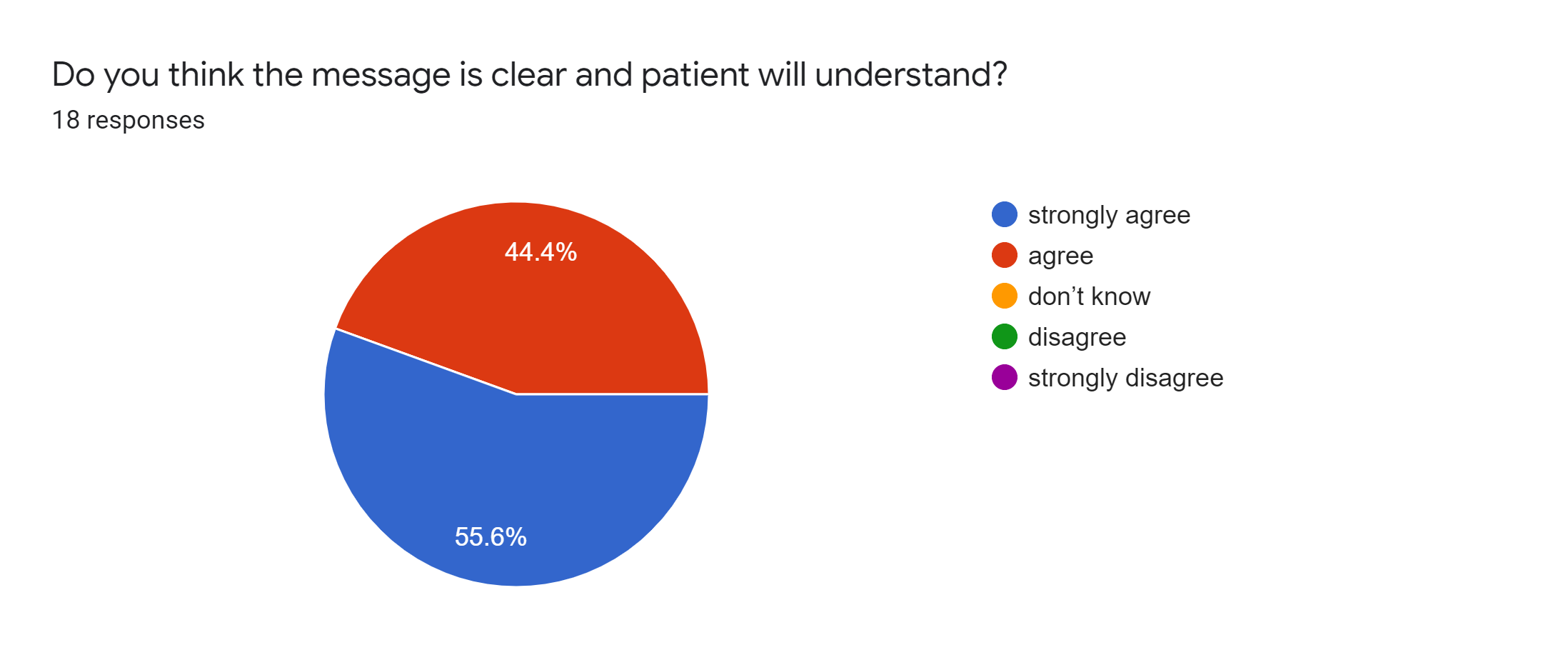


If you have any suggestions regarding this page, please write them in the box.

6 responses

No

None

As i suggested earlier,names shouldnt be too many

More home education should be provided since many patients do not go to the hospital

The problem with detailed information to patients is that they do not recognize their own specific problem so in general for the whole information do not make it to much in detail

Acute Pain


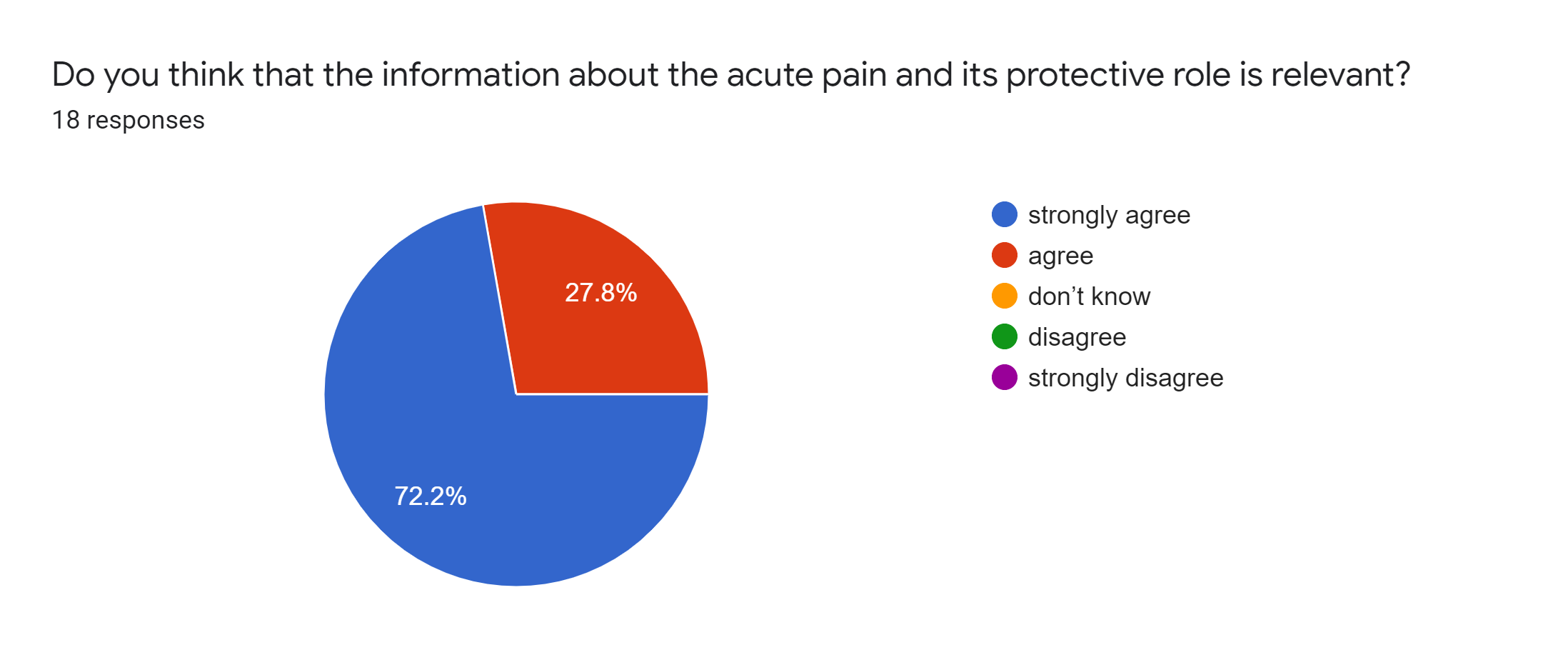


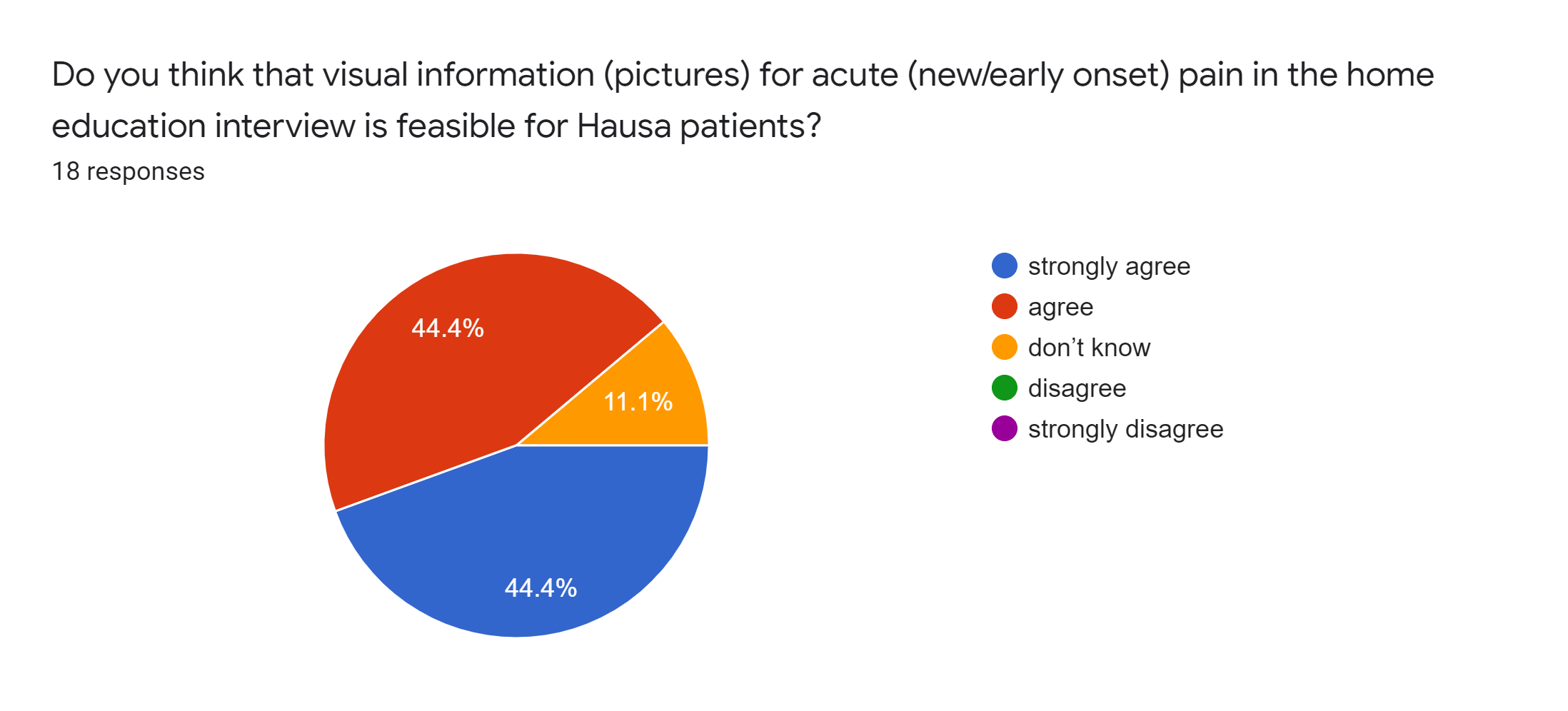


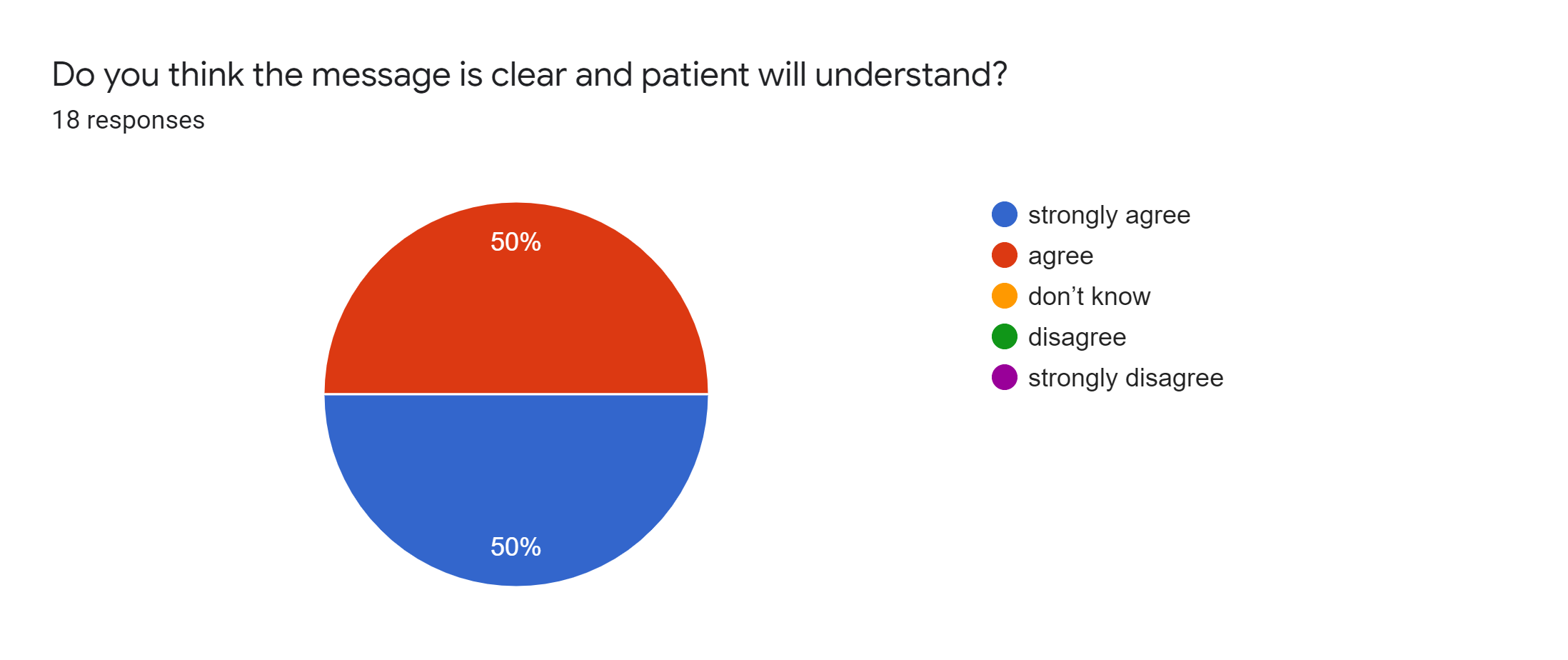


If you have any suggestion(s) regarding acute pain and the related explanations, please write them in them down.6 responses

None

Short video clips may be of help

Pictures

Some of the responses in the conversation seems a little too long

No

Do not start to early explianing everything is in the brain explain it slowly not to lose your patient

Pain biology


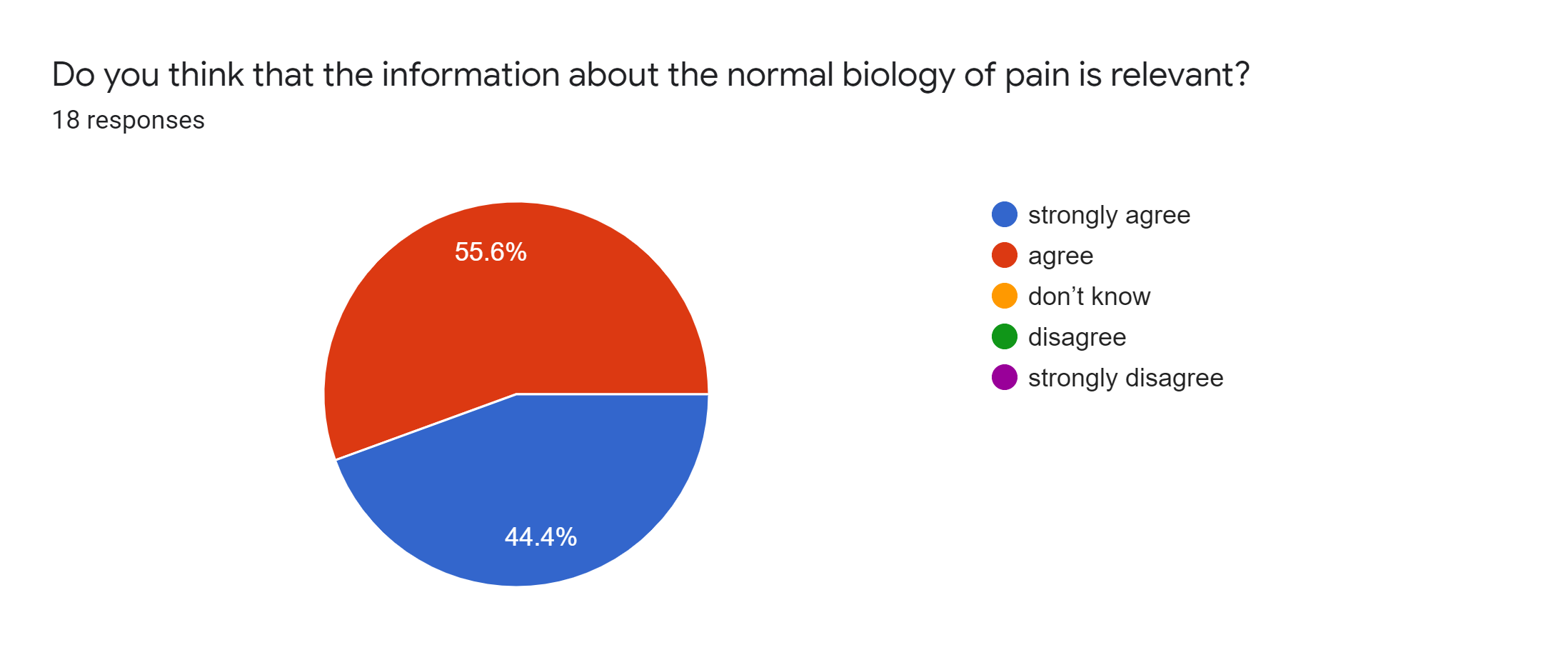


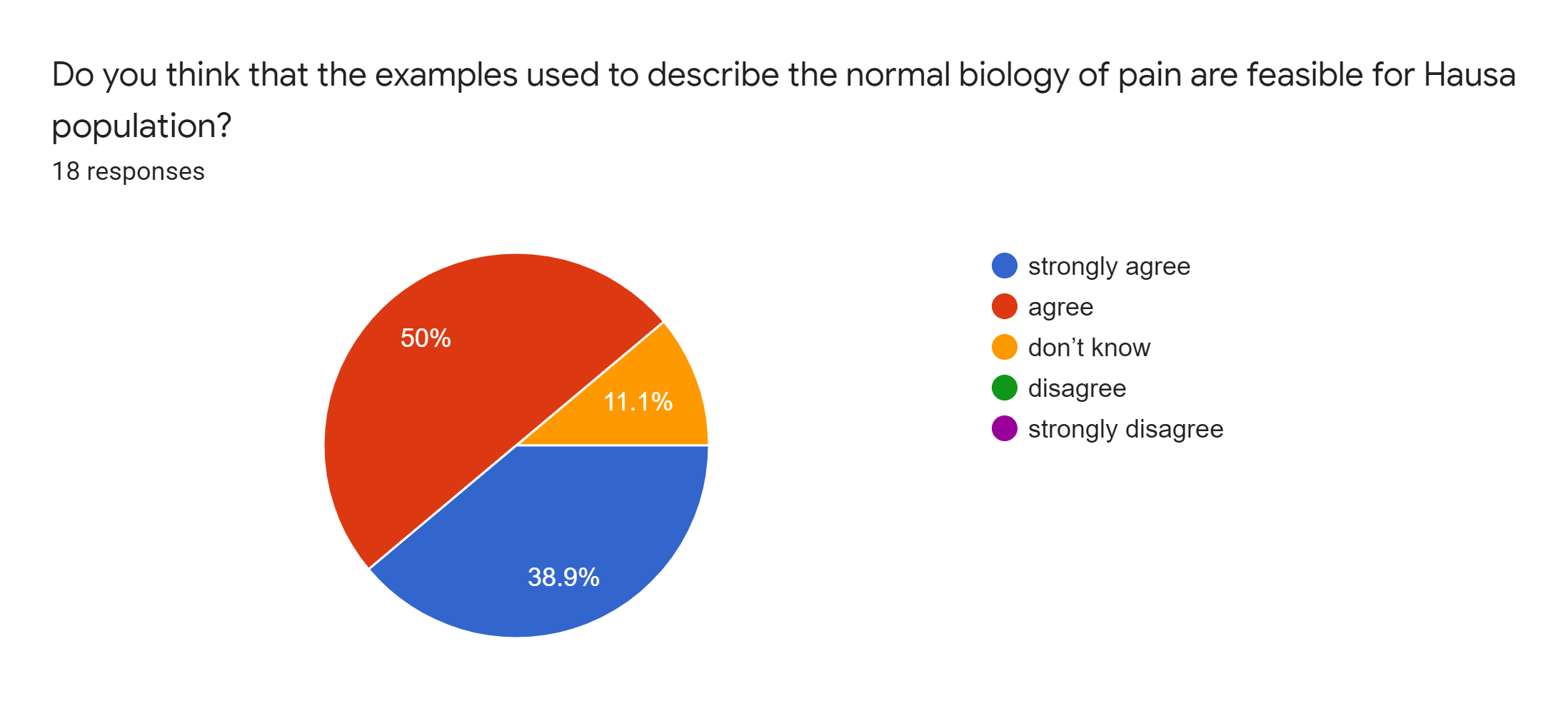


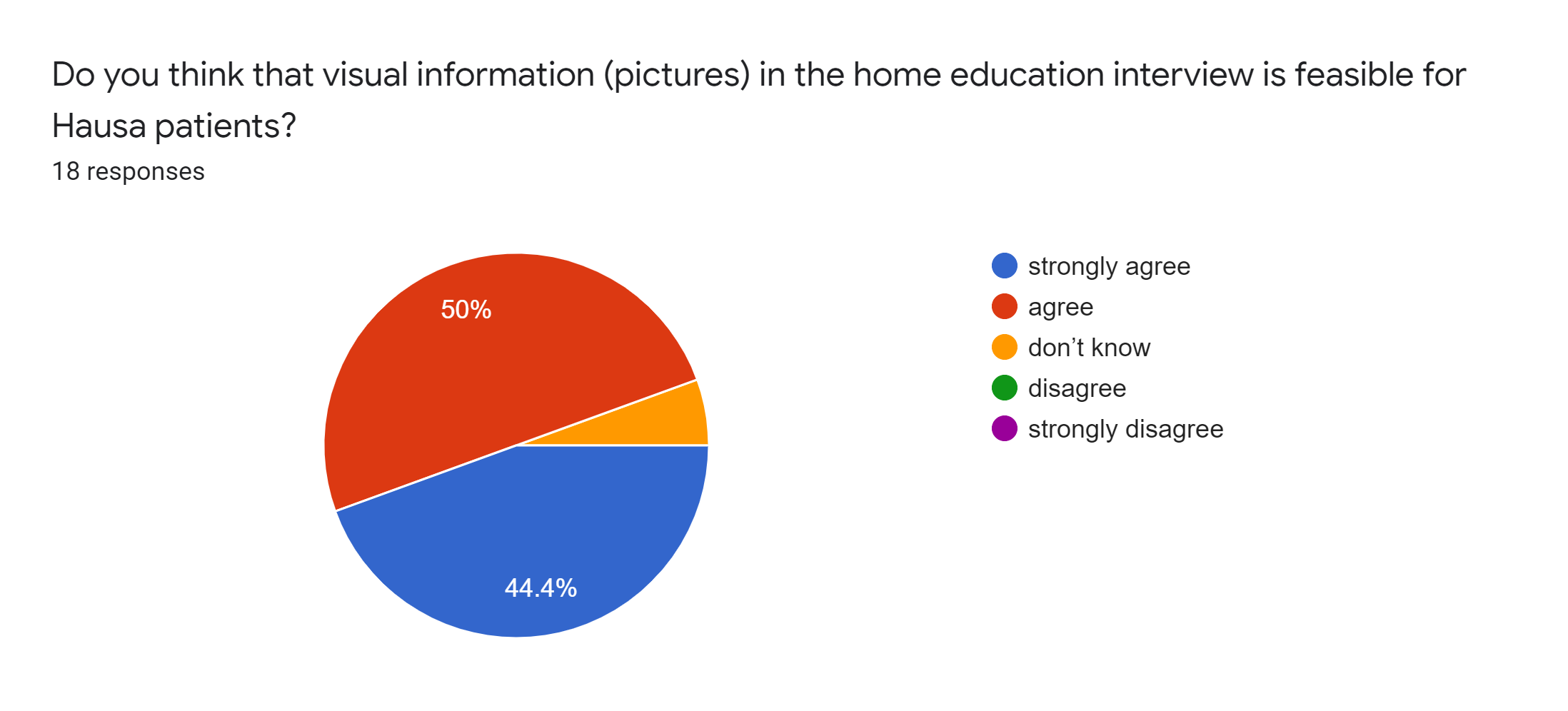


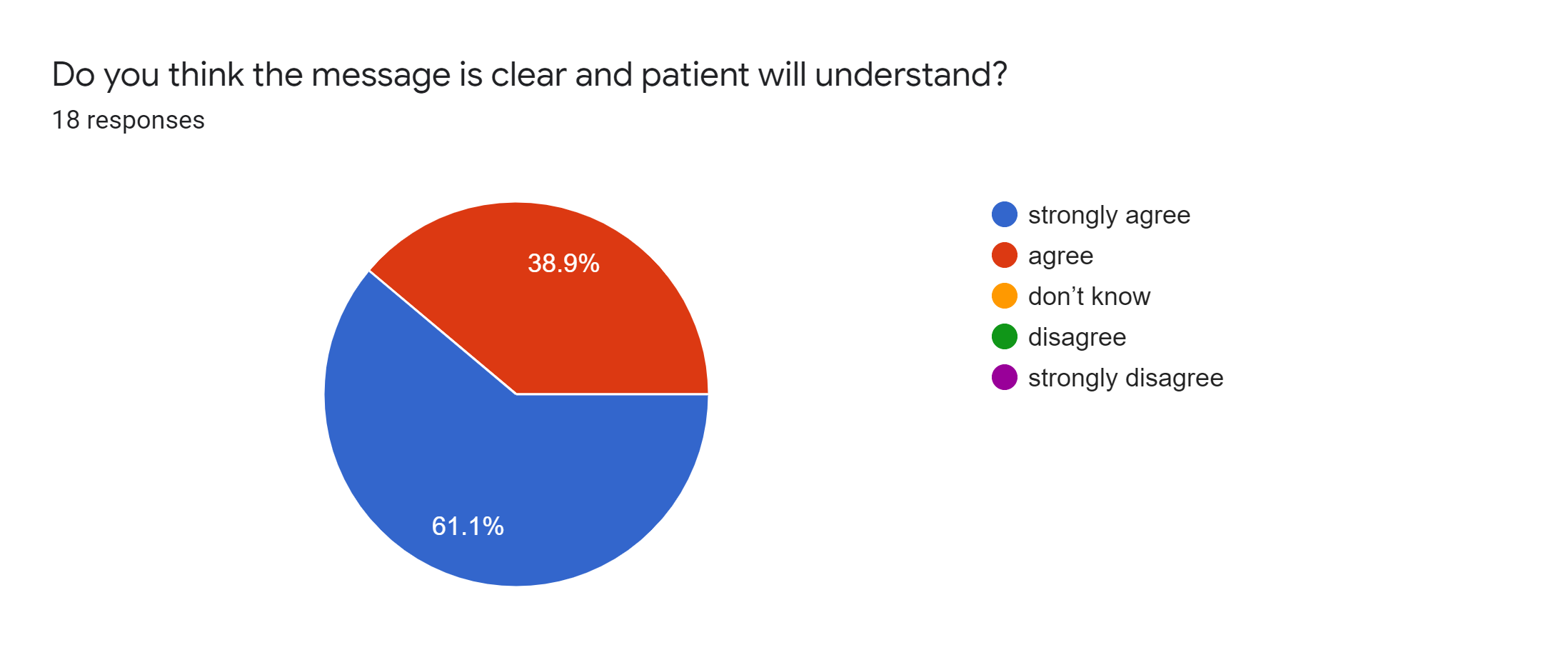


If you have any suggestions regarding the explanation of the normal biology of pain, please write them in the box.

6 responses

None

The normal biology of pain if possible should be more simpler as the illustration is a bit complicated esp for the layman

No

It is difficult for me to understand the cultural background and perceptions and of Hause people

Pain Modulation


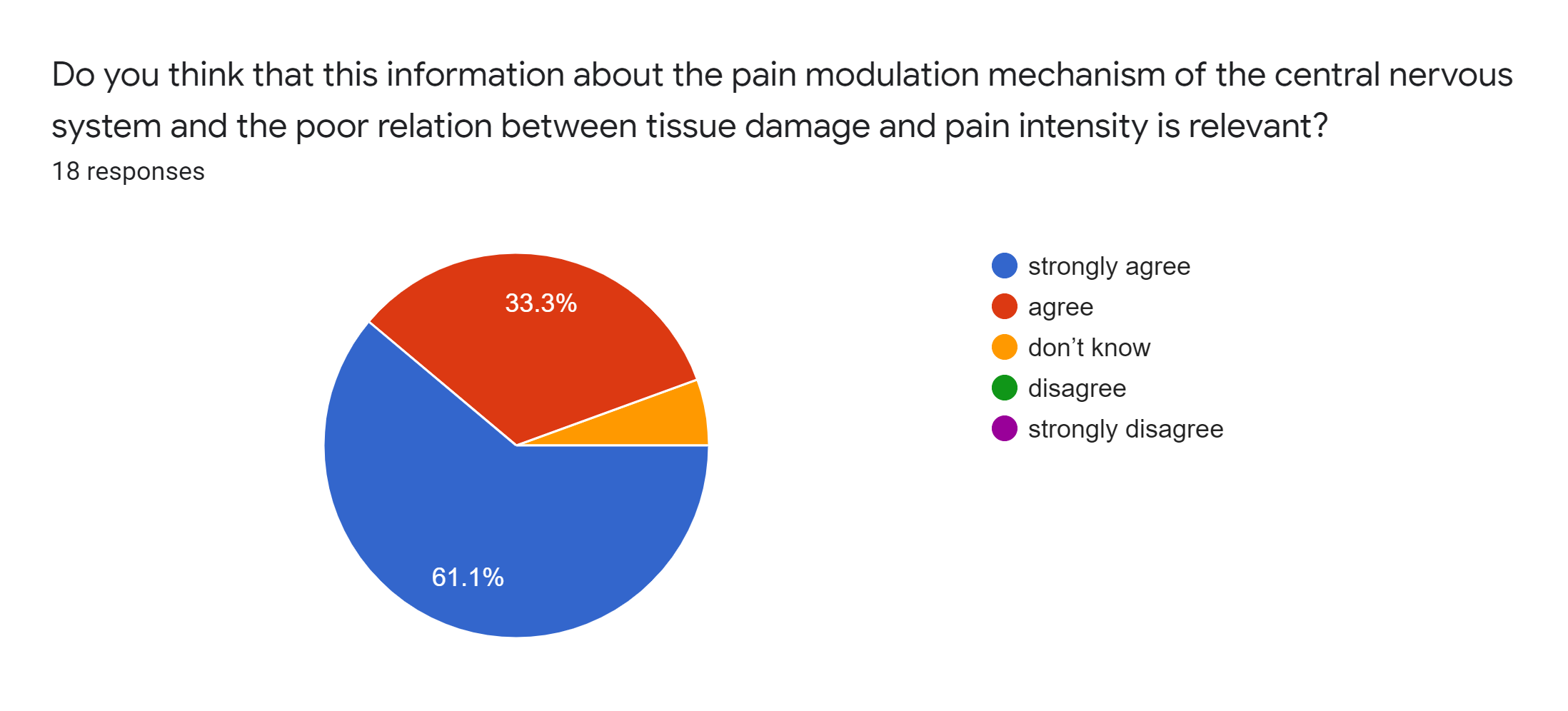


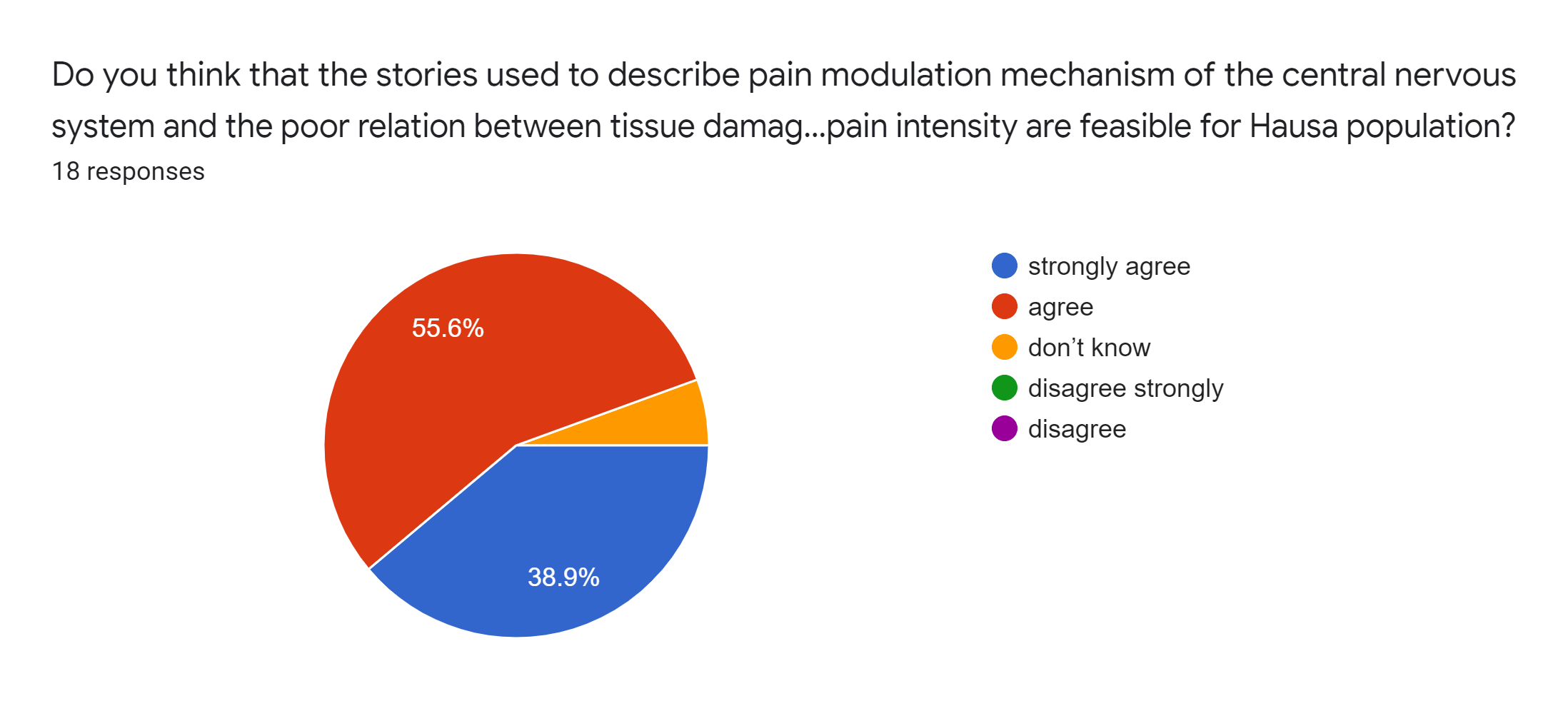


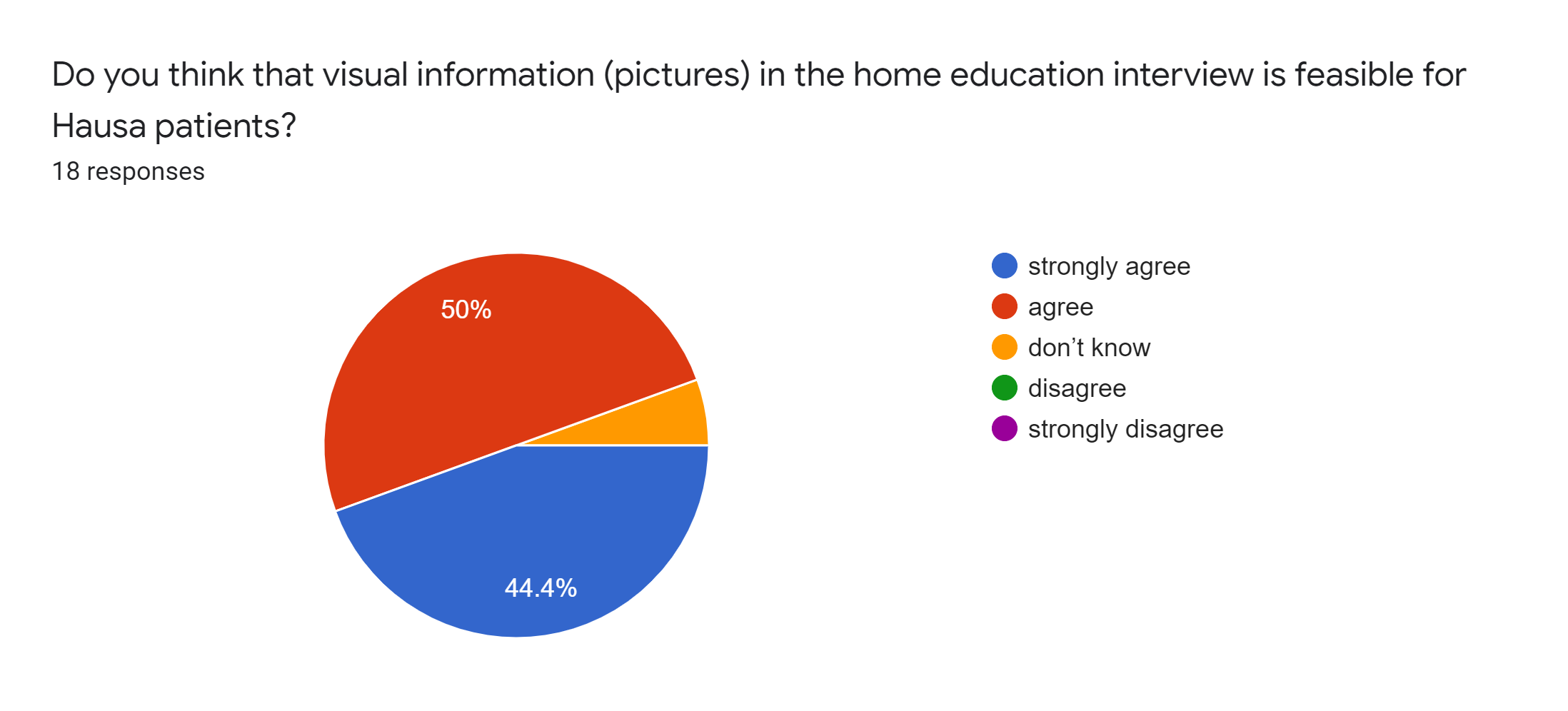


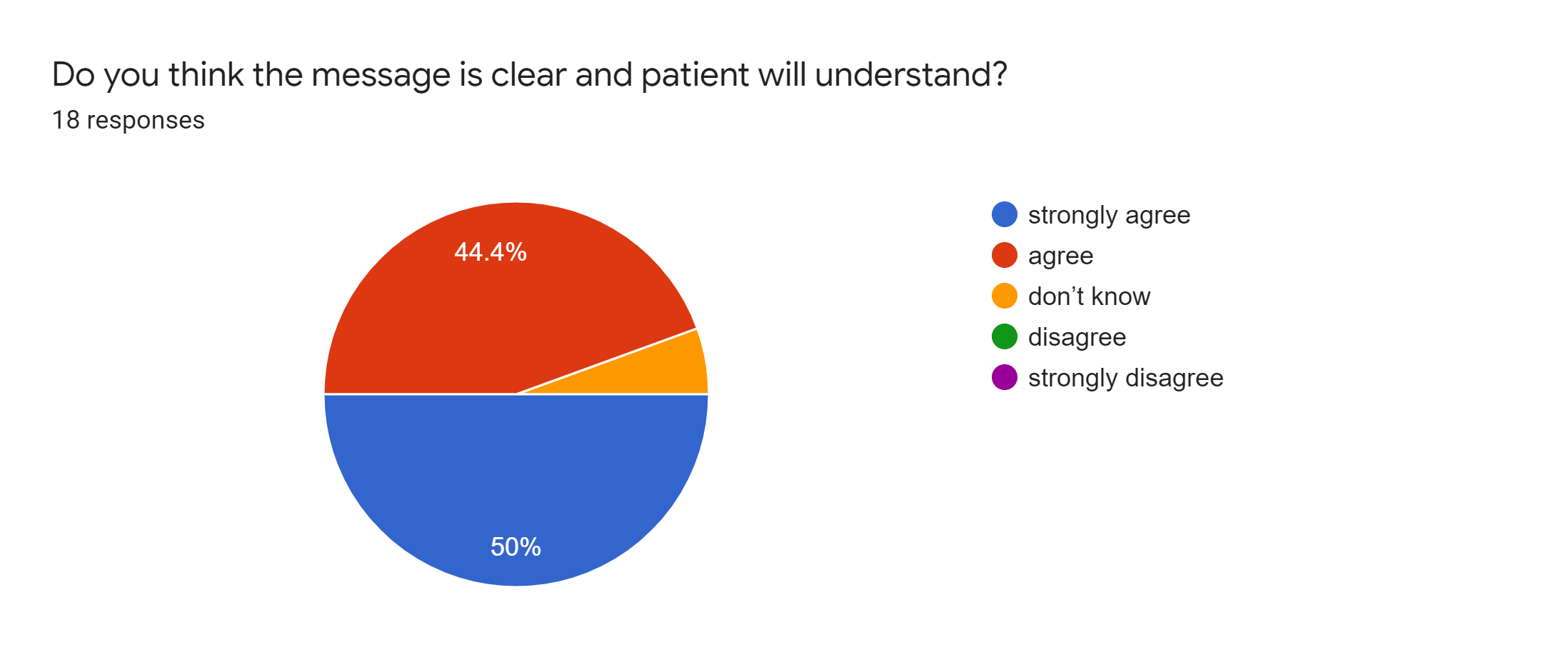


If you have any suggestions regarding the explanation of the pain modulation mechanism of the central nervous system and the poor relation between tissue damage and pain intensity, please write them in the box.5 responses

Nil

No

Please you need to improve on the pictures for the patients to understand very well.

None

torture is a very specific example maybe suitable I dont know but you have to be sure they recognize this

Chronic pain


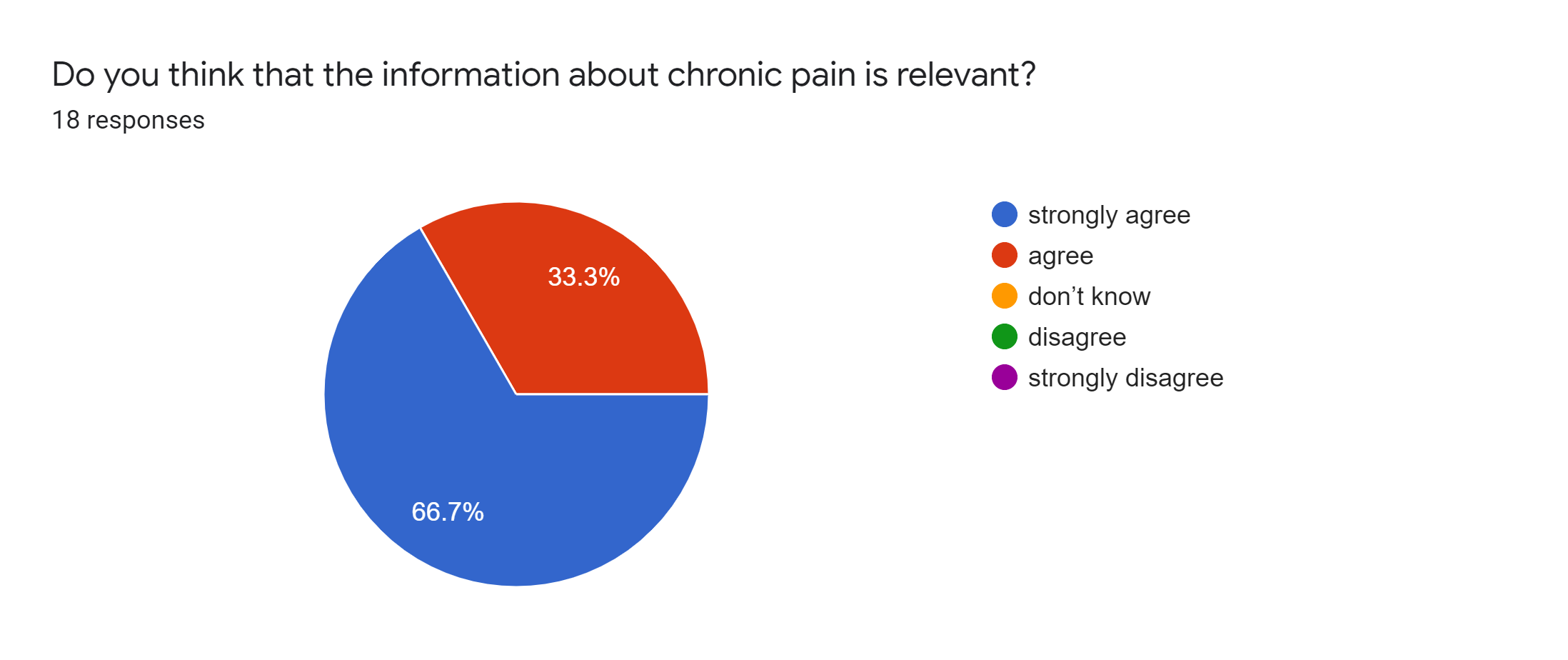


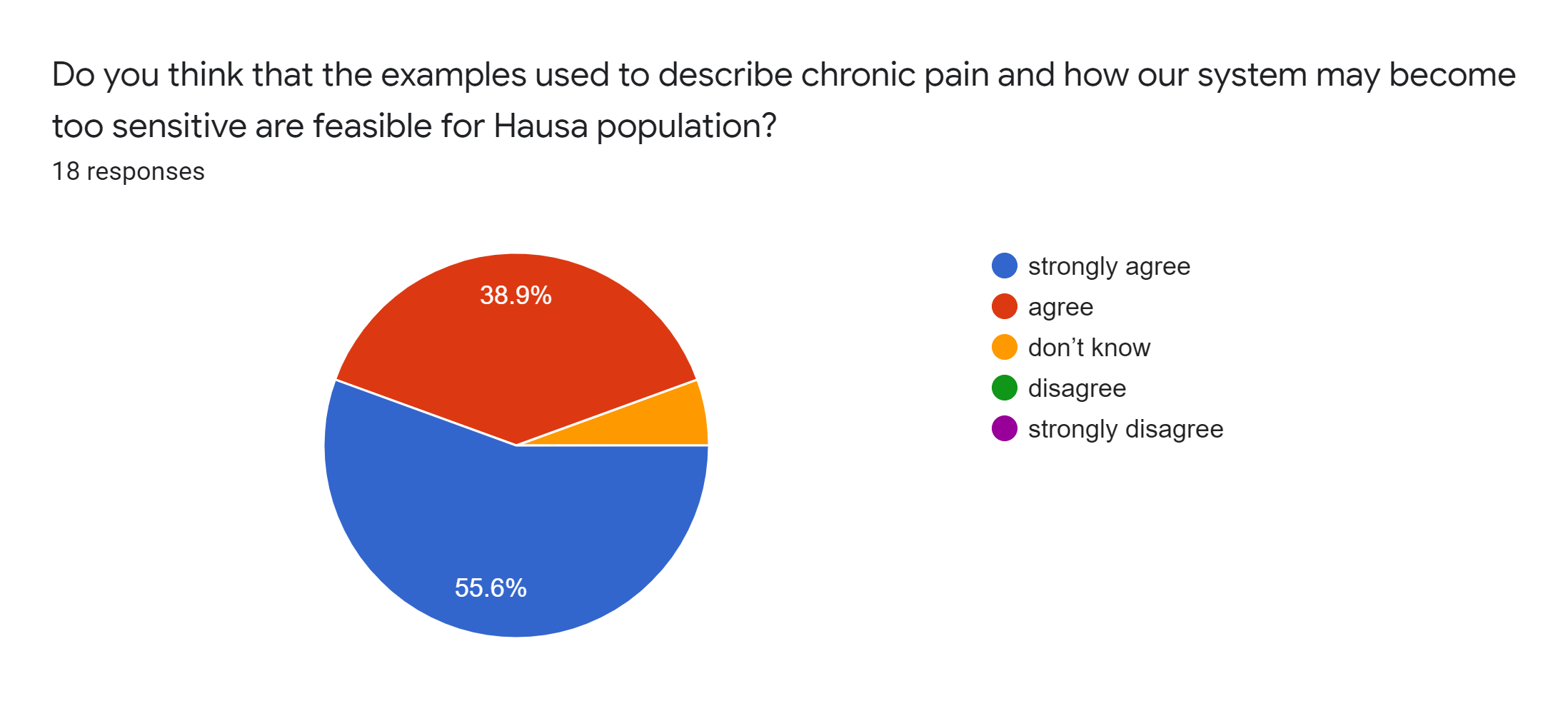


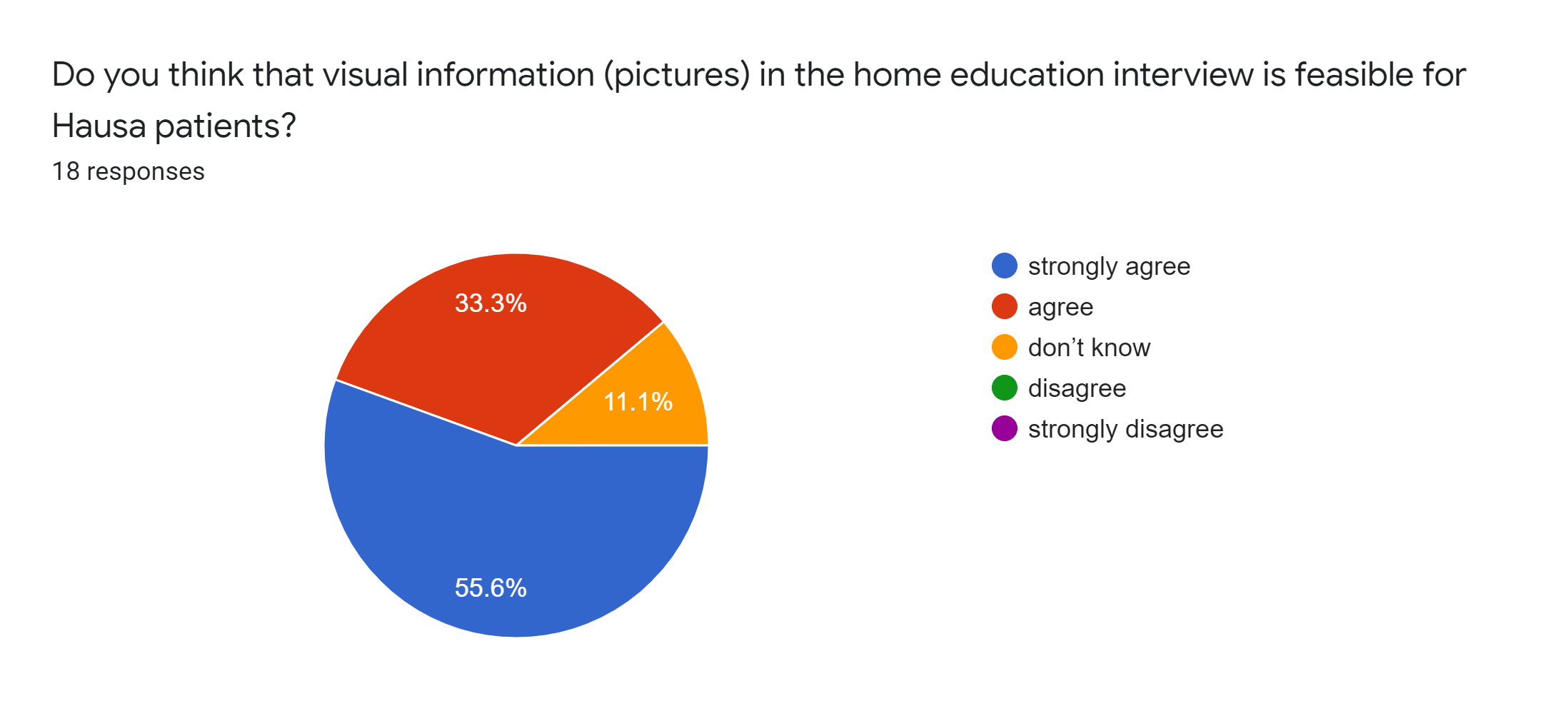


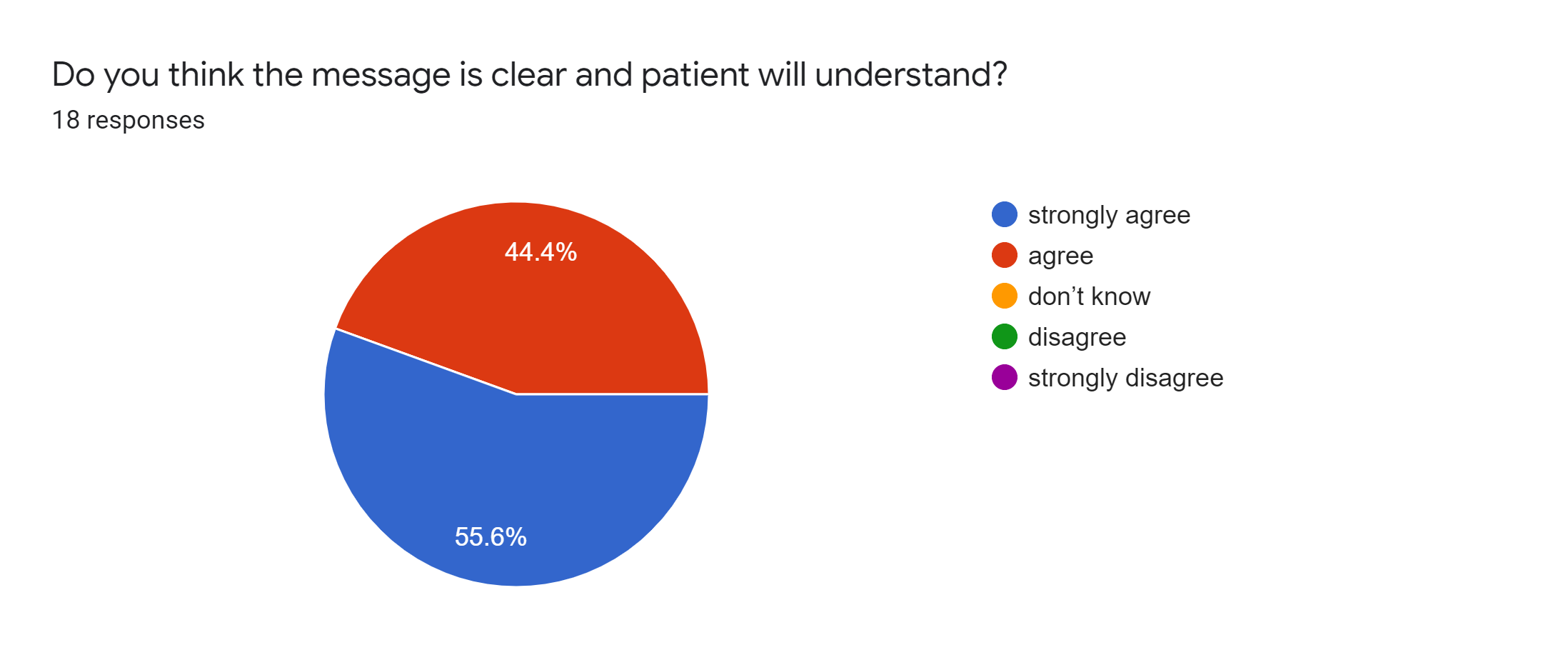


If you have any suggestions regarding the description of chronic pain and how our system may become too sensitive, please write them in the box.5 responses

No

Nil

None

Almost every things is explained.

Beliefs, thoughts and behaviors


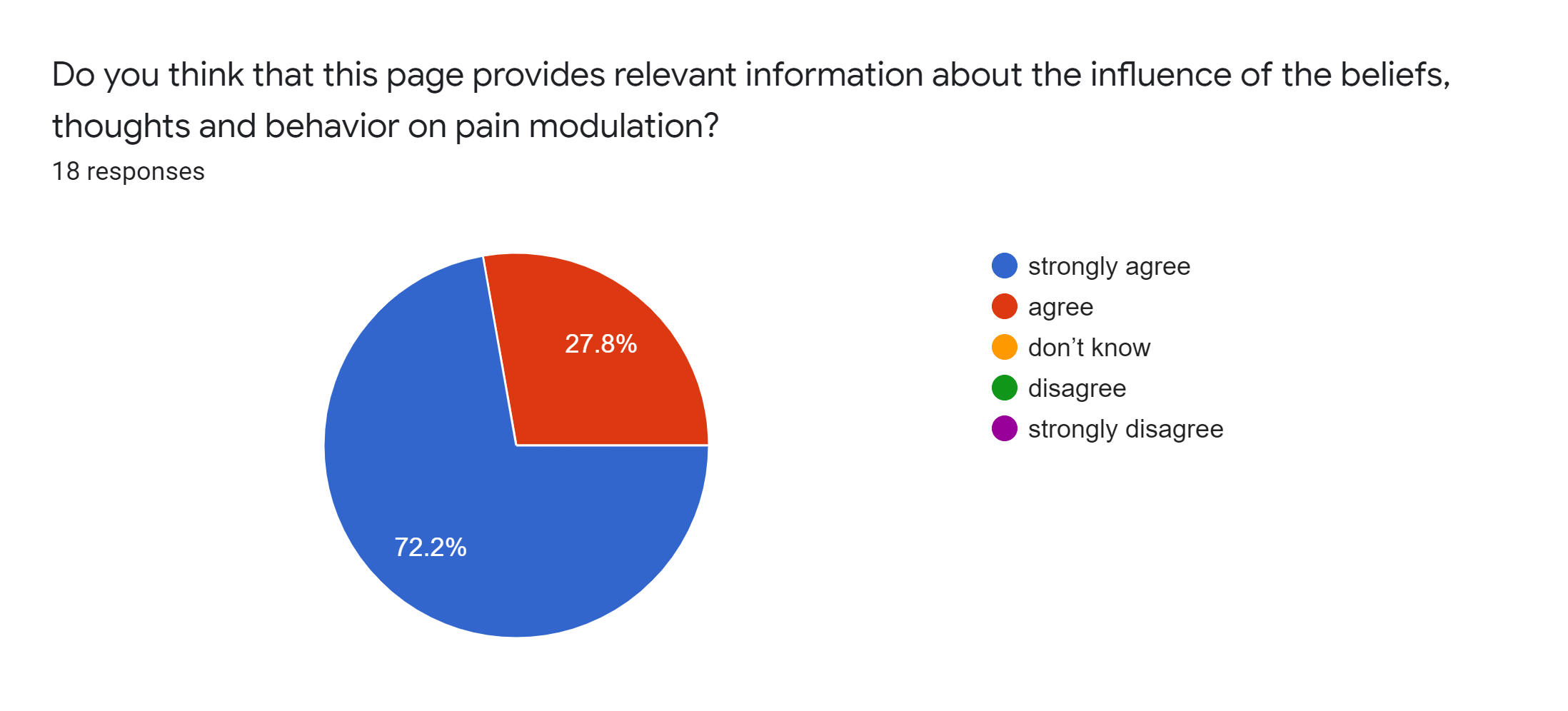


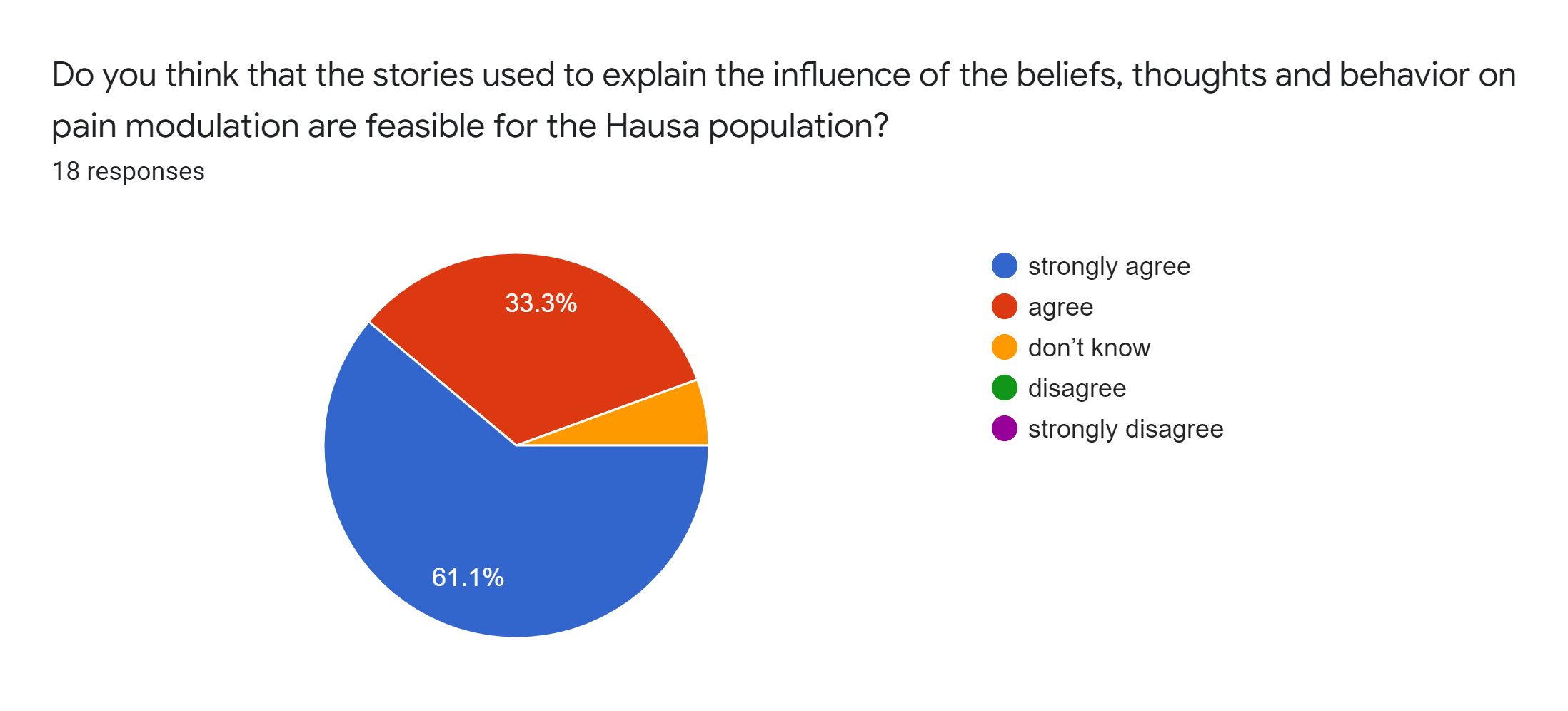


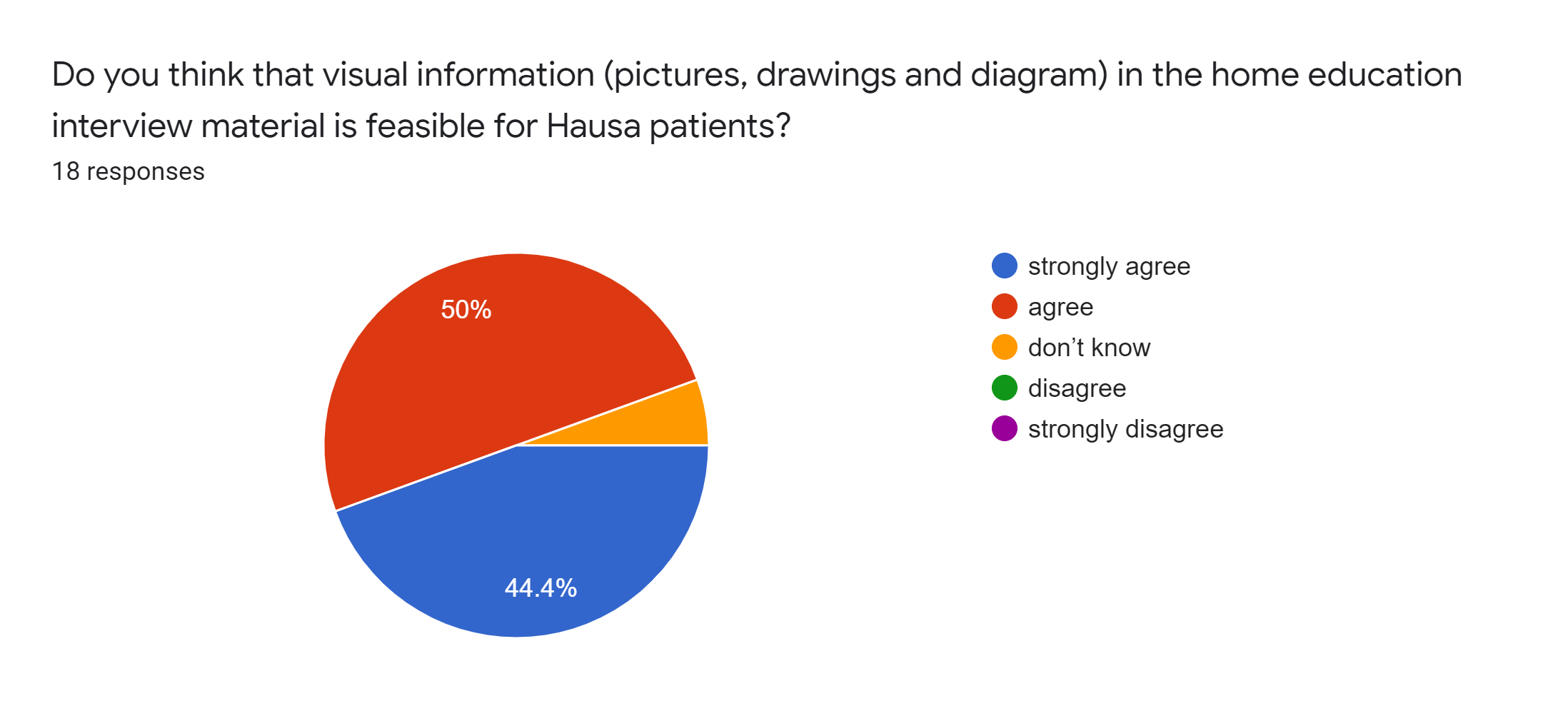


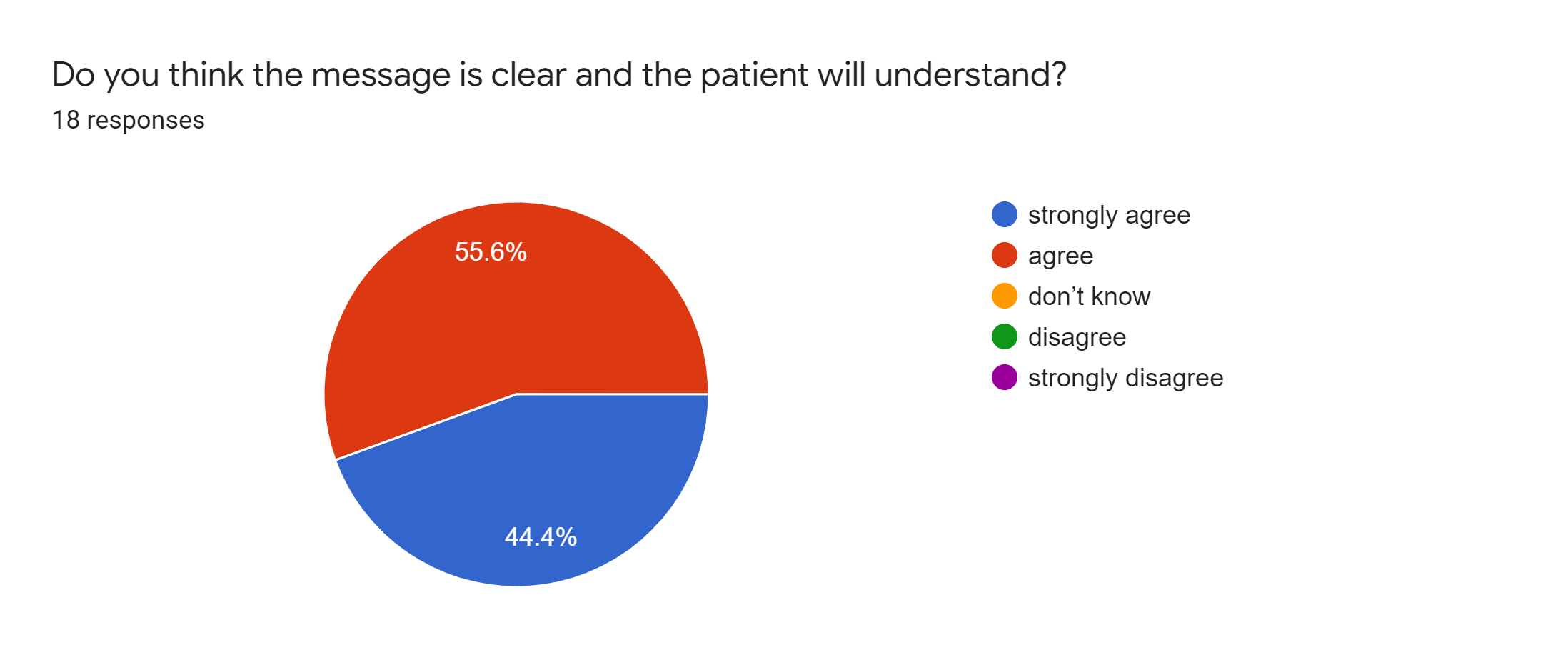


If you have any suggestions the influence of the beliefs, thoughts and behavior on pain modulation, please write them in the box below.5 responses

None

Nil

No

Most people here do not believe in behavioral aspect o pain so this information is supposed to capture that

I am not sure about the example of the woman with pain following divorce and the statement that if she settles with her husband that her pain will get better. If he had abused her - it might not. Perhaps a different example would be better here?

Implications


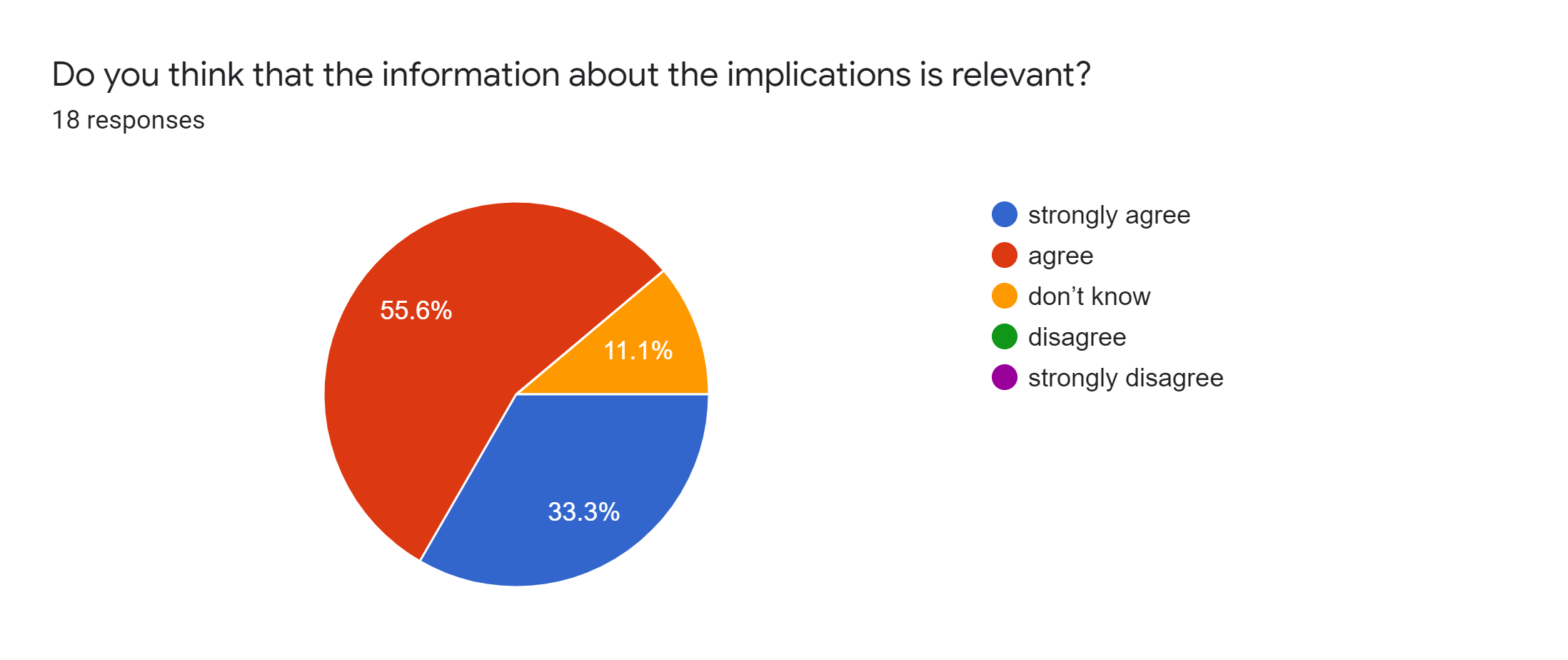


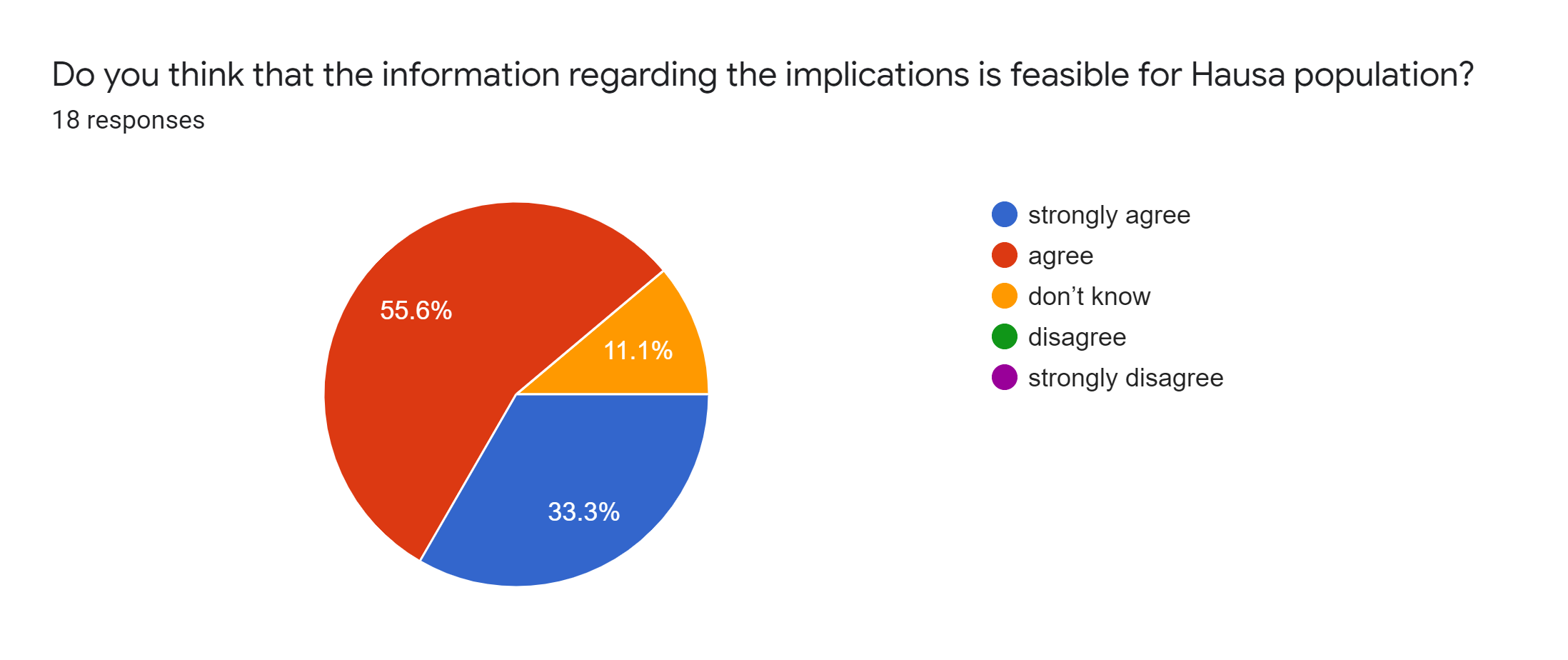


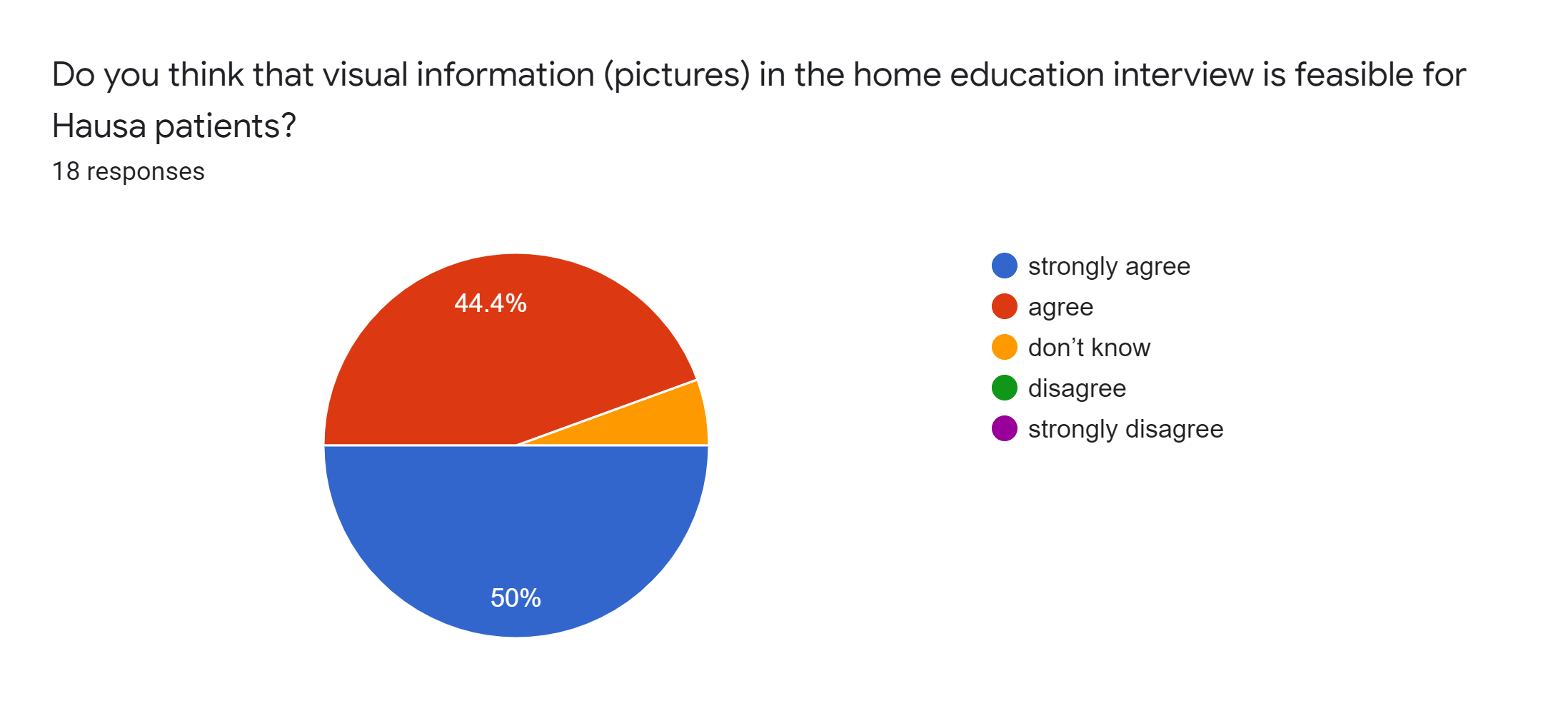


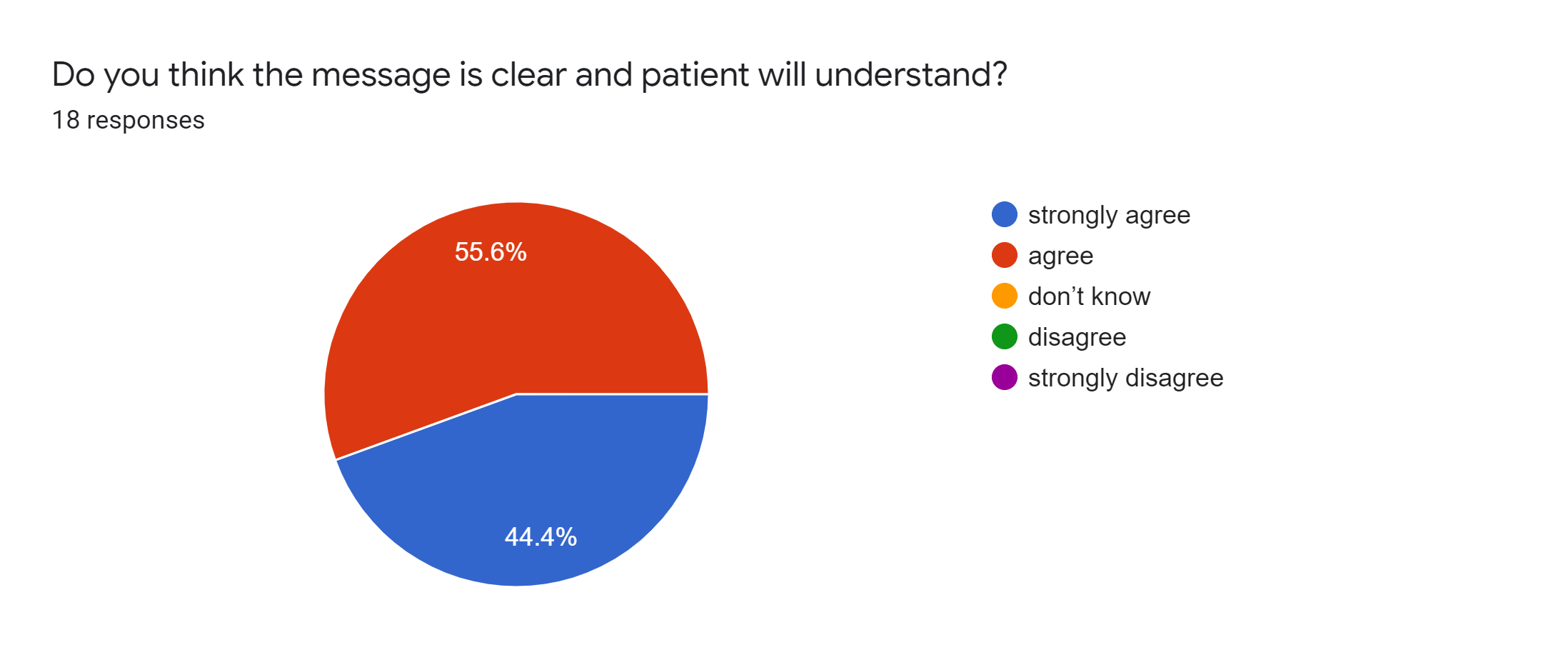
 If you have any suggestions regarding the implications, please write them in the box.4 responses

Nil

none

None

I would add more psychological and sociale issues into the text this is very limited using these examples

GENERAL QUESTIONS

1. What do you think about the order and the concept? Is it understandable, logical?18 responses

Yes

Yes it is

yes

okay

Interview material is ok

Yes. Its logical and understandable

The concept is very OK and interesting

It's understandable and logical

It's logical and understandable

It is understandable

Understandable

Yes builds logically but could perhaps have more information on the actions that patients can take?

Is very understandable

Oke

2. General remarks?17 responses

the questionnaire is feasible for Hausa community

no comments

Thanks you

Excellent work and approach

Self explanatory especially with the pictorial images

Satisfactory

I prefer this file than the other one

Modulation need more easy explanation

Best wishes

The concept is very educative and informative

You only need to improve on the pictures and diagrams

I am happy with this concept.

I think the material developed will be feasible

Is quite an Educative material

Perhaps some guidance on where patients could find more information about how to exercise, sleep hygiene and methods to use for relaxation - telling them where to find these?

It is a very good idea indeed. Because it can help many people to understand their problems regarding pains.

Maybe this is much if they have to read it?

3. Further suggestions?14 responses

the slides for pain education should be short and preside

-

None

It should be given widest dissemination to reach the professionals and encouraged to make use of it

Animations,if possible

see attached file

Nil

None at the moment

none

I am not sure if I have seen anything on sub-acute pain.

Provide to more people

Take it to greater height

Use real patient scenario for the interview material also .

Put more effort in adding cultural specific emotional and social factors maybe ancestors?
