# Supplementary material for: Development of culturally sensitive pain neuroscience education materials for Hausa-speaking patients with chronic spinal pain: A modified Delphi study
Source: PLoS One. 2021 Jul 2;16(7):e0253757. doi: 10.1371/journal.pone.0253757 (PMC8253446; doi:10.1371/journal.pone.0253757)
Supplement: S3 Data — (DOCX) [file pone.0253757.s009.docx]

Delphi round 3

Questionnaire link: <https://docs.google.com/forms/d/e/1FAIpQLSdsBaUFAeicFR1kuyWjc94j_Sykpimf0eQhKij-aKlkg8osOA/viewform?usp=sf_link>


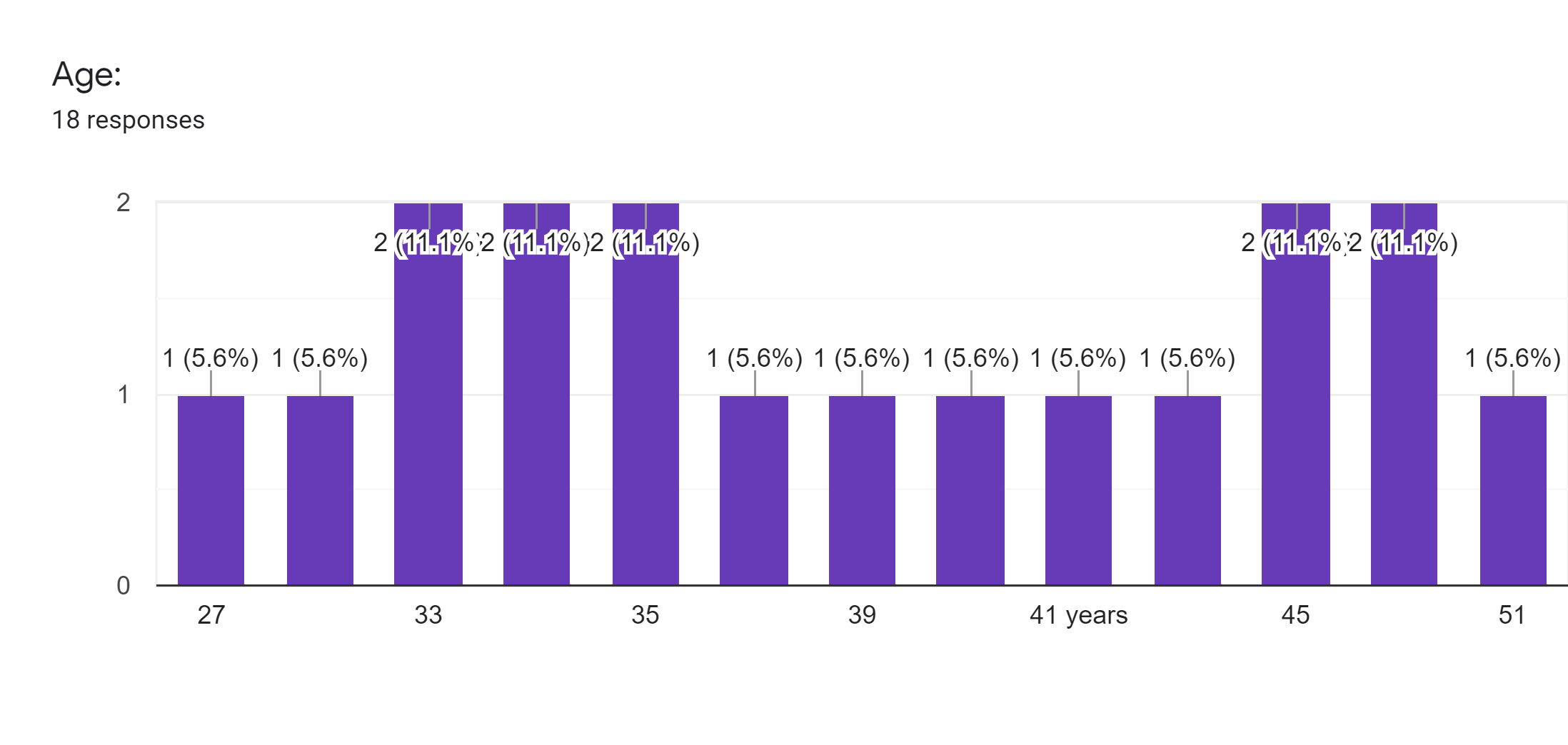


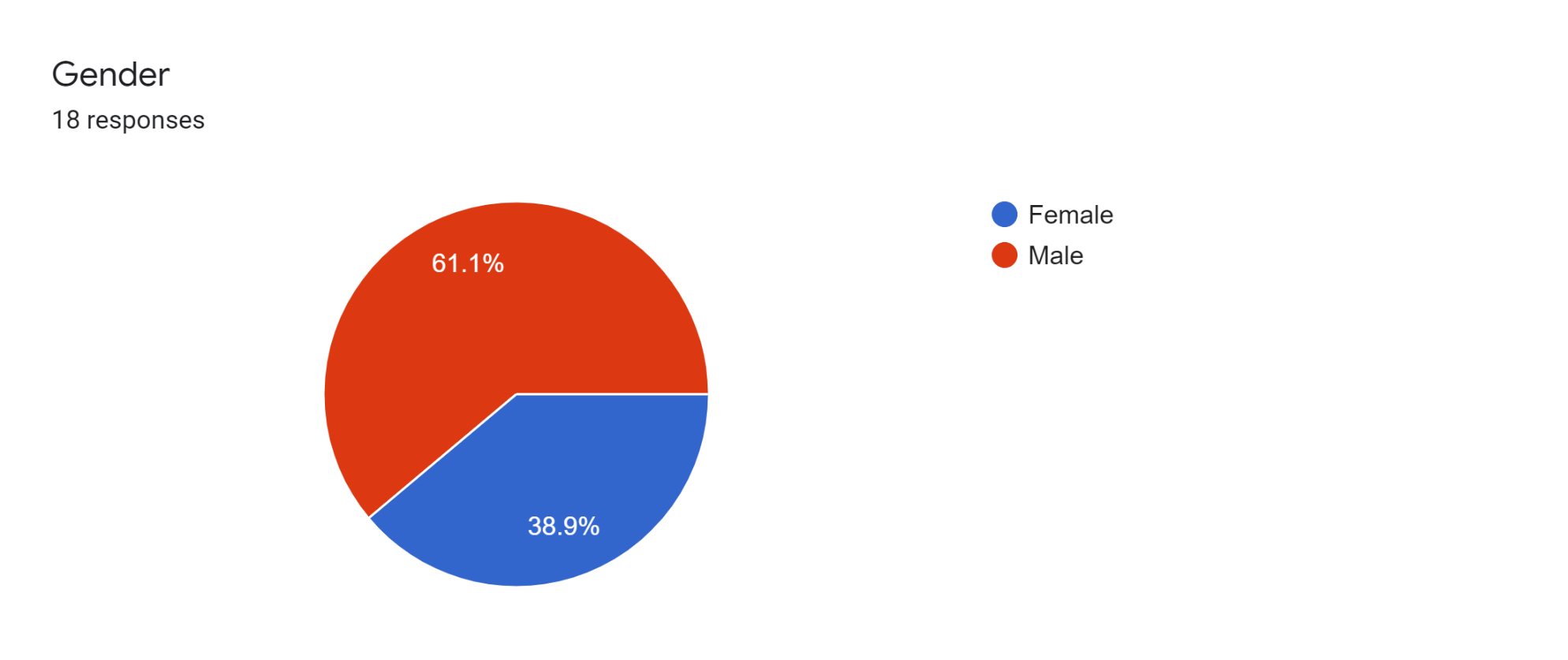


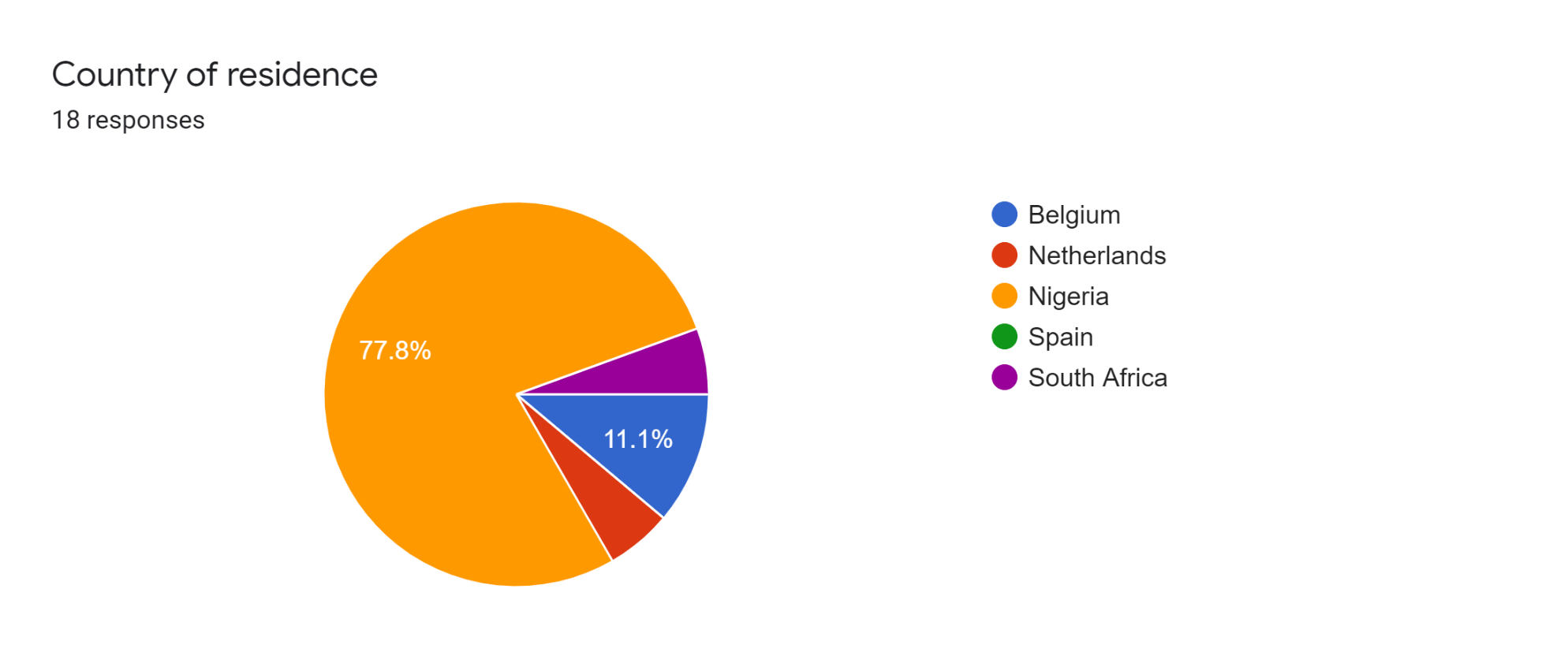


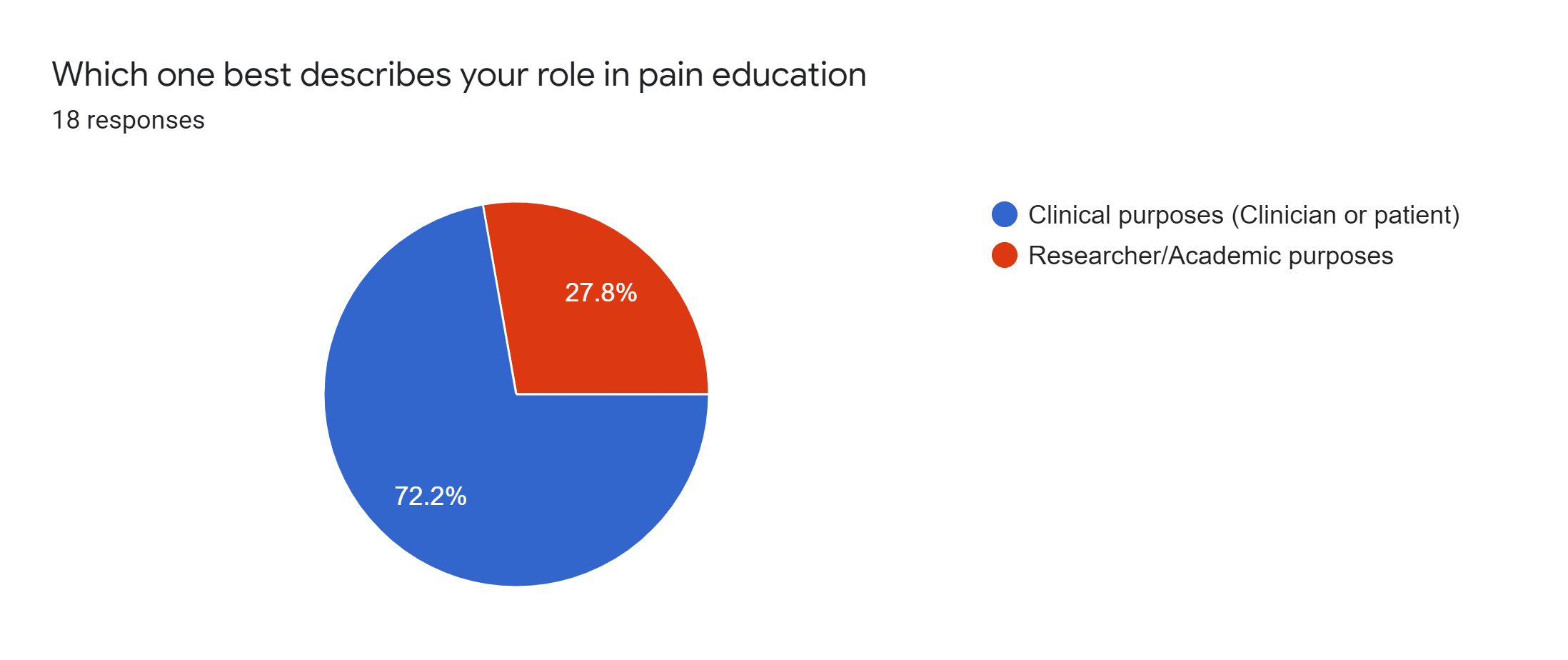


What is your experience with pain education?

18 responses

Non-existent 16.7%

Heard of it 5.6%

Familiar with it, <1 year 16.7%

Familiar with it, 1-5 years 27.8%

Familiar with it, 6-10 years 11.1%

Familiar with it, 11+ years 22.2%


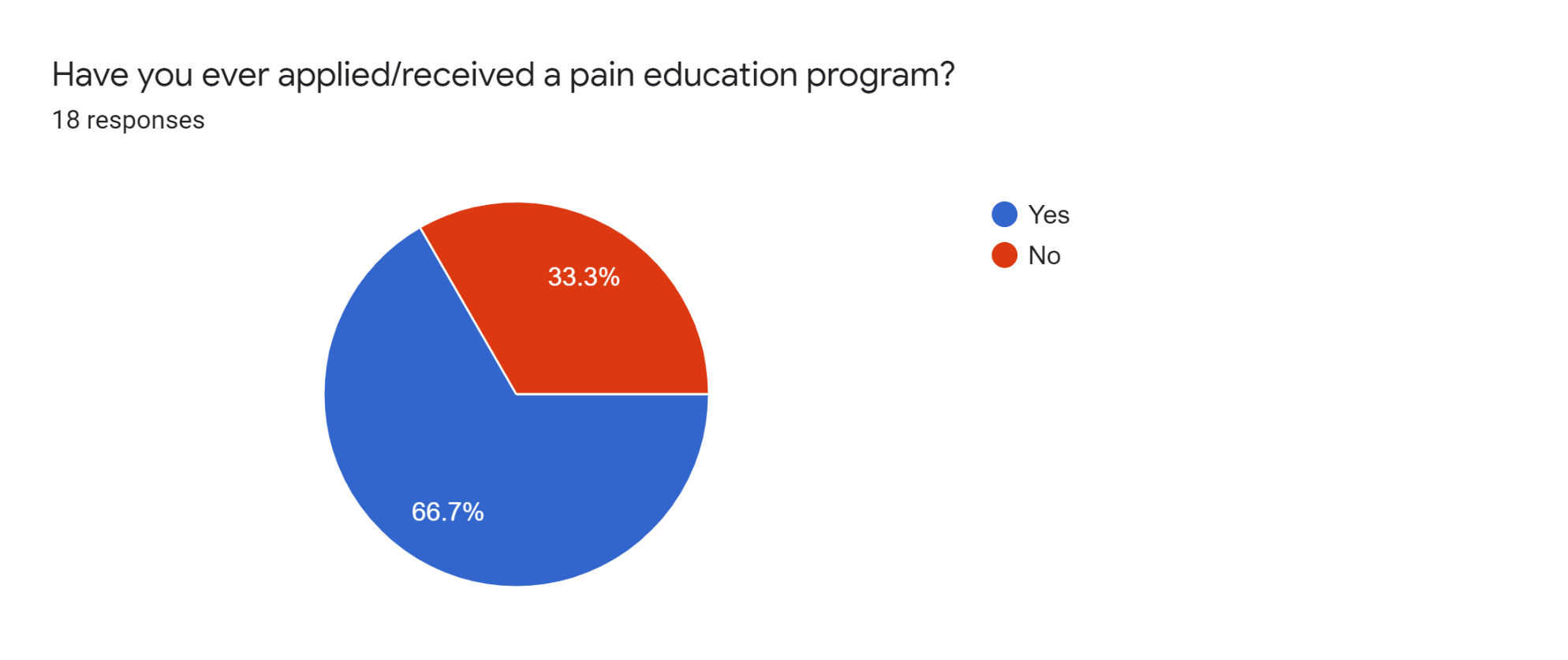


General


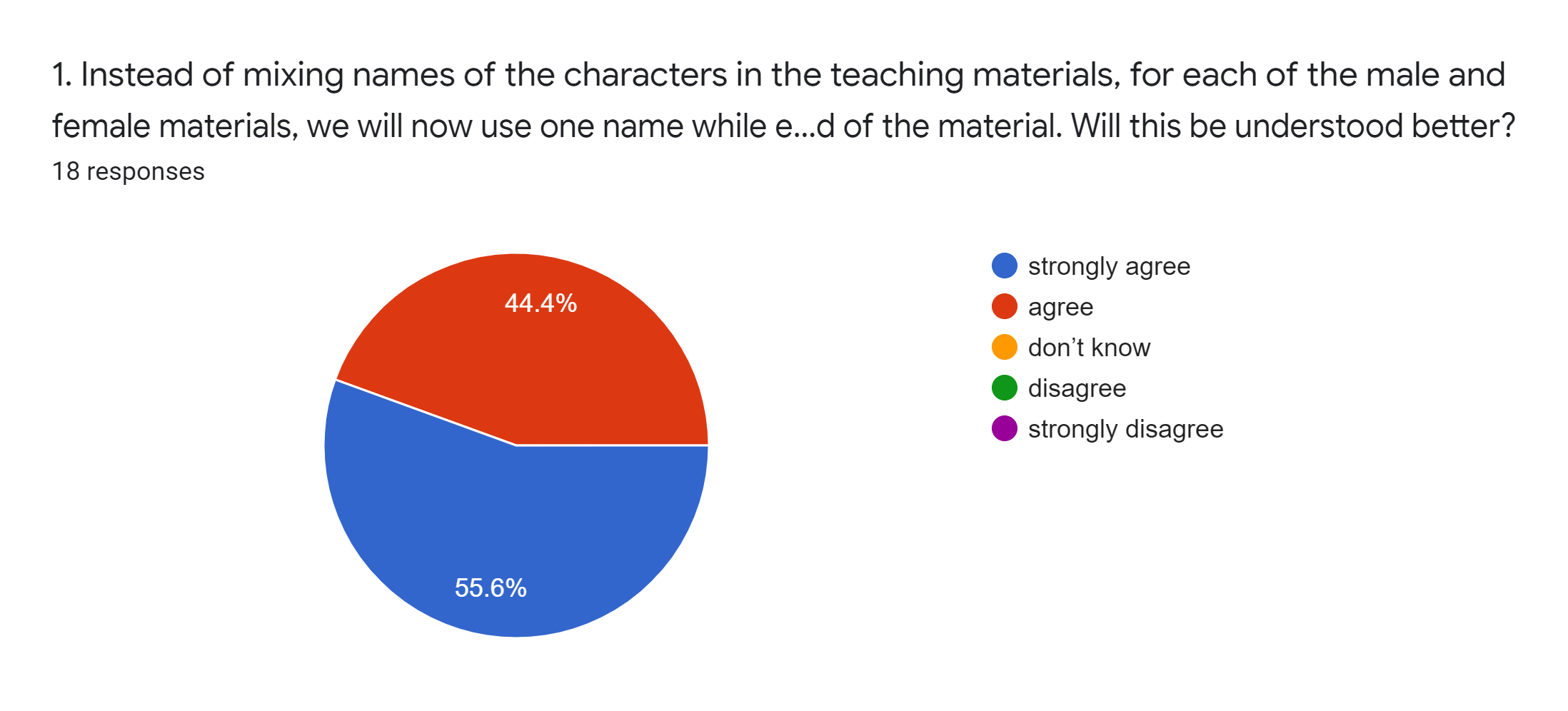

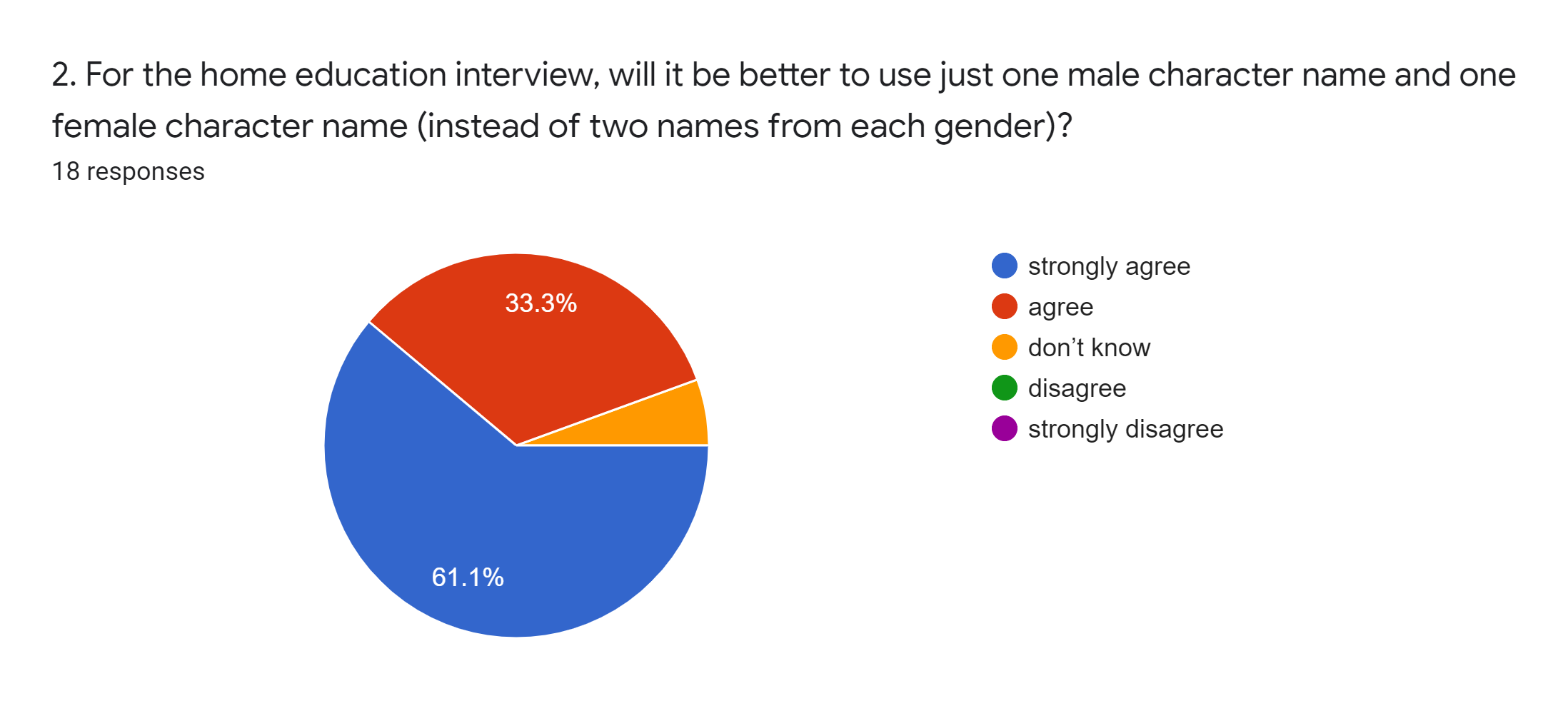


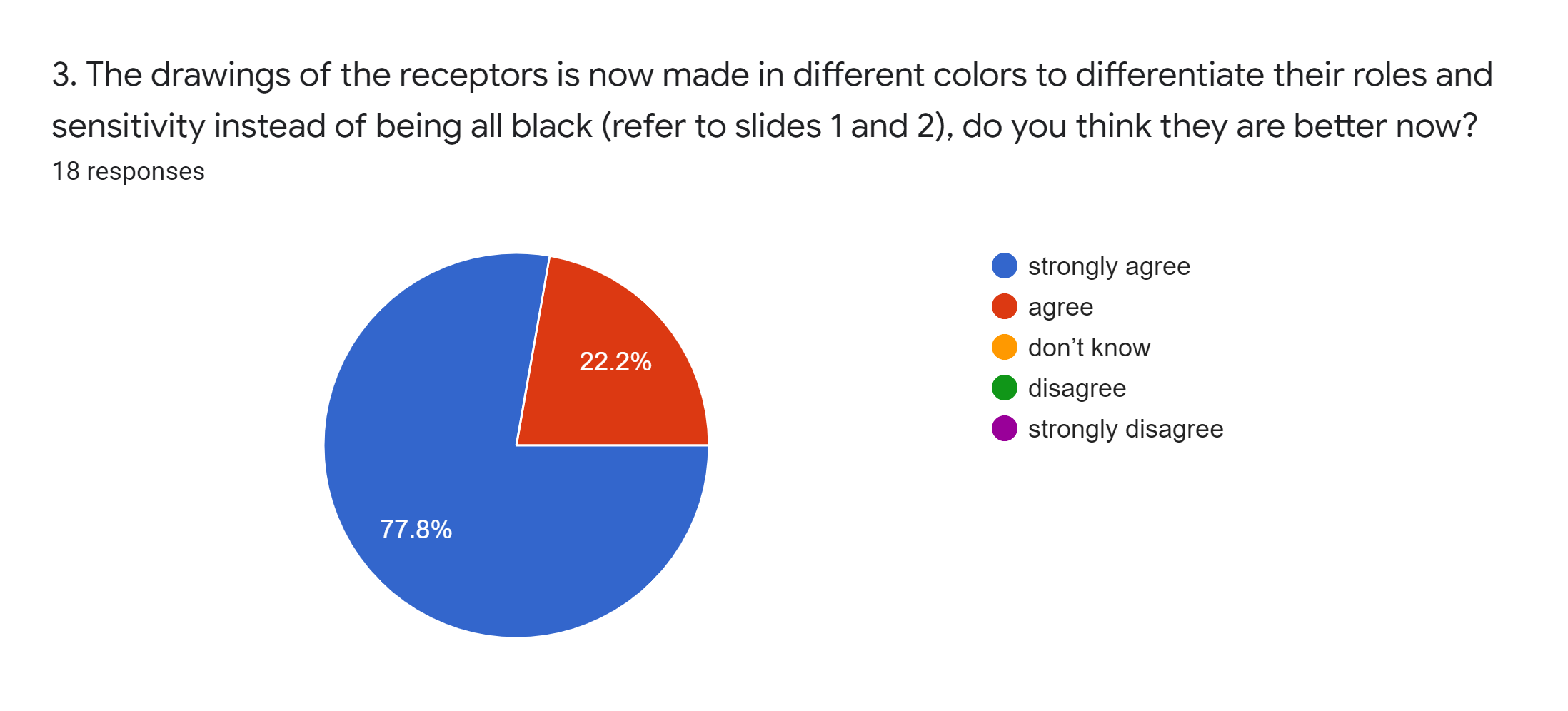


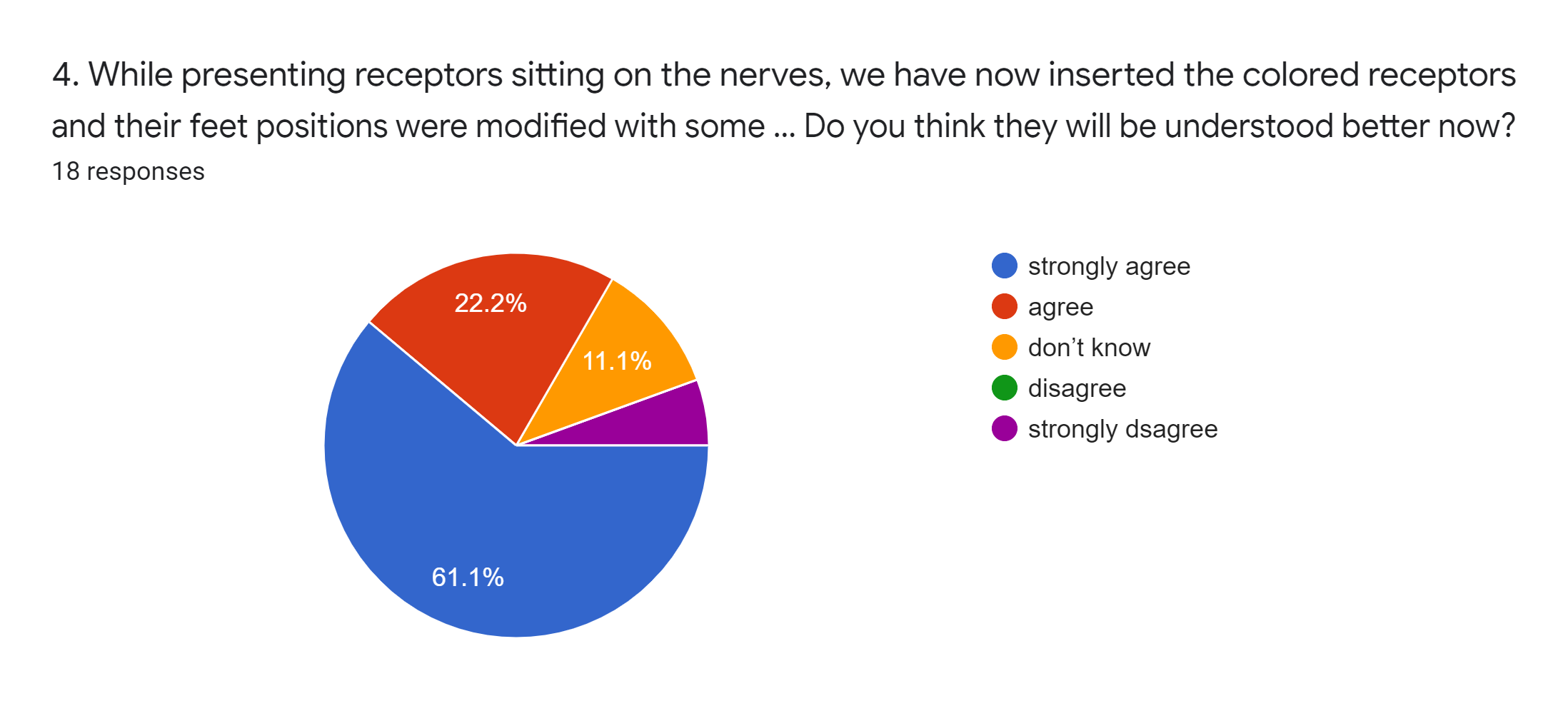


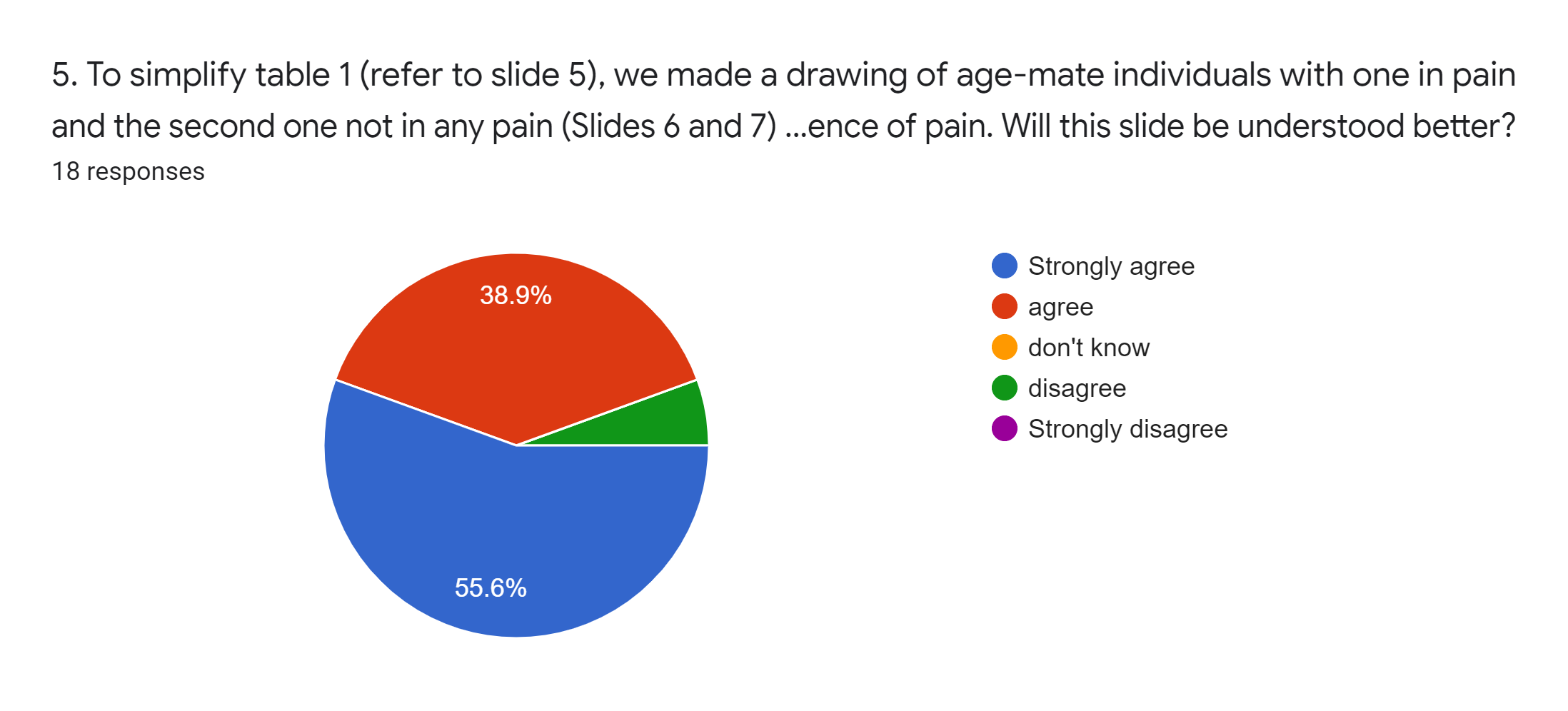


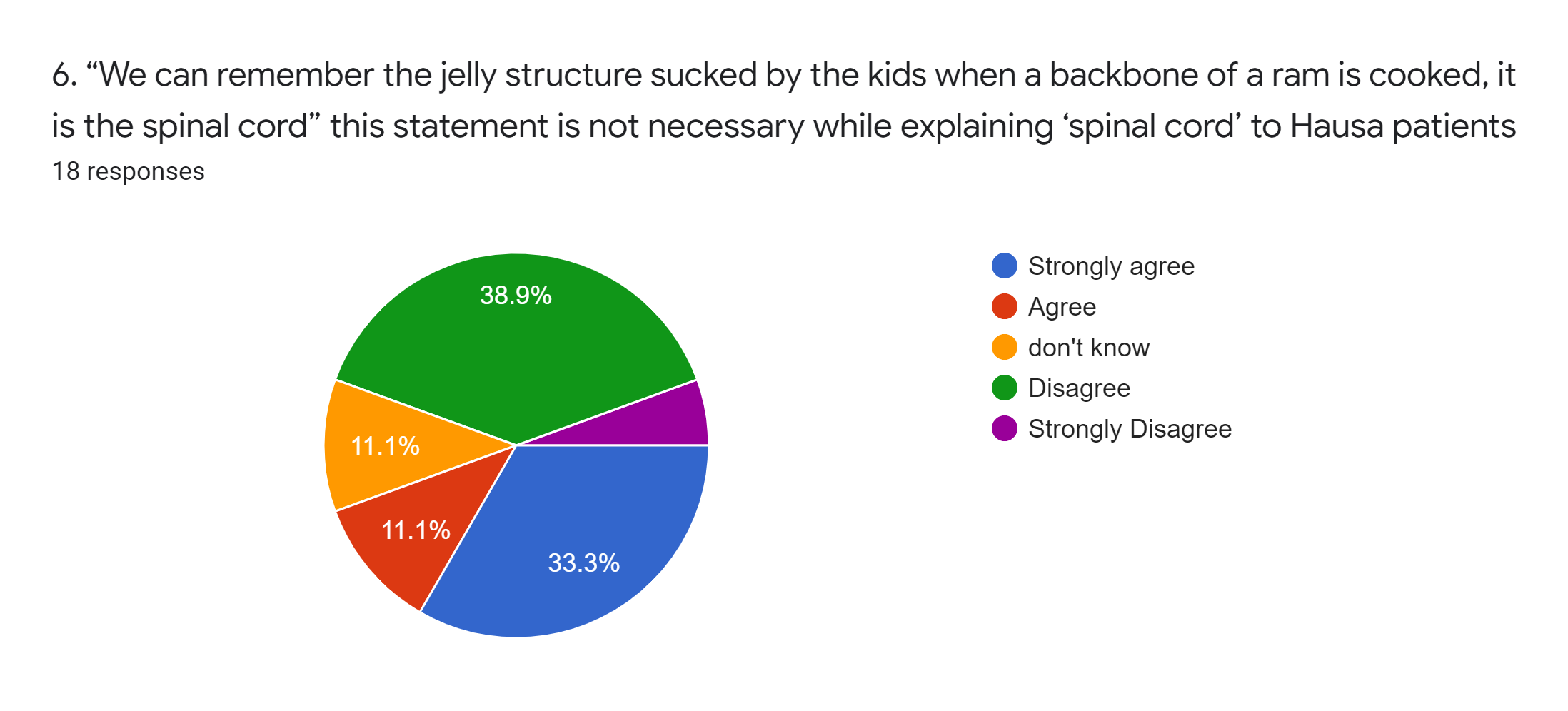


7. Do you have any other issue concerning the teaching materials? Say it in the box below15 responses

No

-

Slide 4: the symbol beside each head is not always present and clear; furthermore, I am really not sure the readers will pay attention to the feet closed or not. Finally, I do not understand why in this slide you "activate" different kind of receptors (feet open) at the same time and why the colours do not correspond to the previous slide. Slides 5-7: not sure the reader will understand what the cross in the table means: does it mean pain or no pain? Personally, I would systematically write "pain" and never "pains" but I might be wrong

On the Receptors on Nerves slides I'm worried about the statement "so they can easily detect any potentially painful stimulus" I really think this needs to be "detect any potentially dangerous stimulus" otherwise there is the risk that pain is placed in the peripheray

No. The modifications are very essentials and have improved the materials for better understanding.

Well explained and detailed

None

I think some people may not pay attention to feet positions in the slides 3 and 4, and the body attitude of age-mate individuals in slides 6 and 7 unless these are referred to in-text.

Make all pages colored

No. Almost everything is explained.

8. Do you have any other issue concerning the home education interview? Say it in the box below13 responses

No

-

No. Everything looks perfect. i wish the candidate a successful delivery and the program will be most beneficial to the population.

Well explained and detailed

Question 6 can be explain better, as is not everyone that can appreciate the explanation of sucking spinal cord, a better one is needed

No

None

It's very ok and good

No.
