# Supplementary material for: Development of culturally sensitive pain neuroscience education materials for Hausa-speaking patients with chronic spinal pain: A modified Delphi study
Source: PLoS One. 2021 Jul 2;16(7):e0253757. doi: 10.1371/journal.pone.0253757 (PMC8253446; doi:10.1371/journal.pone.0253757)
Supplement: Attachment — Submitted filename: PONE-D-20-32518_ Reviewers Comment.pdf [file pone.0253757.s010.pdf]

PLOS ONE

Development of culturally sensitive pain neuroscience education materials for African (Hausa) patients with chronic spinal pain: a modified Delphi study

--Manuscript Draft--

**Manuscript Number:**	PONE-D-20-32518
**Article Type:**	Research Article
**Full Title:**	Development of culturally sensitive pain neuroscience education materials for African (Hausa) patients with chronic spinal pain: a modified Delphi study
**Short Title:**	Pain neuroscience education materials for Hausa patients with chronic spinal pain
**Corresponding Author:**	Naziru Bashir Mukhtar<br>Bayero University Kano<br>Kano, NIGERIA
**Keywords:**	Chronic; Consensus; Culture; Delphi; Education; Focus group; Hausa; Pain Neuroscience; Spinal pain.
**Abstract:**	This study aimed to develop culturally sensitive pain neuroscience education (PNE) materials for Hausa-patients with chronic spinal pain (CSP). PNE is a program of teaching patients about pain that has gained considerable attention in research and is increasingly used during physical therapy for patients with chronic pain. It helps in decreasing pain, disability, fear-avoidance, pain catastrophization, movement restriction, and health care utilization among patients with chronic pain. However, existing PNE materials and their application are limited to few languages and cultures. Due to the variations in pain perceptions, beliefs, and related outcomes among different cultures and populations, culture-sensitive PNE materials addressing these outcomes are warranted. A focus-group discussion comprising 4 experts was used to adapt and develop preliminary PNE materials. Thereafter, an internet-based 3-round modified Delphi-study involving 22 experts ensued. Experts' consensus/recommendations concerning the content were used in modifying the PNE materials. Consensus was predefined as ≥75% level of (dis)agreement. Eighteen experts completed the Delphi rounds. Nineteen, 18 and 18 experts participated in rounds 1, 2 and 3 respectively, representing 86%, 94% and 100% participation rate respectively. Consensus agreement was reached in every round and content of the materials, including drawings, examples, figures and metaphors were adapted following the experts' suggestions. We therefore concluded that, culture-sensitive PNE materials for Hausa-patients with CSP were successfully produced and the present Delphi-study may provide a direction for further research in which the effects of culturally-sensitive PNE materials can be piloted among Hausa-patients with CSP.
**Order of Authors:**	Naziru Bashir Mukhtar
	Mira Meeus
	Ceren Gursen
	Jibril Mohammed
	Vincent Dewitte
	Barbara Cagnie
**Additional Information:**	
**Question**	**Response**
**Financial Disclosure**<br><br>Enter a financial disclosure statement that describes the sources of funding for the work included in this submission. Review the submission guidelines for detailed requirements. View published research	The author(s) received no specific funding for this work.

Powered by Editorial Manager® and ProduXion Manager® from Aries Systems Corporation

articles from *PLOS ONE* for specific examples.<br><br>This statement is required for submission and **will appear in the published article** if the submission is accepted. Please make sure it is accurate.<br><br>**Unfunded studies**<br>Enter: *The author(s) received no specific funding for this work.*<br><br>**Funded studies**<br>Enter a statement with the following details:<br>• Initials of the authors who received each award<br>• Grant numbers awarded to each author<br>• The full name of each funder<br>• URL of each funder website<br>• Did the sponsors or funders play any role in the study design, data collection and analysis, decision to publish, or preparation of the manuscript?<br>• **NO** - Include this sentence at the end of your statement: *The funders had no role in study design, data collection and analysis, decision to publish, or preparation of the manuscript.*<br>• **YES** - Specify the role(s) played.<br><br>* typeset	
**Competing Interests**<br><br>Use the instructions below to enter a competing interest statement for this submission. On behalf of all authors, disclose any competing interests that could be perceived to bias this work—acknowledging all financial support and any other relevant financial or non-financial competing interests.<br><br>This statement **will appear in the published article** if the submission is accepted. Please make sure it is accurate. View published research articles from *PLOS ONE* for specific examples.	The authors have declared that no competing interests exist.

**NO authors have competing interests**<br><br>Enter: *The authors have declared that no competing interests exist.*<br><br>**Authors with competing interests**<br><br>Enter competing interest details beginning with this statement:<br><br>*I have read the journal's policy and the authors of this manuscript have the following competing interests: [insert competing interests here]*<br><br>* typeset	
**Ethics Statement**<br><br>Enter an ethics statement for this submission. This statement is required if the study involved:<br><br>• Human participants<br>• Human specimens or tissue<br>• Vertebrate animals or cephalopods<br>• Vertebrate embryos or tissues<br>• Field research<br><br>Write "N/A" if the submission does not require an ethics statement.<br><br>General guidance is provided below. Consult the submission guidelines for detailed instructions. **Make sure that all information entered here is included in the Methods section of the manuscript.**	N/A

**Format for specific study types**<br><br>**Human Subject Research (involving human participants and/or tissue)**<br>• Give the name of the institutional review board or ethics committee that approved the study<br>• Include the approval number and/or a statement indicating approval of this research<br>• Indicate the form of consent obtained (written/oral) or the reason that consent was not obtained (e.g. the data were analyzed anonymously)<br><br>**Animal Research (involving vertebrate animals, embryos or tissues)**<br>• Provide the name of the Institutional Animal Care and Use Committee (IACUC) or other relevant ethics board that reviewed the study protocol, and indicate whether they approved this research or granted a formal waiver of ethical approval<br>• Include an approval number if one was obtained<br>• If the study involved *non-human primates*, add *additional details* about animal welfare and steps taken to ameliorate suffering<br>• If anesthesia, euthanasia, or any kind of animal sacrifice is part of the study, include briefly which substances and/or methods were applied<br><br>**Field Research**<br><br>Include the following details if this study involves the collection of plant, animal, or other materials from a natural setting:<br>• Field permit number<br>• Name of the institution or relevant body that granted permission	
**Data Availability**<br><br>Authors are required to make all data underlying the findings described fully available, without restriction, and from the time of publication. PLOS allows rare exceptions to address legal and ethical concerns. See the PLOS Data Policy and FAQ for detailed information.	Yes - all data are fully available without restriction

A Data Availability Statement describing where the data can be found is required at submission. Your answers to this question constitute the Data Availability Statement and **will be published in the article**, if accepted.<br><br>**Important:** Stating 'data available on request from the author' is not sufficient. If your data are only available upon request, select 'No' for the first question and explain your exceptional situation in the text box.<br><br>Do the authors confirm that all data underlying the findings described in their manuscript are fully available without restriction?	
**Describe where the data may be found in full sentences. If you are copying our sample text, replace any instances of XXX with the appropriate details.**<br><br>• If the data are **held or will be held in a public repository**, include URLs, accession numbers or DOIs. If this information will only be available after acceptance, indicate this by ticking the box below. For example: *All XXX files are available from the XXX database (accession number(s) XXX, XXX.*).<br>• If the data are all contained **within the manuscript and/or Supporting Information files**, enter the following: *All relevant data are within the manuscript and its Supporting Information files.*<br>• If neither of these applies but you are able to provide **details of access elsewhere**, with or without limitations, please do so. For example:<br><br>*Data cannot be shared publicly because of [XXX]. Data are available from the XXX Institutional Data Access / Ethics Committee (contact via XXX) for researchers who meet the criteria for access to confidential data.*<br><br>*The data underlying the results presented in the study are available from (include the name of the third party*	Data will be available on request

and contact information or URL).<br>• This text is appropriate if the data are owned by a third party and authors do not have permission to share the data.<br><br>* typeset	
Additional data availability information:	

Manuscript

Click here to access/download;Manuscript;Main Document Blinded .docx

Development of culturally sensitive pain neuroscience education materials for African (Hausa) patients with chronic spinal pain: a modified Delphi study

Naziru Bashir Mukhtar *[,1,2], Mira Meeus [1,3,4], Ceren Gursen [5], Jibril Mohammed [2], Vincent Dewitte [1], Barbara Cagnie [1]

[1] Department of Rehabilitation Sciences, Ghent University, Belgium
[2] Department of Physiotherapy, Bayero University Kano, Nigeria
[3] Pain in motion international research group, Belgium
[4] Department of Rehabilitation Sciences and Physiotherapy, University of Antwerp, Belgium
[5] Department of Physiotherapy and Rehabilitation, Hacettepe University, Turkey

*Corresponding author. Ghent University, Department of Rehabilitation Sciences, Corneel Heymanslaan 10, 9000 Ghent, Belgium. Phone number; +32 9 332 52 65, Fax number; 0032 9 332 38 11; email address; nazirubashir.mukhtar@ugent.be

Email addresses: nazirubashir.mukhtar@ugent.be (N.B. Mukhtar), mira.meeus@ugent.be (M. Meeus), cerengursen@yahoo.com (C. Gursen), jmohammed.pth@buk.edu.ng (J. Mohammed), vincent.dewitte@ugent.be (V. Dewitte), barbara.cagnie@ugent.be (B. Cagnie)

Abstract

This study aimed to develop culturally sensitive pain neuroscience education (PNE) materials for Hausa-patients with chronic spinal pain (CSP). PNE is a program of teaching patients about pain that has gained considerable attention in research and is increasingly used during physical therapy for patients with chronic pain. It helps in decreasing pain, disability, fear-avoidance, pain catastrophization, movement restriction, and health care utilization among patients with chronic pain. However, existing PNE materials and their application are limited to few languages and cultures. Due to the variations in pain perceptions, beliefs, and related outcomes among different cultures and populations, culture-sensitive PNE materials addressing these outcomes are warranted. A focus-group discussion comprising 4 experts was used to adapt and develop preliminary PNE materials. Thereafter, an internet-based 3-round modified Delphi-study involving 22 experts ensued. Experts' consensus/recommendations concerning the content were used in modifying the PNE materials. Consensus was predefined as ≥75% level of (dis)agreement. Eighteen experts completed the Delphi rounds. Nineteen, 18 and 18 experts participated in rounds 1, 2 and 3 respectively, representing 86%, 94% and 100% participation rate respectively. Consensus agreement was reached in every round and content of the materials, including drawings, examples, figures and metaphors were adapted following the experts' suggestions. We therefore concluded that, culture-sensitive PNE materials for Hausa-patients with CSP were successfully produced and the present Delphi-study may provide a direction for further research in which the effects of culturally-sensitive PNE materials can be piloted among Hausa-patients with CSP.

Keywords: Chronic; Consensus; Culture; Delphi; Education; Focus group; Hausa; Pain Neuroscience; Spinal pain.

Introduction

An educational program for teaching patients about pain has gained considerable attention in research [1-4] and refers to different terms such as Explain Pain [5], Therapeutic Neuroscience Education [3], and Pain Neuroscience Education (PNE) [6]. PNE teaches people in pain about the biology and physiology of their pain experience, including processes such as normal biology of pain, pain modulation, pain matrix, peripheral and central sensitization, allodynia, and neuroplasticity [7, 8]. There is growing evidence for the value of PNE in decreasing pain, disability, fear-avoidance, pain catastrophization, movement restriction, and health care utilization in people struggling with pain [7, 9, 10]. Consequently, PNE is increasingly used as part of physical-therapy for patients with chronic pain in clinical settings [4, 10, 11]. Moreover, in 2015, the International Association for the Study of Pain endorsed a ‘‘call to action’’ which recognized an urgent need for all countries to improve access to pain management [12].

The development and use of PNE is well-established among Caucasians [9, 13-17]. However, its access in many other world languages and cultures is lacking. In the last two decades, it has been reported that variations in culture, socioeconomic status, gender issues, and literacy levels have to be considered when developing educational tools for any population [18]. Additionally, patients’ beliefs are a core part of pain perception and response, as such response to pain is influenced both by patients’ beliefs about it and the emotional significance attributed to it [19]. A recent systematic review revealed evidence regarding the differences in pain beliefs, pain attitudes, and coping strategies among different cultures and ethnicities [20]. Hence, a few attempts have been made to culturally adapt the PNE materials. One of these attempts involved an internet-based method that requires internet-access, literacy, and at least a smartphone for access among Brazilians [12]. Another study among Turkish-immigrants in Belgium used home education (HE) material that required literacy to read, in addition to having Turkish-specific pictures and metaphors [21].

There is an urgent need to develop culturally-sensitive PNE materials for different cultures and ethnicities worldwide in order to increase access to PNE interventions for patients with chronic pain. Cultural sensitivity is defined by two dimensions: surface and deep structures. Surface structure involves matching intervention materials and messages to observable superficial characteristics of the target population which may involve using people, places, language, clothing, etc. that are familiar to and preferred by the target audience, whereas the deep structure encompasses the integration of cultural, social, historical, environmental, and psychological factors that influence the target health behavior in the proposed target population [22].

Neither the translation nor the culturally-sensitive version of PNE is available in any of the indigenous African languages. Africa has several indigenous languages, of which Hausa is among the most popular [23, 24]. Hausa is one of the leading African languages in terms of number of speakers. In addition, it is the unofficial lingua franca in the West-African region and studies have reported a varying number of Hausa native speakers between 30 to over 50 million [25, 26].

Additionally, the existing PNE materials mainly developed in Australia [5, 13, 27], Europe [14, 16, 17], and America [9] contain pictures, examples and metaphors that may not be appropriate for Hausa population, as some of the cases and scenarios do not relate to them due to differences in culture, religion, educational levels, or technological advancements. Furthermore, the conventional HE leaflets used by previous researchers [13-15, 17, 21] may not be feasible for Hausa population to read due to a high adult illiteracy rate that is estimated to be 43% [28]. The PNE materials developed for first-generation Turkish-immigrants living in Belgium [21] is the only one nearest to Hausa culture due to overlap in religion (Islam) and to some extent with regard to clothing and gender roles. Nevertheless, these materials also used a lot of pictures, metaphors and stories that are not available in a Hausa context. Nijs et al. [6] indicated that intellectual ability and health

literacy of a patient should be taken into account before using a PNE program. Consequently, the lower literacy rate compared to Western populations is another reason to develop culturally-sensitive PNE materials for Hausa-patients suffering from chronic spinal pain (CSP). Finally, Hausa communities also differ from many of the communities where the existing PNE materials were developed in terms of religious and gender issues [29].

Consequently, it has become clear that health care programs should be culturally-sensitive and not just a mere translation of the available materials [22]. The aim of the present study was to develop specific culturally-sensitive PNE Teaching Materials (TM) for Hausa women and men experiencing CSP, and general HE material by using a 2-phase sequential design of a focus group (FG) and modified Delphi-survey.

Materials and methods

Ethical approval: The research protocol was approved by the Hospital ethics committee of 'X' University.

Study design: A *focus group (FG) discussion* of experts followed by an internet based *3-round modified Delphi-survey*.

Procedure: First, a FG discussion was carried out to generate the preliminary materials that were used during the Delphi rounds [30-32], following a similar procedure as described by Orhan et al. [21]. Next, a 3-round modified Delphi-study was designed to gather inputs, corrections, and consensus of experts using a series of online questionnaires [21, 32, 33]. Informed consent was obtained from the experts.

Focus group: The preliminary PNE teaching and HE materials were developed through FG discussions. Three to 4 experts are endorsed in FG meetings when participants have specialized knowledge and/or experiences to discuss [34, 35] and 4 to 6 experts are becoming increasingly popular because smaller groups are easier to recruit, host and are more comfortable for participants [36]. Therefore, 4 experts were included in this FG discussion (these experts were different from those in the 'Delphi expert panel'). Since there is no limit to the number of meetings in FG, as it can vary from 1 to several meetings based on the researchers' need and saturation [37], this FG had 3 meetings and each meeting lasted for up to 1 hour [38].

Experts in FG meetings are individuals that generally have superior knowledge about the topic, but there is no clear definition for such expertise [39]. Therefore, all the 4 experts were physiotherapists that have experience in the PNE concept (with a minimum of 1 peer-reviewed publication in PNE).

In addition, 1 of the experts was a native Hausa language speaker. The first author (NBM) moderated the meetings and recorded all discussions. The content of the existing PNE teaching and HE materials [5, 21] was discussed during the FG meetings. The main message of 'Explain Pain' [5] was preserved. This content comprises the importance of pain, differences between acute and chronic pain, how pain originates in the nervous system, what makes pain to persist for long time, and the sensitivity of the nervous system [5]. For the purpose of explaining this, contents, pictures, drawings, metaphors, and stories were also discussed in relation to Hausa culture.

The FG resulted in the preliminary PNE teaching and HE materials to use in the first round of the Delphi-study. Next, the following major adaptations to the existing materials were performed: (i) the development of HE materials in form of an oral interview between an expert and a journalist, so that Hausa-patients can listen to the interview, since the majority of Hausa people cannot read [28]; (ii) modification of the pictures in the TM with drawings that depict African/Hausa people; (ii) due to cultural and religious peculiarities, separate PNE TM for male and female patients were developed [21]; (iv) metaphors, examples and stories that were thought to be inappropriate for Hausa-patients were changed or modified to fit the Hausa contexts.

The teaching and HE materials were produced in 2 languages; a Hausa version for Hausa speaking experts and an English version for experts that do not speak Hausa. Firstly, all the materials were developed in the English language and then translated into Hausa language by the first author who is fluent in English and a native Hausa speaker. The translated materials, together with the English materials, were sent to 2 Hausa language experts for corrections [21]. This translation procedure was used after each round of the Delphi-surveys. All drawings in the materials were performed by a professional artist based on the feedback of the experts.

Throughout the manuscript, *TM* refer to the PowerPoint slides developed for teaching patients about pain, whereas the *HE material* applies to the written script of the prospective oral interview that patients will listen at home to supplement what they have learned from the TM.

Delphi-study: The 3-rounds Delphi ran from May 2018 to November 2018, and were conducted according to the recommendation of guidance on conducting and reporting of Delphi-studies (CREDES) [40].

Delphi-experts: There is no existing guideline on who is an expert and how many experts should be recruited in a Delphi-study [41], but in this study, 4 key areas of expertise were defined; (1) PNE; (2) Hausa culture; (3) management of Hausa-patients with CSP; and (4) Hausa-patients with CSP. Therefore, the recruited experts were a combination of: (i) physiotherapists with at least an experience in PNE (with 1 published peer-reviewed paper on PNE) or Hausa culture (with one published peer-reviewed paper on Hausa cultural adaptation) or managing Hausa-patients with CSP (with at least 5-years of clinical experience); and (ii) Hausa-patients with CSP (with either neck or back pain that lasted for at least the previous 3 months). A total of 28 experts (with 7 experts from each of the 4 key expertise areas) were purposively sampled [32, 33] and invited to participate in the Delphi-surveys prior to round 1. Experts were identified and selected based on the network and personal contacts of the authors (NBM, MM, JM, BC).

Round 1: Two weeks before the start of the first-round, the participants were sent e-mails containing the PNE TM (male and female) for them to study. After these 2-weeks, the questionnaires consisting of both open and closed-ended questions in English language regarding the submitted material were sent. The open-ended questions provided the experts the freedom to

give any relevant feedback, while the closed-ended questions limited the responses to the PNE contexts, since many of the participants were not PNE experts.

Experts who had limited computer literacy were guided on how to fill in the online questionnaires by one of the researchers (JM), who has experience in online surveys. The experts and patients were asked to complete and submit the questionnaires within 2-weeks. To increase the adherence rate of the experts, a reminder via e-mail with an additional 2-weeks grace period was sent to those that were unable to complete the questionnaires within the initial 2-weeks.

The questionnaire for round 1 consisted of 7-items on demographics and a total of 42 (31 closed-ended and 11 open-ended) PNE materials-related questions that were adapted and modified from a previous study [21].

The questions were divided into 9 sections ("*x*") consisting of 'acute pain', 'pain biology', 'pain modulation', 'pain matrix', 'chronic pain', 'beliefs, thoughts and behaviors', 'central sensitization', 'implications', and 'general questions'. In each of the above mentioned sections (except the 'general questions'), the following 4 multiple choice questions were asked using a 5-point Likert scale, (strongly agree – agree - don't know – disagree - strongly disagree) [42]:

i. *Do you think that these slides/pages provide relevant information about "x" (each section bears its name)?*

ii. *Do you think that the stories used to describe the "x" are feasible for the Hausa population?*

iii. *Do you think that visual information (pictures) for "x" in the TM is feasible for Hausa-patients?*

iv. *Do you think the message is clear and patients will understand?*

At the end of each section, an open-ended question was asked:

i. *If you have any suggestion(s) regarding the description of "x", please write them in the box below.*

Three final and open-ended general questions were asked:

i. *What do you think about the order and the concept? Is it understandable, logical?*
ii. *General remarks?*
iii. *Further suggestions?*

The consensus level was predefined at ≥75% [43], which is the minimum consensus required for a decision upon the content. When 75% or more of the experts choose to 'agree' or 'strongly agree', then the content was retained. If 75% or more of the experts choose to 'disagree' or 'strongly disagree', the content was rejected. The content was subsequently modified based on the experts' suggestions and resubmitted for the next round. In the first round, only the 2 TM (male and female) were sent to experts to minimize participation fatigue and drop-outs due to bulkiness of the materials.

Round 2: In this round, the modified TM together with the HE material were sent to all Delphi-experts that participated in round 1. In case of consensus regarding the inappropriateness of an item in the TM in round 1, the items were modified or removed in both the teaching and HE materials prior to sending them for round 2. Two different online questionnaires were sent to the experts in this round: one for the HE material and another for the modified TM. Two weeks were given (plus two weeks grace) to the experts to complete and submit the questionnaires.

The questions for the HE materials were similar with that of the TM in round 1. Except for the 'pain modulation' and 'central sensitization' sections that were not included (since these 2 sections

were not included in the HE material), instead, an ‘introduction’ section was included. The HE material questionnaire contained a total of 35 (25 closed-ended and 10 open-ended) questions. The questionnaire for the revised TM comprised a total of 31 (23 closed-ended and 8 open-ended) questions about changes/modifications done in the following sections ‘acute pain’, ‘pain biology’, ‘pain modulation’ and ‘beliefs, thoughts and behaviors’, because experts suggested for that.

Additionally, closed-ended questions were asked concerning the male and female TM after the update:

i. *Do you think the updated material provides more relevant ‘a=information, b=pictures, c=stories and metaphors’ for educating Hausa-patients about pain than the round 1 material?*
ii. *Do you think the message in the updated material is clear and patients will understand?*

All the questions above were graded using the same 5-point Likert scale as in round 1 and the consensus level was maintained at ≥75%.

Three general open-ended questions concerning the updated material were added: *Is there anything*

i. *You would like us to add to this TM?*
ii. *Specific that you would like us to modify or simplify again in this TM?*
iii. *You will further suggest?*

Round 3: After qualitatively analyzing the responses of the experts from Round 2, questions were asked to finalize the development of the materials. Six closed-ended and 2 open-ended questions were asked to finalize this round. The questions had the usual 5-point Likert scale as in the previous rounds. The questions and responses are presented in the Results section (Table 6). This round was completed within 2-weeks.

Closure: After round 3, the final teaching and HE materials were updated and developed (see supplements; I, II, III for the Hausa materials and IV, V & VI for the English versions). The finalized materials were sent to the experts along with an appreciation message for participation. The HE interview was then orally conducted and recorded (supplement III) between a professional Hausa journalist and the first author (NBM).

Data analysis: Content analysis [32, 44] was used to qualitatively analyze the data of each round. The first, second and last authors independently analyzed the comments of the experts. Based on the comments and suggestions of the experts, the authors identified the topics in relation to the comments of the experts, which enabled the authors to effect the necessary modifications and corrections on the materials

Results

Four experts who are all physiotherapists participated in the FG discussion (3 PhDs and 1 MSc holders). Although 28 experts were invited to participate in the Delphi-study, only 22 agreed. Nineteen out of 22, 18 out of 19, and all 18 experts participated in the rounds 1, 2 and 3 respectively, representing 86%, 94%, and 100% participation rate for each round. Five PNE experts, 5 CSP patients, 4 Hausa culture experts and 4 physiotherapists managing CSP patients completed round 3. The demographic characteristics of the experts in each round are presented in Table 1, while the participation flowchart can be seen in Figure 1.

In all 3 rounds, the experts were predominantly males (61.1-68.4%), resident in Nigeria (73.7-77.4%), and most had a clinical role in PNE (66.7-77.2%). Among the experts, a significant proportion had either applied or received a PNE program (52-66.7%), while 21% of them were not familiar with PNE at all.

Round 1: Table 2 shows the responses of the experts to the closed-ended questions during round 1. For all the content of the materials, the experts reached a consensus of ≥75%, and as such, the content was retained. However, the experts made suggestions for changes through the open-ended questions. The experts' responses to the open-ended questions of round 1 are presented in Table 3. Since the materials were developed for Hausa patients, suggestions from experts that were not familiar with the Hausa culture that contradict suggestions of experts familiar with Hausa culture about the feasibility/cultural context of content were not used for modification of the materials. Most of the suggestions and changes that were made were related to drawings (change or modification to fit the Hausa culture) and the simplification of information, e.g. giving an explanation of what a spinal cord is and giving some additional content like maladaptive beliefs.

Round 2: In Table 4, the responses of the experts to closed-ended questions during round 2 are presented. In this round, the revised TM (male and female) and the HE material were reviewed by the experts. The experts reached a consensus of ≥75% for the entire content, with some parts reaching complete consensus (100%). Therefore, additional changes in content were only done in response to the open-ended questions.

Table 5 presents the responses to the open-ended questions of experts in round 2, but suggestions requiring the use of video/animations were not considered because of resource limitations and the low technological advancements among the end-users.

Round 3: The results of the closed and open-ended questions in the final round are presented in Table 6. Consensus was attained in all the closed-ended questions except in one item (see the Table 6 for details).

Discussion

The aim of the study was to develop culturally-sensitive PNE materials for Hausa-patients with CSP. Preliminary teaching and HE materials were developed through a FG discussion and subsequently the final materials were developed through a 3-round Delphi-study. The TM were adapted and modified from Orhan et al. [21], who conducted a similar study among Turkish-immigrants in Belgium, whose culture is considered to be the closest to a Hausa culture among all the culturally adapted PNE materials. In this study, an audio interview was developed as HE material, given the low literacy level in that region [28]. This is supported by a previous study [11] that recommended repetitions of PNE, in different forms (verbal or other) as it helps patients to understand the theory of neurophysiology.

The PNE materials developed are so far the first culturally-sensitive materials for any of the African culture. To our knowledge, this is the first study that has tried to provide HE materials that can be used by the non-literates. This as necessary because the previous PNE materials were developed for populations that, as reports by UNESCO shows, have higher literacy level than the general Hausa population [28]. Therefore, this development is in-line with the recommendations for improving access to pain management for all [12]. Although data on Hausa people being frequent listeners of audio talks is lacking, a previous study has reported as high as 89% of Nigerians to own an audio listening set [45]. This will assist the listening of the HE interview among Hausa patients.

Similarly, the development of the separate male and female TM has covered the importance attached to gender variations among Hausa people due to religion and culture. Varied gender treatment and differences exist in Hausa context, e.g. preventing girls from attending school; withdrawing girl-children from school; using girls for street hawking; and unequal treatment of

children by the parent [46]. Moreover, a previous review has reported variation of pain perception, emotion and understanding between males and females among different cultures [47], as such, a uniform material for both males and females may not be appropriate. Both the teaching and HE materials bear names, examples, metaphors, and drawings/pictures that are available and familiar for Hausa-patients which is in conformity with the concept of cultural sensitivity [22].

During round 1, the experts reached the minimum consensus level in almost all the closed-ended questions related to the TM. This may be a consequence of the adapted materials being modified [21] in relation to the religion of the Hausa people, and to some extent their culture. The experts' responses to the open-ended questions during this round were suggestive of changes of some pictures and drawings to fit the culture and also simplification of some specific language terminologies used, which are very vital for the development of any culture sensitive tool [22].

The experts' consensus level increased in round 2, with 100% of experts agreeing on most of the content of the materials. This could indicate that the materials in round 2 were better accepted by the experts compared to the prior materials. Obviously, this trend is aimed for in tool development using experts' opinions [48, 49]. During round 2, there were some suggestions regarding the use of videos and animations, but such additions were not included. The additions may not be appropriate for Hausa-patients due to low technological advancement, low literacy level, and high poverty among the target population [28, 50].

During the final round, the experts reached consensus on all the contents of the teaching and the HE materials and there were no suggestions that warranted an additional round. The researchers considered the TM ready for applications and the HE material was then recorded into the oral interview by the first author and a professional Hausa journalist.

Conclusion: The authors therefore concluded that, PNE materials that could be used to teach Hausa-patients with CSP and an audio interview that Hausa-patients can listen to at home, were successfully developed, following a well-documented, consensus building procedure. Considering the composition of the expert panel that participated in the development (i.e. physiotherapists that are experts in PNE, Hausa culture, and management of Hausa-patients with CSP, supplemented with the Hausa-patients with CSP themselves), the materials hold the promise to have high face validity and to be user-friendly.

Practice implication: The present Delphi-study may provide a direction for further research in which the effects of culturally-sensitive PNE materials can be piloted among Hausa-patients with CSP.

Limitations:

There was variation in the language of the experts. Therefore, the experts that did not speak Hausa language had to study the English version of the document.

The experts recruited were predominantly Nigerians and this is because Hausa people are predominantly found in Nigeria. Additionally, some of the patients recruited were not computer literate and not fluent in English language. Consequently, a research-assistant had to guide them on how to respond. However, the research-assistant was told not to influence their choices.

Conflicts of interest:

Authors declared no conflict of interest.

Funding:

This research did not receive any specific grant from funding agencies in the public, commercial, or not-for-profit sectors.

Acknowledgement:

The authors would like to thank; the experts/patients for their participation, Prof YMA and Dr AS both of the department of ‘Z’ languages (‘Q’ University) for their time in proofreading and reviewing the translation of the materials.

REFERENCES

1. Louw A, Puentedura EJ, Zimney K, Cox T, Rico D. The clinical implementation of pain neuroscience education: A survey study. Physiother Theory Pract. 2017;33(11):869-79. Epub 2017/08/19. doi: 10.1080/09593985.2017.1359870. PubMed PMID: 28820626.

2. van Ittersum MW, van Wilgen CP, van der Schans CP, Lambrecht L, Groothoff JW, Nijs J. Written pain neuroscience education in fibromyalgia: a multicenter randomized controlled trial. Pain practice : the official journal of World Institute of Pain. 2014;14(8):689-700. Epub 2013/11/21. doi: 10.1111/papr.12137. PubMed PMID: 24251724.

3. Zimney K, Louw A, Puentedura EJ. Use of Therapeutic Neuroscience Education to address psychosocial factors associated with acute low back pain: a case report. Physiother Theory Pract. 2014;30(3):202-9. Epub 2013/11/21. doi: 10.3109/09593985.2013.856508. PubMed PMID: 24252071.

4. Louw A, Puentedura EJ, Zimney K, Schmidt S. Know Pain, Know Gain? A Perspective on Pain Neuroscience Education in Physical Therapy. J Orthop Sports Phys Ther. 2016;46(3):131-4. Epub 2016/03/02. doi: 10.2519/jospt.2016.0602. PubMed PMID: 26928735.

5. Butler DS, Moseley GL. Explain Pain 2nd Edn: Noigroup publications; 2013.

6. Nijs J, Meeus M, Cagnie B, Roussel NA, Dolphens M, Van Oosterwijck J, et al. A modern neuroscience approach to chronic spinal pain: combining pain neuroscience education with cognition-targeted motor control training. Physical therapy. 2014;94(5):730-8.

7. Louw A, Nijs J, Puentedura EJ. A clinical perspective on a pain neuroscience education approach to manual therapy. J Man Manip Ther. 2017;25(3):160-8. Epub 2017/07/12. doi: 10.1080/10669817.2017.1323699. PubMed PMID: 28694679; PubMed Central PMCID: PMCPMC5498797.

8. Moseley GL, Butler DS. Fifteen years of explaining pain: the past, present, and future. The Journal of Pain. 2015;16(9):807-13.

9. Louw A, Diener I, Landers MR, Puentedura EJ. Preoperative pain neuroscience education for lumbar radiculopathy: a multicenter randomized controlled trial with 1-year follow-up. Spine. 2014;39(18):1449-57. Epub 2014/05/31. doi: 10.1097/BRS.0000000000000444. PubMed PMID: 24875964.

10. Louw A, Zimney K, Puentedura EJ, Diener I. The efficacy of pain neuroscience education on musculoskeletal pain: A systematic review of the literature. Physiother Theory Pract. 2016;32(5):332-55. Epub 2016/06/29. doi: 10.1080/09593985.2016.1194646. PubMed PMID: 27351541.

11. Wijma AJ, van Wilgen CP, Meeus M, Nijs J. Clinical biopsychosocial physiotherapy assessment of patients with chronic pain: The first step in pain neuroscience education. Physiother Theory Pract. 2016;32(5):368-84. Epub 2016/06/29. doi: 10.1080/09593985.2016.1194651. PubMed PMID: 27351769.

12. Reis FJJ, Bengaly AGC, Valentim JCP, Santos LC, Martins EF, O'Keeffe M, et al. An E-Pain intervention to spread modern pain education in Brazil. Braz J Phys Ther. 2017;21(5):305-6. Epub 2017/09/03. doi: 10.1016/j.bjpt.2017.06.020. PubMed PMID: 28864352; PubMed Central PMCID: PMCPMC5628361.

13. Moseley L. Combined physiotherapy and education is efficacious for chronic low back pain. Australian journal of physiotherapy. 2002;48(4):297-302.

14. Meeus M, Nijs J, Van Oosterwijck J, Van Alsenoy V, Truijen S. Pain physiology education improves pain beliefs in patients with chronic fatigue syndrome compared with pacing and self-management education: a double-blind randomized controlled trial. Archives of physical medicine and rehabilitation. 2010;91(8):1153-9.

15. Van Oosterwijck J, Meeus M, Paul L, De Schryver M, Pascal A, Lambrecht L, et al. Pain physiology education improves health status and endogenous pain inhibition in fibromyalgia: a double-blind randomized controlled trial. The Clinical journal of pain. 2013;29(10):873-82.
16. Pires D, Cruz EB, Caeiro C. Aquatic exercise and pain neurophysiology education versus aquatic exercise alone for patients with chronic low back pain: a randomized controlled trial. Clinical rehabilitation. 2015;29(6):538-47.
17. Téllez-García M, de-la-Llave-Rincón AI, Salom-Moreno J, Palacios-Ceña M, Ortega-Santiago R, Fernández-de-las-Peñas C. Neuroscience education in addition to trigger point dry needling for the management of patients with mechanical chronic low back pain: a preliminary clinical trial. Journal of bodywork and movement therapies. 2015;19(3):464-72.
18. Lasch KE, Wilkes G, Montuori LM, Chew P, Leonard C, Hilton S. Using focus group methods to develop multicultural cancer pain education materials. Pain management nursing : official journal of the American Society of Pain Management Nurses. 2000;1(4):129-38. Epub 2001/11/17. doi: 10.1053/jpmn.2000.18743. PubMed PMID: 11709866.
19. Main CJ, Foster N, Buchbinder R. How important are back pain beliefs and expectations for satisfactory recovery from back pain? Best practice & research Clinical rheumatology. 2010;24(2):205-17.
20. Orhan C, Van Looveren E, Cagnie B, Mukhtar NB, Lenoir D, Meeus M. Are pain beliefs, cognitions, and behaviors influenced by race, ethnicity, and culture in patients with chronic musculoskeletal pain: A systematic review. Pain physician. 2018;21(6):541-58.
21. Orhan C, Cagnie B, Favoreel A, Van Looveren E, Akel U, Mukhtar NB, et al. Development of culturally sensitive Pain Neuroscience Education for first-generation Turkish patients with chronic pain: A modified Delphi study. Musculoskeletal Science and Practice. 2019;39:1-9.
22. Resnicow K, Baranowski T, Ahluwalia JS, Braithwaite RL. Cultural sensitivity in public health: defined and demystified. Ethnicity & disease. 1999;9(1):10-21.
23. Lodhi AY. The Language Situation in Africa Today. Nordic Journal of African Studies. 1993;2(1):8.
24. Ouane A, Glanz C. Why and how Africa should invest in African languages and multilingual education; An evidence- and practice-based policy advocacy brief. Hamburg, Germany: UNESCO 2010 Contract No.: 978-92-820-1171-3.
25. ECOWAS. Atlas on Regional Integration in West Africa. ECOWAS, 2006 October. Report No.
26. Gleich U, Wolff E. STANDARDIZATION OF NATIONAL LANGUAGES. Unesco Institute for Education, Hamburg: UNESCO, (UIE) UIfE; 1991.
27. Moseley GL. Evidence for a direct relationship between cognitive and physical change during an education intervention in people with chronic low back pain. European journal of pain. 2004;8(1):39-45.
28. UNESCO AP. Reaching the 2015 Literacy Target: Delivering on the promise. High Level International Round Table on Literacy, UNESCO, Paris. 2012:6-7.
29. Pittin R. Selective education: Issues of gender, class and ideology in northern Nigeria. Review of African political economy. 1990;17(48):7-25.
30. Gibbs A. Focus groups. Social research update. 1997;19(8):1-8.
31. Hasson F, Keeney S, McKenna H. Research guidelines for the Delphi survey technique. Journal of advanced nursing. 2000;32(4):1008-15.
32. Dewitte V, De Pauw R, De Meulemeester K, Peersman W, Danneels L, Bouche K, et al. Clinical classification criteria for nonspecific low back pain: A Delphi-survey of clinical experts. Musculoskeletal Science and Practice. 2018;34:66-76.
33. Smart KM, Blake C, Staines A, Doody C. Clinical indicators of 'nociceptive','peripheral neuropathic'and 'central'mechanisms of musculoskeletal pain. A Delphi survey of expert clinicians. Manual therapy. 2010;15(1):80-7.

34. Krueger RA. Focus groups: A practical guide for applied research: Sage publications; 2014.
35. Kitzinger J. Qualitative research: introducing focus groups. BMJ (Clinical research ed). 1995;311(7000):299-302.
36. Krueger RA, Casey MA. Designing and conducting focus group interviews. St Paul, Minnesota, USA; 2002.
37. Onwuegbuzie AJ, Dickinson WB, Leech NL, Zoran AG. A qualitative framework for collecting and analyzing data in focus group research. International journal of qualitative methods. 2009;8(3):1-21.
38. De Cocker K, Veldeman C, De Bacquer D, Braeckman L, Owen N, Cardon G, et al. Acceptability and feasibility of potential intervention strategies for influencing sedentary time at work: focus group interviews in executives and employees. International Journal of Behavioral Nutrition and Physical Activity. 2015;12(1):22.
39. Nagle B, Williams N. Methodology brief: Introduction to focus groups. Center for Assessment, Planning and Accountability. 2013;(1-12).
40. Jünger S, Payne SA, Brine J, Radbruch L, Brearley SG. Guidance on Conducting and REporting DElphi Studies (CREDES) in palliative care: recommendations based on a methodological systematic review. Palliative Medicine. 2017;31(8):684-706.
41. Luedtke K, Boissonnault W, Caspersen N, Castien R, Chaibi A, Falla D, et al. International consensus on the most useful physical examination tests used by physiotherapists for patients with headache: A Delphi study. Manual therapy. 2016;23:17-24.
42. Jamieson S. Likert scales: how to (ab) use them. Medical education. 2004;38(12):1217-8.
43. Cook C, Brismée J-M, Fleming R, Sizer Jr PS. Identifiers suggestive of clinical cervical spine instability: a Delphi study of physical therapists. Physical Therapy. 2005;85(9):895-906.
44. Patton MQ. Enhancing the quality and credibility of qualitative analysis. Health services research. 1999;34(5 Pt 2):1189.
45. Nwuneli O, Okoye I, Okunna C, Ayo J. Media use, knowledge of world affairs and image of nations among Nigerian youth. Africa Media Review. 1993;7(1):33-49.
46. Sada IN, Adamu FL, Ahmad A. Promoting women's rights through sharia in northern Nigeria. 2006.
47. Miller C, Newton SE. Pain perception and expression: the influence of gender, personal self-efficacy, and lifespan socialization. Pain Management Nursing. 2006;7(4):148-52.
48. Powell C. The Delphi technique: myths and realities. Journal of advanced nursing. 2003;41(4):376-82.
49. Keeney S, Hasson F, McKenna HP. A critical review of the Delphi technique as a research methodology for nursing. International journal of nursing studies. 2001;38(2):195-200.
50. Yusuf OB, Adeoye BW, Oladepo OO, Peters DH, Bishai D. Poverty and fever vulnerability in Nigeria: a multilevel analysis. Malaria Journal. 2010;9(1):235.

Figure Legend

Figure 1: Flow chart of the experts' recruitment and participation for the Delphi study

Supporting information captions

Supplement I: Pain education teaching slides (for males) in Hausa language

Supplement II: Pain education teaching slides (for females) in Hausa language

Supplement III: Home education audio interview in Hausa language

Supplement IV: Pain education teaching slides (for males) in English language

Supplement V: Pain education teaching slides (for females) in English language

Supplement VI: Home education audio interview (transcript) in English language

 Click here to access/download;Figure;Figure 1 .docx

Fig 1. Flow chart of the experts' recruitment and participation for the Delphi study

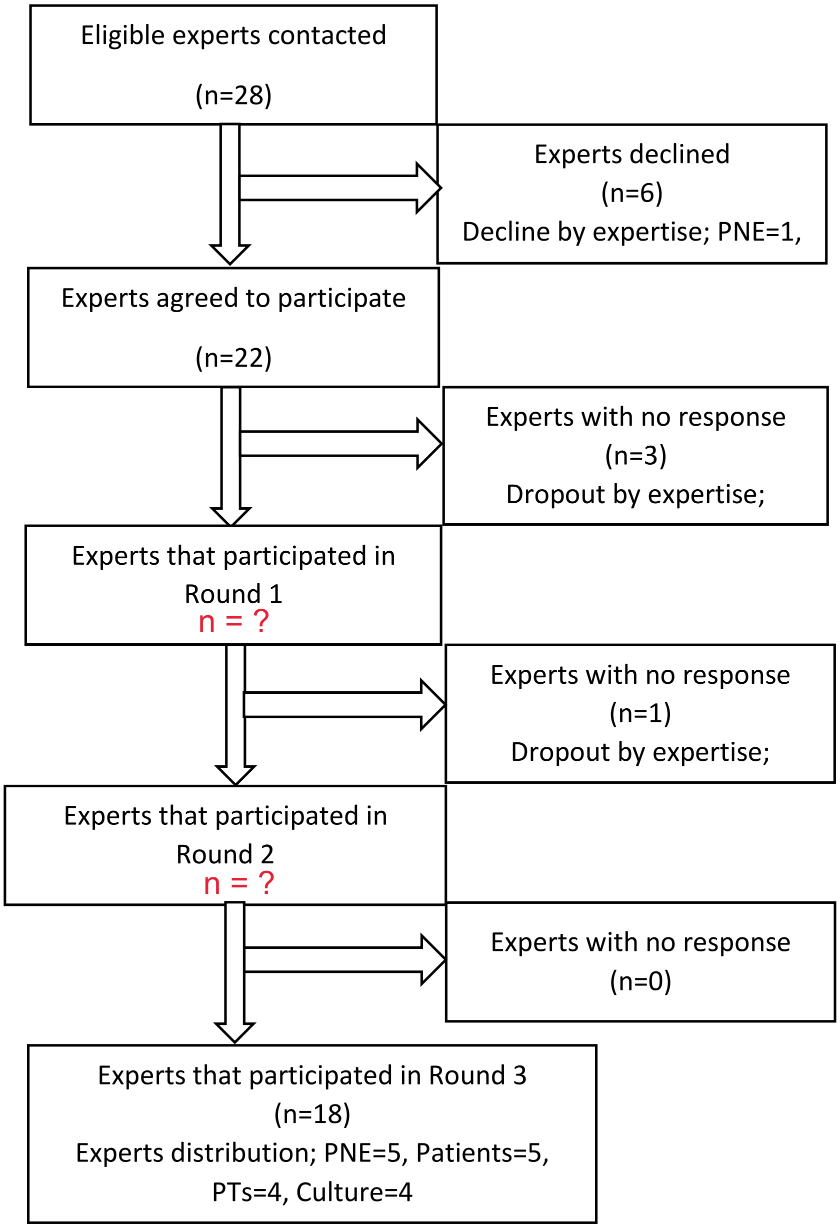

Tables

Click here to access/download
Supporting Information
Tables .docx

PNE teaching slides for males in Hausa

Click here to access/download
Supporting Information
Supplement I for Males .pdf

PNE teaching slides for females in Hausa

Click here to access/download
Supporting Information
Supplement II for Females .pdf

Home education interview audio in Hausa

Click here to access/download

Supporting Information

Supplement III home education .mp3

PNE teaching slides for males in English

Click here to access/download
Supporting Information
Supplement IV for Males-English .pdf

PNE teaching slides for females in English

Click here to access/download
Supporting Information
Supplement V for Females-English .pdf

Home education interview transcript in English

Click here to access/download

Supporting Information

Supplement VI home education English .pdf

Reviewer's Comment:

1. Replace with Hausa Speaking Patients.

I do not see the need for Africa given that the instrument can be used by a non-African who speaks and understands Hausa very well or better than other languages.

2. "significant" on page 13: Change language except you have a statistical evidence
3. "Table 5" on page 14: Referring to the wrong table, I guess
4. "Table 6" on page 14: No table 6???
5. Insert number (n = ?) for the number of experts that participated in rounds 1 and 2 as shown in Figure 1.
6. The discussion needs to be rewritten to reflect both deductive and inductive reasoning based on other published studies. The discussion appears like a repetition of the result section. This should be avoided.

1. Recommendation - Minor Revision
2. Is the manuscript technically sound, and do the data support the conclusions? Yes
3. Has the statistical analysis been performed appropriately and rigorously? Yes
4. Does the manuscript adhere to the PLOS Data Policy? I believe it does
5. Is the manuscript presented in an intelligible fashion and written in standard English? Yes
6. Review Comments to the Author - See above 6points in red font
7. Would you like your identity revealed to the authors of this submission? Yes
8. Do you have any potentially competing interests? None.
