# Supplementary material for: Development of culturally sensitive pain neuroscience education materials for Hausa-speaking patients with chronic spinal pain: A modified Delphi study
Source: PLoS One. 2021 Jul 2;16(7):e0253757. doi: 10.1371/journal.pone.0253757 (PMC8253446; doi:10.1371/journal.pone.0253757)
Supplement: Attachment — Submitted filename: Response to Reviewers_.docx [file pone.0253757.s011.docx]

January 11, 2021

Ukachukwu Okoroafor Abaraogu, BMR PT, MSc, PhD
Academic Editor
PLOSONE

Dear Academic Editor,

***Submission of revised manuscript (PONE-D-20-32518)***

First, we wish to start by expressing our profound gratitude for considering our manuscript for review in the *PLOS ONE* Journal. We also thank the reviewers who have provided very useful corrections and suggestions that have improved the overall quality of our work. It is our hope that the current revised version of the manuscript presents a better and more focused information that will be helpful to readers.

As requested, we have now provided a point by point response to the editor and reviewer(s) comments below;

**Academic editor:**

i. Please ensure that your manuscript meets PLOS ONE's style requirements, including those for file naming.

**Response:** *We have checked the manuscript once again and ensure conformity with PLOS ONE’s requirement.*

ii. Thank you for including your methods section "The research protocol was approved by the Hospital ethics committee of ‘X’ University."
    Please amend your current ethics statement to include the full name of the ethics committee/institutional review board(s) that approved your specific study, or to remove this." Once you have amended this/these statement(s) in the Methods section of the manuscript, please add the same text to the “Ethics Statement” field of the submission form (via “Edit Submission”).

**Response:** *The correction was effected in the method section and also reflected in the “Ethics Statement”.*

iii. Thank you for stating in your methods "Informed consent was obtained from the experts." Please provide additional details regarding participant consent. In the ethics statement in the Methods and online submission information, please ensure that you have specified what type of consent you obtained (for instance, written or verbal, and if verbal, how it was documented and witnessed). Please also add all of this information to your ethics statement in the online submission form.

**Response:** *Additional information concerning participants’ consent has been added in the Methods (procedure sub-section) and also reflected in the ethics statement of the online submission.*

iv. Please include additional information regarding the Delphi survey used in the study and ensure that you have provided sufficient details that others could replicate the analyses. For instance, if you developed the survey as part of this study and it is not under a copyright more restrictive than CC-BY, please include a copy, in both the original language and English, as Supporting Information.

**Response:** *We used the word “survey” to refer to the online questionnaire method that was utilized in collecting experts’ opinions. Nonetheless, for the purpose of clarity, we have now replaced the word “survey” with alternative words (Delphi-study or Delphi-rounds as the case maybe) throughout the manuscript. The link to the online questionnaires used for the Delphi-rounds are now attached as supplementary materials.*

v. We note that you have indicated that data from this study are available upon request. PLOS only allows data to be available upon request if there are legal or ethical restrictions on sharing data publicly. For information on unacceptable data access restrictions, please see

**Response:** *We have now provided the data as a supplementary attachment*

In your revised cover letter, please address the following prompts:

a) If there are ethical or legal restrictions on sharing a de-identified data set, please explain them in detail (e.g., data contain potentially identifying or sensitive patient information) and who has imposed them (e.g., an ethics committee). Please also provide contact information for a data access committee, ethics committee, or other institutional body to which data requests may be sent.

**Response:** *There are no ethical restrictions to sharing the data set of our study. The response above (item v.) also applies here.*

b) If there are no restrictions, please upload the minimal anonymized data set necessary to replicate your study findings as either Supporting Information files or to a stable, public repository and provide us with the relevant URLs, DOIs, or accession numbers. Please see <http://www.bmj.com/content/340/bmj.c181.long> for guidelines on how to de-identify and prepare clinical data for publication. For a list of acceptable repositories, please see <http://journals.plos.org/plosone/s/data-availability#loc-recommended-repositories>.

We will update your Data Availability statement on your behalf to reflect the information you provide.

6. We note that Supporting Information Figures in your submission contain copyrighted images. All PLOS content is published under the Creative Commons Attribution License (CC BY 4.0), which means that the manuscript, images, and Supporting Information files will be freely available online, and any third party is permitted to access, download, copy, distribute, and use these materials in any way, even commercially, with proper attribution. For more information, see our copyright guidelines: <http://journals.plos.org/plosone/s/licenses-and-copyright>.

**Response:** *We own the copyright of all the images used in our submission. The concept and drawing of the original images were made by our team and a contracted artist (Simen K. Lambrecht). Simen Lambrecht is a friend of one of the co-authors who graduated in Architecture. In his spare time he also draws pictures. In view of the current project described in the manuscript, he was asked to draw all the pictures for the educational material. He was paid a specific fee for each picture he made based on a contract.*

*We also intend to publish the materials on an open access website* [*http://www.paininmotion.be/*](http://www.paininmotion.be/) *for easy access by patients and researchers.*

We require you to either (1) present written permission from the copyright holder to publish these figures specifically under the CC BY 4.0 license, or (2) remove the figures from your submission:

6.1.    You may seek permission from the original copyright holder of Supporting Information Figures to publish the content specifically under the CC BY 4.0 license.

We recommend that you contact the original copyright holder with the Content Permission Form (<http://journals.plos.org/plosone/s/file?id=7c09/content-permission-form.pdf>) and the following text:

“I request permission for the open-access journal PLOS ONE to publish XXX under the Creative Commons Attribution License (CCAL) CC BY 4.0 (<http://creativecommons.org/licenses/by/4.0/>). Please be aware that this license allows unrestricted use and distribution, even commercially, by third parties. Please reply and provide explicit written permission to publish XXX under a CC BY license and complete the attached form.”

Please upload the completed Content Permission Form or other proof of granted permissions as an "Other" file with your submission.

**Response:** *We have uploaded the content permission form.*

In the figure caption of the copyrighted figure, please include the following text: “Reprinted from [ref] under a CC BY license, with permission from [name of publisher], original copyright [original copyright year].”

**Response:** *The figures are not part of the main manuscript to be published and they are numerous. We intend to publish the supplementary materials on an open access website (see response to query number 6)*

6.2.    If you are unable to obtain permission from the original copyright holder to publish these figures under the CC BY 4.0 license or if the copyright holder’s requirements are incompatible with the CC BY 4.0 license, please either i) remove the figure or ii) supply a replacement figure that complies with the CC BY 4.0 license. Please check copyright information on all replacement figures and update the figure caption with source information. If applicable, please specify in the figure caption text when a figure is similar but not identical to the original image and is therefore for illustrative purposes only.

7. Please include your tables as part of your main manuscript and remove the individual files. Please note that supplementary tables should be uploaded as separate "supporting information" files.

**Response:** *The tables are now included in the main manuscript and the individual files are now removed.*

**Response to reviewers**

**Reviewer I:**

a. The focus group discussion was not properly constituted. For me, I felt you needed a neurologist also as a panel member and some patients with pain to share their experience. This is the least you could have done, Physiotherapists alone may not be sufficiently resourceful enough to help with constructs for developing pain related education materials. Your content analysis could also have been highlighted in the appendix, for purpose of scrutiny. You mentioned that you adapted and developed, I basically find these two words very confusing. Did you adapt the material from another work or did you develop a novel material for the PNG education? Please you need to be very precise and clear in your documentations.

**Response:** *We thank the reviewer for raising these critical points.*

*Firstly, the focus group discussion was aimed at providing preliminary materials that will be sent to experts during Delphi rounds, therefore, including all experts in the focus group will amount to duplicating the Delphi study.*

*Secondly, in the current practice of PNE, neurologists are not considered experts unless they have specific expertise in PNE. In general, the concept of injury being synonymous to pain is always avoided in PNE, which maybe the belief of many physicians. Consequently, we considered physiotherapists with expertise in PNE to be enough for the purpose of developing a preliminary material, which was later subjected to Delphi rounds comprising experts.*

*Thirdly, the words adaptation and development were used in our study because, as mentioned in the method section, we adapted the pain teaching materials from an existing PNE materials (Orhan et al. cited in our method section), and we also developed a novel interview aspect that has not been in existence in the PNE field. Nevertheless, we have included some modification in the method section for better clarity.*

*Finally, part of the content analysis is provided for better understanding (see Table 3 and Table 4 for some of the contents analysed and the decisions taken by the authors)*

b. You had a focus group discussion in which you developed a preliminary PNG and HE education material in English language and later translated these materials to Hausa language. For me, your translation and cultural adaptation was very substandard and shallow. Translating research materials and cultural adaptation has a standard procedure there are basically six stages which must be compulsorily adapted to make such acceptable in standard research.
   STAGE 1:initial translation(Two forward translation by at least two bilingual translators they should have different profile background)
   STAGE 2:Synthesis of translation
   STAGE 3:Back translation
   STAGE 4:Expert committee
   STAGE 5:Test of the pre-final version.
   STAGE 6:Submission and appraisal of all written reports by developers/committee.
   All these important steps were not followed, these therefore makes your translation highly sub-standard and unacceptable for a research of these magnitude.
   The processes of translation and cultural adaptation are very central in this work and less attention was paid on it but rather you spent all your time discussing the audit trail which was the Delphi study. The Delphi study is important but the main crust of this work is the translation to Hausa language.
   I think you need to do a major revision of this work to make it standard and acceptable, its indeed a good and nice innovation but it must be done to international standard to make it acceptable for publication.

**Response:** *We thank the reviewer for raising this important issue. First, the aim of our study is to develop a culture-sensitive material. Therefore, the focus of our study is not on translation and cultural adaptation of an existing material. As such, a translation and cultural adaptation procedure is not reflected in our study.*

*Second***,** *the composition of the experts that participated in our study was not homogenous (English speaking and Hausa speaking). Hence, we had to first developed a preliminary material in English language through a focus group, since we lack PNE experts that are Hausa speaking. And it was these materials that were then translated through an acceptable procedure. Moreover, since the materials had to be subjected to Delphi rounds and it was expected that there will be changes based on the comments of experts after each round, translating the material via the method mentioned above may not be practical or feasible, and will not permit for the development of the tool through a Delphi procedure. Furthermore, a similar procedure has been used for the development of PNE materials in a previous study (Orhan et al -cited in our methods section). If the translation procedure suggested by the reviewer will be followed, then after each round of the Delphi we have to make similar translation which will be counterproductive and my encourage dropouts among our experts.*

**Reviewer II:**

1. Replace with Hausa Speaking Patients.
   I do not see the need for Africa given that the instrument can be used by a non-African who speaks and understands Hausa very well or better than other languages.

**Response:** *We thank the reviewer for the valuable suggestions. Hausa speaking patients is now reflected throughout the manuscript. We have also removed African in reference to the tool we developed throughout the manuscript including the title.*

2. "significant" on page 13: Change language except you have a statistical evidence

**Response:** *The word significant has been replaced with substantial*

3. "Table 5" on page 14: Referring to the wrong table, I guess

**Response:** *We have previously collapsed Table 5 and 6 together, which earlier interfered with or Table numbering, but we have now effected the corrections.*

4. "Table 6" on page 14: No table 6???

**Response:** *Noted and corrected* (*As responded on item 3 above)*

5. Insert number (n = ?) for the number of experts that participated in rounds 1 and 2 as shown in Figure 1.

**Response:** *The ‘n’ has been reflected in the Figure. We lost the ‘n’ in the earlier submission due to inappropriate box-zooming.*

6. The discussion needs to be rewritten to reflect both deductive and inductive reasoning based on other published studies.

**Response:** *There are limited number of studies published for PNE tool development using a qualitative approach, hence we had to discuss our findings based on the existing studies including the peculiarities and novelties of our study. However, we made sure that the discussion section is improved upon, as much as possible, after also taking into account the lack of published studies in the same field adopting similar methods.*
