# Supplementary material for: Development of culturally sensitive pain neuroscience education materials for Hausa-speaking patients with chronic spinal pain: A modified Delphi study
Source: PLoS One. 2021 Jul 2;16(7):e0253757. doi: 10.1371/journal.pone.0253757 (PMC8253446; doi:10.1371/journal.pone.0253757)
Supplement: Attachment — Submitted filename: LETTER TO AUTHORS.doc [file pone.0253757.s012.doc]

**LETTER TO AUTHORS**

1. Having the knowledge of PNG education for me is an added advantage for the members of the preliminary focus group. That does not replace the issues of professionalism and training. By training the anesthetist is the specialist in pain management and matters, I only mentioned a neurologist in my initial comment. Preparing the preliminary material for any research is crucial and should not be trivialized. Having a panel for Delphi study does not necessary mean that they are to do the initial work of developing, their role is to modify the document. Saying that getting the right people for the preliminary document will be a waste of time is very wrong. I would rather suggest you have it as a limitation, stating that the initial document was developed only by physiotherapist with PNG education knowledge, and that no other pain specialist and professionals was involved in the initial focus group discussion. You should also note that because ***Orhan et al*** used and cited it in their work does not make the pattern a golden standard.
2. Can I ask you to kindly provide the specialties of your panelist for the Delphi study for our perusal? Pushing all responsibilities to the panelist to me may not be fair, is any of our panelist an expert in Hausa language by training? Kindly note that the six stages of translation I mentioned to you are standard and internationally accepted. I would rather suggest that you acknowledge also as a limitation your not following the process due to lack of personnel than trying to explain it away. Citing ***Orhan et al*** is not sufficient, are they experts in translation and instrumentation? Saying that your work is not focused on translation is not correct, you set out to develop a culturally sensitive pain neuroscience education material for Hausa speaking patients. The translation aspect is equally important.Goodluck.
