# Supplementary material for: Development of culturally sensitive pain neuroscience education materials for Hausa-speaking patients with chronic spinal pain: A modified Delphi study
Source: PLoS One. 2021 Jul 2;16(7):e0253757. doi: 10.1371/journal.pone.0253757 (PMC8253446; doi:10.1371/journal.pone.0253757)
Supplement: Attachment — Submitted filename: Response to Reviewers .docx [file pone.0253757.s013.docx]

May 20, 2021

Ukachukwu Okoroafor Abaraogu, BMR PT, MSc, PhD
Academic Editor
PLOSONE

Dear Academic Editor,

***Submission of re-revised manuscript (PONE-D-20-32518)***

We wish to express our profound gratitude for considering our revised manuscript in the *PLOS ONE* Journal and recommending a minor revision. We also thank the reviewers who have gone through the revised manuscript and have provided their feedbacks that have improved the overall quality of our work. It is our hope that, after this additional revision, the current version of the manuscript presents a better and more focused information that will be helpful to readers.

As requested, we have now provided a point by point response to the Reviewer I

**Reviewer I:**

1. Having the knowledge of PNG education for me is an added advantage for the members of the preliminary focus group. That does not replace the issues of professionalism and training. By training the anesthetist is the specialist in pain management and matters, I only mentioned a neurologist in my initial comment. Preparing the preliminary material for any research is crucial and should not be trivialized. Having a panel for Delphi study does not necessary mean that they are to do the initial work of developing, their role is to modify the document. Saying that getting the right people for the preliminary document will be a waste of time is very wrong. I would rather suggest you have it as a limitation, stating that the initial document was developed only by physiotherapist with PNG education knowledge, and that no other pain specialist and professionals was involved in the initial focus group discussion. You should also note that because ***Orhan et al*** used and cited it in their work does not make the pattern a golden standard.

**Response:** *We thank the reviewer for raising these critical points and we totally agree with the reviewer. We have now updated our limitation section and included the lack of other professionals in pain management as a limitation for the focus group discussion.*

2. Can I ask you to kindly provide the specialties of your panelist for the Delphi study for our perusal? Pushing all responsibilities to the panelist to me may not be fair, is any of our panelist an expert in Hausa language by training? Kindly note that the six stages of translation I mentioned to you are standard and internationally accepted. I would rather suggest that you acknowledge also as a limitation you’re not following the process due to lack of personnel than trying to explain it away. Citing ***Orhan et al*** is not sufficient, are they experts in translation and instrumentation? Saying that your work is not focused on translation is not correct, you set out to develop a culturally sensitive pain neuroscience education material for Hausa speaking patients. The translation aspect is equally important.

**Response:** *We thank the reviewer for raising these important issues as well. The expertise of the Delphi panel members has been presented under the ‘Delphi panel experts’ from pages 160 to 170. None of the panel experts was a Hausa language expert (by training). However, we have identified our inability to translate the developed materials using standard translation procedure as a limitation for the study (see limitations). Moreover, we have also presented the qualifications, and acknowledged the Hausa language experts involved in the translation of the materials (see limitations and acknowledgments).*
